# Tetragonal phosphorus(v) cations as tunable and robust catalytic Lewis acids†
†Electronic supplementary information (ESI) available. CCDC 1904833 and 1904834. For ESI and crystallographic data in CIF or other electronic format see DOI: 10.1039/c9sc02463h


**DOI:** 10.1039/c9sc02463h

**Published:** 2019-06-18

**Authors:** James C. Gilhula, Alexander T. Radosevich

**Affiliations:** a Department of Chemistry , Massachusetts Institute of Technology , Cambridge , MA 02139 , USA . Email: radosevich@mit.edu

## Abstract

The synthesis and catalytic reactivity of a class of water-tolerant cationic phosphorus-based Lewis acids is reported. Corrole-based phosphorus(v) cations of the type [ArP(cor)][B(C_6_F_5_)_4_] (Ar = C_6_H_5_, 3,5-(CF_3_)_2_C_6_H_3_; cor = 5,10,15-(C_6_H_5_)_3_corrolato^3–^, 5,10,15-(C_6_F_5_)_3_corrolato^3–^) were synthesized and characterized by NMR and X-ray diffraction. The visible electronic absorption spectra of these cationic phosphacorroles depend strongly on the coordination environment at phosphorus, and their Lewis acidities are quantified by spectrophotometric titrations. DFT analyses establish that the character of the P-acceptor orbital comprises P–N antibonding interactions in the basal plane of the phosphacorrole. Consequently, the cationic phosphacorroles display unprecedented stability to water and alcohols while remaining highly active and robust Lewis acid catalysts for carbonyl hydrosilylation, C_sp^3^_–H bond functionalization, and carbohydrate deoxygenation reactions.

## Abstract 2

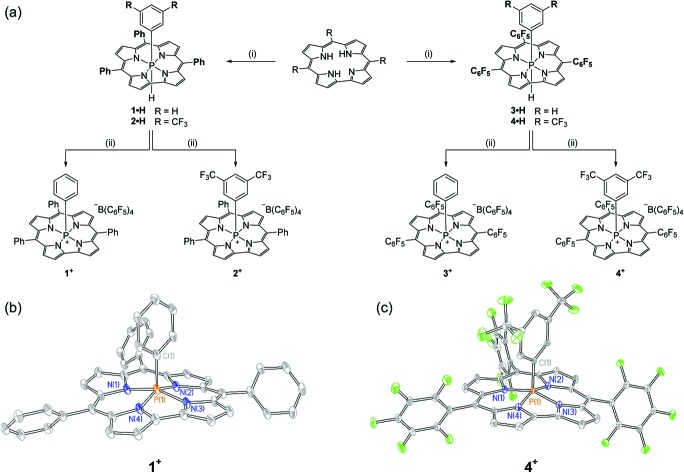
The synthesis and catalytic reactivity of a class of water-tolerant cationic phosphorus-based Lewis acids is reported.

## Introduction

Tetracoordinate phosphonium cations (R_4_P^+^) are electrophilic at the phosphorus center, a fact that can be leveraged in Lewis acid catalysis.^1^ Stephan reported a major innovation in this area by showing that fluorophosphonium cations (R_3_P^+^–F) display an exceptionally low-lying σ*(P–F) orbital that is accessible to even weak Lewis bases.^2^ Tailoring of the fluorophosphonium core with electron-withdrawing substituents (*i.e.* (per)fluoroarenes, *N*-alkylpyridiniums) accentuates the *P*-electrophilicity of the fluorophosphonium, permitting access to compounds with fluoride affinities exceeding that of SbF_5_, the benchmark Lewis acid.^3^ A family of compositionally diverse electrophilic phosphonium Lewis acids is now known,^2^ and work from Gabbaï has shown that the underlying design principle is portable to heavier group 15 Lewis acids.^4^

**Scheme 1:**
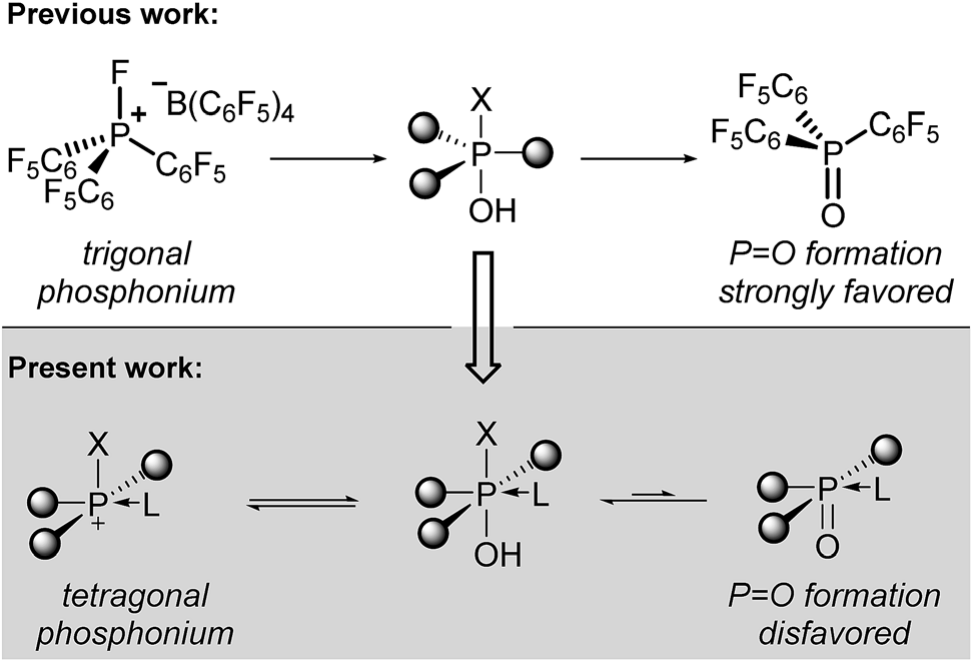
(top) Electrophilic phosphonium cations and their hydrolytic decomposition to phosphine oxides. (bottom) Notional tetragonal electrophilic phosphonium cations that enhance robustness but preserve Lewis acidity.

Coinciding with their exceptional electrophilic character, fluorophosphonium cations display a marked sensitivity to water and hydroxylic functionality.^2^ Hydrolysis of the fluorophosphonium P–F bond results in decomposition by irreversible formation of a thermodynamically stable phosphine oxide (R_3_P=O) in which the *P*-centered electrophilicity is effectively quenched (Scheme 1, top). A reduced hydrolytic sensitivity of certain (trifluoro)methylphosphonium Lewis acids has recently been achieved by Ingleson and Stephan.^5^ Additionally, Stephan has developed air-stable P(iii) dicationic Lewis acids.^6^

We considered that an alternate approach to robust phosphorus-based Lewis acids might be accessible by deliberate alteration of the molecular geometry. Having previously demonstrated that molecular deformation of neutral phosphorus compounds allows for novel bond activation reactions and catalytic transformations,^7^ we reasoned that the undesirable hydrolysis pathway leading to inactive phosphine oxides for electrophilic phosphonium cations might be avoided by enforcing *nontrigonal* substitution at phosphorus. Specifically, we envisioned that a *tetragonal* substituent field would be less conducive to formation of a formal P=O multiple bond, diminishing the propensity for phosphine oxide formation and thereby preserving the Lewis acidity at the cationic phosphorus center (Scheme 1, bottom). Indeed, Kadish and Vogel^8^ and Ravikanth^9^ have demonstrated that square pyramidal phosphine oxides embedded within a corrole binding pocket have a propensity to form hexacoordinate structures by association of an exogenous nucleophile. Moreover, it has been shown that phosphorus(v) corroles readily undergo apical halide/alkoxide exchange^10^ and are even stable in aqueous media.^11^

We demonstrate here that square pyramidal corrole-based phosphorus(v) cations are robust, tunable, and catalytically-active Lewis acids. We find that the rigid tetragonal geometry imparts stability to water and alcohols while maintaining Lewis acidity, enabling these compounds to effect transformations which were previously inaccessible to phosphonium catalysts. This desirable superposition of properties is rationalized within an electronic structure argument that advances our ongoing program to establish new reactivity for p-block elements by imposition of underexplored molecular geometries.

## Results and discussion

**Fig. 1:**
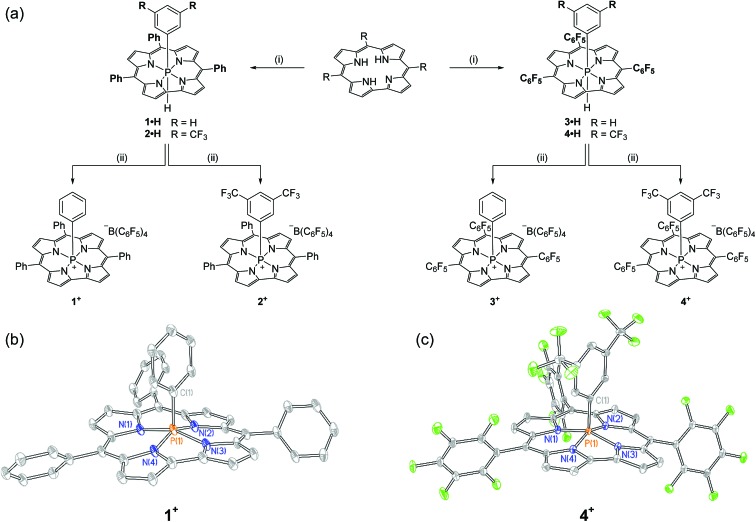
(a) Synthetic path to phosphacorroles **1^+^–4^+^**. (i) PhPCl_4_ or 3,5-(CF_3_)_2_C_6_H_3_PCl_4_, Et_3_N, PhMe, Δ, 1 h; [Bu_4_N][BH(OAc)_3_], PhMe, RT, overnight (ii) [Ph_3_C][B(C_6_F_5_)_4_], CH_2_Cl_2_, RT, 5 min. (b) X-ray structure of **1^+^**. Counterions and hydrogen atoms are omitted for clarity. Thermal ellipsoids are rendered at the 50% probability level. Selected bond lengths [Å], angles [°], and dihedrals [°]: P(1)–N_avg_ 1.804(6), P(1)–C(1) 1.819(3), N(1)–P(1)–N(3) 158.1(1), N(2)–P(1)–N(4) 152.9(1), N(1)–N(2)–N(3)–N(4) –3.38(1). (c) X-ray structure of **4^+^**. Counterions and hydrogen atoms are omitted for clarity. Thermal ellipsoids are rendered at the 50% probability level. Selected bond lengths [Å], angles [°], and dihedrals [°]: P(1)–N_avg_ 1.792(3), P(1)–C(1) 1.817(2), N(1)–P(1)–N(3) 158.21(7), N(2)–P(1)–N(4) 155.48(2), N(1)–N(2)–N(3)–N(4) 1.90(1).

Synthesis of the target cations was achieved in two steps from the freebase corrole. First, treatment of 5,10,15-triphenylcorrole with 1 equiv. of phenyl tetrachlorophosphorane (PhPCl_4_) in the presence of triethylamine furnished an unstable intermediate, which upon the addition of [Bu_4_N][BH(OAc)_3_] yielded hexacoordinate **1·H** as a chromatographically stable green solid (Fig. 1a). The ^31^P{^1^H} NMR spectrum of **1·H** showed a resonance at high field (*δ* – 231.3 ppm), consistent with compositionally similar hexacoordinate phosphorus compounds reported previously.^8^–^11^ The proton-coupled ^31^P NMR resonance evolves into a doublet of triplets, with coupling constants evidencing a direct P–H bond (^1^*J*_P–H_ = 928.0 Hz) as well as longer range coupling to the *ortho* protons of the apical *P*-aryl moiety (^3^*J*_P–H_ = 25.1 Hz).^13^ In the ^1^H NMR channel, the P–H unit was observed with complementary coupling (*δ* – 2.73 ppm, d, ^1^*J*_P–H_ = 928.8 Hz); the rather high-field chemical shift of this ^1^H nucleus is attributed to shielding from the diamagnetic ring current of the corrole system,^14^ securing the assignment of the structure of **1·H** as in Fig. 1.

The apical hydride of hexacoordinate compound **1·H** is readily removed by treatment with abstraction reagents. Specifically, a green solution of **1·H** in CH_2_Cl_2_ when treated with 1 equiv. of [Ph_3_C][B(C_6_F_5_)_4_] immediately produced a red solution, from which a new phosphorus-containing product was obtained by precipitation *via* slow addition of pentane. A ^31^P NMR spectrum of the resulting maroon solid displayed a single new triplet resonance downfield of the starting compound (*δ* – 97.2 ppm, *t*, ^3^*J*_P–H_ = 20.6 Hz). This chemical shift is indicative of a pentacoordinate phosphorus center shielded by diamagnetic ring current;^8^,^9^,10b,^15^ loss of ^1^*J*_P–H_ coupling and concomitant formation of triphenylmethane further evidence the formation of the hydride abstraction product **1^+^**. Related phosphacorroles **2^+^–4^+^** were synthesized analogously from the corresponding triarylcorrole and aryl phosphorane as depicted in Fig. 1.

The solid state structure of **1^+^** (as its triflate salt) was revealed by X-ray diffraction experiments (Fig. 1b). As expected, the rigid constraint imposed by the corrole ligand framework enforces a local geometry closely resembling a square pyramid (*τ* = 0.09),^16^ where the phosphorus center projects 0.373 Å out of the plane containing the four pyrrolic nitrogen atoms.^17^ Fluorinated congener **4^+^** (Fig. 1c) similarly exhibits a near-perfect square pyramidal geometry (*τ* = 0.05)^16^ where the *P* center protrudes less from the binding pocket relative to **1^+^** (Δ*d* = 0.023 Å). In both instances, the overall geometry imposed by the corrole ligand may be viewed as a monovacant octahedron about phosphorus. In conjunction with structural data for known hexacoordinate phosphorus corrole compounds (where the phosphorus atom is essentially coplanar with the tetrapyrrolic nitrogens^8^–^11^), association of Lewis bases to the apical site could be anticipated to proceed with a rather small energy penalty for structural reorganization.

The affinity of cationic phosphacorroles **1^+^–4^+^** for Lewis bases was initially assayed by recording ^31^P NMR chemical shift differences (Δ*δ*) for a phosphine oxide ((*n*-octyl)_3_P=O) probe upon binding according to a modification of the Gutmann–Beckett method.^18^,^19^ In agreement with expectations based on inductive substituent effects, the Δ*δ* values (Table 1) report a self-consistent picture of the increasing Lewis acidity (**1^+^** < **2^+^** < **3^+^** < **4^+^**) as a function of increasing modular fluorination. A direct comparison of the Lewis acidity of **1^+^–4^+^** to other Lewis acids on the basis of these Δ*δ* values is tempting, but we caution against such a potentially specious interpretation in the present circumstance. In view of the diamagnetic ring current of the corrole moiety,^14^ a phosphine oxide probe bound apically to the phosphacorrole cation would experience shielding effects that would tend to produce anomalously small Δ*δ* values. Other NMR-based methods for the determination of Lewis acidity (Childs,^20^ Hilt^21^) would similarly be expected to show a systematic underestimate of Lewis acidity for porphyrinoid-based Lewis acids like **1^+^–4^+^**.^22^

**Table 1:** Summary of important metrics for phosphacorroles **1^+^–4^+^**

Compound	^31^P *δ* (ppm)^*a*^	*ε*_LUMO+N_ (eV)^*b*^	FIA (kJ mol^–1^)^*c*^	GEI (eV)^*d*^	Δ*δ* (ppm)^*a*^ ^,^^*e*^	*K*_d_ (μM)^*f*^
**1^+^**	–97.1	–1.47	274	3.75	2.0	2660 ± 90
**2^+^**	–102.2	–1.72	295	3.90	13.6	30.1 ± 0.7
**3^+^**	–95.2	–1.85	298	4.23	15.3	26 ± 1
**4^+^**	–100.3	–2.31^*g*^	343^*g*^	4.53^*g*^	21.3	11.3 ± 0.4

^*a*^Chemical shift externally referenced to 85% H_3_PO_4_. Spectra recorded in CD_2_Cl_2_ at 293 K.

^*b*^Computed at the B3LYP/def2-TZVP/CPCM(CH_2_Cl_2_)//B3LYP/def2-TZVP level. The orbital with appropriate symmetry was LUMO+*N*, where *N* = 3 (**1^+^**), 4 (**2^+^**), 2, (**3^+^**), 3 (**4^+^**).

^*c*^Computed according to Christe's pseudoisodesmic method^28^ at the B3LYP/def2-TZVP/CPCM(CH_2_Cl_2_)//B3LYP/def2-TZVP level.

^*d*^Computed at the B3LYP/def2-TVZP/CPCM(CH_2_Cl_2_)//B3LYP/def2-TZVP level as described by Stephan *et al.*^12^

^*e*^Change in ^31^P NMR chemical shift of (*n*-octyl)_3_P=O.

^*f*^Measurements are bracketed by one standard error.

^*g*^Values obtained at the B3LYP/def2-TZVP/CPCM(CH_2_Cl_2_)//B3LYP/def2-SVP level of theory.

**Fig. 2:**
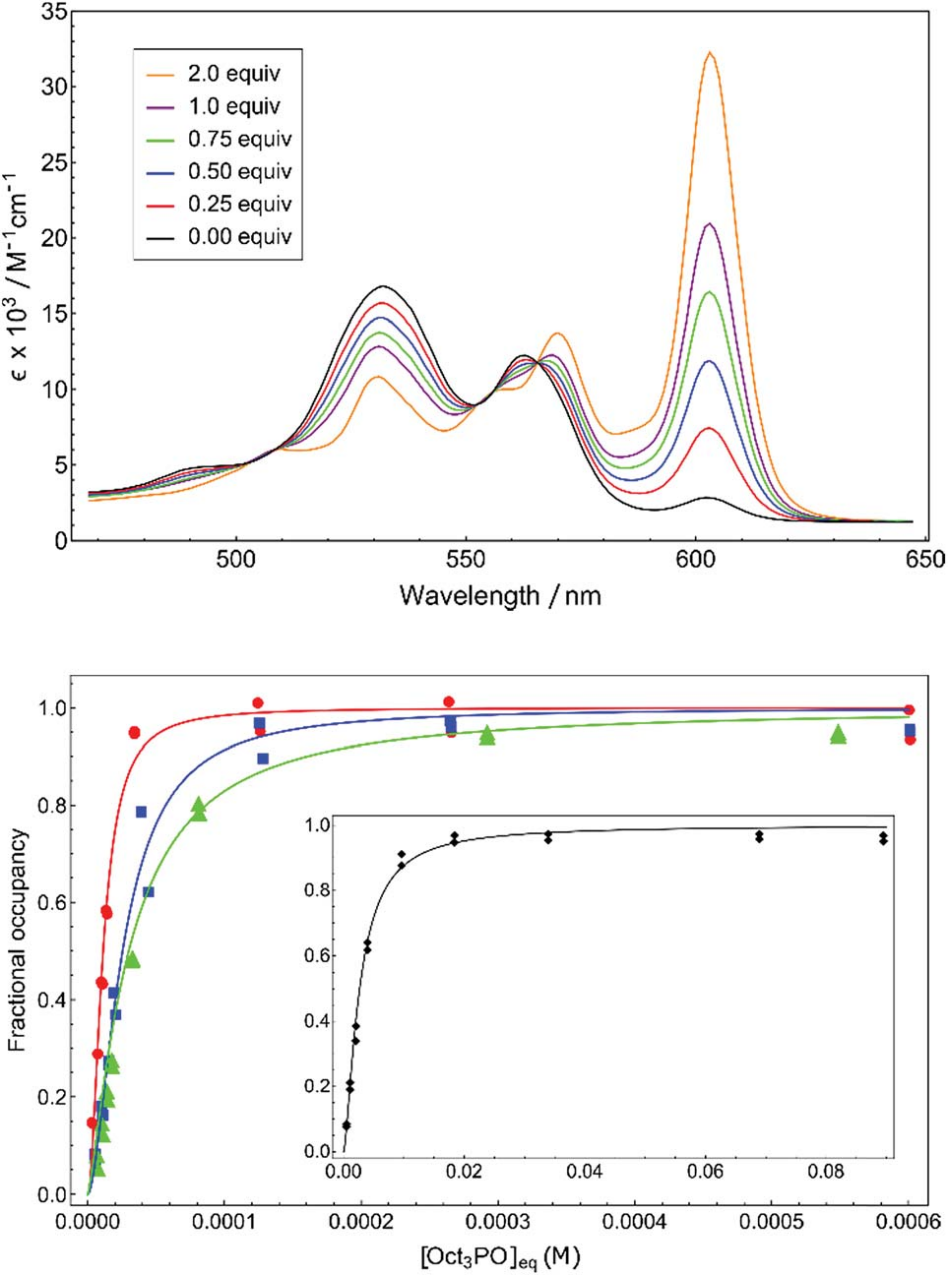
(top) Q-band region of **4^+^** upon titration with 0 to 2 equiv. of (*n*-octyl)_3_P=O in CH_2_Cl_2_. (bottom) Binding isotherms for (*n*-octyl)_3_P=O with **4^+^** (red, circle), **3^+^** (blue, square), **2^+^** (green, triangle), and (inset) **1^+^** (black, diamond).

A unbiased quantification of Lewis acidity for **1^+^–4^+^** is given by the binding dissociation constant (*K*_d_). The marked difference in color between cationic five-coordinate phosphacorroles (red) and neutral six-coordinate congeners (green) provided a convenient colorimetric method for measuring equilibrium binding in **1^+^–4^+^**. The sensitivity of the color dependence to the concentration of an exogenous Lewis base was demonstrated by titrating cationic phosphacorrole **4^+^** with varying amounts of (*n*-octyl)_3_P=O (Fig. 2, top). The presence of several isosbestic points (*e.g. λ* = 566 nm) confirms adduct formation free of decomposition or other deleterious reactivity.

The isotherms obtained by monitoring absorption at 610 nm with increasing amounts of (*n*-octyl)_3_P=O were fitted to the Hill equation^23^ to obtain equilibrium dissociation constants *K*_d_ (Table 1, see ESI† for full details). The micromolar dissociation constants confirm the pronounced affinity of the cationic phosphacorroles for phosphine oxide Lewis bases and afford an intrinsic thermodynamic parameter of Lewis acidity for this chemotype.

To further understand the varying Lewis acidities of **1^+^–4^+^**, DFT calculations of the electronic structure of these cationic phosphacorroles were performed. The wavefunctions of **1^+^–4^+^** were computed at the B3LYP/def2-TZVP/CPCM(CH_2_Cl_2_)//B3LYP/def2-TZVP level^24^ as implemented in the ORCA 4.0.0 software package,^25^ and are found to resemble experimental structures closely (see ESI† for full details). In excellent agreement with previous theoretical studies on isolobal Ga(iii) corroles,^26^ the LUMO and LUMO+1 of **1^+^–4^+^** correspond to the corrole π manifold, and are apparently not responsible for the experimentally observed Lewis acidity of cationic phosphacorroles.

**Fig. 3:**
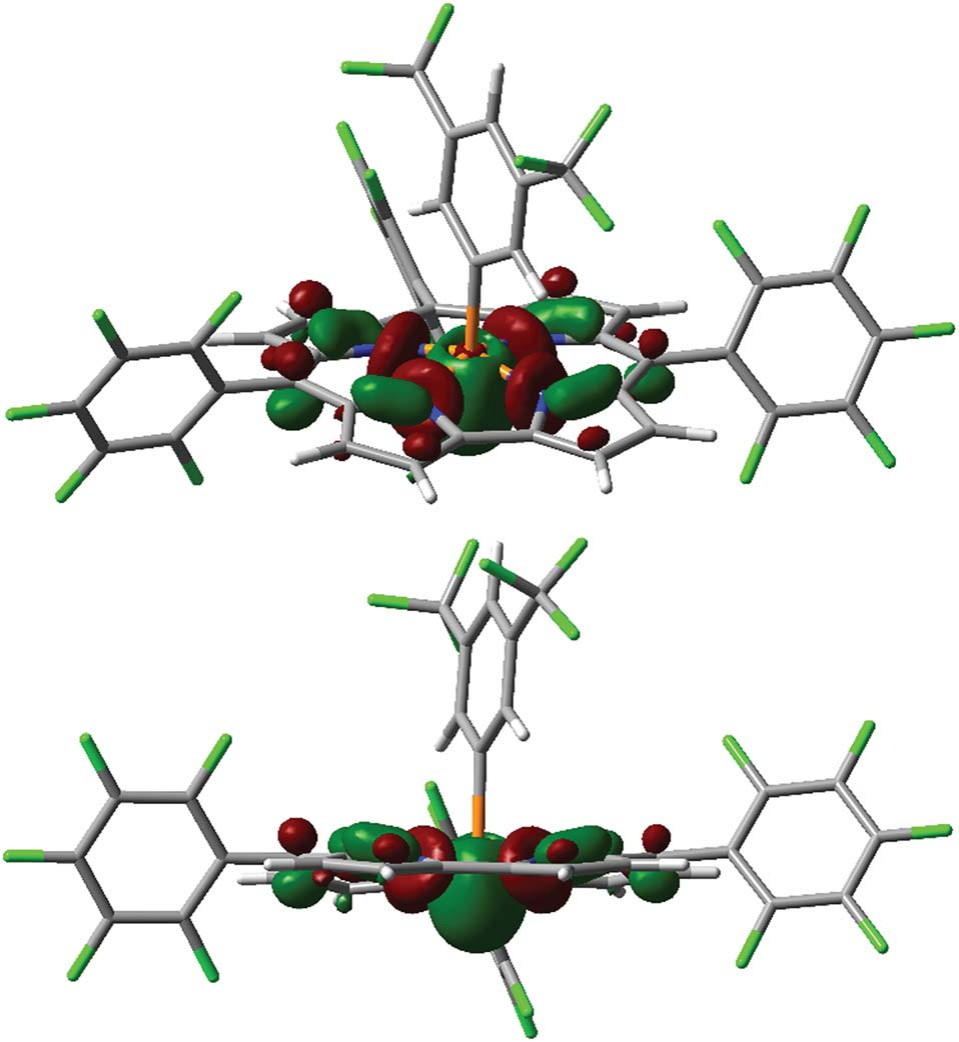
Kohn–Sham orbital LUMO+3 for **4^+^** (top: in perspective; bottom: side-on view) computed at the B3LYP/def2-TZVP//B3LYP/def2-SVP level.

Another low-lying unoccupied orbital orbital (LUMO+3 for **4^+^**, Fig. 3)^27^ is still quite low in energy (*e.g.* –2.31 eV for **4^+^**, see Table 1) and projects into the apical space proximal to phosphorus, rendering it both energetically and sterically accessible for attack by exogenous nucleophiles. Indeed, the calculated fluoride ion affinities^28^ (FIAs) for **1^+^–4^+^** correlate with the experimental dissociation constants *K*_d_ for phosphine oxide binding, implying that Lewis acid/base interactions are hosted by this orbital (see ESI† for details). Most notably, the character of this P-acceptor orbital illustrates a fundamental distinction with respect to prior phosphonium Lewis acids that has important implications for their stability. Whereas the acceptor orbital for *trigonal* electrophilic phosphonium cations is σ-antibonding with respect to the *trans*-apical substituent in the developing Lewis acid/base adduct,^2^ the phosphorus-centered acceptor orbital in *tetragonal* phosphorus cations **1^+^–4^+^** primarily constitutes basal P–N antibonding interactions. As a consequence, the mechanistic pathway initiated by water addition which leads to apical ejection of a P-substituent for trigonal phosphoniums^29^ is denied by the corrole chelate. On this basis, we anticipated that tetragonal cations **1^+^–4^+^** might be relatively resistant to decomposition by water.

**Scheme 2:**
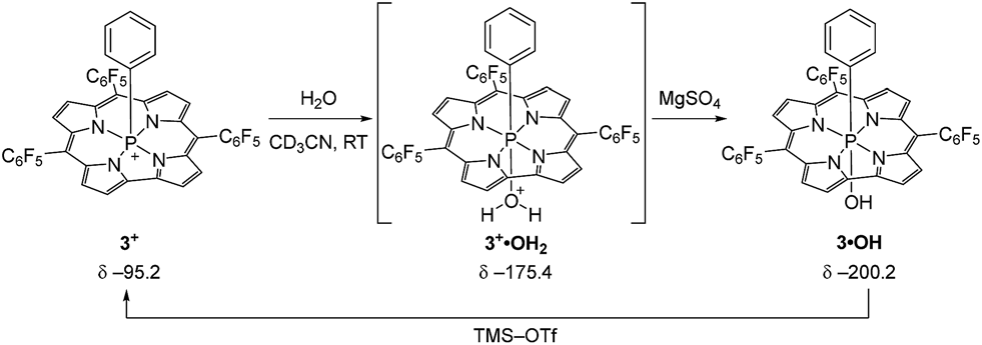
Reaction of water with **3^+^** and formation of putative water adduct **3^+^·OH_2_**. Deprotonation by MgSO_4_ gives product **3·OH**, which is reactivated to **3^+^** by TMS–OTf.

In order to probe this conjecture, we investigated the chemical stability of **1^+^–4^+^** with respect to hydrolysis. In a representative experiment (Scheme 2), treatment of a red CD_3_CN solution of **3^+^** with 10 equiv. of water resulted in a purple-green solution whose ^31^P{^1^H} NMR spectrum exhibited a broad resonance at *δ* – 175.4 ppm. The chemical shift is consistent with a six-coordinate P atom, and the broadness of the peak suggests reversible binding to the Lewis acidic phosphorus. Importantly, there is no evidence of decomposition of this intermediate upon prolonged standing. Although this adduct has thus far eluded isolation, the addition of even a weak base (MgSO_4_) cleanly gives *P*-hydroxide **3·OH** (*δ* – 200.2 ppm) in quantitative fashion (see ESI† for details) without further *in situ* transformation. Moreover, the active cationic phosphacorrole **3^+^** may be regenerated from **3·OH** by treatment with trimethylsilyl trifluoromethanesulfonate (TMS–OTf) as in Scheme 2. Consequently, we believe that reaction with water does not irreversibly decompose the Lewis acidic phosphacorrole **3^+^** but instead gives an adduct that we formulate as **3^+^·OH_2_** (Scheme 2). The same reasoning may be extended by analogy to **1^+^**, **2^+^**, and **4^+^**.

**Scheme 3:**
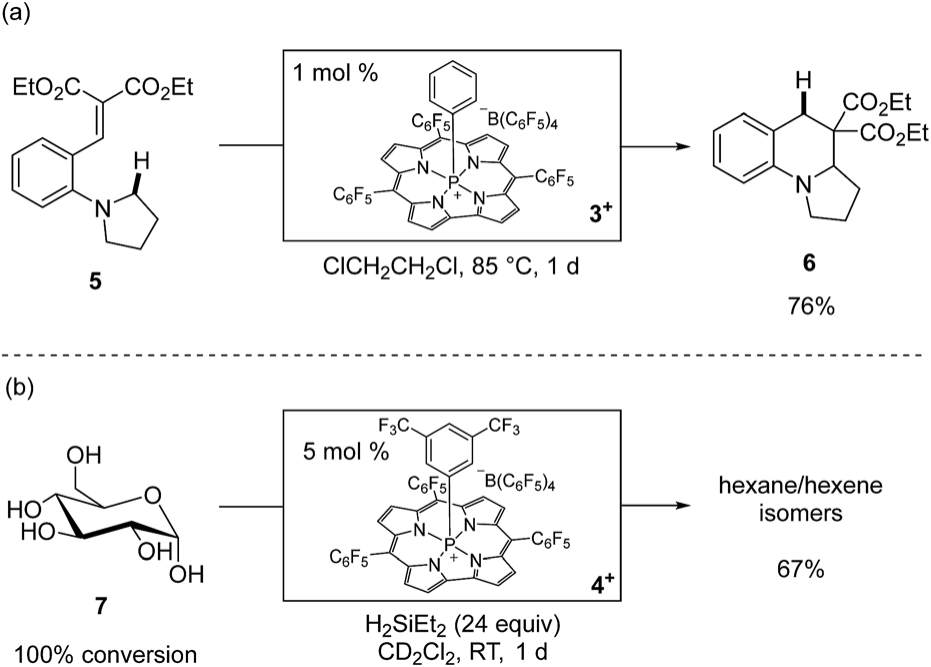
(a) Reaction was performed on a 1.0 mmol scale. Isolated yield is reported. (b) Reaction was performed with 0.1 mmol of ^13^C_6_-glucose. Quantitative ^13^C NMR yields are reported. The ratio of products is 27 : 22 : 14 : 4 *n*-hexane : 3-methylpentane : 2-methylpentane : hexenes.

The phosphacorrole cations **1^+^–4^+^** are potent Lewis acid catalysts for a range of transformations.^30^ We benchmarked the catalytic activity of **1^+^** and **2^+^** with typical carbonyl reduction reactions, such as ketone hydrosilylation and deoxygenation using HSiEt_3_ as a terminal reductant (see ESI† for full details). The high activity of **1^+^** and **2^+^** for these reductions encouraged us to attempt more challenging catalytic transformations. For instance, ring-forming C_sp^3^_–H functionalization of substrate **5** is induced by catalytic **3^+^** (1 mol%) *via* 1,5-hydride shift from a *N*,*N*-dialkylaniline donor to a malonate alkylidene acceptor followed by intramolecular cyclization to give **6** in good isolated yield (Scheme 3a).^31^ Furthermore, the robustness of the tetragonal cationic phosphorus corrole Lewis acids is exemplified by the observation that unprotected ^13^C_6_-d-glucose (**7**) is exhaustively deoxygenated by a catalytic amount of **4^+^** (5 mol%) in the presence of an excess of H_2_SiEt_2_ at room temperature to give a mixture of hexanes and hexenes (67% total yield, Scheme 3b).^32^ Notably, analysis after completion of the reaction shows that **4^+^** is not degraded; instead, an approximately equimolar amount of **4·H** and **4^+^** were observed spectroscopically as the only ^31^P NMR resonances. The lack of reaction between **4^+^** and H_2_SiEt_2_ in a control experiment further indicated that **4^+^** is a true catalyst for this reaction. The persistence of **4^+^** with respect to a substrate that under these conditions presents a 100 : 1 ratio of free hydroxyl moieties to catalyst confirms the noteworthy chemical robustness inherent to tetragonal cationic phosphorus-based Lewis acids.

## Conclusions

In summary, we have shown that cationic phosphacorroles are potent Lewis acids that exhibit marked tolerance toward hydroxylic functionality including water. We propose that this useful property arises from the tetragonal geometry of **1^+^–4^+^** as enforced by the tetraazamacrocycle, which produces an acceptor orbital that is distinct in character from prior trigonal electrophilic phosphonium cations and prohibits irreversible decomposition to phosphine oxides. The modularity of the corrole supporting framework allows the Lewis acidities of these electrophilic phosphorus species to be readily tuned. Together, these results establish within the family of designer main group Lewis acids a new structural type that extends the range of potential applications for this valuable class of compounds.

## Conflicts of interest

There are no conflicts to declare.

## Supplementary Material

Supplementary information

Crystal structure data
