# Supplementary material for: Tetragonal phosphorus(v) cations as tunable and robust catalytic Lewis acids
Source: Chem Sci. 2019 Jun 18;10(30):7177–82. doi: 10.1039/c9sc02463h (PMC6685354; doi:10.1039/c9sc02463h)
Supplement: Supplementary file 1 [file SC-010-C9SC02463H-s001.pdf]

Electronic Supplementary Material (ESI) for Chemical Science.
This journal is © The Royal Society of Chemistry 2019

Tetragonal Phosphorus(V) Cations: Tunable and Robust Catalytic Lewis Acids

James C. Gilhula and Alexander T. Radosevich*

Department of Chemistry
Massachusetts Institute of Technology
Cambridge, MA 02139
E-mail: radosevich@mit.edu

Contents

1. General procedures ... 2
2. Synthesis of starting materials, **1•H–4•H**, and **1**$^{+}$–**4**$^{+}$ ... 2
3. Gutmann-Beckett tests for **1**$^{+}$–**4**$^{+}$ ... 9
4. UV-vis spectrophotometric titrations ... 9
5. Water stability test for **3**$^{+}$ ... 10
6. Experimental procedures for catalytic transformations ... 10
7. Heteronuclear NMR spectra ... 12
8. UV-VIS spectra ... 67
9. Mass spectra ... 76
10. X-Ray structures ... 84
11. DFT calculations ... 124
12. References ... 139

1. General procedures

Dichloromethane and toluene used for reaction chemistry were purified and collected under argon using a Glass Contour Solvent Purification System. Reagents were purchased from commercial vendors (Sigma-Aldrich, Alfa Aesar, Acros, TCI, or Oakwood Chemical). Tris(pentafluorophenyl)corrole,[1] bis(diethylamino)chlorophosphine,[2] trityl tetrakis(pentafluorophenylborate),[3] and diethyl 2-(2-(pyrrolidin-1-yl)benzylidene)malonate[4] were synthesized according to reported procedures. Tri-*n*-octylphosphine oxide and benzophenone were recrystallized before use. Pyrrole and benzaldehyde were distilled before use. Other reagents were used as received. Column chromatography was carried out on silica gel (SiliFlash® Irregular Silica Gel, P60 40-63μm) or aluminum oxide (activated, neutral, Brockmann I) as noted. ^{1}H, ^{13}C, ^{19}F, and ^{31}P NMR spectra were recorded with either Bruker AVANCE-400, JEOL 500 MHz, or Bruker Avance-600 spectrometers and processed using MestReNova software. ^{1}H NMR chemical shifts are given in ppm with respect to solvent residual peak ($CDCl_3$, δ 7.26; CD_2Cl_2, δ 5.32; CD_3CN, δ 1.94). ^{13}C NMR shifts are given in ppm with respect to solvent residual peak ($CDCl_3$ δ 77.1, CD_2Cl_2 δ 53.8). ^{31}P shifts are given in ppm with respect to 85% H_3PO_4 (δ 0.0). Multiplicities are described as s = singlet, d = doublet, t = triplet, q = quartet, p = pentet, h = hextet, dd = doublet of doublets, td = triplet of doublets, m = multiplet. Coupling constants are reported in Hertz (Hz). UV-visible spectra were obtained on an Agilent Cary 60 spectrometer. High-resolution ESI mass spectra were obtained from the Mass Spectrometry Laboratory at the School of Chemical Sciences, University of Illinois at Urbana-Champaign. Nonlinear curve fitting for Michaelis-Menten and Hill analyses was performed using Mathematica 11.

2. Synthesis of starting materials, 1•H–4•H, and 1^{+}–4^{+}

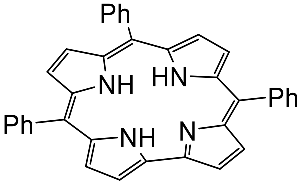

S1

5,10,15-Triphenylcorrole was synthesized using a modification of the reported procedure.[5] Benzaldehyde and pyrrole were distilled before use and were stored in the dark at –30 °C under nitrogen. 400 mL of water and 400 mL of MeOH were charged to a 1-L RB flask. The flask was wrapped in aluminum foil to exclude light, and pyrrole (2.8 mL, 40 mmol) and benzaldehyde (2.0 mL, 20 mmol) were added. Concentrated aqueous HCl (17 mL) was added in one portion with vigorous stirring. The mixture was stirred at room temperature under nitrogen for 1 h. The mixture was then extracted with chloroform (2 x 100 mL). The combined organic layers were washed twice with water and dried over anhydrous magnesium sulfate. The mixture was diluted to a total volume of 600 mL with chloroform, and *p*-chloranil (4.9 g, 20 mmol) was added in one portion with vigorous stirring. The dark solution was heated to reflux under air for 1 h. After cooling to room temperature, the reaction mixture was evaporated to dryness. The resulting residue was dissolved in CH_2Cl_2, and dry silica was added. After removal of solvent, the adsorbed mixture was quickly charged to a silica column (note: the crude mixture adsorbed on silica is prone to rapid degradation). The mixture was eluted with CH_2Cl_2, and the dark green band (R_f = 0.6) containing crude product was collected. All fractions containing the product were combined and evaporated to dryness. The product was recrystallized by dissolving in a minimum volume of hot CH_2Cl_2 (ca. 50 mL) followed by addition of hexanes (200 mL) and cooling at –30 °C overnight. The black solids were collected by filtration and were washed with hexanes until the filtrate became almost

colorless, leaving the desired product as shiny, dark purple solids (1.2 g, 32% yield). Spectral data are consistent with reported values.[5]

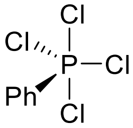

S2

P,*P*-dichlorophenylphosphine (3.0 mL, 16 mmol) was charged to a 3-neck round bottom flask equipped with a Teflon-coated stir bar and was dissolved in anhydrous CH_2Cl_2 (10 mL). Chlorine gas (generated by mixing concentrated aqueous HCl and trichloroisocyanuric acid) was passed through a $CaSO_4$-packed drying column and was bubbled through the phosphine solution until the solution became bright yellow (ca. 20 min). Excess chlorine was removed by sparging the solution with nitrogen until the yellow color disappeared. Removal of volatiles in vacuo afforded the product as a white solid (3.9 g, 99%), contaminated by a small amount of phenylphosphonic dichloride. The product is stable indefinitely when stored in a nitrogen-filled glovebox at –30°C. **^{1}H NMR** (400 MHz, $CDCl_3$) δ 8.07 (dd, *J* = 6.8, 3.0 Hz, 1H), 8.01 (dd, *J* = 6.5, 2.7 Hz, 1H), 7.62 – 7.50 (m, 3H). **^{13}C{^{1}H} NMR** (101 MHz, $CDCl_3$) δ 152.1 (d, *J* = 154.5 Hz), 132.0 (d, *J* = 5.6 Hz), 128.6 (d, *J* = 24.9 Hz), 124.0 (d, *J* = 17.4 Hz). **^{31}P NMR** (162 MHz, $CDCl_3$) δ -44.1 (tq, *J* = 27.1, 8.7 Hz).

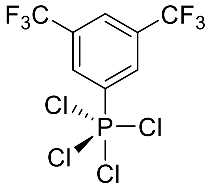

S3

To a –78 °C solution of 3,5-bis(trifluoromethyl)bromobenzene (3.6 mL, 21 mmol) in ether (11 mL) was added a solution of ^{n}BuLi (2.5 M in hexanes, 8.4 mL, 21 mmol) dropwise. The mixture was stirred at –78 °C for 1 h. The mixture was then cannula transferred to a solution of bis(diethylamino)chlorophosphine (4.9 mL, 23 mmol) in ether (47 mL) at –78 °C. The mixture was warmed to ambient temperature and stirred for 1 h. The reaction flask was then cooled to –78 °C, and a solution of HCl in ether (2.0 M, 47 mL, 94 mmol) was added slowly. The heterogeneous mixture was warmed to room temperature and stirred overnight (ca. 16 h). The mixture was then cannula filtered into a new flask. The remaining solids were washed with ether (2 x 20 mL), and the filtrates were also transferred to the new flask by cannula filtration. The volatiles were removed under high vacuum to afford an orange oil. The oil was vacuum distilled (35 °C, <1 torr) to afford a colorless liquid. The liquid was dissolved in anhydrous CH_2Cl_2 (10 mL) and chlorinated as in the procedure for **S2**. After removal of solvents, the product was obtained as a white solid in 58% overall yield (4.7 g, 12 mmol). The product was contaminated by ca. 3% of the phosphonic dichloride but was used without further purification. **^{1}H NMR** (400 MHz, $CDCl_3$) δ 8.41 (d, *J* = 25.3 Hz, 2H), 8.07 (d, *J* = 3.1 Hz, 1H). **^{13}C{^{1}H} NMR** (101 MHz, $CDCl_3$) δ 153.3 (d, *J* = 172.9 Hz), 132.5 (qd, *J* = 34.5, 25.3 Hz), 125.5 (dq, *J* = 7.5, 3.7 Hz), 124.2 (dq, *J* = 20.5, 3.6 Hz), 122.5 (dd, *J* = 273.7, 4.6 Hz). **^{19}F NMR** (376 MHz, $CDCl_3$) δ -62.9 (s). **^{31}P NMR** (162 MHz, $CDCl_3$) δ -50.2 (t, *J* = 25.4 Hz).

$[Bu_4N][BH(OAc)_3]$
S4

Tetrabutylammonium triacetoxyborohydride was prepared according to a procedure adapted from the literature.[6] A solution of tetrabutylammonium borohydride (3.0 g, 12 mmol) in toluene (25 mL) was cooled to 0 °C, and glacial acetic acid (2.3 mL, 41 mmol) was added dropwise. After the addition was complete, the mixture was stirred at room temperature for 3 h. Volatiles were removed by rotary evaporator, and further drying under high vacuum overnight afforded the product as an off-white semisolid (4.9 g, 97%). The product was contaminated with residual acetic acid but was used without further purification. **^{1}H NMR** (400 MHz, $CDCl_3$) δ 3.30 – 3.16 (m, 8H), 2.02 (s, 8H), 1.64 (p, *J* = 7.7 Hz, 8H), 1.41 (h, *J* = 7.4 Hz, 8H), 0.98 (t, *J* = 7.3 Hz, 12H). **^{13}C{^{1}H} NMR** (101 MHz, $CDCl_3$) δ 173.1, 58.7, 23.8, 23.4, 19.6, 13.6.

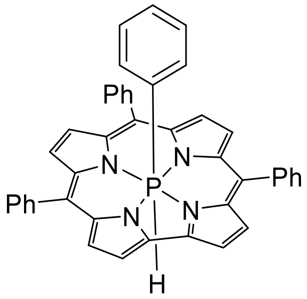

1•H

A solution of **S1** (1.1 g, 2.0 mmol) in toluene (25 mL) was charged to a 2-neck round bottom flask fitted with a stir bar and reflux condenser under nitrogen atmosphere. The solution was cooled to –78 °C and a solution of **S2** (500 mg, 2 mmol) in toluene (6 mL) was added via syringe with vigorous stirring. Triethylamine (900 µL, 6 mmol) was added, and the flask was warmed to room temperature. The reaction mixture was heated to reflux for 1 h before cooling back to room temperature. The mixture was washed with water and dried over magnesium sulfate before being concentrated to dryness by rotary evaporation. The mixture was separated by column chromatography on silica (1:5 EtOAc:hexanes as eluent), and the green band (R_f = 0.3) containing product was collected. This product contained a small amount of a partially chlorinated byproduct which was inseparable by chromatography or crystallization. To eliminate this impurity, the mixture of products was dissolved in ethyl acetate, and 10 mol % of 10 wt. % Pd/C (100 mg of wet material) was added. The mixture was stirred at room temperature under H_2 pressure (400 psi) overnight. The green residue was passed through a celite plug, and ethyl acetate was removed by rotary evaporator. The dark purple solids were dissolved in CH_2Cl_2 (5 mL), and TMS–OTf (100 µL, 0.6 mmol) was added, resulting in a dark red solution. The reaction was quenched with $[Bu_4N][BH_4]$ (300 mg, 1 mmol) to produce a brown-green solution. The desired product was purified on neutral alumina (5% EtOAc in hexanes) and was isolated as a blue solid (240 mg, 20% overall). **^{1}H NMR** (500 MHz, $CDCl_3$) δ 9.15 (d, *J* = 4.3 Hz, 2H), 9.02 (d, *J* = 4.9 Hz, 2H), 8.94 (d, *J* = 4.3 Hz, 2H), 8.70 (d, *J* = 4.7 Hz, 2H), 8.45 – 8.13 (m, 5H), 7.73 (tt, *J* = 33.0, 7.2 Hz, 10H), 5.51 (td, *J* = 7.2, 3.7 Hz, 1H), 5.06 (q, *J* = 7.3 Hz, 2H), 0.87 (dd, *J* = 24.7, 8.3 Hz, 2H), -2.73 (d, *J* = 928.8 Hz, 1H). **^{13}C{^{1}H} NMR** (126 MHz, $CDCl_3$) δ 149.4 (d, *J* = 265.8 Hz), 141.0, 139.9 (d, *J* = 49.8 Hz), 137.2, 134.5, 131.1, 130.0 (d, *J* = 4.2 Hz), 127.8, 127.7, 127.4, 126.6, 125.1, 124.5 (d, *J* = 24.4 Hz), 124.1, 123.3 (d, *J* = 19.4 Hz), 114.0, 113.5, 106.3. **^{31}P NMR** (203 MHz, $CDCl_3$) δ -

231.3 (dt, J = 928.0, 25.1 Hz). **HRMS (ESI)** calculated for $C_{43}H_{28}N_4P$ [M–H]$^+$: 631.2052, found: 631.2047.

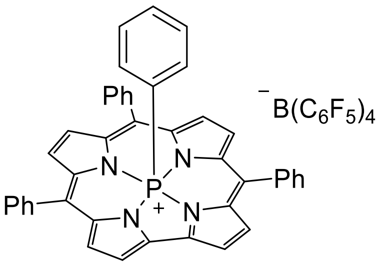

1⁺

Inside a nitrogen-filled glovebox, a solution of **1•H** (200 mg, 0.3 mmol) in CH_2Cl_2 (2 mL) was charged to a 20-mL glass vial containing a Teflon-coated stir bar. Trityl tetrakis(pentafluorophenyl)borate (280 mg, 0.30 mmol) was added with stirring. The color of the reaction mixture immediately changed from dark green to deep red. The mixture was stirred at room temperature for 5 min. With vigorous stirring, the desired product was precipitated from solution by dropwise addition of pentane (12 mL), and the supernatant containing triphenylmethane coproduct was decanted off. The precipitation was repeated for quantitative removal of triphenylmethane. The precipitated oily, maroon solid was then dried in vacuo to afford the product as a dark purple powder (290 mg, 74%). **^{1}H NMR** (400 MHz, CD_2Cl_2) δ 9.85 (dd, J = 4.6, 2.5 Hz, 2H), 9.53 (q, J = 4.2 Hz, 4H), 9.27 (dd, J = 5.2, 3.3 Hz, 2H), 8.23 – 8.06 (m, 6H), 7.94 – 7.83 (m, 9H), 6.17 (td, J = 7.6, 2.7 Hz, 1H), 5.64 (q, J = 7.4 Hz, 2H), 1.56 (dd, J = 20.3, 8.0 Hz, 2H). **^{19}F NMR** (376 MHz, CD_2Cl_2) δ -132.94 – -133.31 (m), -163.75 (t, J = 20.4 Hz), -167.56 (t, J = 19.6 Hz). **^{31}P NMR** (162 MHz, CD_2Cl_2) δ -97.1 (t, J = 20.6 Hz). HRMS (ESI) calculated for $C_{43}H_{28}N_4P$ [M]$^+$: 631.2052, found: 631.2050. ^{13}C NMR was not obtained due to poor solubility.

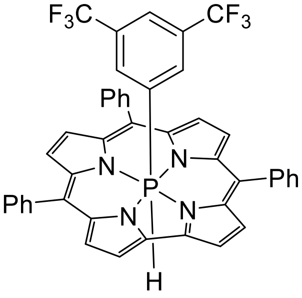

2•H

A solution of **S1** (530 mg, 1.0 mmol) in toluene (20 mL) was charged to a 2-neck round bottom flask fitted with a stir bar and reflux condenser under nitrogen atmosphere. The solution was cooled to –78°C and a solution of **S3** (390 mg, 1.0 mmol) in CH_2Cl_2 (3 mL) was added via syringe with vigorous stirring. Triethylamine (450 µL, 3.0 mmol) was added, and the flask was warmed to room temperature. The reaction mixture was heated to reflux for 1 h before cooling back to room temperature. A solution of **S4** (2.2 g, 5.0 mmol) in CH_2Cl_2 (8 mL) was added via syringe, and the

mixture was stirred at room temperature overnight. The flask was opened to air, and the dark residue was concentrated by rotary evaporator. The mixture was separated by column chromatography on neutral alumina (7% EtOAc in hexanes as eluent), and the brown-green band containing product was collected. After removal of solvents the product was obtained as a dark blue solid (180 mg, 24%). **^{1}H NMR** (600 MHz, $CDCl_3$) δ 9.22 (d, *J* = 4.2 Hz, 2H), 9.08 (d, *J* = 4.8 Hz, 2H), 8.99 (d, *J* = 4.2 Hz, 2H), 8.75 (d, *J* = 4.8 Hz, 2H), 8.26 (s, 5H), 7.90 – 7.62 (m, 10H), 6.00 (s, 1H), 1.08 (dd, *J* = 23.6, 1.6 Hz, 2H), -2.66 (d, *J* = 923.6 Hz, 1H). **^{13}C{^{1}H,^{19}F} NMR** (126 MHz, $CDCl_3$) δ 151.2, 149.1, 146.5, 146.3, 146.3 (d, *J* = 48.4 Hz), 142.4, 142.1, 138.6, 138.0 (d, *J* = 12.7 Hz), 137.8 (d, *J* = 4.4 Hz), 137.1, 130.8, 127.9 (d, *J* = 24.6 Hz), 126.0 (d, *J* = 40.4 Hz), 124.3, 122.8 (d, *J* = 22.0 Hz), 121.5, 119.0 (d, *J* = 4.4 Hz), 117.1, 114.0, 112.9, 96.7, 90.4. **^{19}F NMR** (565 MHz, $CDCl_3$) δ -64.0. **^{31}P NMR** (243 MHz, $CDCl_3$) δ -235.1 (dt, *J* = 923.2, 23.9 Hz). **HRMS (ESI)** calculated for $C_{45}H_{26}F_6N_4P$ $[M–H]^+$: 767.1799, found: 767.1802.

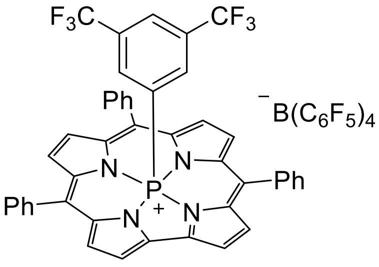

2^+

Inside a nitrogen-filled glovebox, a solution of **2•H** (130 mg, 0.20 mmol) in CH_2Cl_2 (2 mL) was charged to a 20-mL glass vial containing a Teflon-coated stir bar. Trityl tetrakis(pentafluorophenyl)borate (190 mg, 0.20 mmol) was added with stirring. The color of the reaction mixture immediately changed from dark green to deep red. The mixture was stirred at room temperature for 5 min. With vigorous stirring, the desired product was precipitated from solution by dropwise addition of pentane (12 mL), and the supernatant containing triphenylmethane coproduct was decanted off. The precipitation was repeated for quantitative removal of triphenylmethane. The precipitated oily, maroon solid was then dried in vacuo to afford the product as a dark purple powder (250 mg, 86%). **^{1}H NMR** (400 MHz, CD_2Cl_2) δ 9.93 (dd, *J* = 4.8, 2.7 Hz, 2H), 9.61 (q, *J* = 5.0 Hz, 4H), 9.34 (dd, *J* = 5.3, 3.4 Hz, 2H), 8.19 (br s, 4H), 8.10 – 8.01 (m, 2H), 7.97 – 7.81 (m, 9H), 6.68 (s, 1H), 1.82 (d, *J* = 20.0 Hz, 2H). **^{19}F NMR** (376 MHz, CD_2Cl_2) δ -64.9, -133.01 – -133.28 (m), -163.8 (t, *J* = 20.3 Hz), -167.6 (t, *J* = 19.9 Hz). **^{31}P NMR** (162 MHz, CD_2Cl_2) δ -102.2 (t, *J* = 18.7 Hz). HRMS (ESI) calculated for $C_{45}H_{26}F_6N_4P$ $[M]^+$: 767.1799, found: 767.1796. ^{13}C NMR was not obtained due to poor solubility.

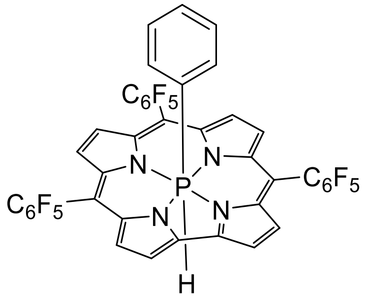

3•H

A solution of 5,10,15-tris(pentafluorophenyl)corrole (800 mg, 1 mmol) in toluene (25 mL) was charged to a 2-neck round bottom flask fitted with a stir bar and reflux condenser under nitrogen atmosphere. The solution was cooled to –78 °C and a solution of **S2** (250 mg, 1.0 mmol) in toluene (3 mL) was added via syringe with vigorous stirring. Triethylamine (450 µL, 3.0 mmol) was added, and the flask warmed to room temperature. The reaction mixture was heated to reflux for 1 h before cooling back to room temperature. A solution of **S4** (2.2 g, 5.0 mmol) in CH_2Cl_2 (8 mL) was added via syringe, and the mixture was stirred at room temperature overnight. The flask was opened to air, and the dark residue was concentrated by rotary evaporation. The mixture was purified by column chromatography on neutral alumina (1:2 CH_2Cl_2:hexanes as eluent), and the brown-yellow band containing product was collected. After removal of solvents the product was obtained as a dark blue solid (380 mg, 42%). **^{1}H NMR** (400 MHz, $CDCl_3$) δ 9.30 (d, *J* = 4.3 Hz, 2H), 8.96 (d, *J* = 4.8 Hz, 2H), 8.91 (d, *J* = 4.4 Hz, 2H), 8.71 (d, *J* = 4.8 Hz, 2H), 5.50 (td, *J* = 7.1, 3.7 Hz, 1H), 5.01 (dt, *J* = 8.5, 6.8 Hz, 2H), 0.68 (dd, *J* = 25.4, 8.1 Hz, 2H), -3.03 (d, *J* = 962.0 Hz, 1H). **^{13}C{^{1}H,^{19}F} NMR** (126 MHz, $CDCl_3$) δ 148.81, 146.61 (d, *J* = 8.3 Hz), 146.47, 146.19, 142.20, 141.99, 138.79, 138.02, 137.78 (d, *J* = 10.0 Hz), 137.28, 130.94 (d, *J* = 18.6 Hz), 125.85, 125.29, 125.18 (d, *J* = 5.6 Hz), 124.79 (d, *J* = 24.6 Hz), 124.01, 122.70 (d, *J* = 20.0 Hz), 116.65, 114.74, 113.64, 96.35, 90.08. **^{19}F NMR** (376 MHz, $CDCl_3$) δ -136.6 – -136.8 (m), -136.8 (dd, *J* = 24.9, 8.6 Hz), -137.0 (ddd, *J* = 24.0, 8.7, 3.3 Hz), -151.9 (t, *J* = 20.8 Hz), -152.6 (t, *J* = 20.9 Hz), -161.2 (ddd, *J* = 24.2, 20.9, 8.6 Hz), -161.3 (ddd, *J* = 24.5, 20.9, 8.5 Hz), -161.8 (dddd, *J* = 50.3, 24.7, 20.8, 8.5 Hz). **^{31}P NMR** (162 MHz, $CDCl_3$) δ -230.8 (dt, *J* = 962.1, 25.6 Hz). **HRMS (ESI)** calculated for $C_{43}H_{13}N_4F_{15}P$ $[M–H]^+$: 901.0638, found: 901.0629.

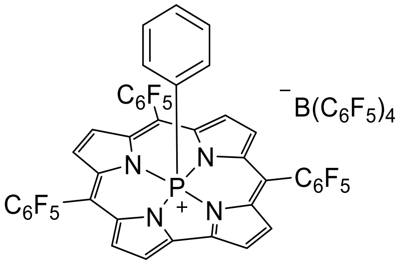

3^+

Inside a nitrogen-filled glovebox, a solution of **3•H** (370 mg, 0.42 mmol) in CH_2Cl_2 (2 mL) was charged to a 20-mL glass vial containing a Teflon-coated stir bar. Trityl tetrakis(pentafluorophenyl)borate (360 mg, 0.40 mmol) was added with stirring. The color of the reaction mixture immediately changed from dark green to deep red. The mixture was stirred at room temperature for 5 min. With vigorous stirring, the desired product was precipitated from solution by dropwise addition of pentane (12 mL), and the supernatant containing triphenylmethane coproduct was decanted off. The precipitation was repeated for quantitative removal of triphenylmethane. The precipitated oily, maroon solid was then dried in vacuo to afford the product as a red powder (570 mg, 90%). **^{1}H NMR** (400 MHz, CD_2Cl_2) δ 10.05 (dd, *J* = 4.9, 2.6 Hz, 2H), 9.57 (t, *J* = 4.5 Hz, 2H), 9.53 (t, *J* = 4.8 Hz, 2H), 9.35 (dd, *J* = 5.3, 3.3 Hz, 2H), 6.18 (td, *J* = 7.5, 2.9 Hz, 1H), 5.62 (q, *J* = 7.6 Hz, 2H), 1.43 (dd, *J* = 20.8, 8.0 Hz, 2H). **^{19}F NMR** (376 MHz, CD_2Cl_2) δ -133.04 – -133.41 (m), -137.46 (tdd, *J* = 26.1, 13.9, 6.5 Hz), -148.40 (tt, *J* = 20.8, 2.7 Hz), -148.59 (tt, *J* = 20.8, 2.7 Hz), -159.32 – -159.52 (m), -159.51 – -159.83 (m), -163.84 (t, *J* = 20.3 Hz), -167.68 (t, *J* = 19.7 Hz). **^{31}P NMR** (162 MHz, CD_2Cl_2) δ -95.2. **HRMS (ESI)** calculated for $C_{43}H_{13}F_{15}N_4P$ $[M]^+$: 901.0638, found: 901.0626. ^{13}C NMR was not obtained due to poor solubility.

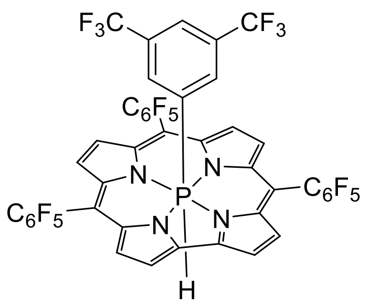

4•H

A solution of 5,10,15-tris(pentafluorophenyl)corrole (800 mg, 1 mmol) in toluene (20 mL) was charged to a 2-neck round bottom flask fitted with a stir bar and reflux condenser under nitrogen atmosphere. The solution was cooled to –78 °C and a solution of **S3** (390 mg, 1.0 mmol) in CH_2Cl_2 (3 mL) was added via syringe with vigorous stirring. Triethylamine (280 µL, 3.0 mmol) was added, and the flask warmed to room temperature. The reaction mixture was heated to reflux for 1 h before cooling back to room temperature. A solution of tetrabutylammonium triacetoxyborohydride (1.9 g, 4.3 mmol) in CH_2Cl_2 (8 mL) was added via syringe, and the mixture was stirred at room temperature overnight. The flask was opened to air, and the dark residue was concentrated by rotary evaporator. The mixture was separated by column chromatography on neutral alumina (1:2 CH_2Cl_2:hexanes as eluent), and the purple-yellow band containing product was collected. After removal of solvents the product was obtained as a dark blue solid (350 mg, 34%). **^{1}H NMR** (400 MHz, $CDCl_3$) δ 9.39 (d, *J* = 4.4 Hz, 2H), 9.06 (d, *J* = 4.9 Hz, 2H), 9.01 (d, *J* = 4.4 Hz, 2H), 8.80 (d, *J* = 4.9 Hz, 2H), 6.03 (s, 1H), 0.97 (d, *J* = 24.5 Hz, 2H), -2.97 (d, *J* = 951.8 Hz, 1H). **^{13}C{^{1}H,^{19}F} NMR** (126 MHz,$CDCl_3$) δ 151.33, 149.17, 146.63, 146.59, 146.44, 146.24, 142.45, 142.22, 138.69, 138.09 (d, *J* = 12.6 Hz), 137.87 (d, *J* = 4.4 Hz), 137.25, 130.91, 128.00 (d, *J* = 24.7 Hz), 126.11 (d, *J* = 40.4 Hz), 124.36, 122.90 (d, *J* = 22.0 Hz), 121.63, 119.09 (d, *J* = 4.4 Hz), 117.16, 114.12, 113.04, 96.75, 90.46. **^{19}F NMR** (376 MHz, $CDCl_3$) δ -64.41 (t, *J* = 6.0 Hz), -136.79 – -136.92 (m), -136.98 (dd, *J* = 24.4, 8.7 Hz), -137.21 (ddd, *J* = 24.2, 8.8, 3.6 Hz), -151.33 (t, *J* = 21.0 Hz), -151.97 (t, *J* = 21.0 Hz), -160.69 (ddd, *J* = 23.9, 20.8, 8.4 Hz), -161.04 (ddd, *J* = 23.9, 20.8, 8.6 Hz), -161.17 (ddd, *J* = 24.5, 21.2, 8.8 Hz), -161.40 (ddd, *J* = 24.3, 20.8, 8.6 Hz). **^{31}P NMR** (162 MHz, $CDCl_3$) δ -234.3 (dt, *J* = 952.3, 24.4 Hz). **HRMS (ESI)** calculated for $C_{45}H_{11}F_{21}N_4P$ [M–H]$^+$: 1037.0386, found: 1037.0415.

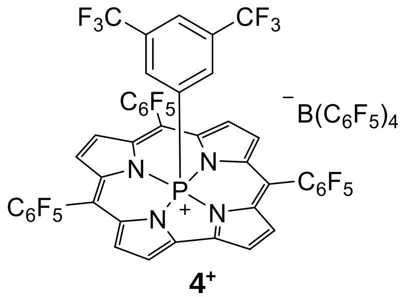

4$^+$

A solution of **4•H** (320 mg, 0.31 mmol) in CH_2Cl_2 (2 mL) was charged to a 20-mL glass vial containing a Teflon-coated stir bar. Trityl tetrakis(pentafluorophenyl)borate (270 mg, 0.30 mmol) was added with stirring. The color of the reaction mixture immediately changed from dark green to deep red. The mixture was stirred at room temperature for 5 min. With vigorous stirring, the desired product was precipitated from solution by dropwise addition of pentane (12 mL), and the supernatant containing triphenylmethane coproduct was decanted off. The precipitation was

repeated for quantitative removal of triphenylmethane. The precipitated oily, maroon solid was then dried in vacuo to afford the product as a red powder (370 mg, 68%). **^{1}H NMR** (400 MHz, CD_2Cl_2) δ 10.15 (dd, *J* = 5.0, 2.7 Hz, 2H), 9.68 (t, *J* = 4.6 Hz, 2H), 9.64 (t, *J* = 4.8 Hz, 2H), 9.46 (t, *J* = 4.4 Hz, 2H), 6.72 (s, 1H), 1.76 (d, *J* = 20.4 Hz, 2H). **^{19}F NMR** (376 MHz, CD_2Cl_2) δ -65.2, -133.0 – -133.4 (m), -137.3 (ddt, *J* = 22.8, 14.7, 7.1 Hz), -137.9 (ddq, *J* = 18.4, 9.5, 5.0 Hz), -138.1 (d, *J* = 22.9 Hz), -147.7 (t, *J* = 20.7 Hz), -147.9 (t, *J* = 20.7 Hz), -159.1 (ddtd, *J* = 44.5, 30.4, 21.8, 8.5 Hz), -163.9 (t, *J* = 20.3 Hz), -167.7 (t, *J* = 19.8 Hz). **^{31}P NMR** (162 MHz, CD_2Cl_2) δ -100.3. **HRMS (ESI)** calculated for $C_{45}H_{11}N_4F_{21}P$ [M^+]: 1037.0386, found: 1037.0377. ^{13}C NMR was not obtained due to poor solubility.

3. Gutmann-Beckett tests for 1^+–4^+

Inside a nitrogen-filled glovebox, 0.20 mmol of the phosphacorrole was charged to a glass vial, and a solution of tri-*n*-octylphosphine oxide (7.7 mg, 0.20 mmol) in CH_2Cl_2 (0.6 mL) was added. The mixture quickly changed color and became homogeneous. The solution was transferred to an NMR tube with threaded end and was stoppered with a cap with a PTFE septum. The mixture was then analyzed by ^{31}P NMR. The Δδ value was obtained by subtracting the chemical shift of the bound (*n*-octyl)$_3$P=O signal from that of free (*n*-octyl)$_3$P=O in CH_2Cl_2 (δ 46.7).

We observed that (*n*-octyl)$_3$P=O reacted slowly with the $B(C_6F_5)_4$ anion to generate a minor impurity with a new ^{31}P resonance at δ 72.2 ppm. We have assigned the structure of the byproduct as the cationic $[(n\text{-Oct})_3POC_6F_4H]^+$ (detected by HRMS (ESI), calculated for $C_{30}H_{52}F_4OP$ [M^+]: 535.3692, found 535.3725), which is presumably formed from an S_NAr-like reaction on a C_6F_5 group of the $B(C_6F_5)_4$ anion. This assignment is further supported by the concomitant appearance of the fluoride adducts of **1^+–4^+** (^{31}P NMR δ ca. –200 ppm, $^1J_{P\text{-}F}$ ca. 770 Hz), indicating formation of a P–F bond. The ^{19}F NMR spectrum showed a resonance with the same coupling constant at δ ca. –15 ppm. Furthermore, the sharp singlet resonance and the chemical shift of the byproduct (δ 72.2 ppm) are consistent with a phosphonium species. Although the identity of this impurity could not be confirmed definitively, its low abundance in the reaction mixture is expected to have a negligible effect on the Gutmann-Beckett measurement.

4. UV-vis spectrophotometric titrations

A stock solution (3.33 x 10^{-5} M) of each compound **1^+–4^+** in CH_2Cl_2 was prepared in a nitrogen-filled glovebox. 3.00 mL of this solution was transferred to a 1.00-cm long quartz cuvette with threaded end for a plastic cap lined with a silicone septum. The cuvette was removed from the glovebox for data acquisition and brought back inside for addition of each equivalent of (*n*-octyl)$_3$P=O. The solution was equilibrated (determined by constant absorbance between several consecutive scans) before acquiring each spectrum. Scans were acquired over the range of 700–300 nm. The Q band region of the spectrum (700–500 nm) proved most useful for spectroscopic analysis. λ_{max} was taken as 629 nm for **1^+**, 624 nm for **2^+**, 607 nm for **3^+**, and 603 nm for **4^+**. The molar absorptivity of the (*n*-octyl)$_3$P=O adduct was approximated by asymptotic convergence of absorbance at λ_{max} to a single value ($\log_{10} \varepsilon$ = 4.62 for **1^+**, 4.65 for **2^+**, 4.47 for **3^+**, and 4.48 for **4^+**). These molar absorptivities were used to compute fractional occupancy for fitting to the Hill equation. All titrations were performed with two replicates.

5. Water stability test for 3^+

A solution of $\mathbf{3^+}$ (16 mg, 0.010 mmol) in CD_3CN (0.6 mL) was charged to an NMR tube with threaded end and capped with a plastic cap with PTFE rubber septum. Water (2 μL, 0.1 mmol) was injected via syringe. The red solution immediately changed color to purple-green. The sample was then analyzed by NMR. A very small amount (< 1% as determined by ^{19}F NMR) of the counterion was observed to react with water as evidenced by formation of a P–F bond ($^1J_{P\text{-}F}$ = 725.6 Hz), but otherwise the only ^{31}P resonance corresponded to $\mathbf{3^+{\bullet}OH_2}$. **^{1}H NMR** (400 MHz, CD_3CN) δ 9.59 (d, *J* = 4.6 Hz, 2H), 9.28 (d, *J* = 5.0 Hz, 2H), 9.22 (t, *J* = 3.3 Hz, 2H), 9.04 (d, *J* = 5.0 Hz, 2H), 5.65 – 5.54 (m, 1H), 5.09 (q, *J* = 7.5 Hz, 2H), 0.70 (dd, *J* = 21.3, 8.1 Hz, 2H). **^{31}P NMR** (162 MHz, CD_3CN) δ -175.3 (br).

6. Experimental procedures for catalytic transformations

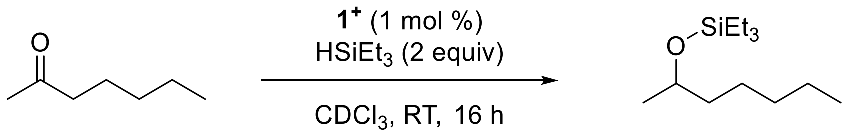

Catalytic hydrosilylation of 2-heptanone. Inside of a nitrogen-filled glovebox, $\mathbf{1^+}$ (7 mg, 0.005 mmol) was charged to a glass vial and dissolved in $CDCl_3$ (0.7 mL), followed by addition of 2-heptanone (57 mg, 0.50 mmol) and triethylsilane (160 uL, 1.0 mmol). CH_2Br_2 (26 uL, 0.38 mmol) was added as an internal standard. The mixture was transferred to an NMR tube with threaded end and stoppered with a cap with a PTFE septum. The mixture was aged at room temperature overnight (16 h). The reaction mixture was analyzed by NMR (^{1}H chemical shifts are referenced to CH_2Br_2, taken as δ 5.00), and the yield was determined by integration of the ^{1}H diagnostic peak (δ 3.86, h, *J* = 6.1 Hz) vs the internal standard (90% yield). The 2-heptanone was fully consumed, as evidenced by a lack of characteristic resonances (^{1}H: δ 2.12, s; $^{13}C\{^1H\}$: δ 208.9). **$^{13}C\{^1H\}$ NMR** (126 MHz, $CDCl_3$) δ 68.5, 39.8, 32.0, 25.5, 23.8, 22.7, 14.0, 8.1, 6.9. Further characterization by ^{1}H NMR was precluded by overlapping signals from $HSiEt_3$. **HRMS (EI)** calculated for $C_{13}H_{29}OSi$ $[M–H]^+$: 229.1988, found: 229.1992.

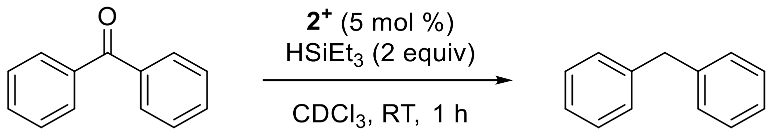

Catalytic deoxygenation of benzophenone. Inside of a nitrogen-filled glovebox, $\mathbf{1^+}$ (15 mg, 0.010 mmol) was charged to a glass vial and dissolved in $CDCl_3$ (0.7 mL), followed by addition of benzophenone (46 mg, 0.25 mmol) and triethylsilane (81 uL, 0.50 mmol). CH_2Br_2 (18 uL, 0.25 mmol) was added as an internal standard. The mixture was transferred to an NMR tube with threaded end and was stoppered with a cap with a PTFE septum. The mixture was aged at room temperature for 1 h. The reaction mixture was analyzed by NMR (^{1}H chemical shifts are referenced to CH_2Br_2, taken as δ 5.00), and the yield was determined by integration of the diagnostic ^{1}H peak (δ 4.11, s) vs the internal standard (97% yield). The benzophenone was fully

consumed, as evidenced by a lack of the characteristic resonance $^{13}C\{^1H\}$: δ 196.4. **^{1}H NMR** (400 MHz, $CDCl_3$) δ 7.41 (t, *J* = 7.5 Hz, 5H), 7.36 – 7.22 (m, 5H), 4.11 (s, 2H). **$^{13}C\{^1H\}$ NMR** (101 MHz, $CDCl_3$) δ 141.1, 129.0, 128.5, 126.1, 42.0.

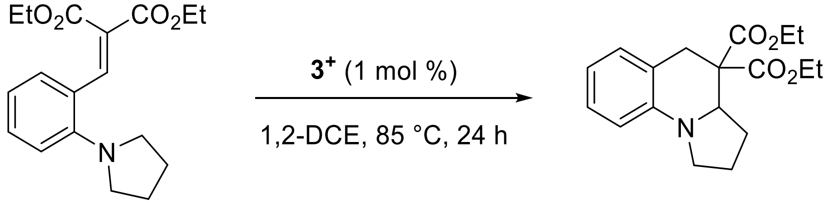

Catalytic 1,5-hydride shift of diethyl 2-(2-(pyrrolidin-1-yl)benzylidene)malonate. Inside of a nitrogen-filled glovebox, **3**$^+$ (16 mg, 0.010 mmol) was charged to a 10-mL glass culture tube with threaded end outfitted with a phenolic screw-cap open top cap and PTFE-lined silicone septum equipped with a Teflon-coated stir bar. The catalyst was dissolved in anhydrous 1,2-dichloroethane (3 mL), and the alkylidene starting material (317 mg, 1.00 mmol) was added. The tube was heated at 85 °C for 24 h. After cooling to room temperature, all volatiles were removed by rotary evaporation. The mixture was then purified on silica (gradient of 5% EtOAc to 10% EtOAc in hexanes) to afford the product as an oil (240 mg, 76%). **^{1}H NMR** (400 MHz, $CDCl_3$) δ 7.08 (t, *J* = 7.8 Hz, 1H), 7.02 (d, *J* = 7.4 Hz, 1H), 6.59 (t, *J* = 7.4 Hz, 1H), 6.45 (d, *J* = 8.1 Hz, 1H), 4.36 – 4.16 (m, 2H), 4.13 – 3.95 (m, 2H), 3.78 (dd, *J* = 9.2, 6.8 Hz, 1H), 3.44 – 3.15 (m, 4H), 2.57 – 2.36 (m, 1H), 2.22 – 2.03 (m, 2H), 2.02 – 1.86 (m, 1H), 1.30 (t, *J* = 7.2 Hz, 3H), 1.06 (t, *J* = 7.1 Hz, 3H). **$^{13}C\{^1H\}$ NMR** (101 MHz, $CDCl_3$) δ 171.1, 168.6, 143.8, 128.5, 127.5, 118.7, 115.8, 110.8, 62.2, 61.5, 60.8, 53.0, 47.4, 37.0, 27.8, 23.6, 14.1, 13.9. These values are consistent with the literature report.[4]

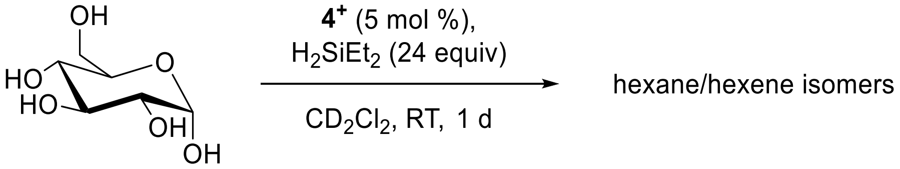

*Catalytic deoxygenation of $^{13}C_6$-*D*-glucose.* Inside of a nitrogen-filled glovebox, **4**$^+$ (8.7 mg, 0.0050 mmol) was charged to a 1-dram glass vial with a Teflon-coated stir bar. The catalyst was suspended in CD_2Cl_2 (200 μL) and diethylsilane (310 μL, 2.4 mmol). To this mixture was added $^{13}C_6$-*D*-glucose (99 atom %, 18.0 mg, 0.100 mmol). The heterogenous mixture was sealed with a screw-cap open top cap and PTFE-lined silicone septum and was stirred for 24 h. At the end of the reaction, the system had become pressurized (presumably due to formation of hydrogen gas), and the mixture was homogeneous and green in color. The mixture was transferred to an NMR tube with a threaded end, and $Cr(acac)_3$ (25 mg, 0.072 mmol) was added to the reaction mixture as a paramagnetic relaxation reagent. Natural-abundance cyclooctane (108 uL, 0.800 mmol) was added as an internal standard. T1 values for each ^{13}C nucleus in the solution were obtained using standard inversion-recovery pulse sequences, and the longest T1 measured was 1.7 s. A delay time of 10.0 s was used in the quantitative measurement to ensure that all spins were fully relaxed. Peaks were assigned by comparing to the literature report.[7]

7. Multinuclear NMR spectra

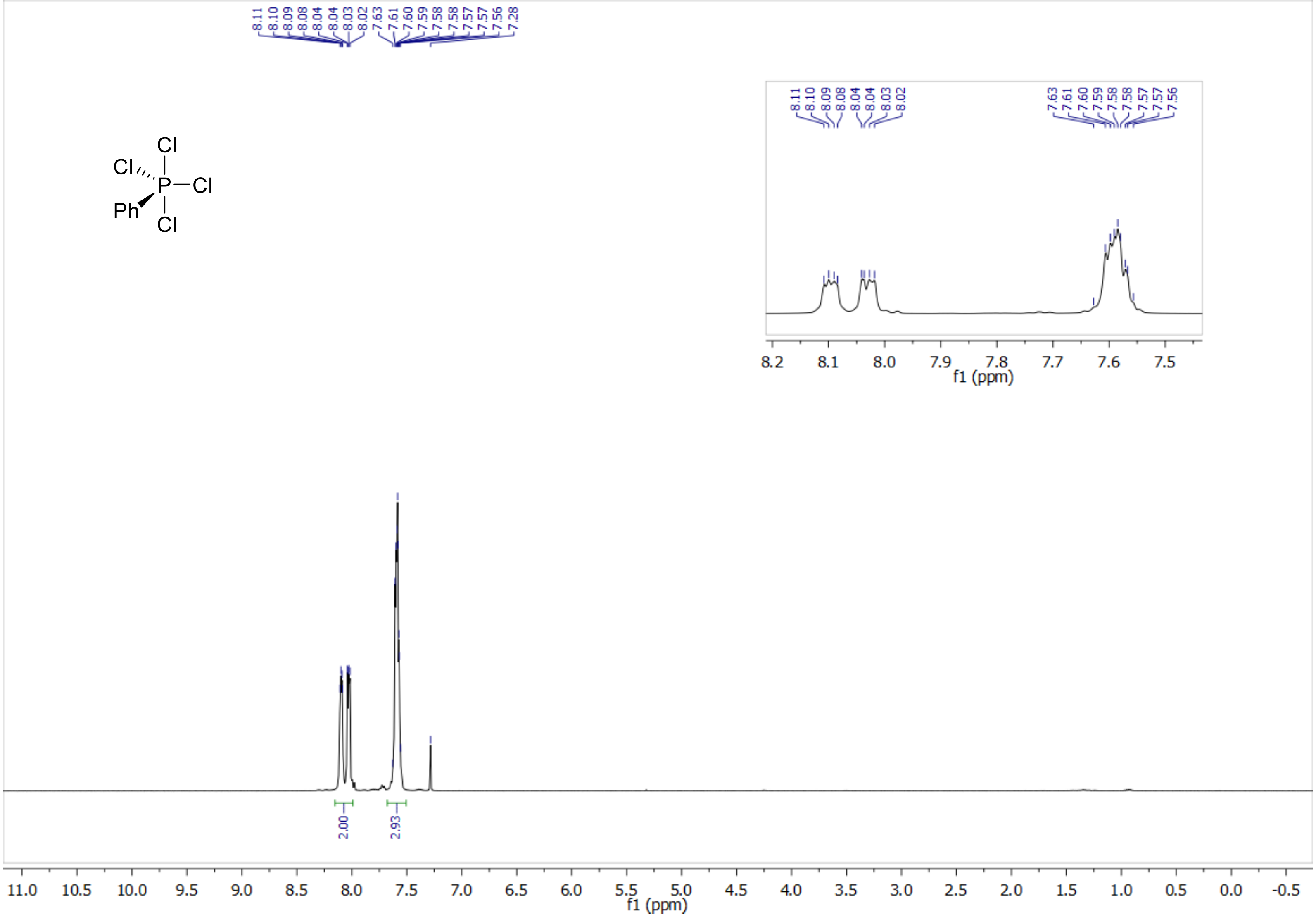

Figure S1. *^{1}H NMR spectrum of* ***S2****.*

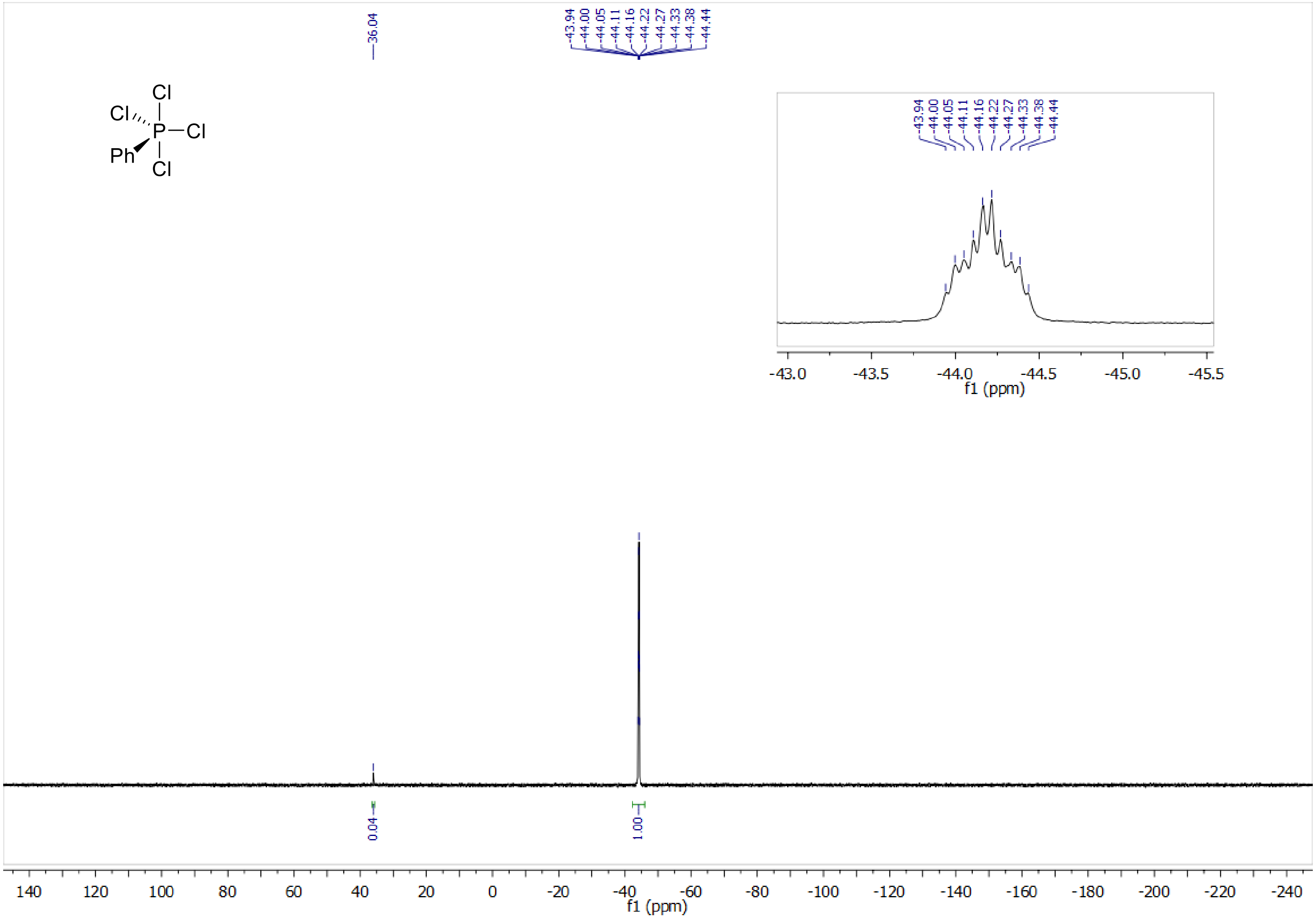

Figure S2. *^{31}P NMR spectrum of* ***S2****.*

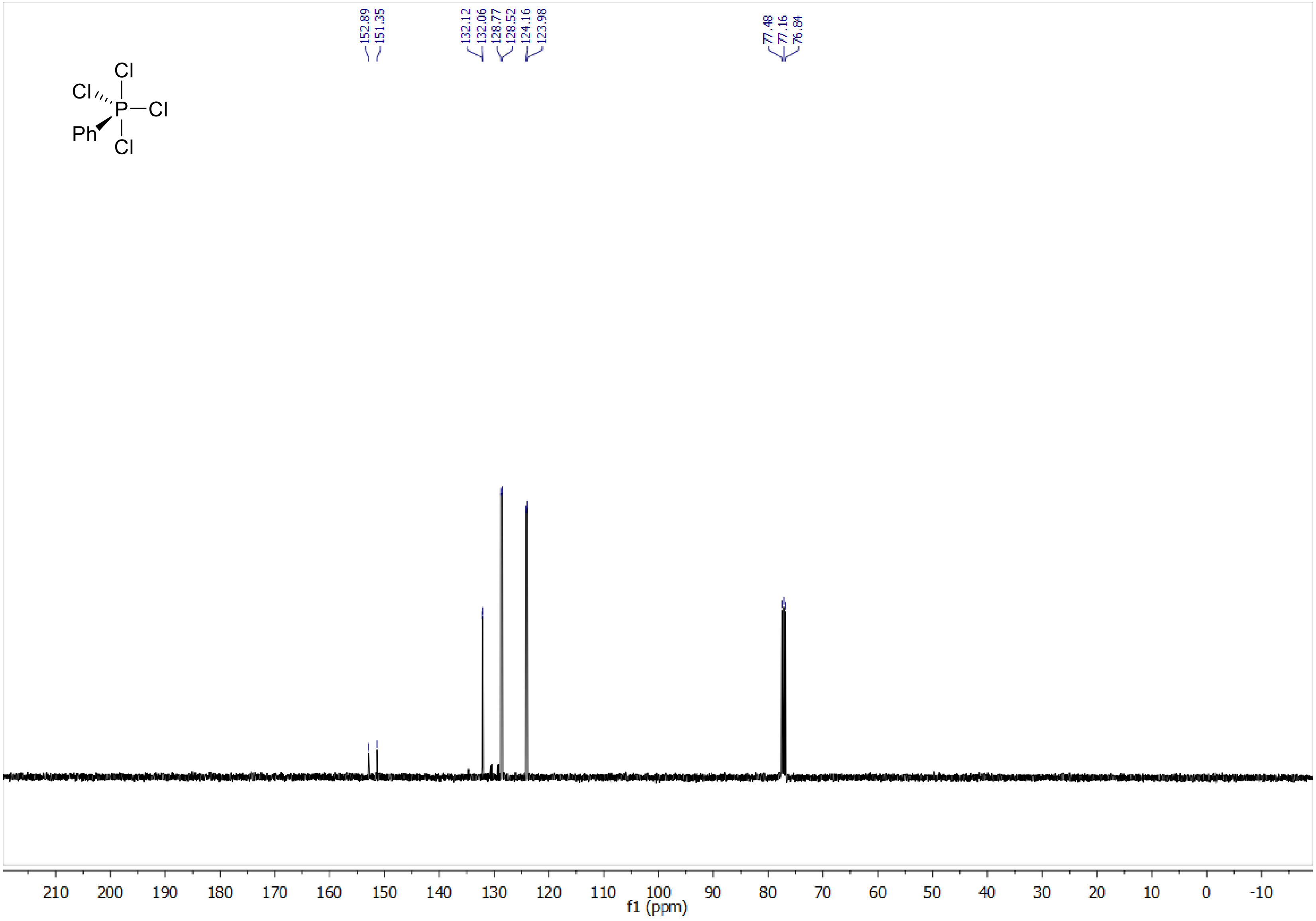

Figure S3. *^{13}C NMR spectrum of **S2**.*

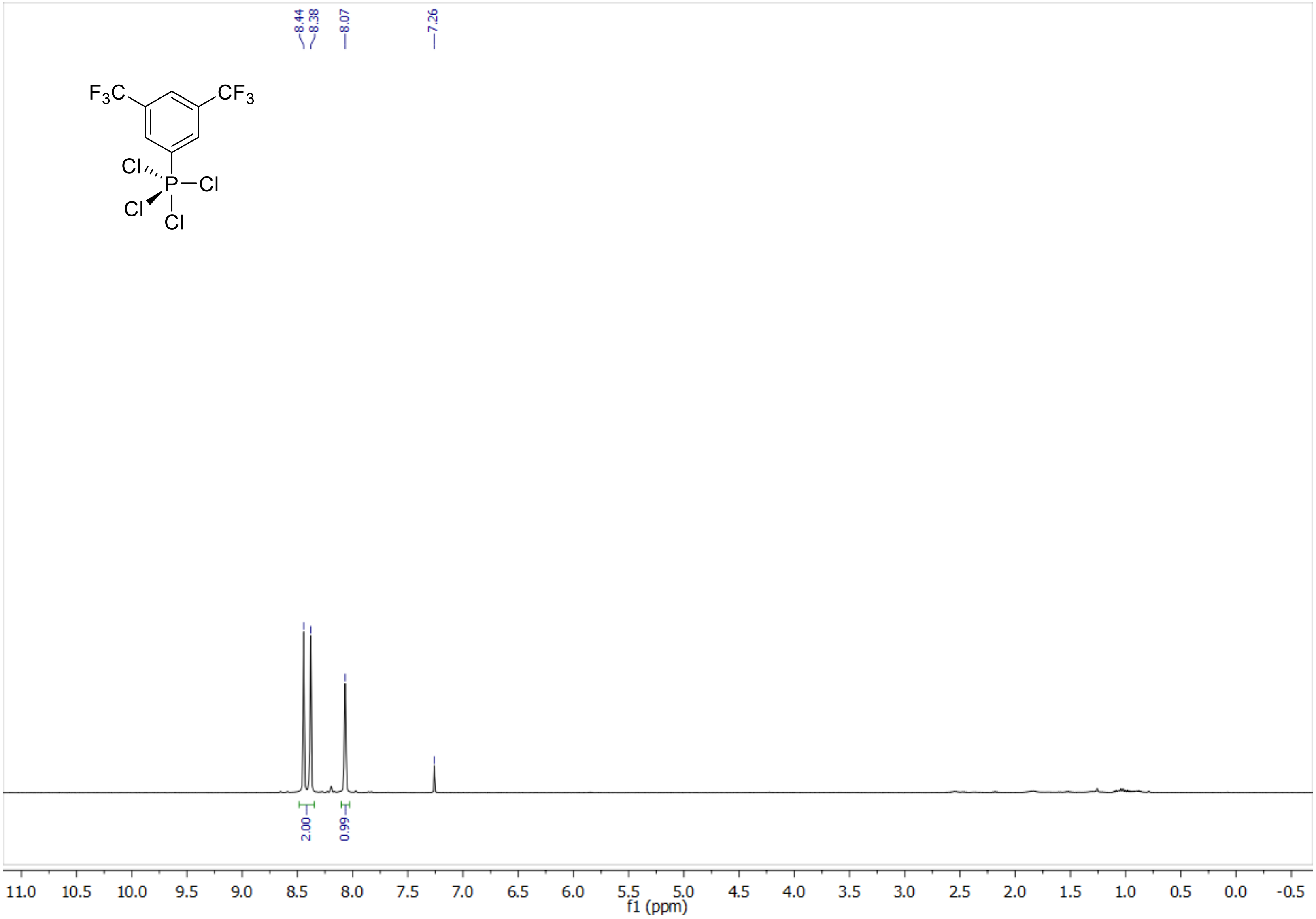

Figure S4. *^{1}H NMR spectrum of* ***S3****.*

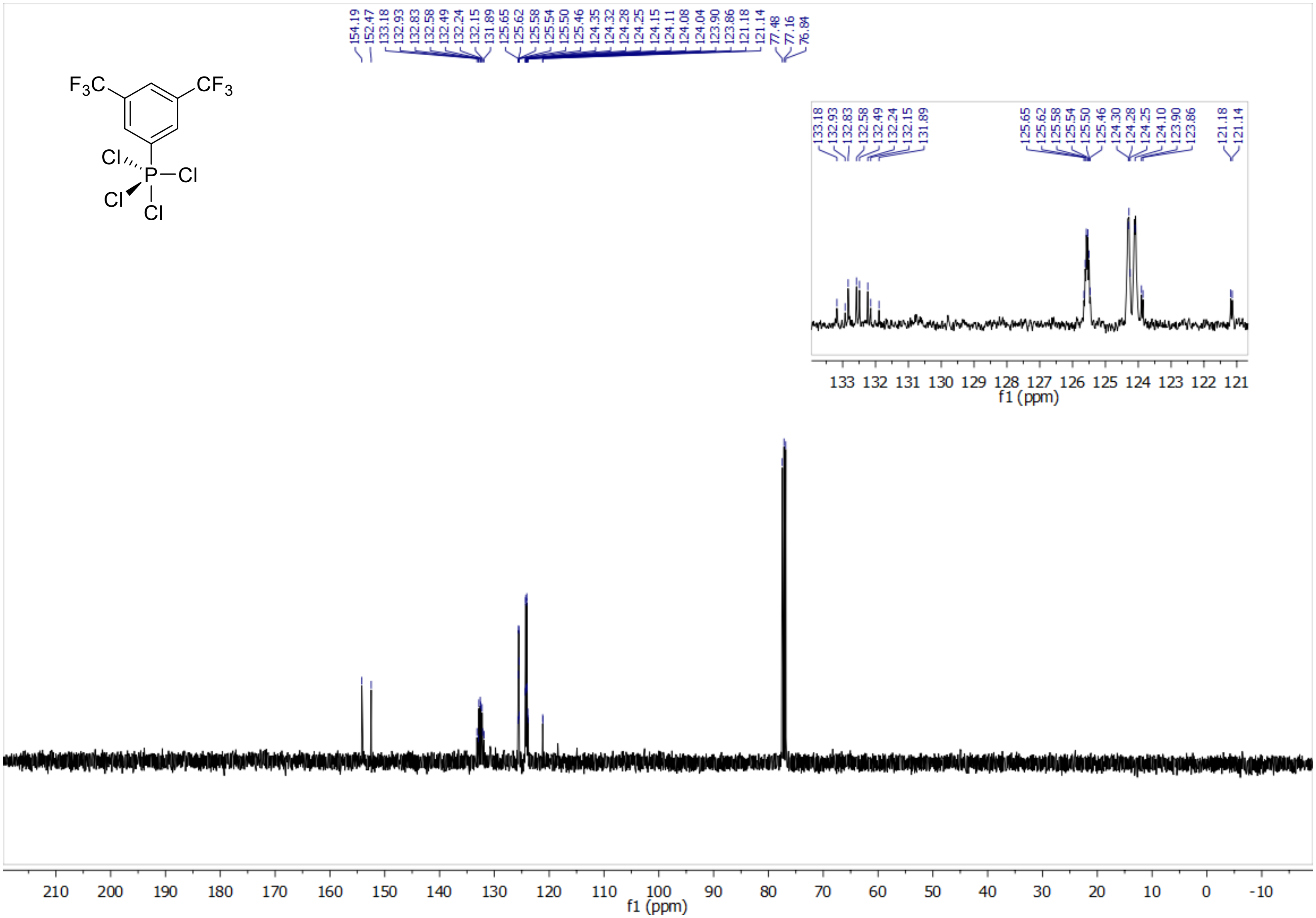

Figure S5. *^{13}C{^{1}H} NMR spectrum of* ***S3****.*

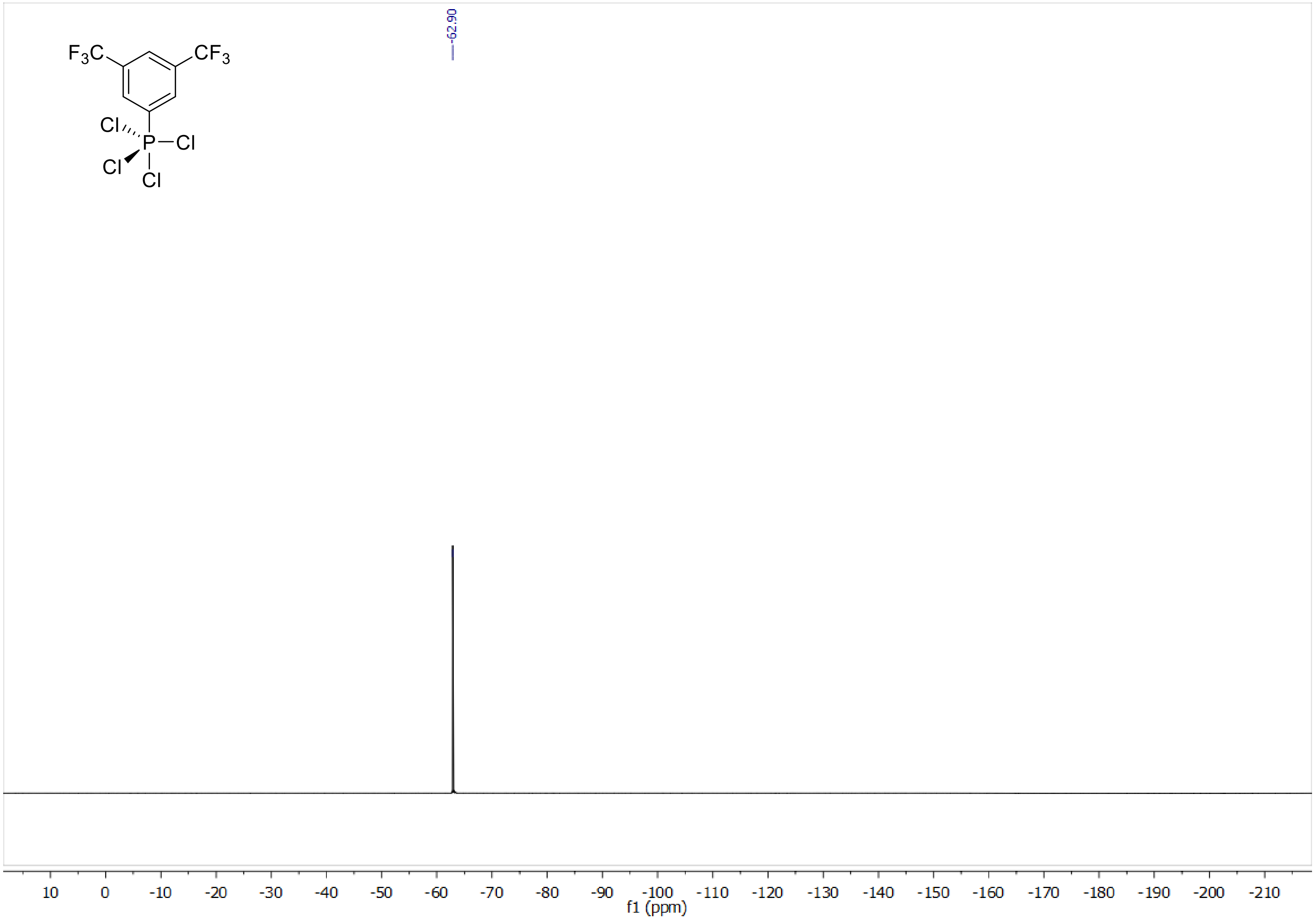

Figure S6. *^{19}F NMR spectrum of* ***S3****.*

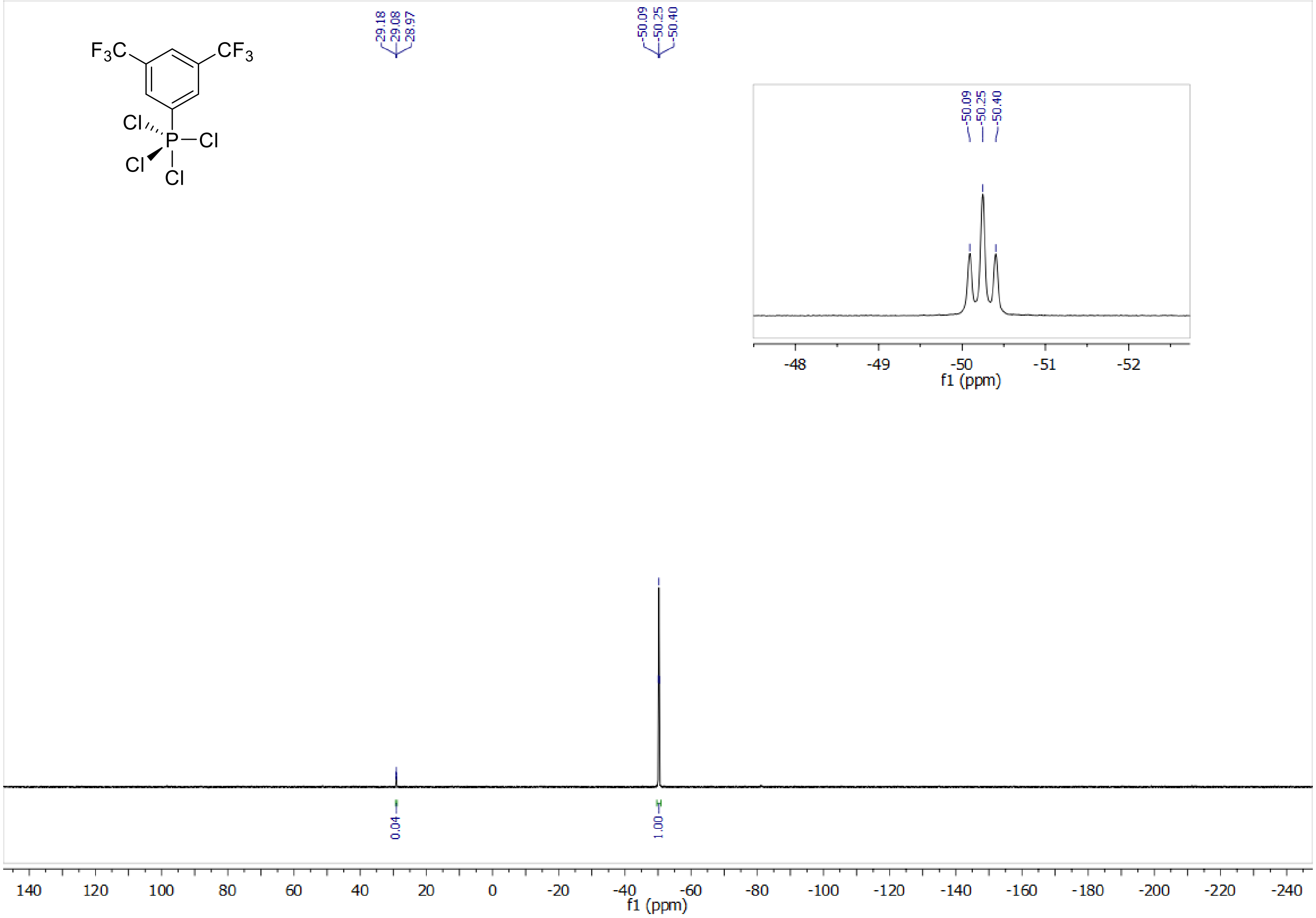

Figure S7. *^{31}P NMR spectrum of* ***S3****.*

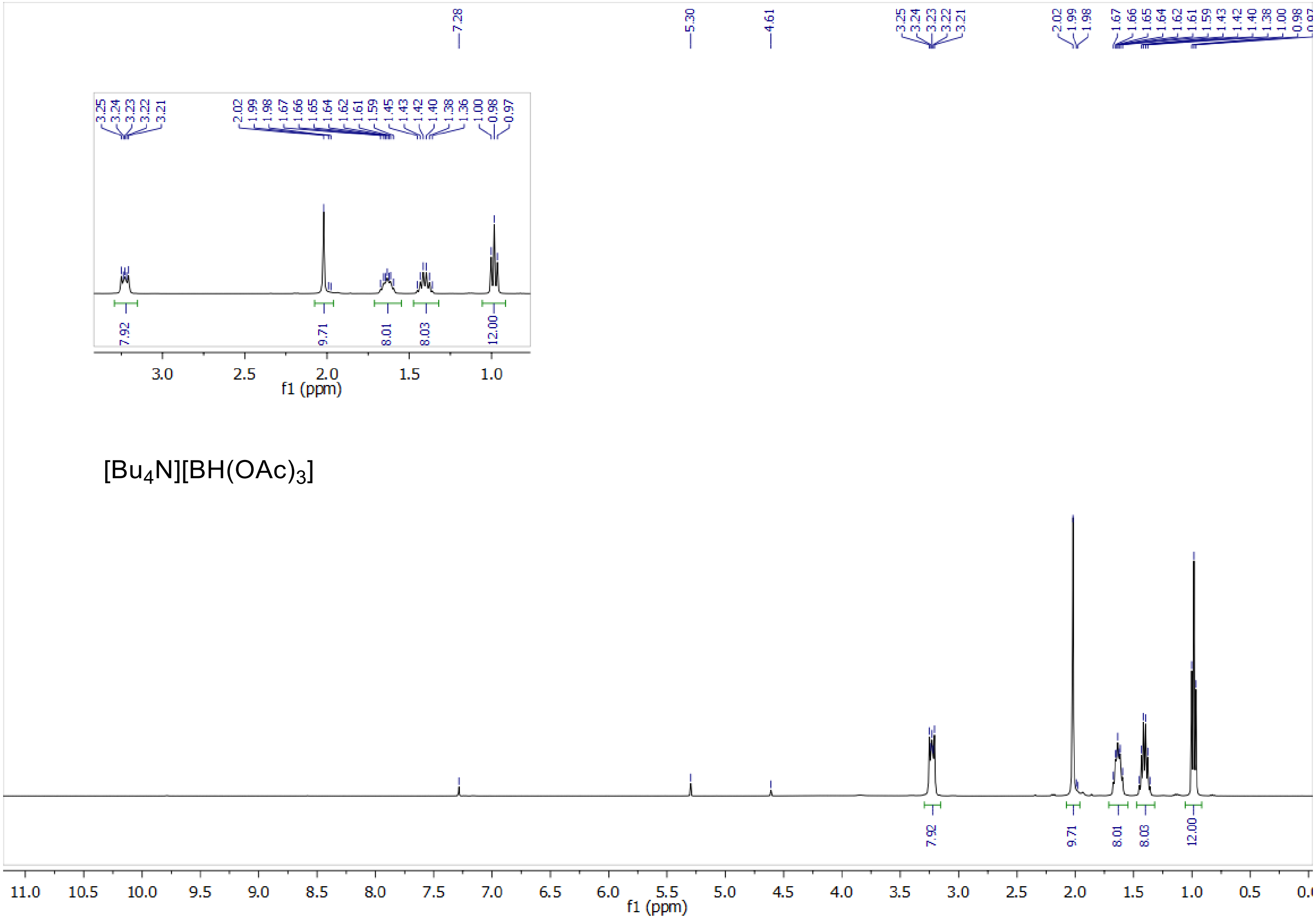

Figure S8. *^{1}H NMR spectrum of* ***S4****.*

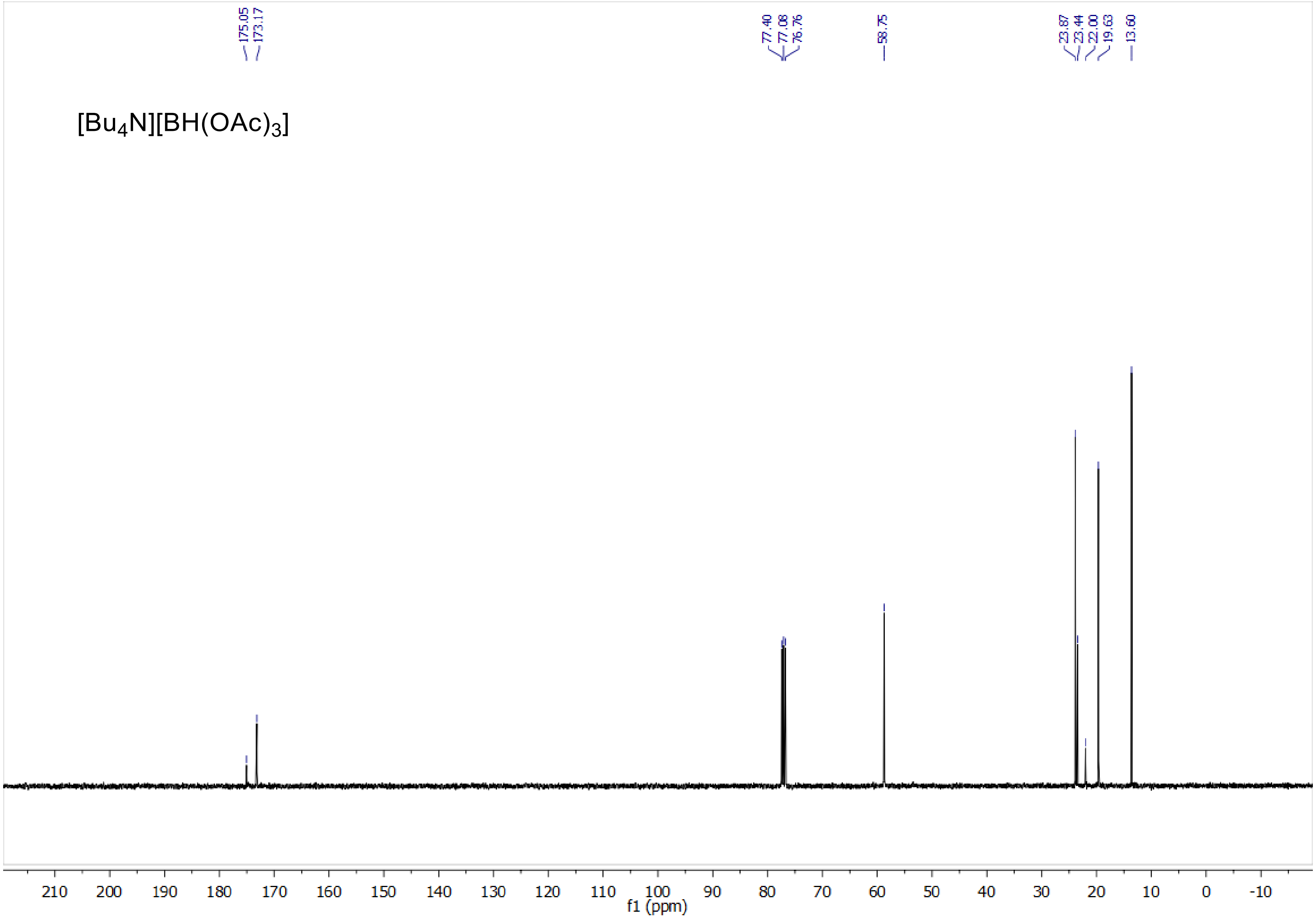

Figure S9. *$^{13}C\{^1H\}$ NMR spectrum of **S4**. Residual HOAc is a minor contaminant.*

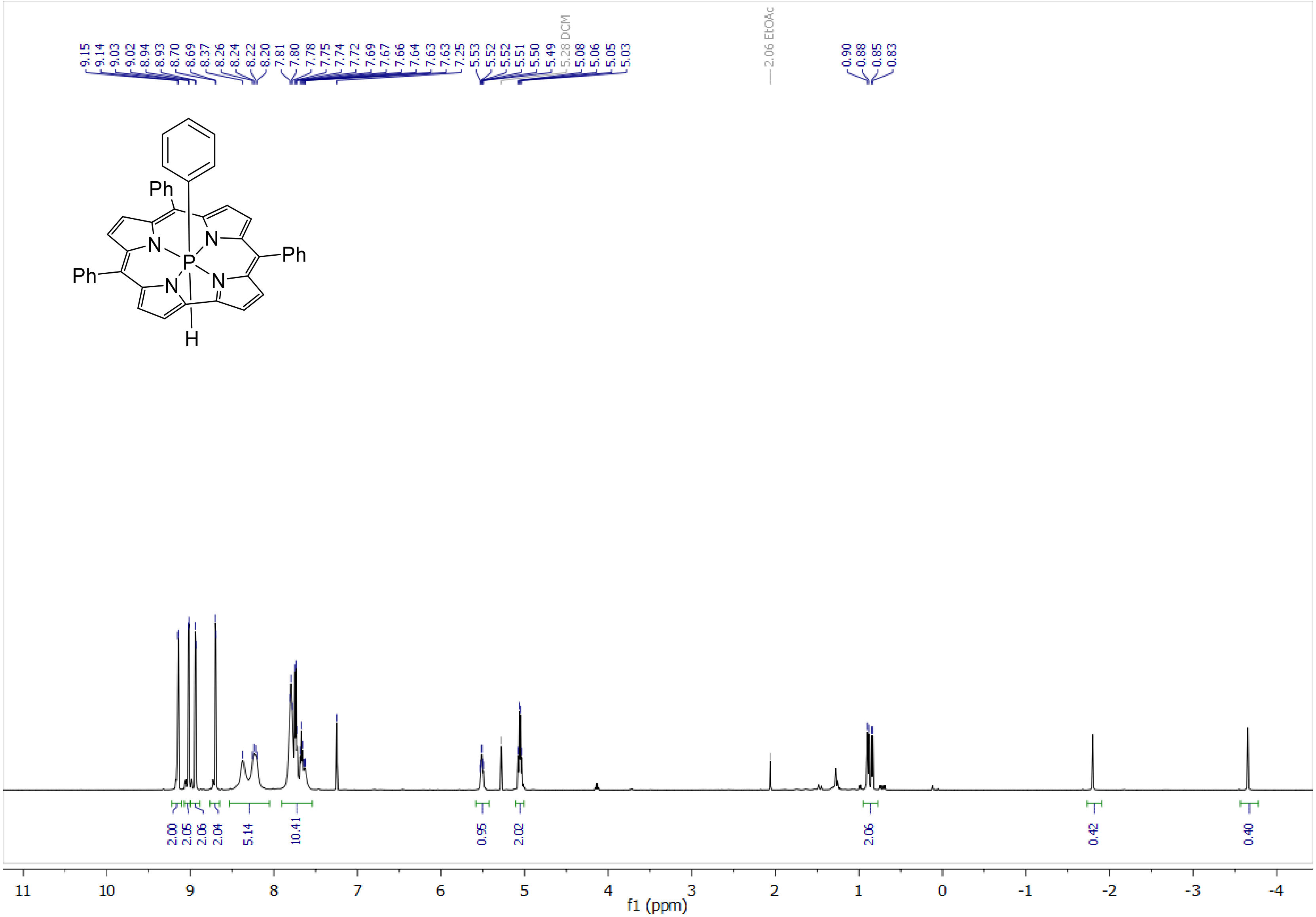

Figure S10. *^{1}H NMR spectrum of* ***1•H****.*

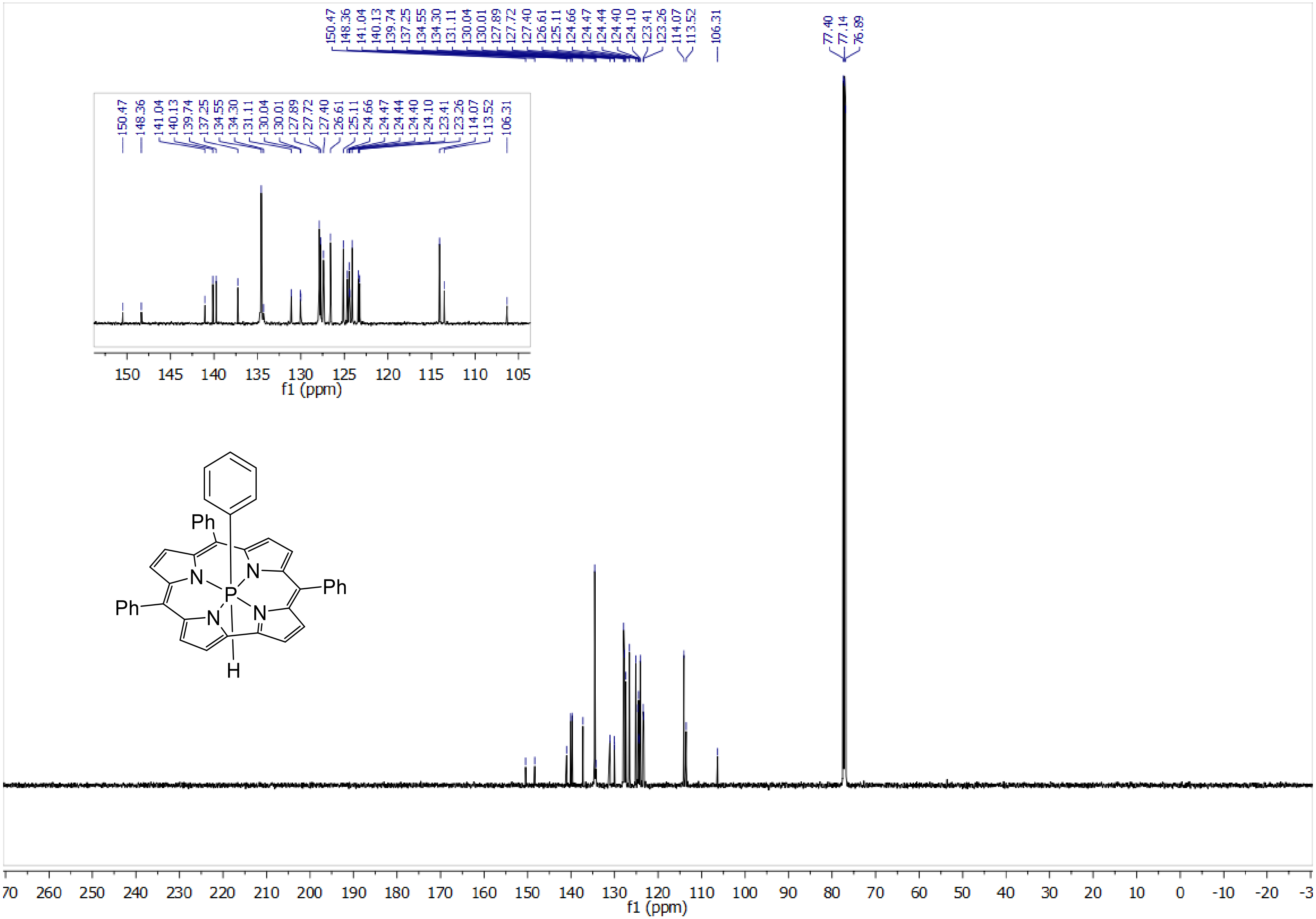

Figure S11. *$^{13}C\{^1H\}$ NMR spectrum of* ***1•H****.*

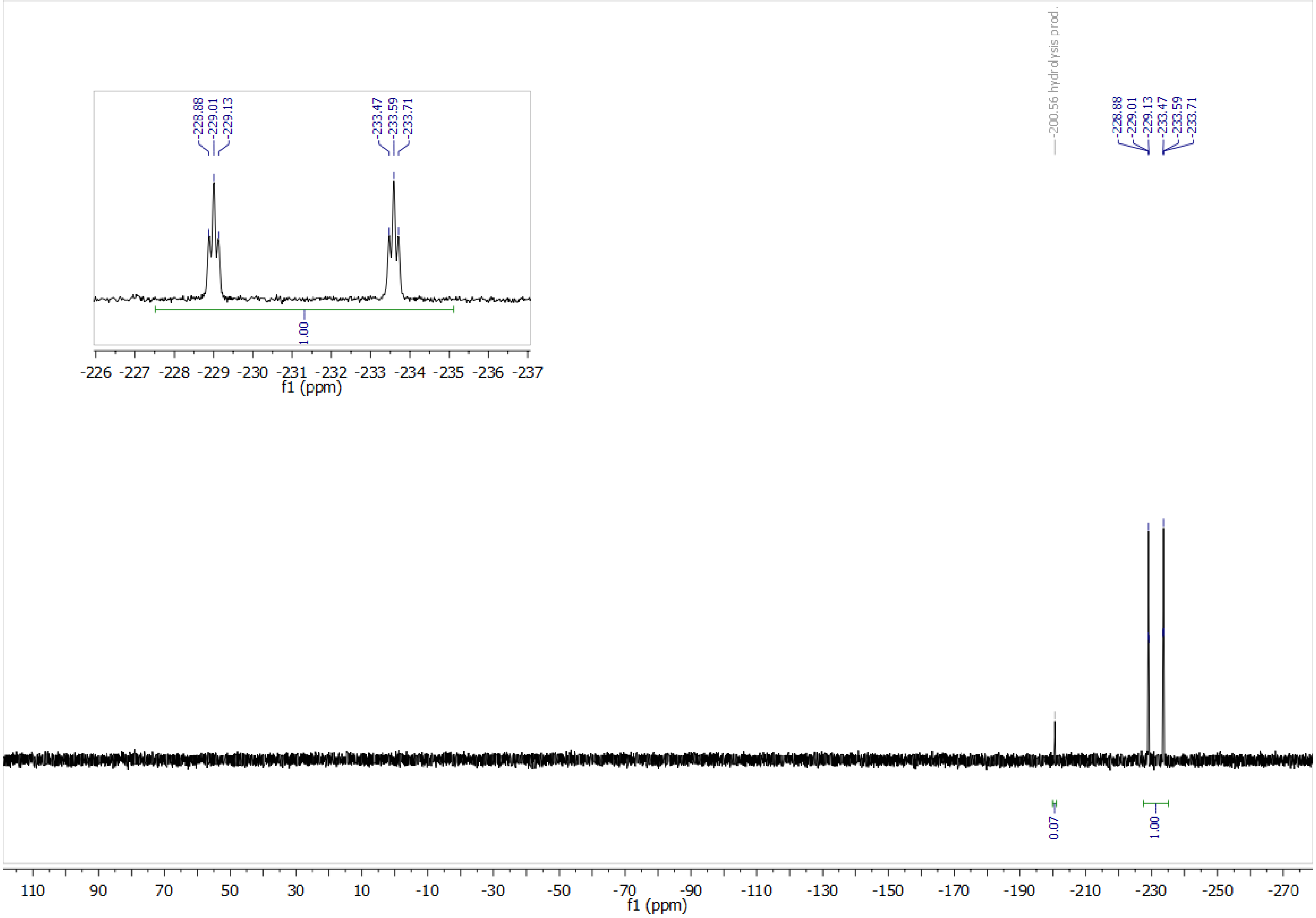

Figure S12. *^{31}P NMR spectrum of **1•H**. The compound containing a P–OH bond is evident at δ –200.6 ppm.*

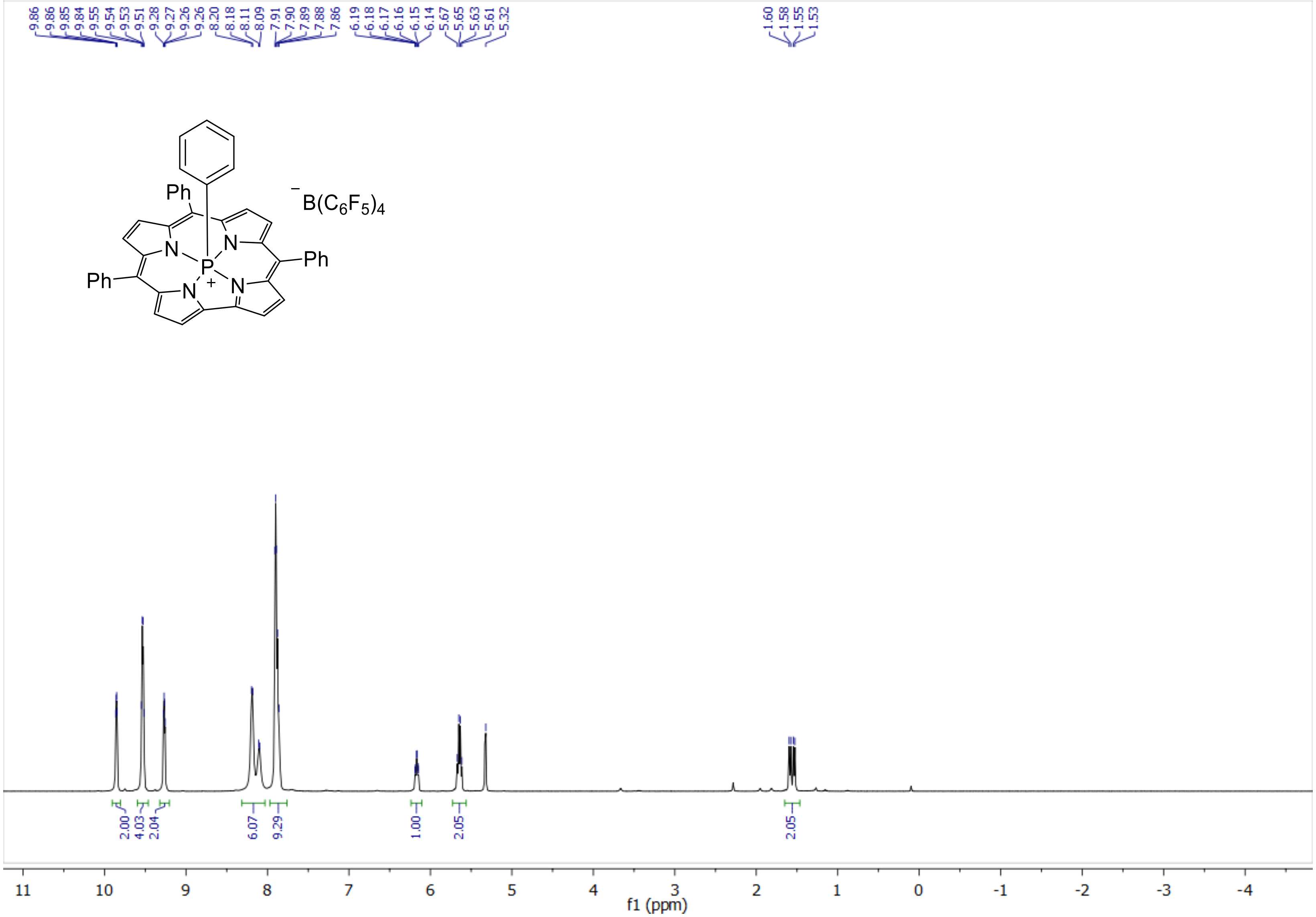

Figure S13. ^{1}H NMR spectrum of **1**$^{+}$.

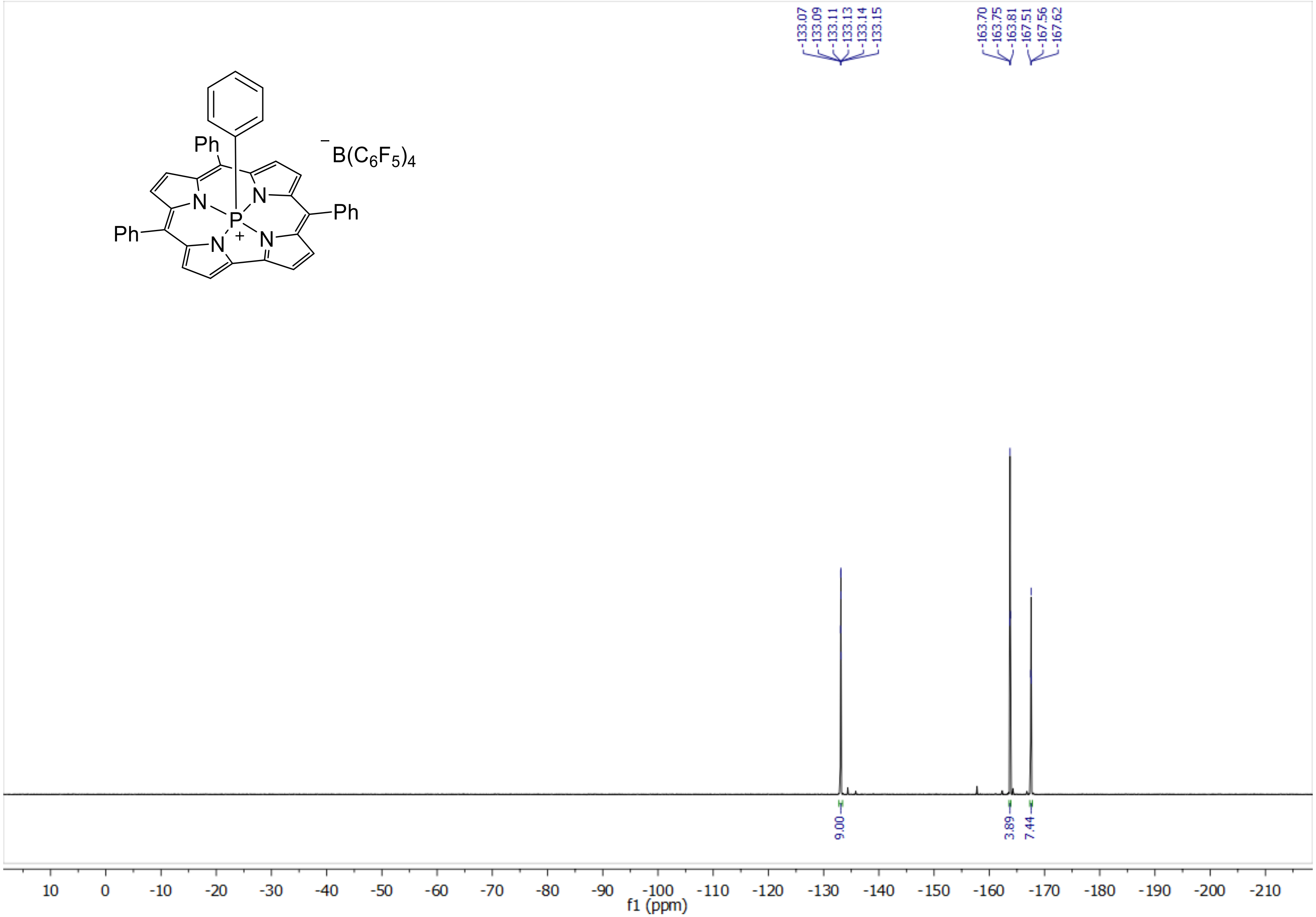

Figure S14. *^{19}F NMR spectrum of **1**$^+$.*

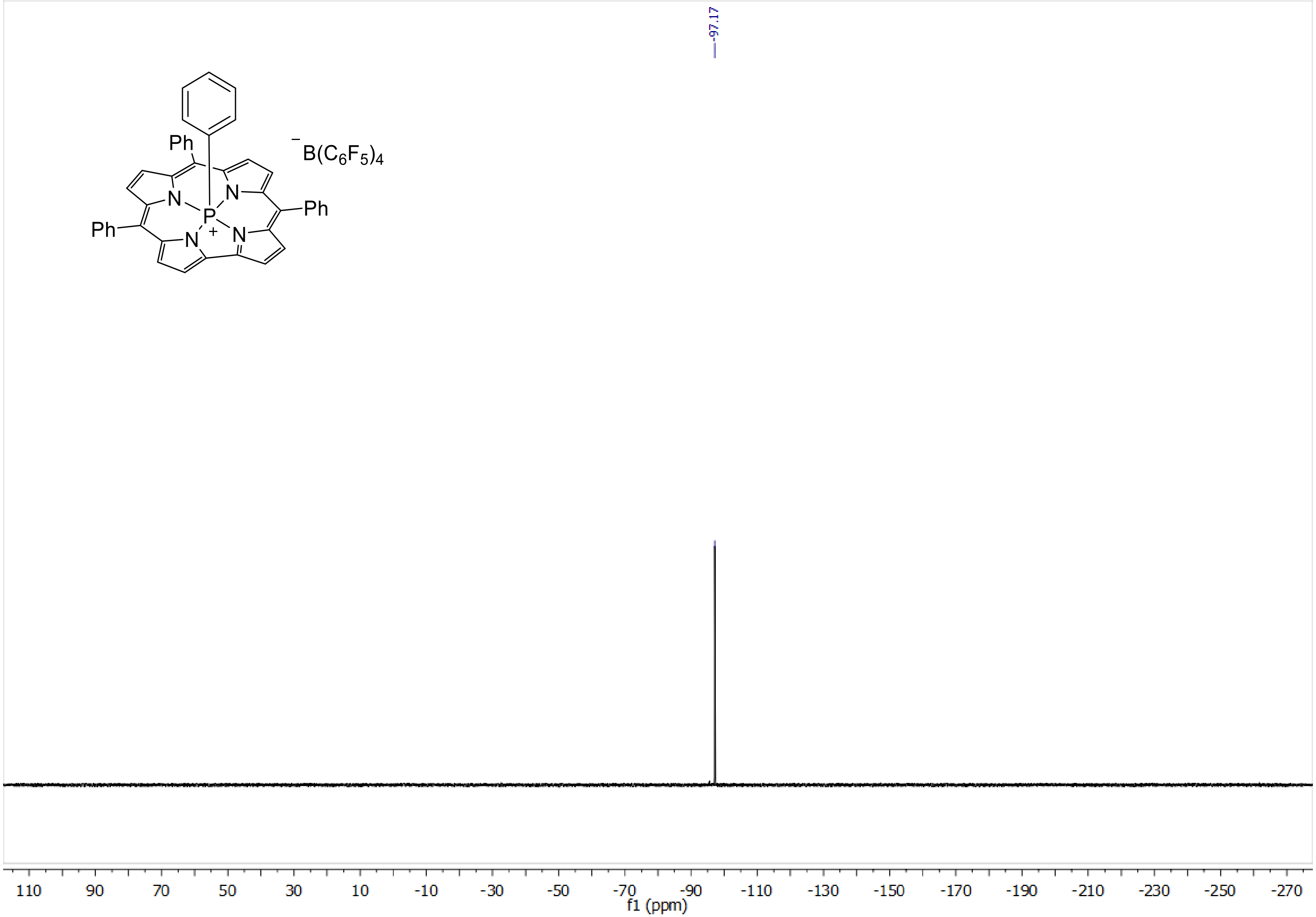

Figure S15. *$^{31}P\{^{1}H\}$ NMR spectrum of* ***1***$^{+}$.

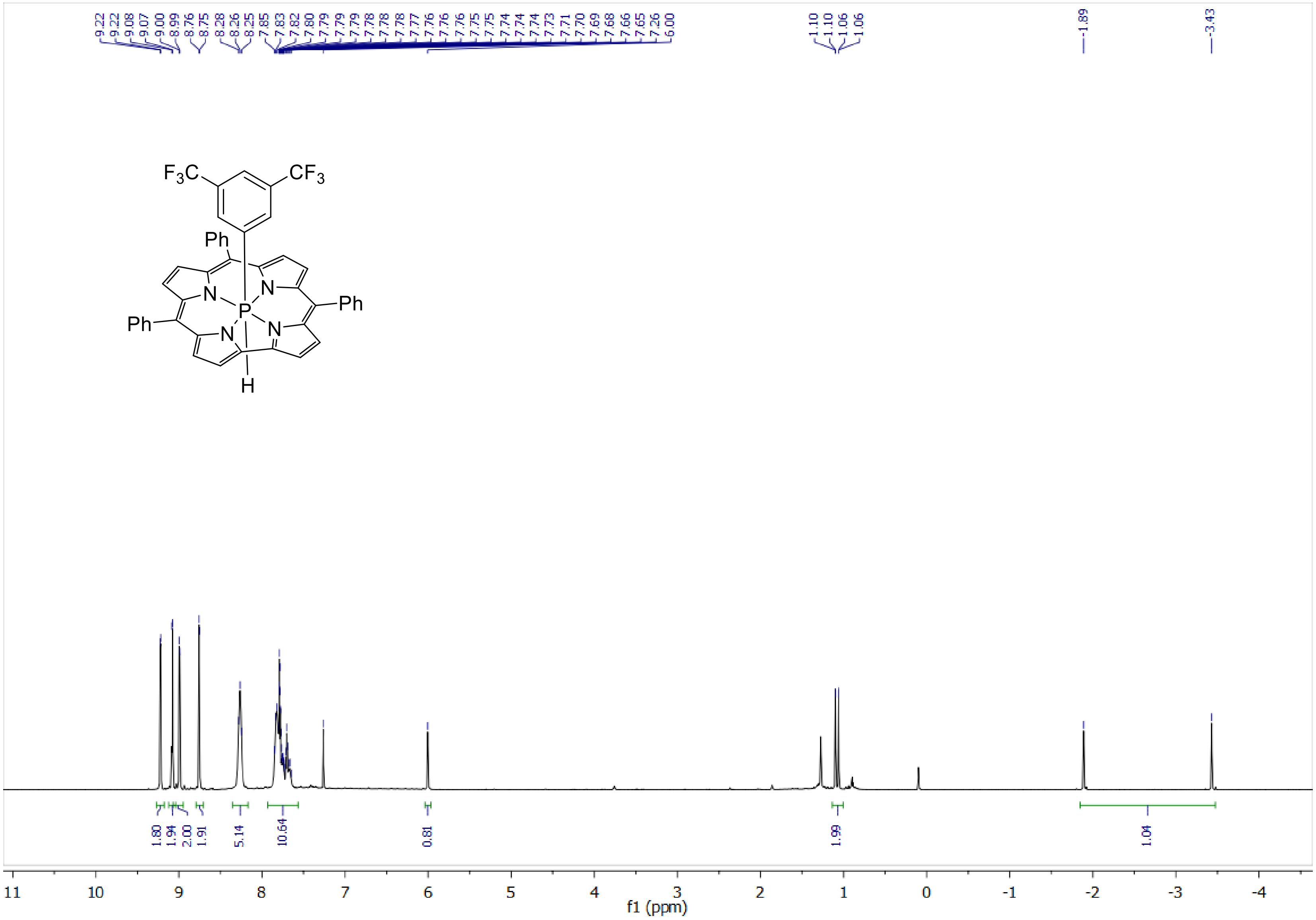

Figure S16. *^{1}H NMR spectrum of **2•H**.*

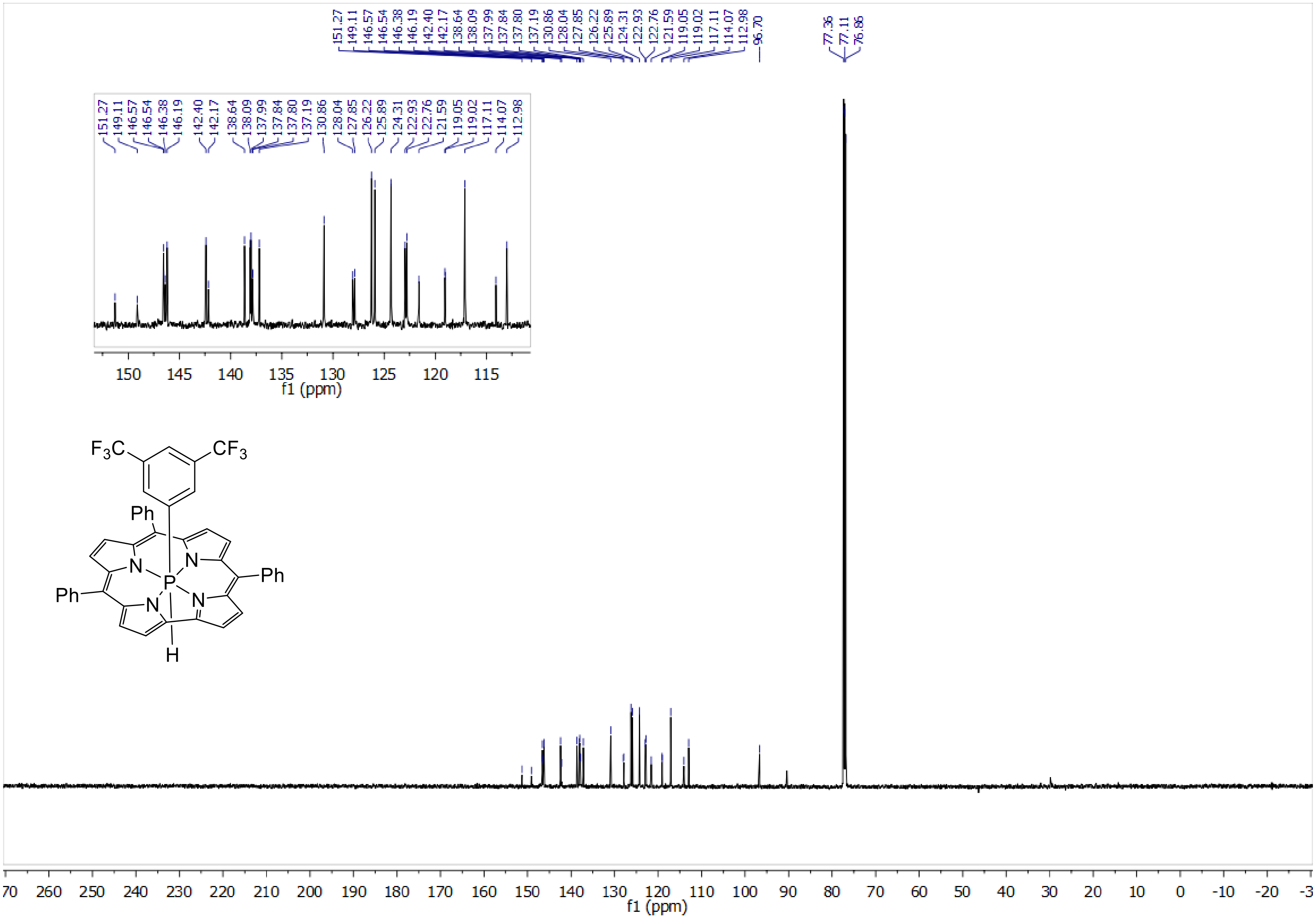

Figure S17. *$^{13}C\{^{1}H,^{19}F\}$ NMR spectrum of* ***2•H****.*

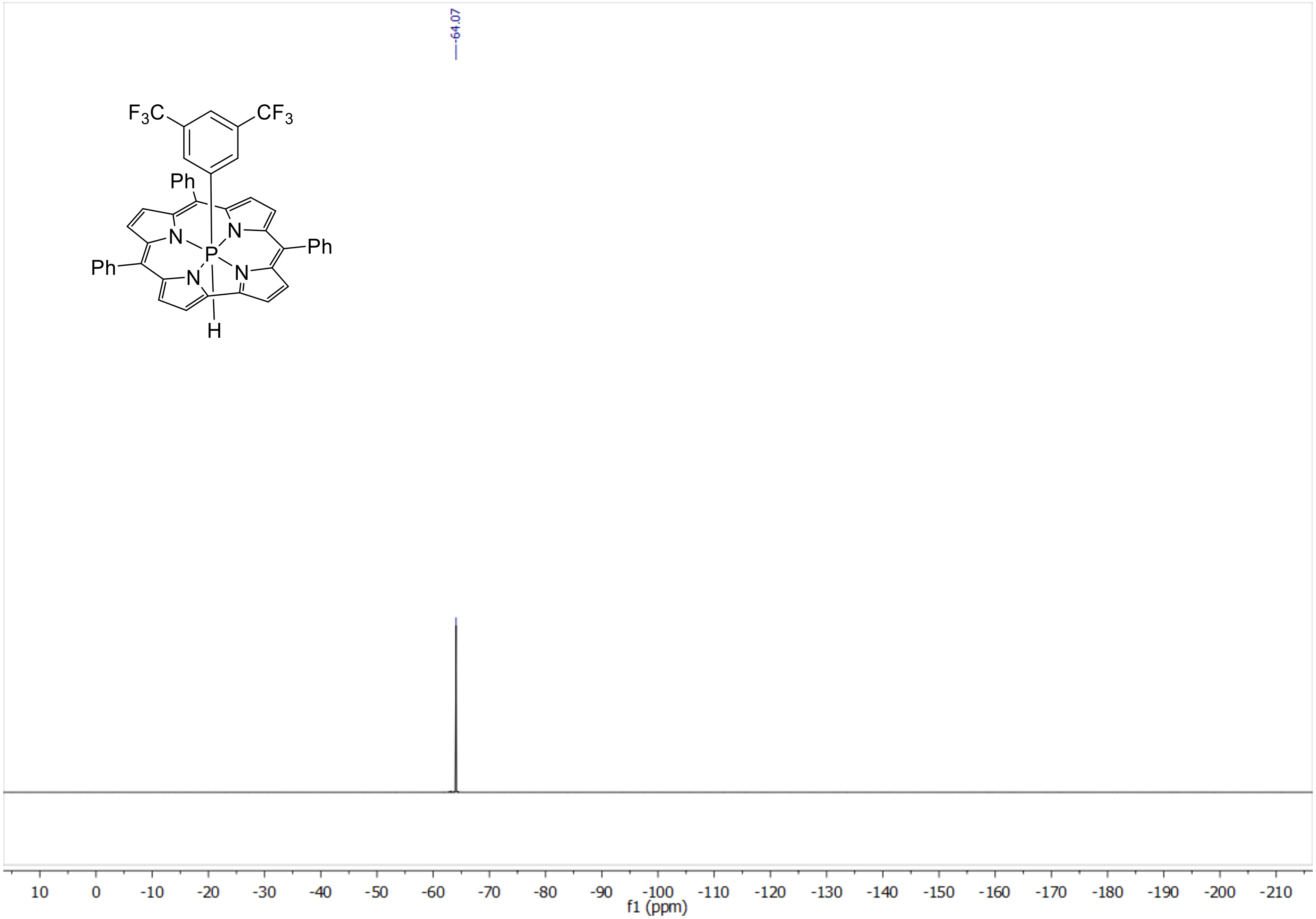

Figure S18. *^{19}F NMR spectrum of* ***2•H****.*

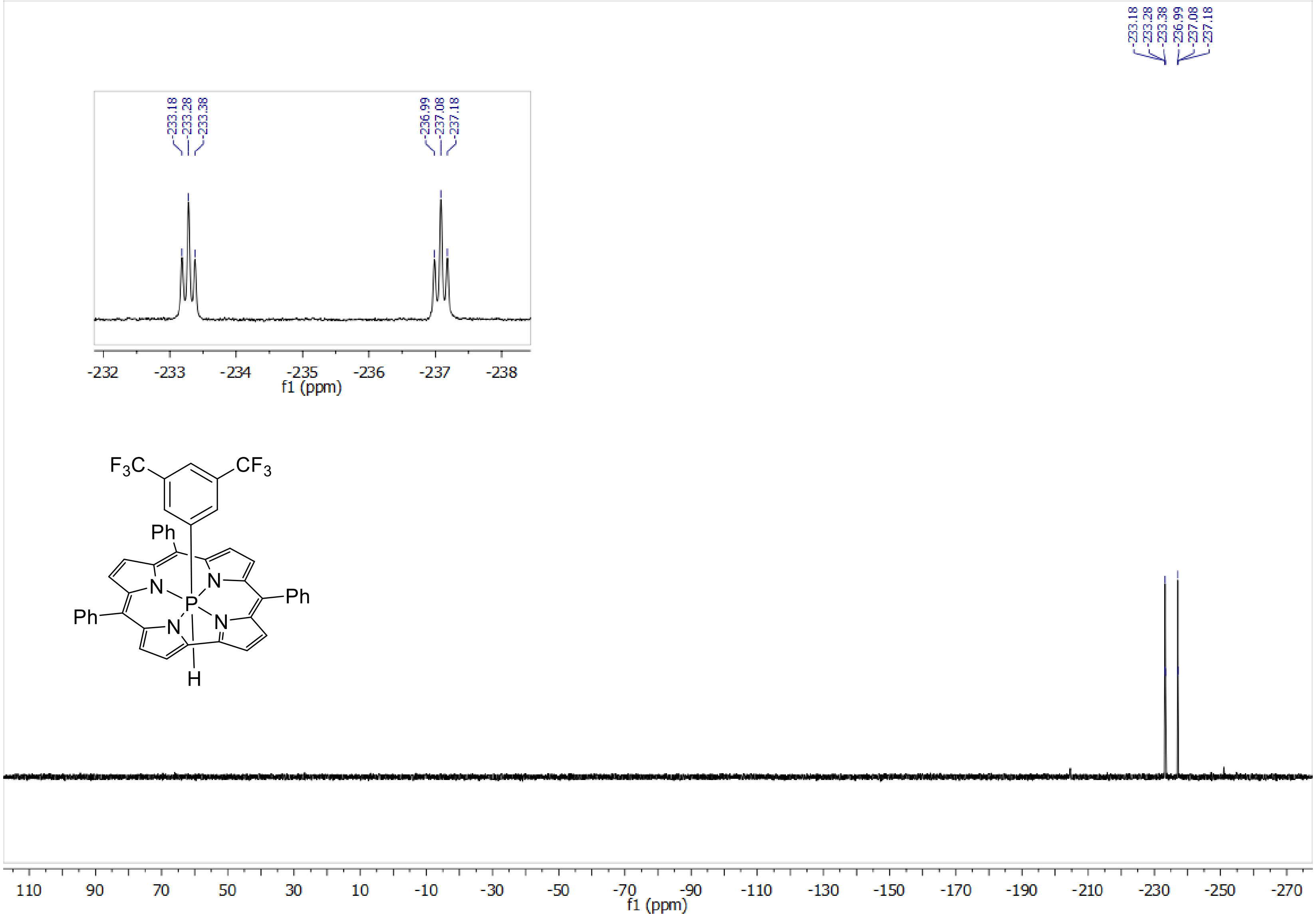

Figure S19. *^{31}P NMR spectrum of **2•H**.*

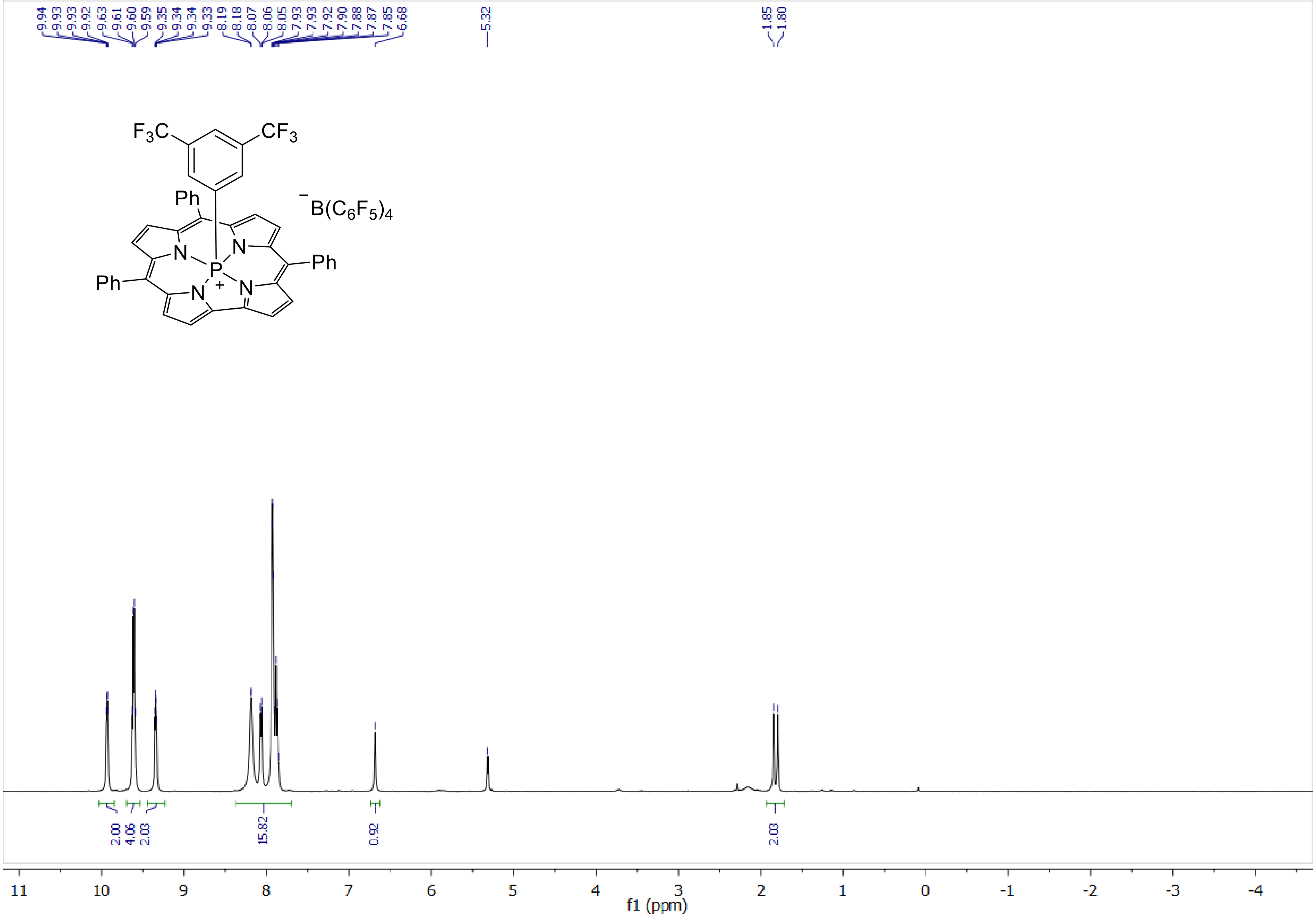

Figure S20. *^{1}H NMR spectrum of **2**$^{+}$.*

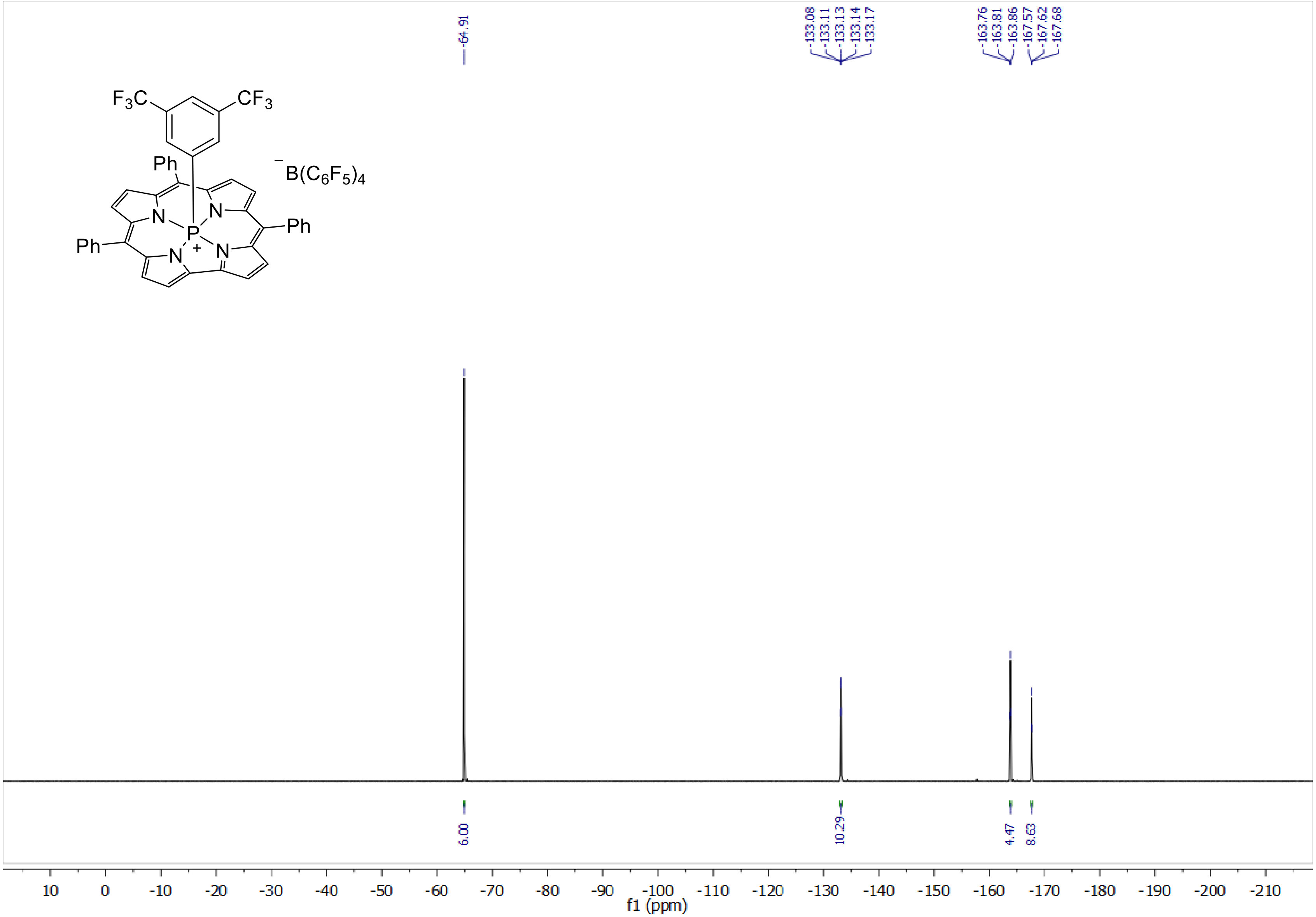

Figure S21. *^{19}F NMR spectrum of* ***2***$^+$.

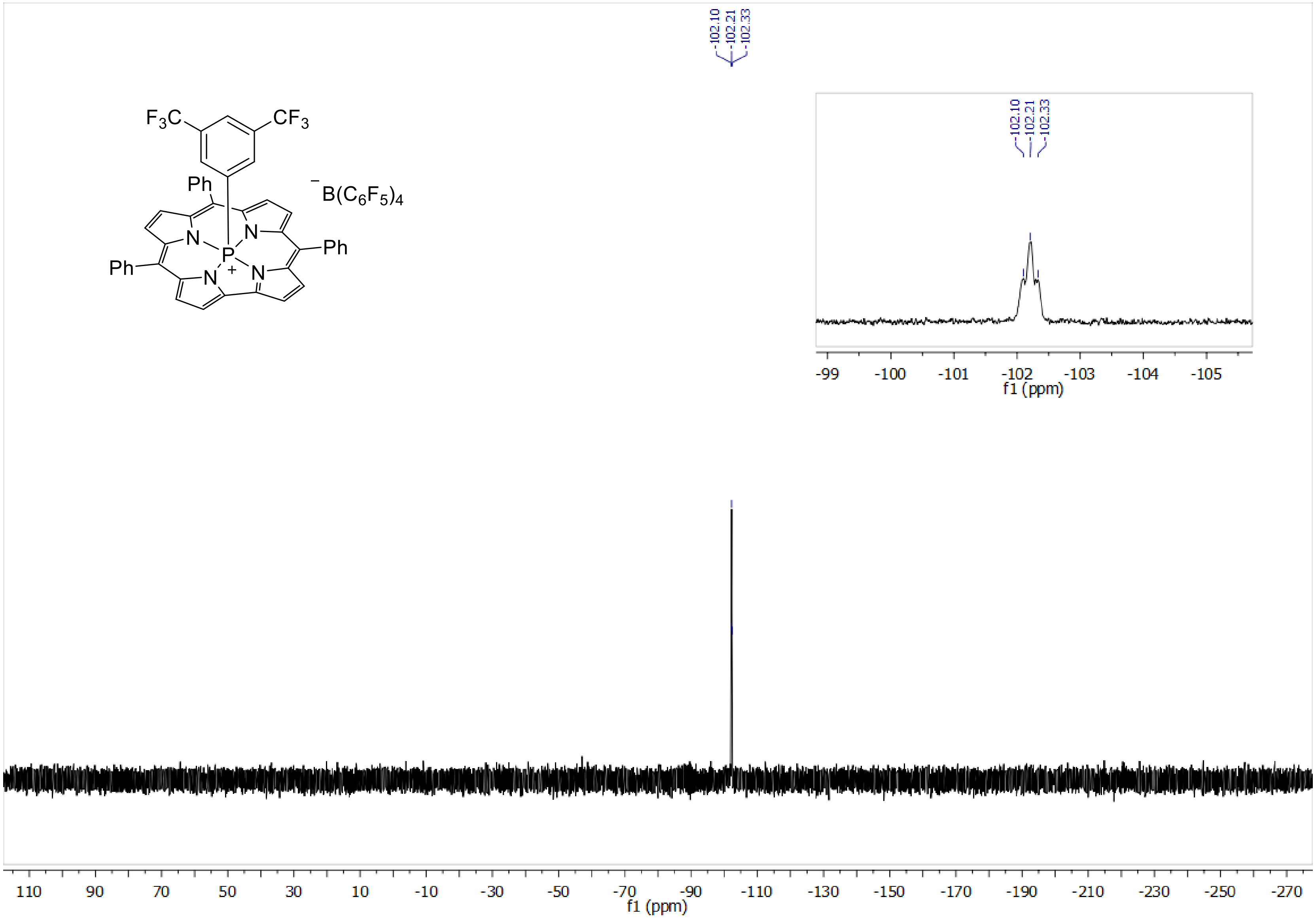

Figure S22. *^{31}P NMR spectrum of **2**⁺.*

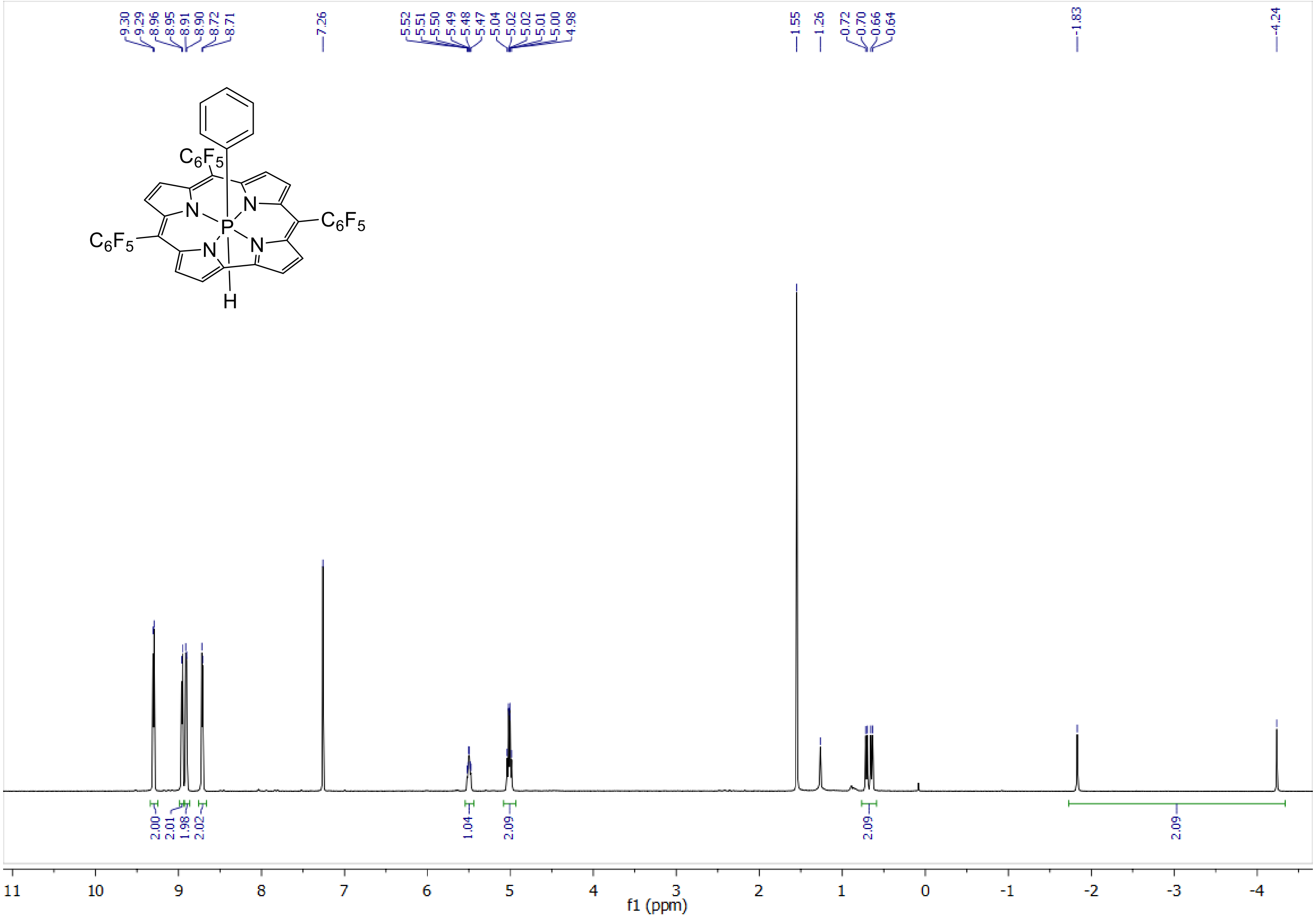

Figure S23. *^{1}H NMR spectrum of* ***3•H****.*

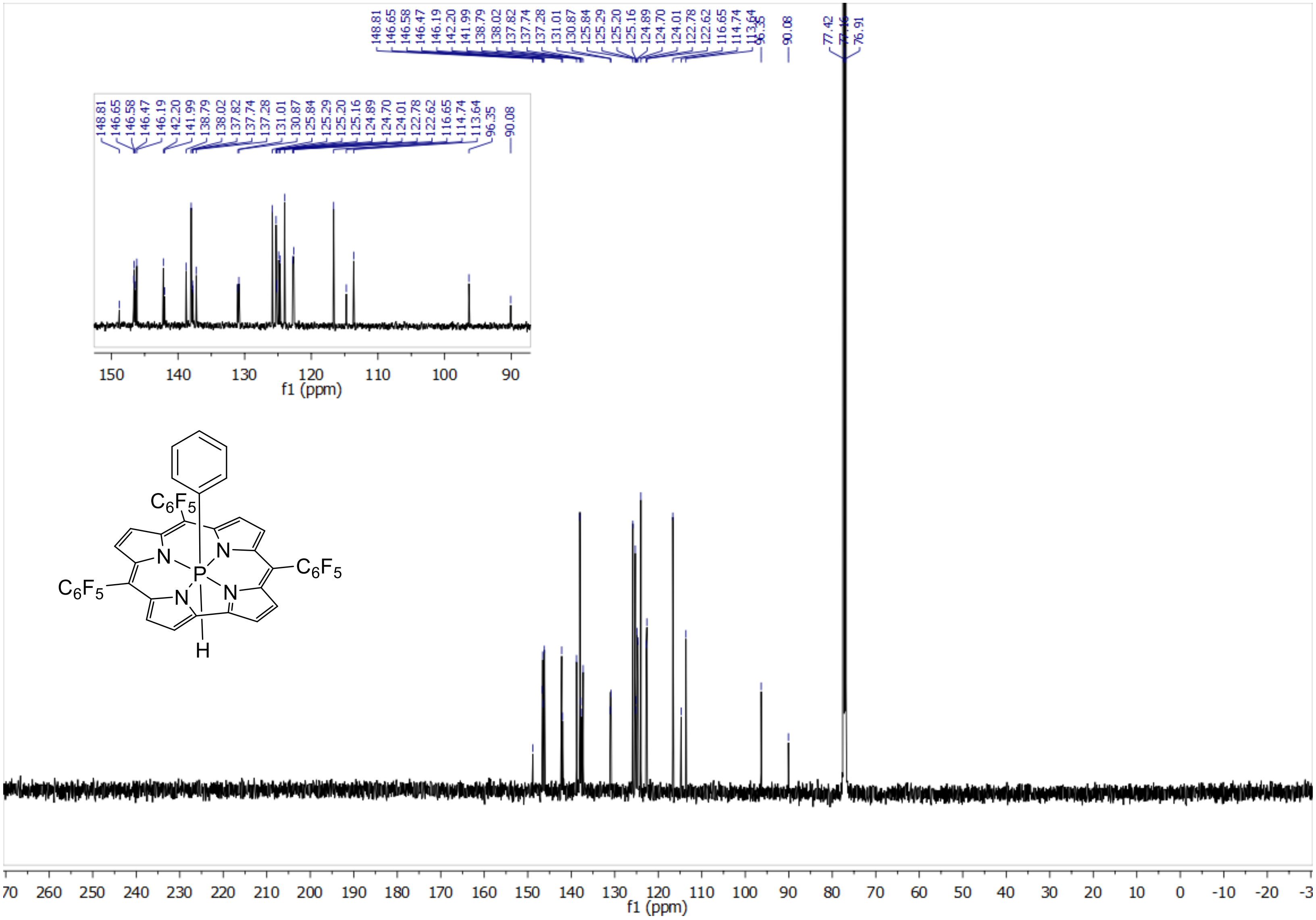

Figure S24. $^{13}C\{^{1}H,^{19}F\}$ NMR spectrum of **3•H**.

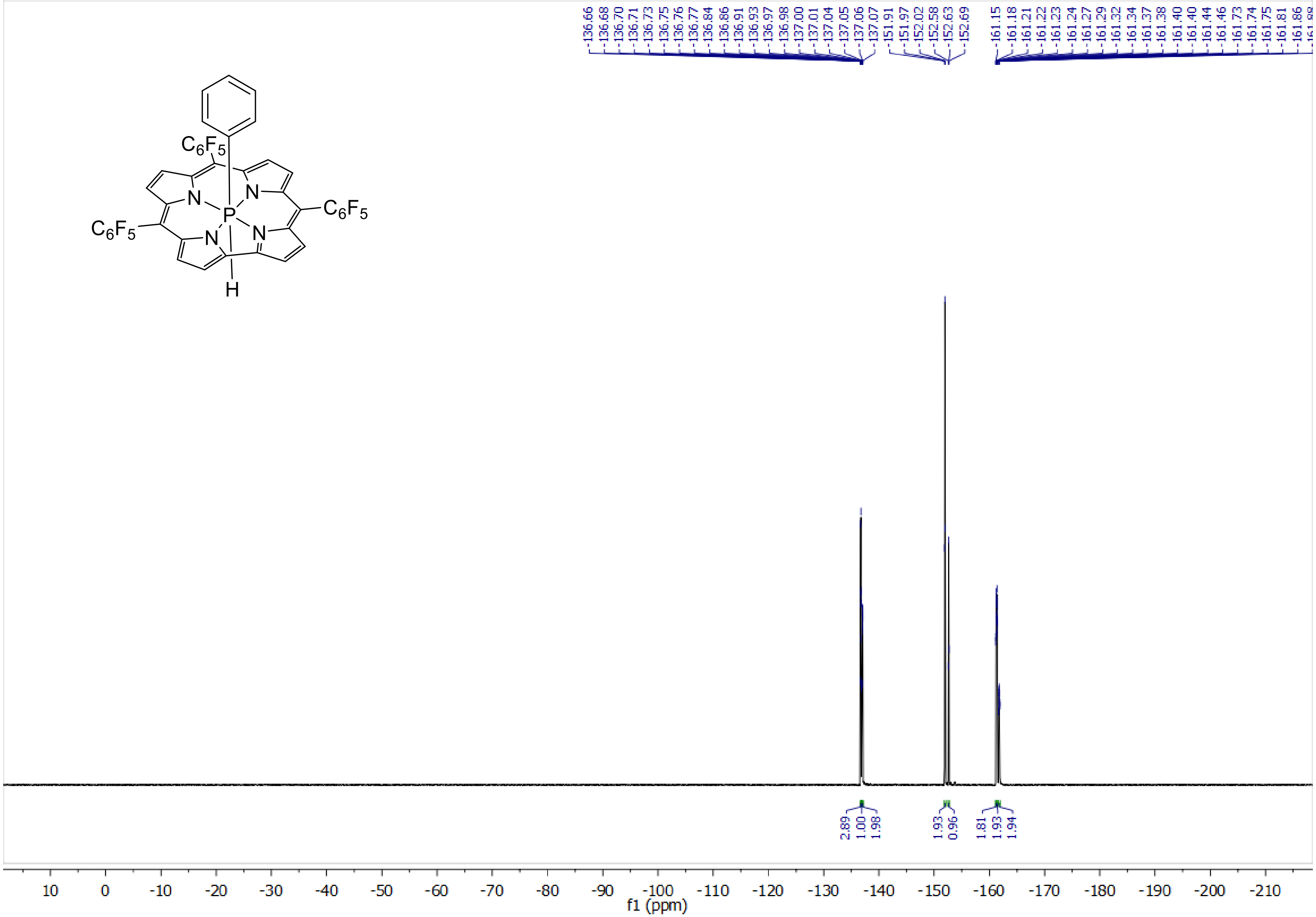

Figure S25. *^{19}F NMR spectrum of* ***3•H.***

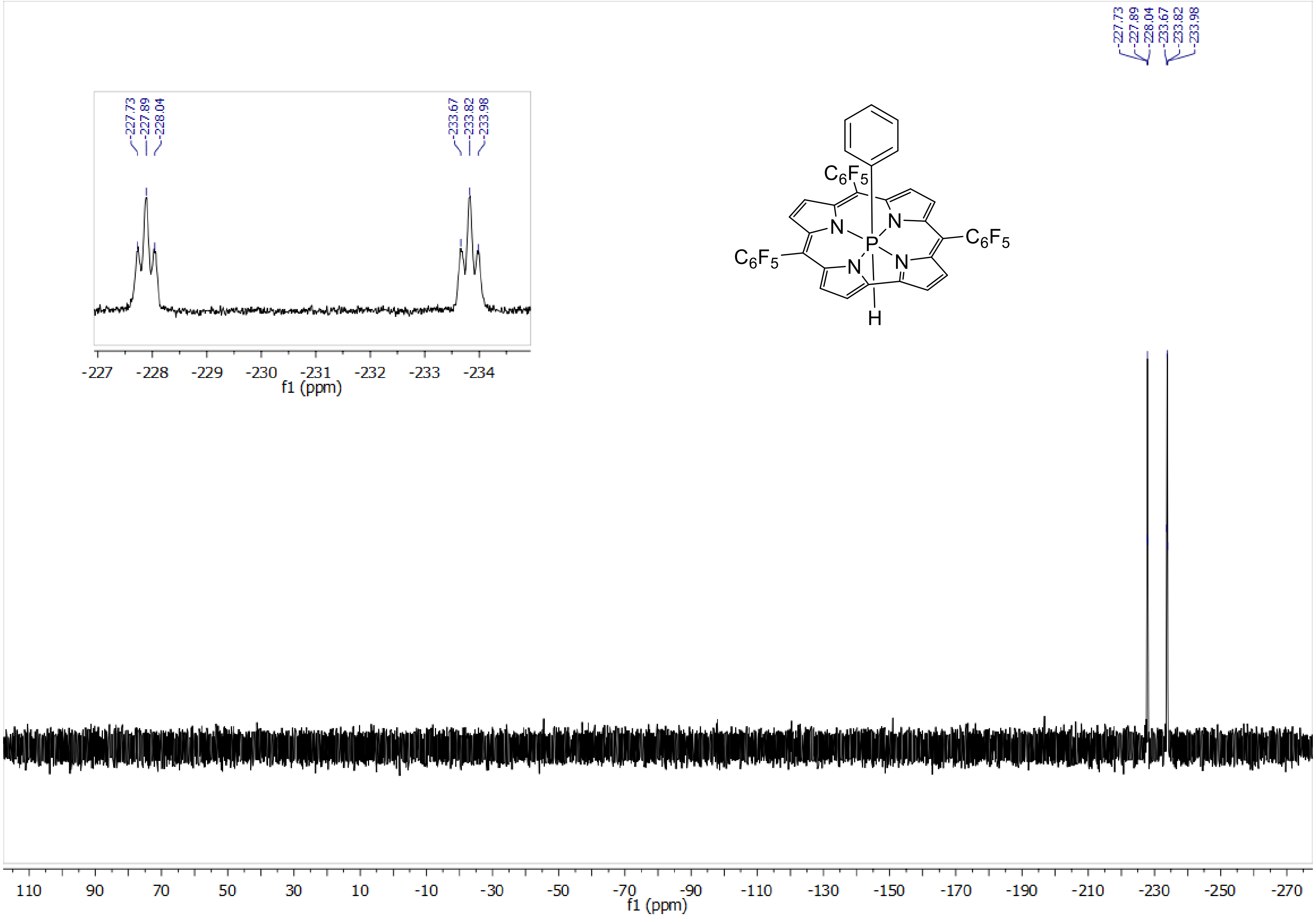

Figure S26. *^{31}P NMR spectrum of **3•H**.*

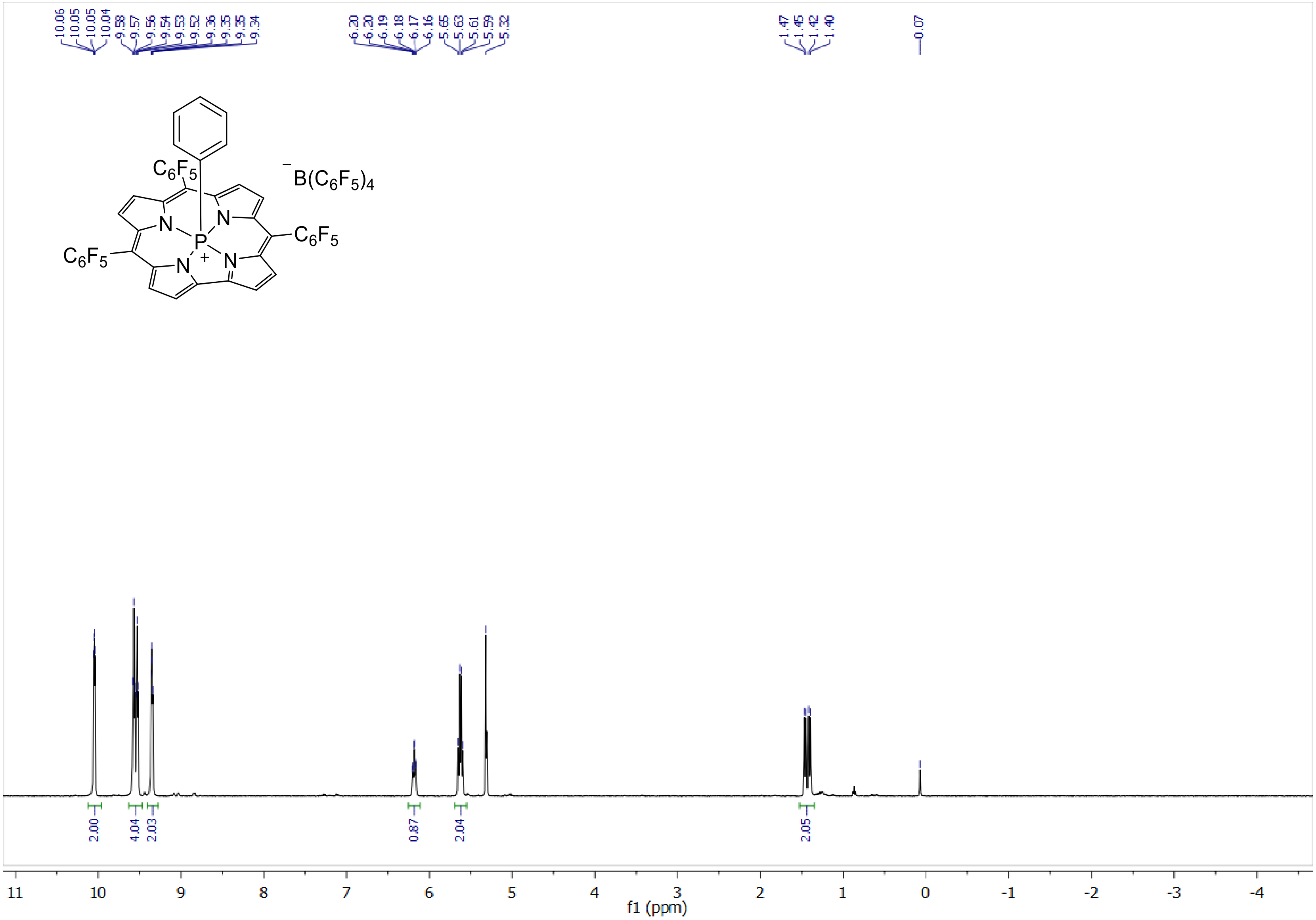

Figure S27. *^{1}H NMR spectrum of **3**$^{+}$.*

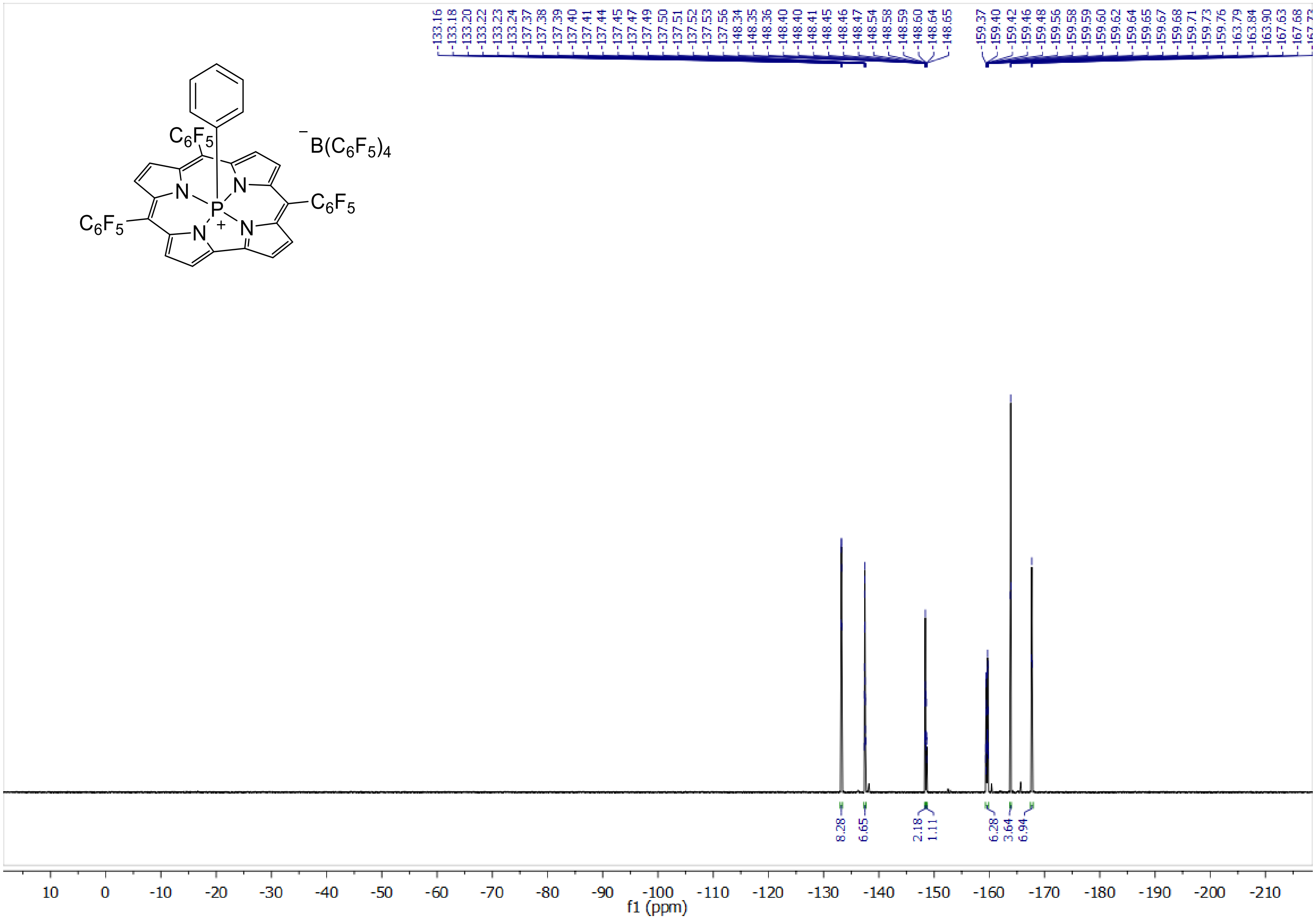

Figure S28. *^{19}F NMR spectrum of* ***3***$^{+}$.

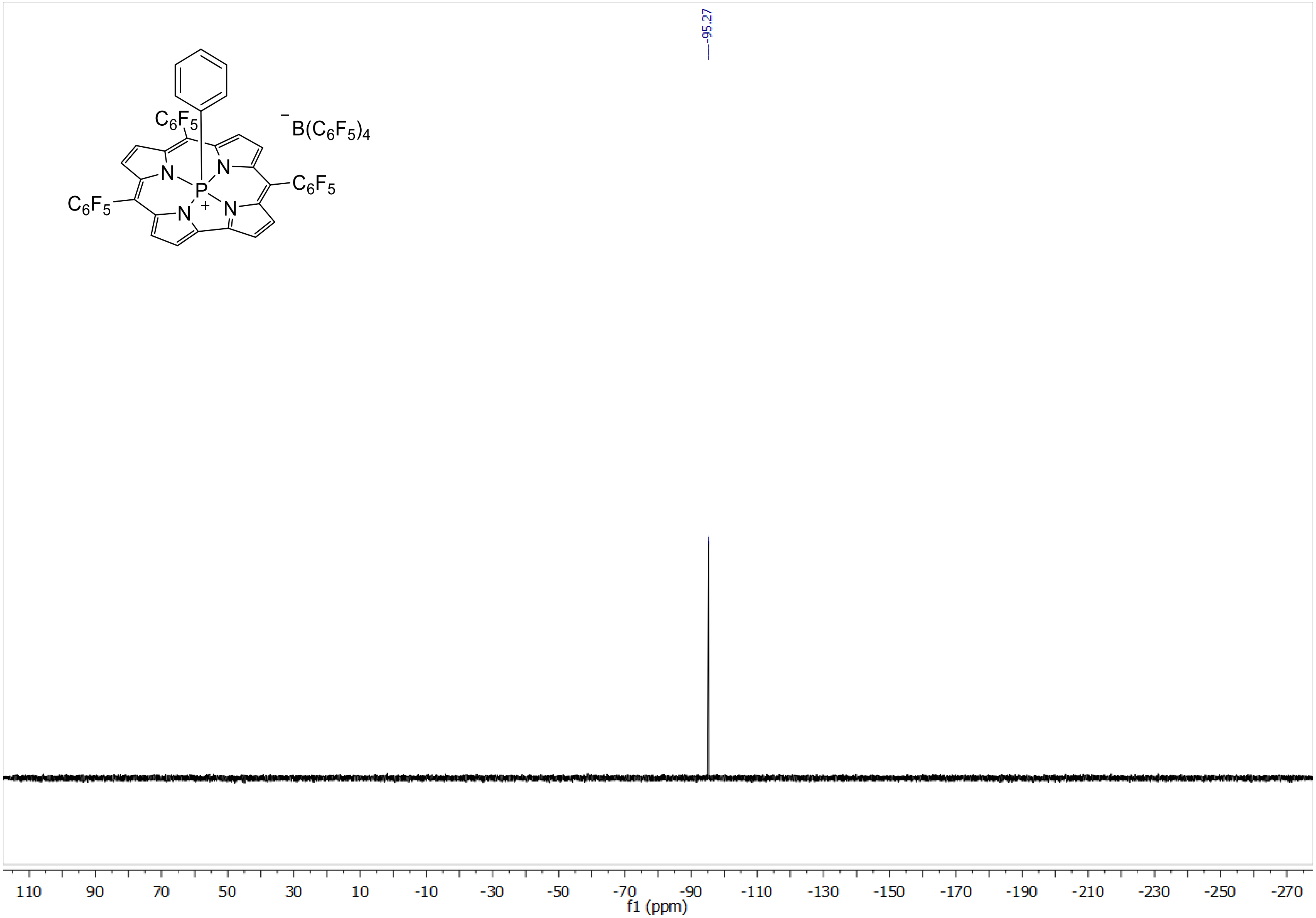

Figure S29. *$^{31}P\{^{1}H\}$ NMR spectrum of **3^{+}**.*

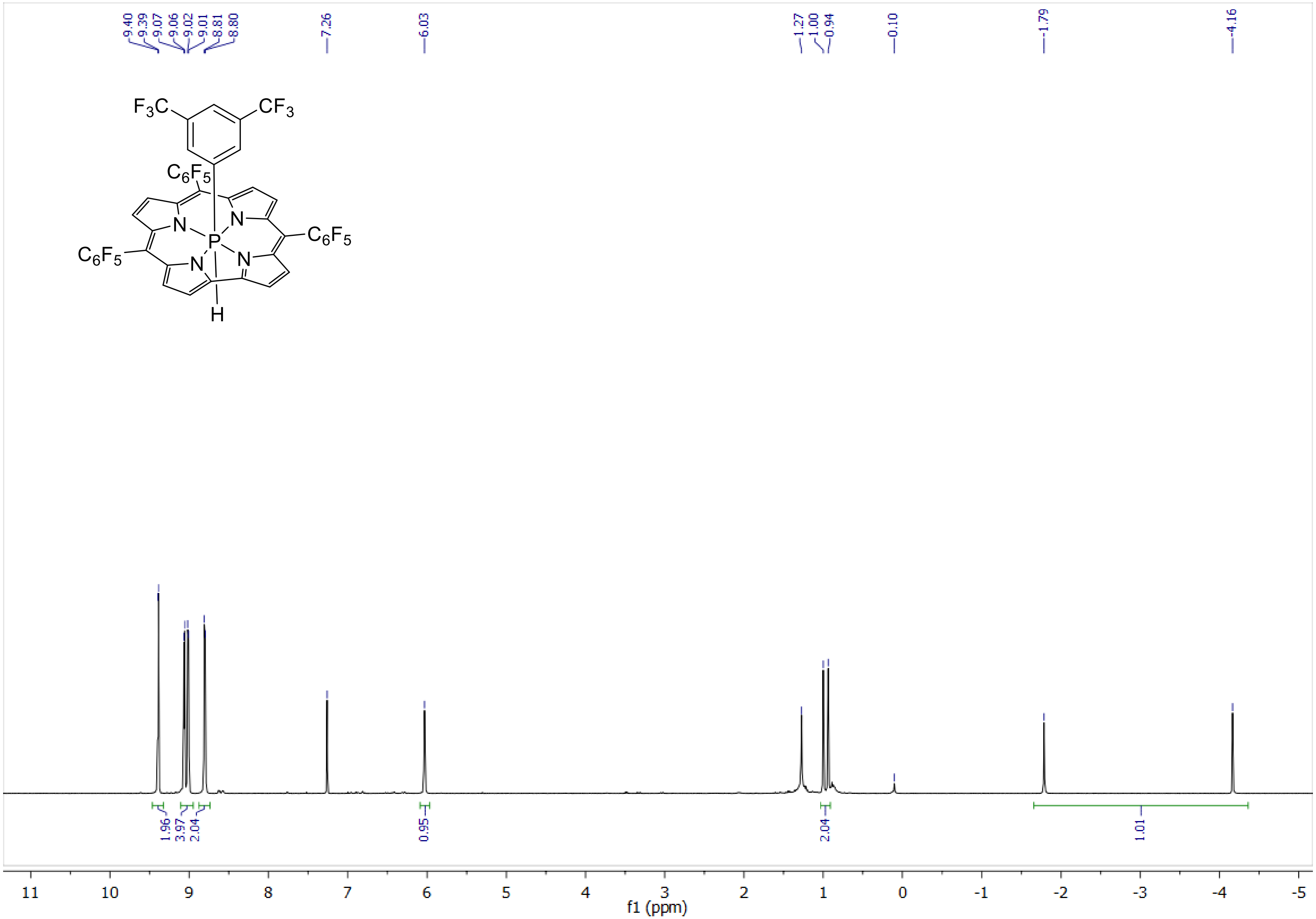

Figure S30. *^{1}H NMR spectrum of **4•H**.*

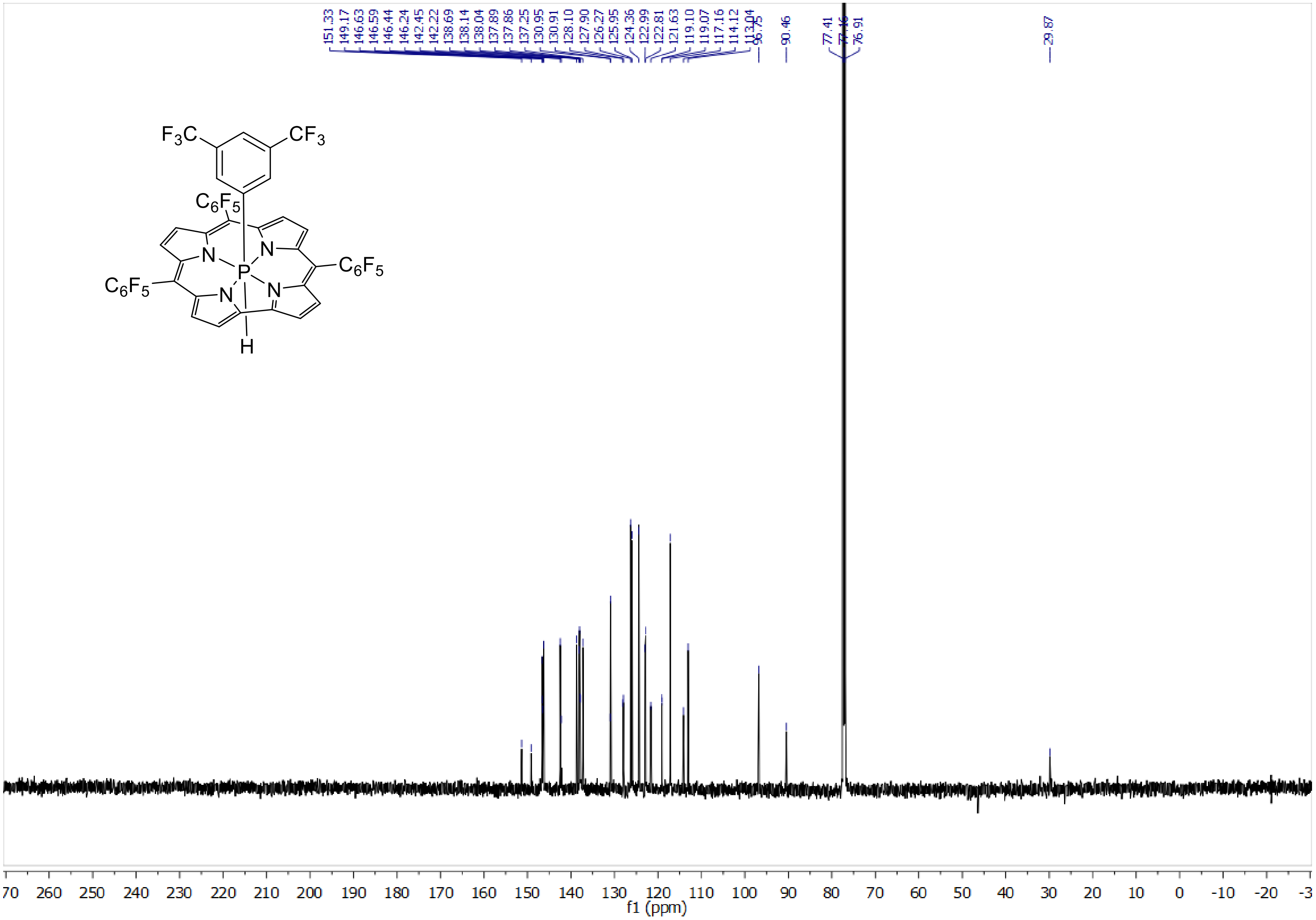

Figure S31. *$^{13}C\{^{1}H,^{19}F\}$ NMR spectrum of **4•H**.*

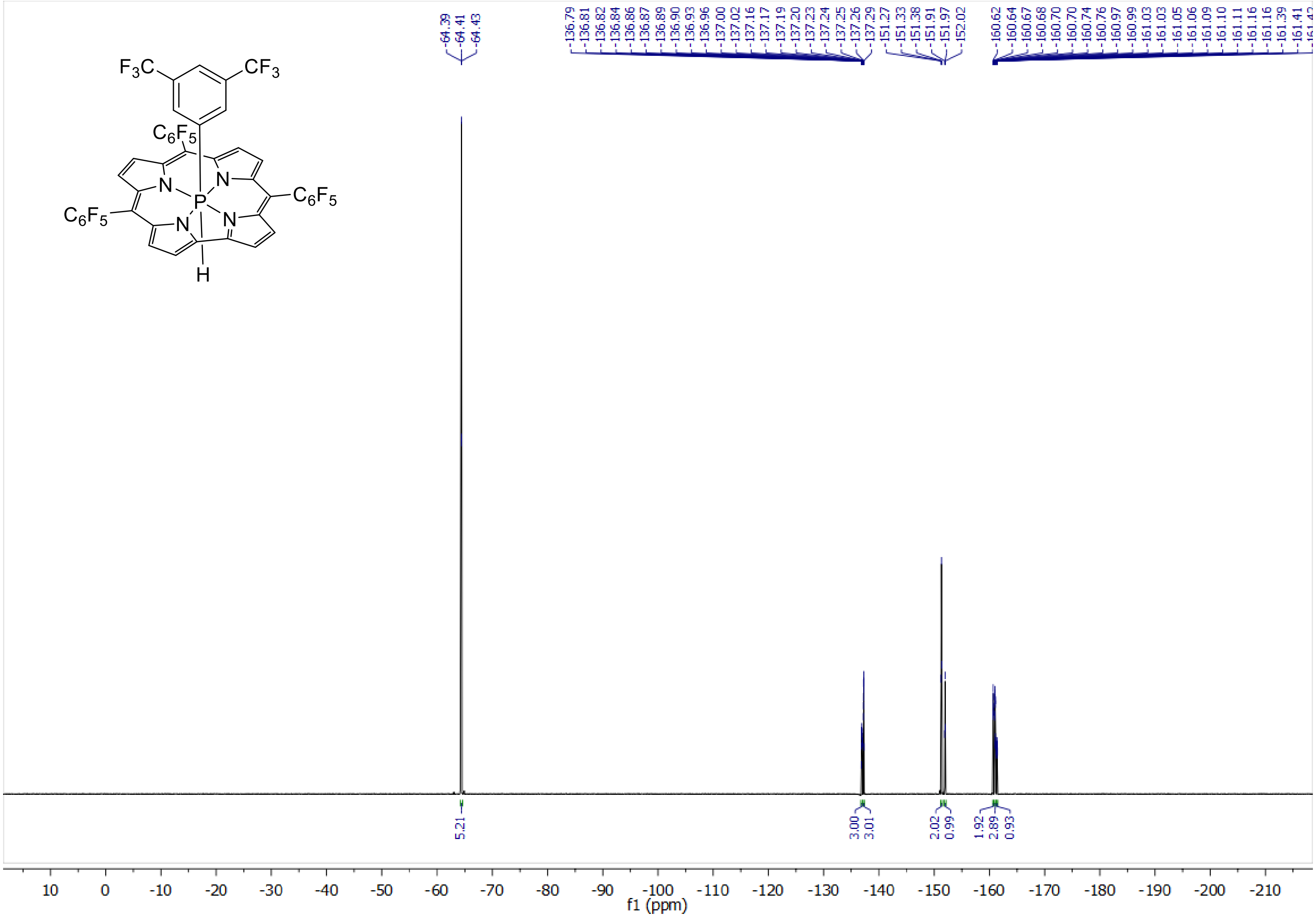

Figure S32. *^{19}F NMR spectrum of* ***4•H****.*

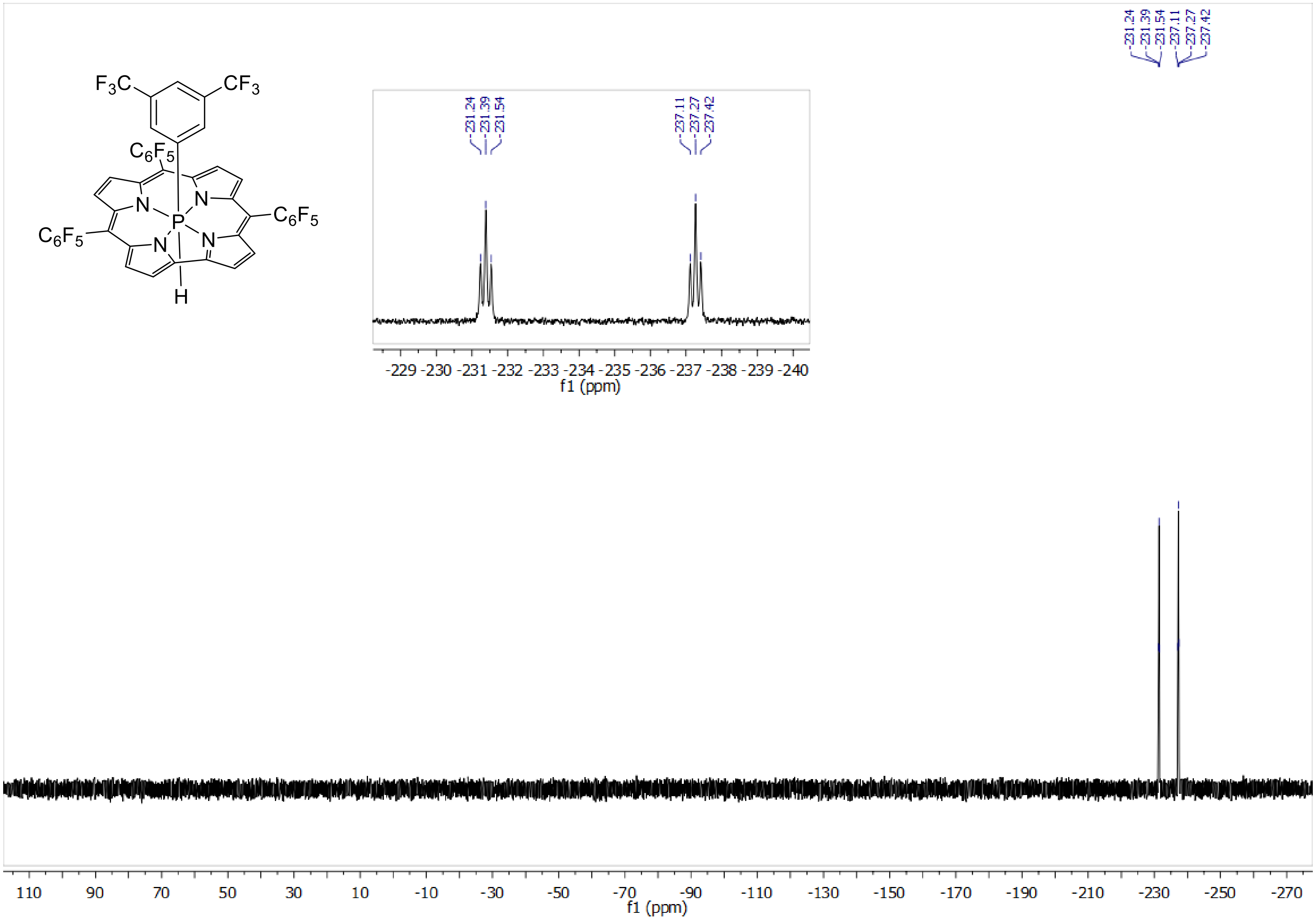

Figure S33. *^{31}P NMR spectrum of **4•H**.*

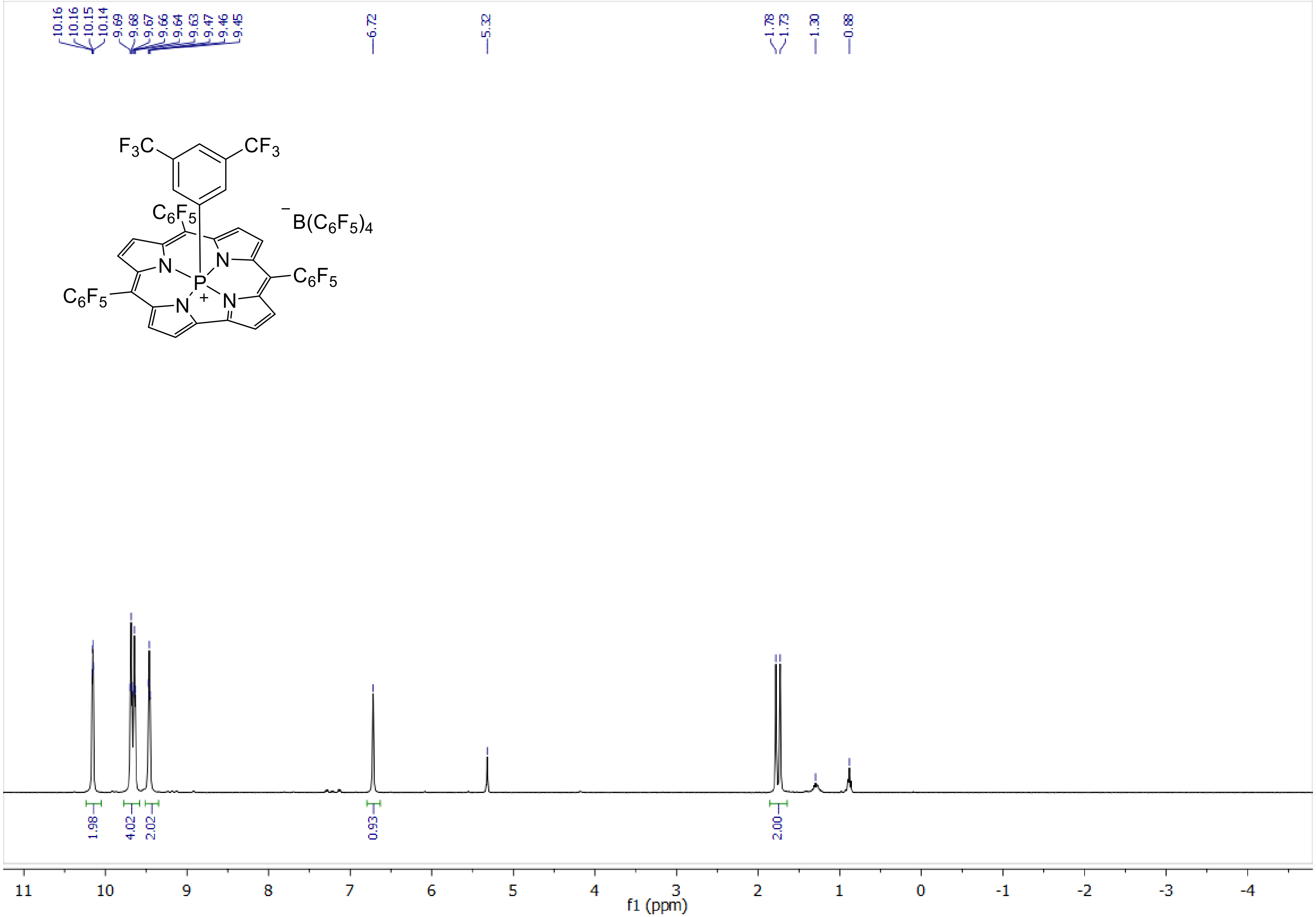

Figure S34. *^{1}H NMR spectrum of **4^+**.*

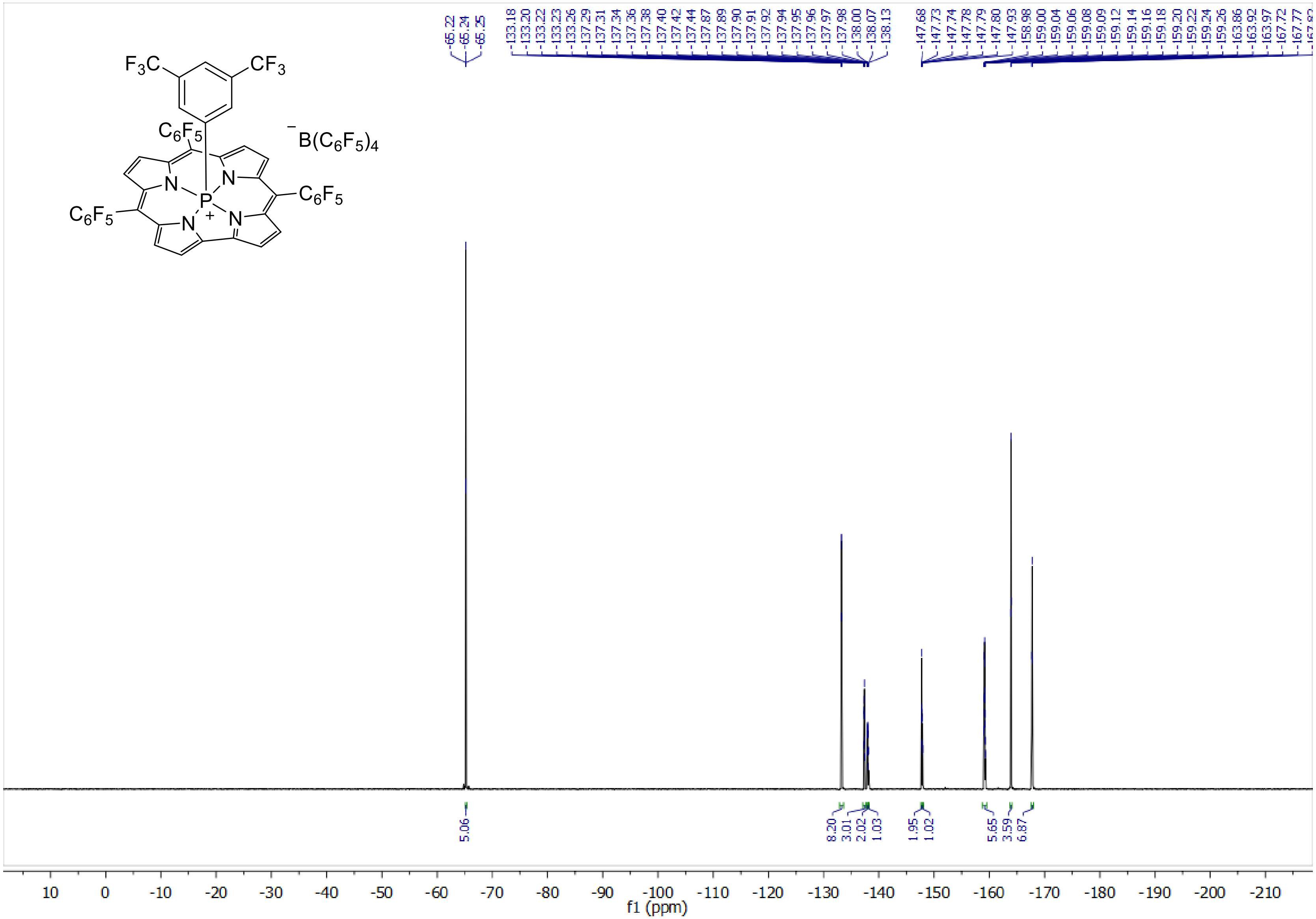

Figure S35. *^{19}F NMR spectrum of **4**$^{+}$.*

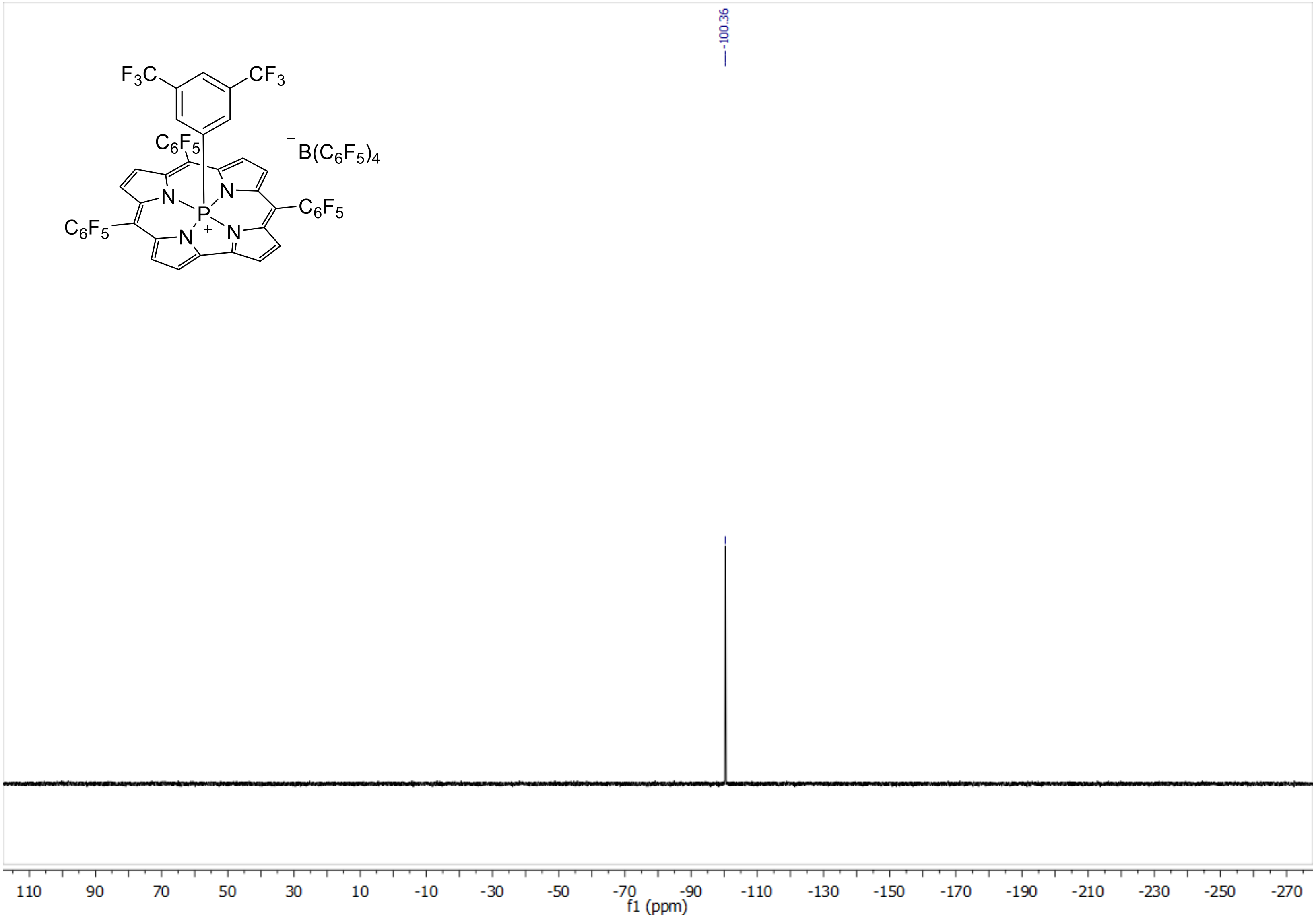

Figure S36. *^{31}P NMR spectrum of **4^{+}**.*

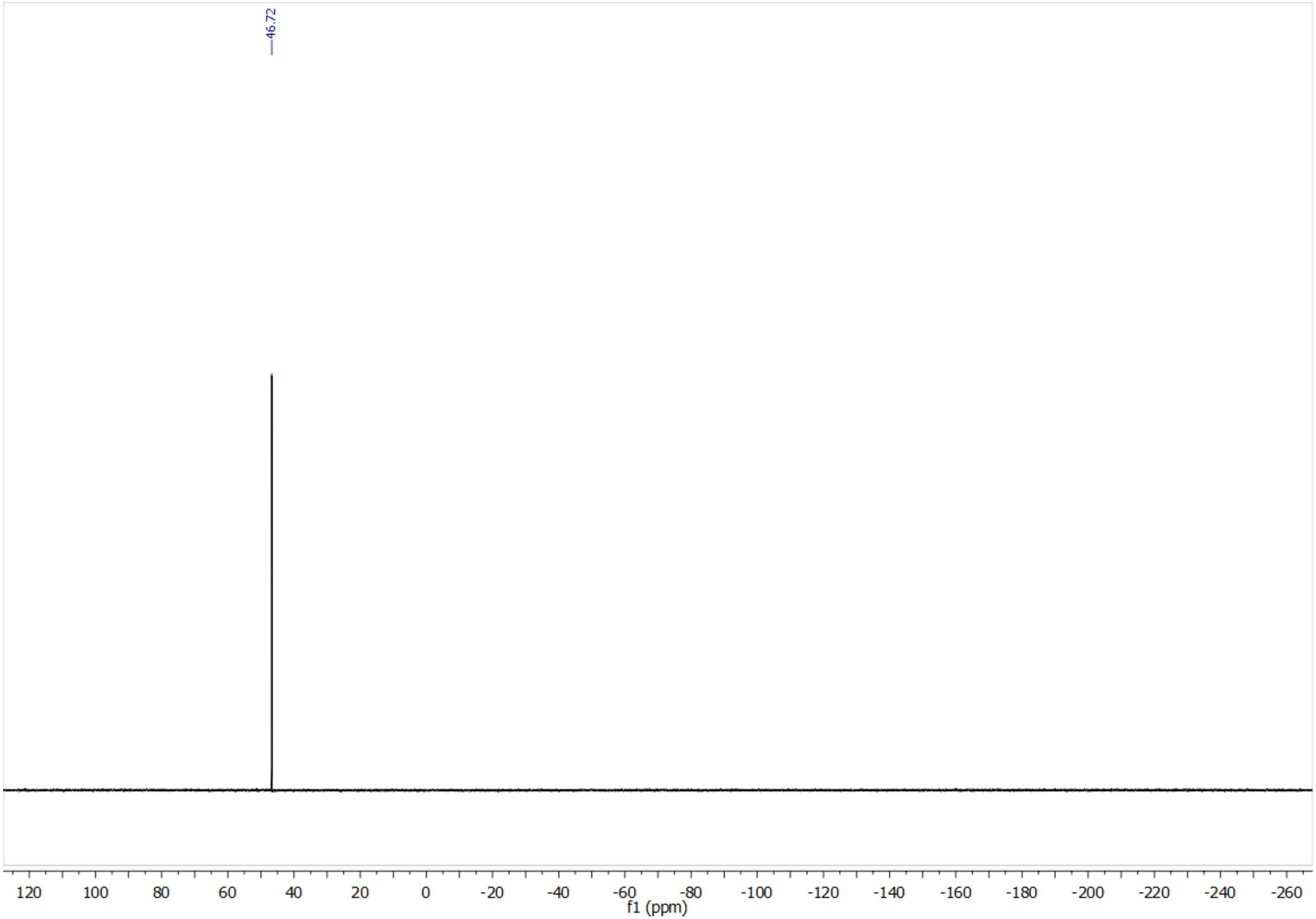

Figure S37. *^{31}P{^{1}H} NMR spectrum of tri-*n*-octylphosphine oxide.*

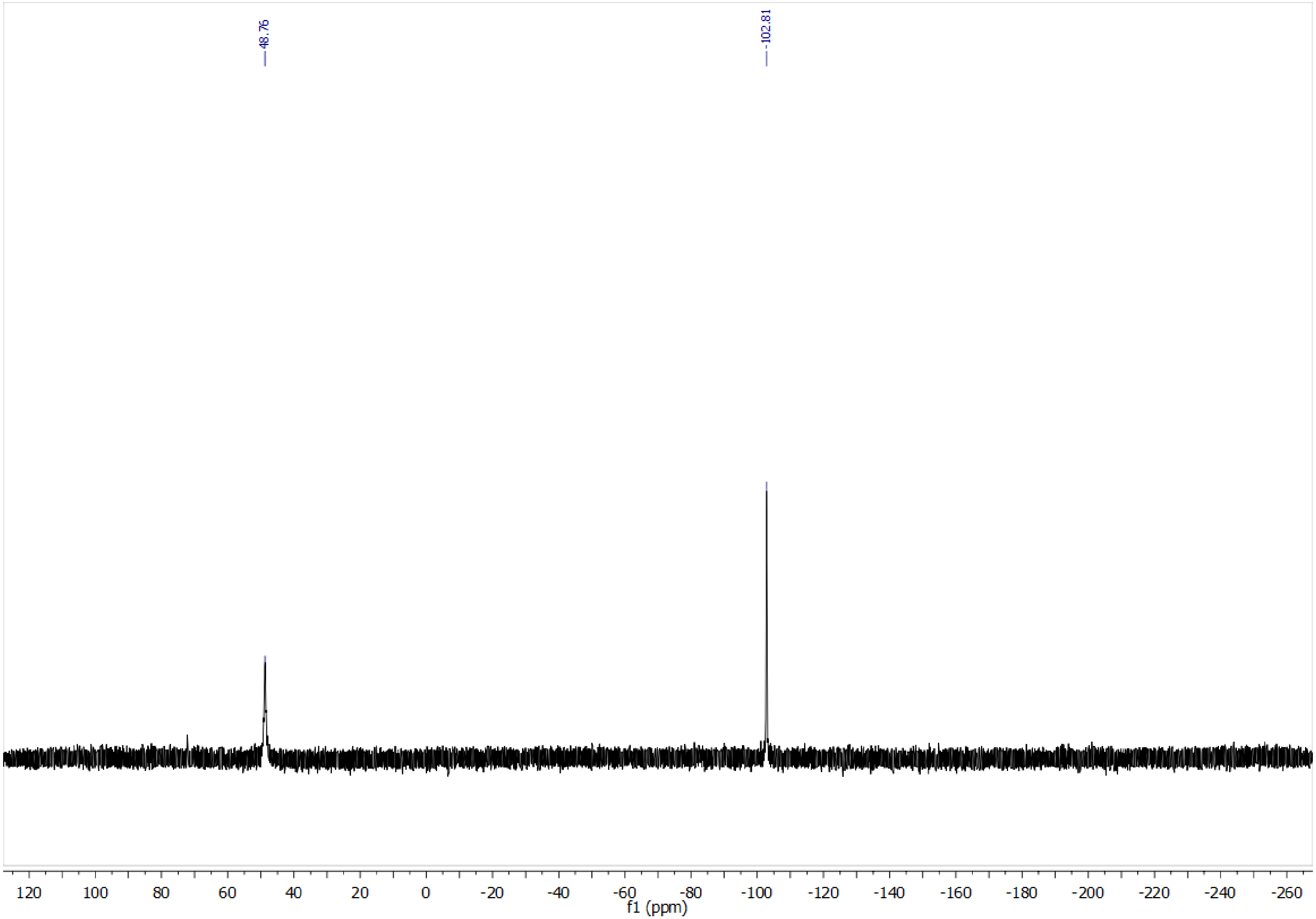

Figure S38. *^{31}P NMR spectrum of the Gutmann-Beckett test for* ***1***$^{+}$.

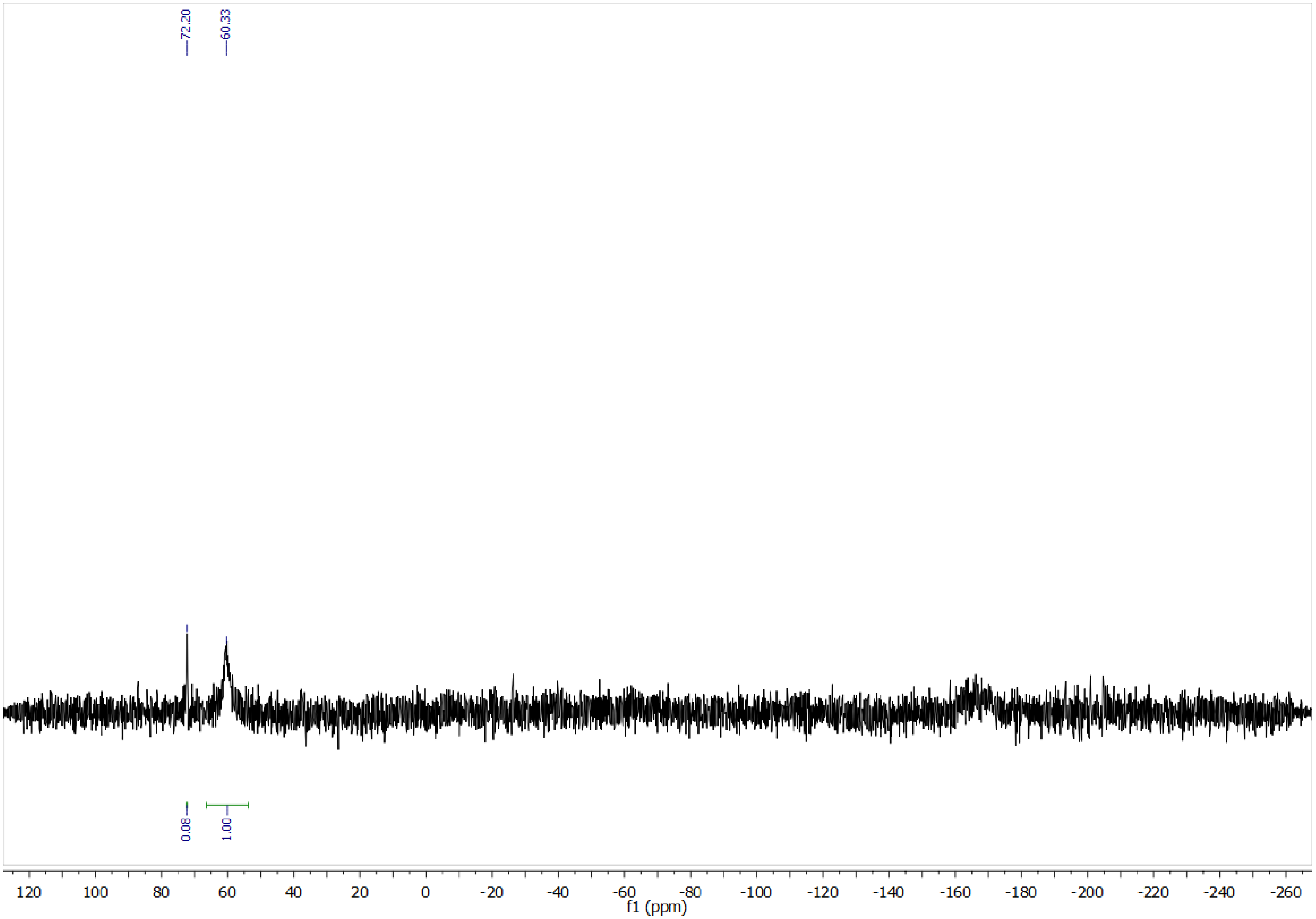

Figure S39. *^{31}P NMR spectrum of the Gutmann-Beckett test for **2^+**. The minor resonance at δ 72.2 ppm is attributed to a slow reaction of (n-octyl)$_3$PO with B(C_6F_5)$_4^-$.*

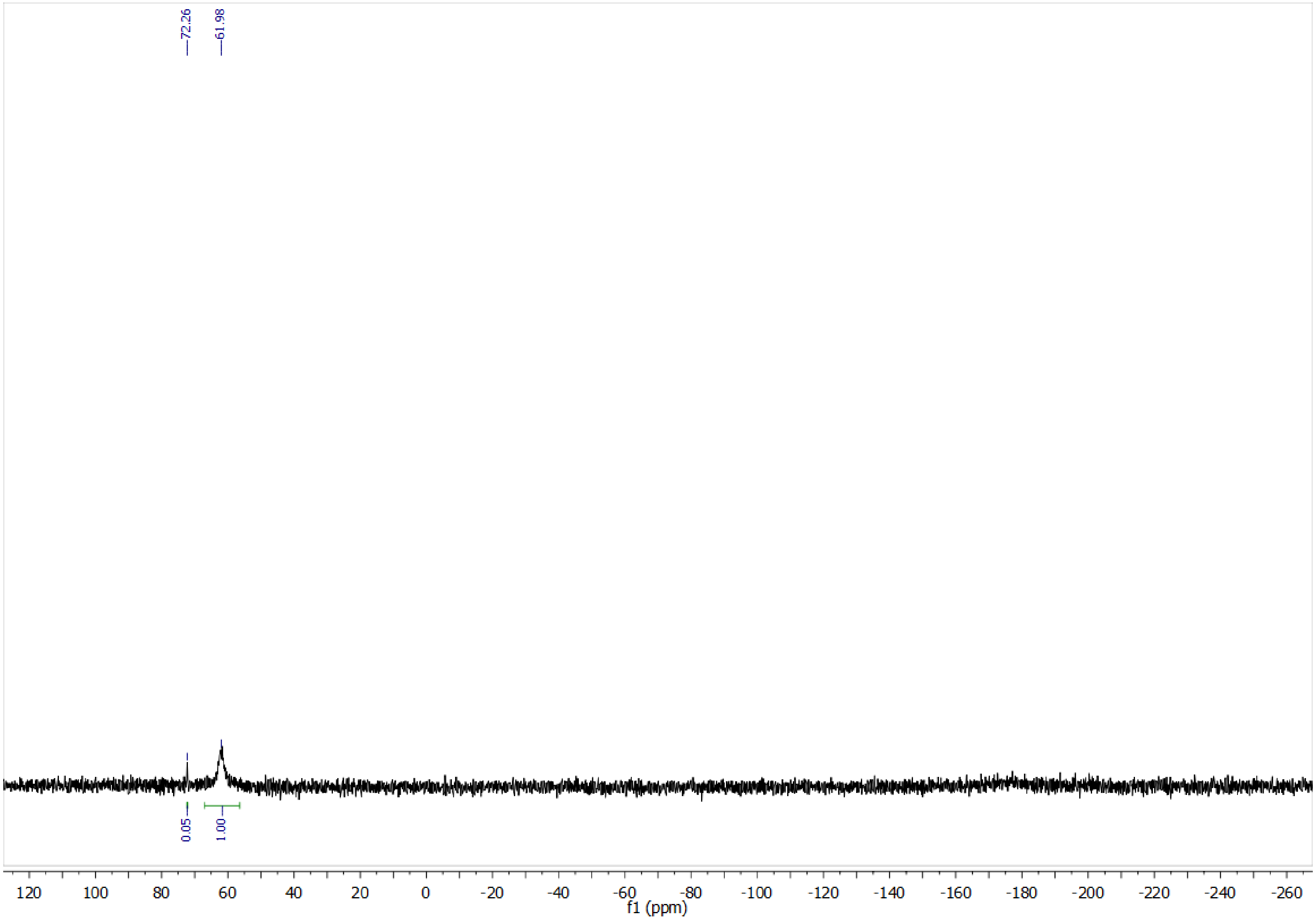

Figure S40. *^{31}P NMR spectrum of the Gutmann-Beckett test for **3^{+}**. The minor resonance at δ 72.2 ppm is attributed to a slow reaction of Oct_3PO with $B(C_6F_5)_4^-$.*

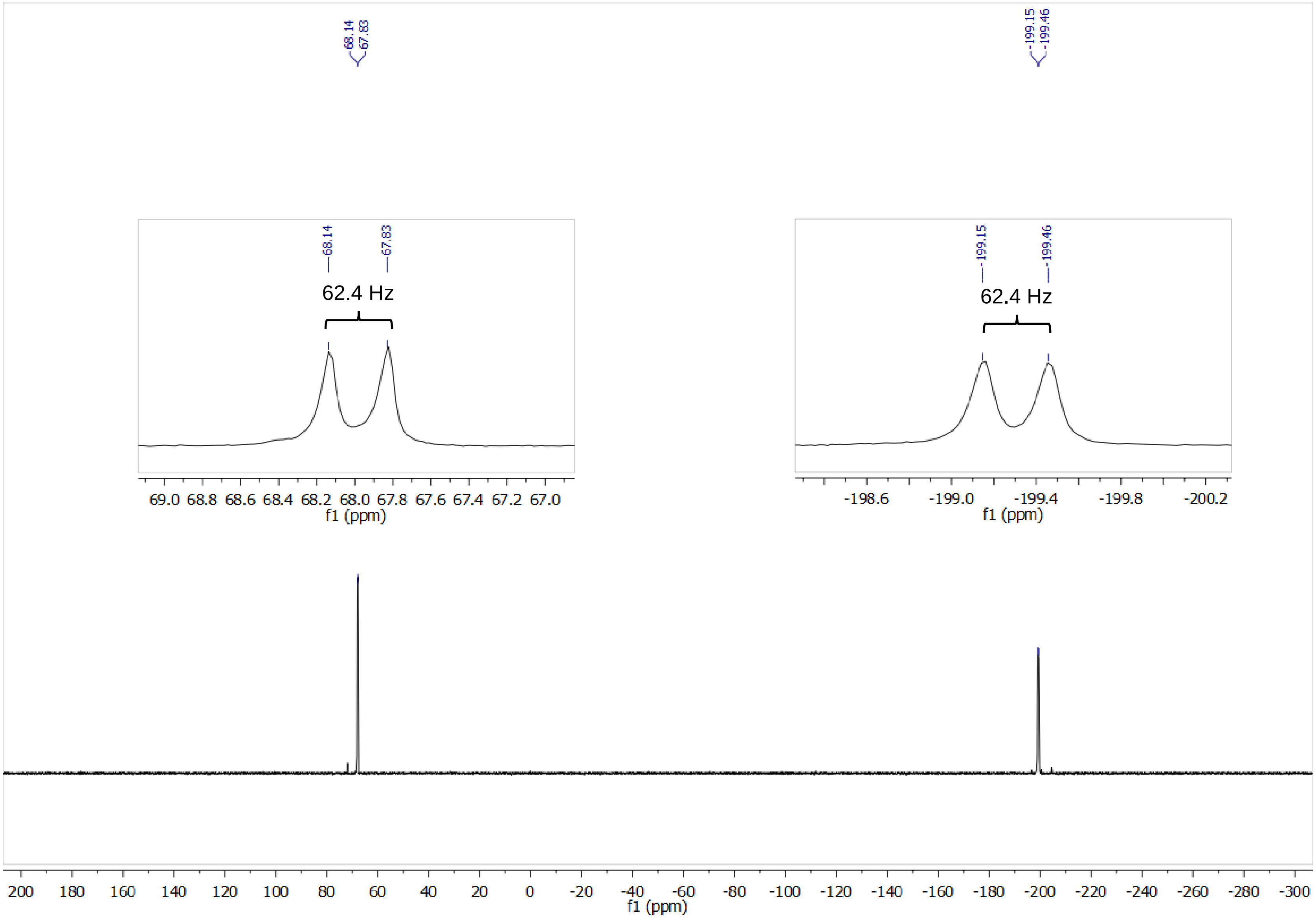

Figure S41. *^{31}P NMR spectrum of the Gutmann-Beckett test for* ***4***$^{+}$. *$^{2}J_{P-P}$ coupling is observed between the two P centers.*

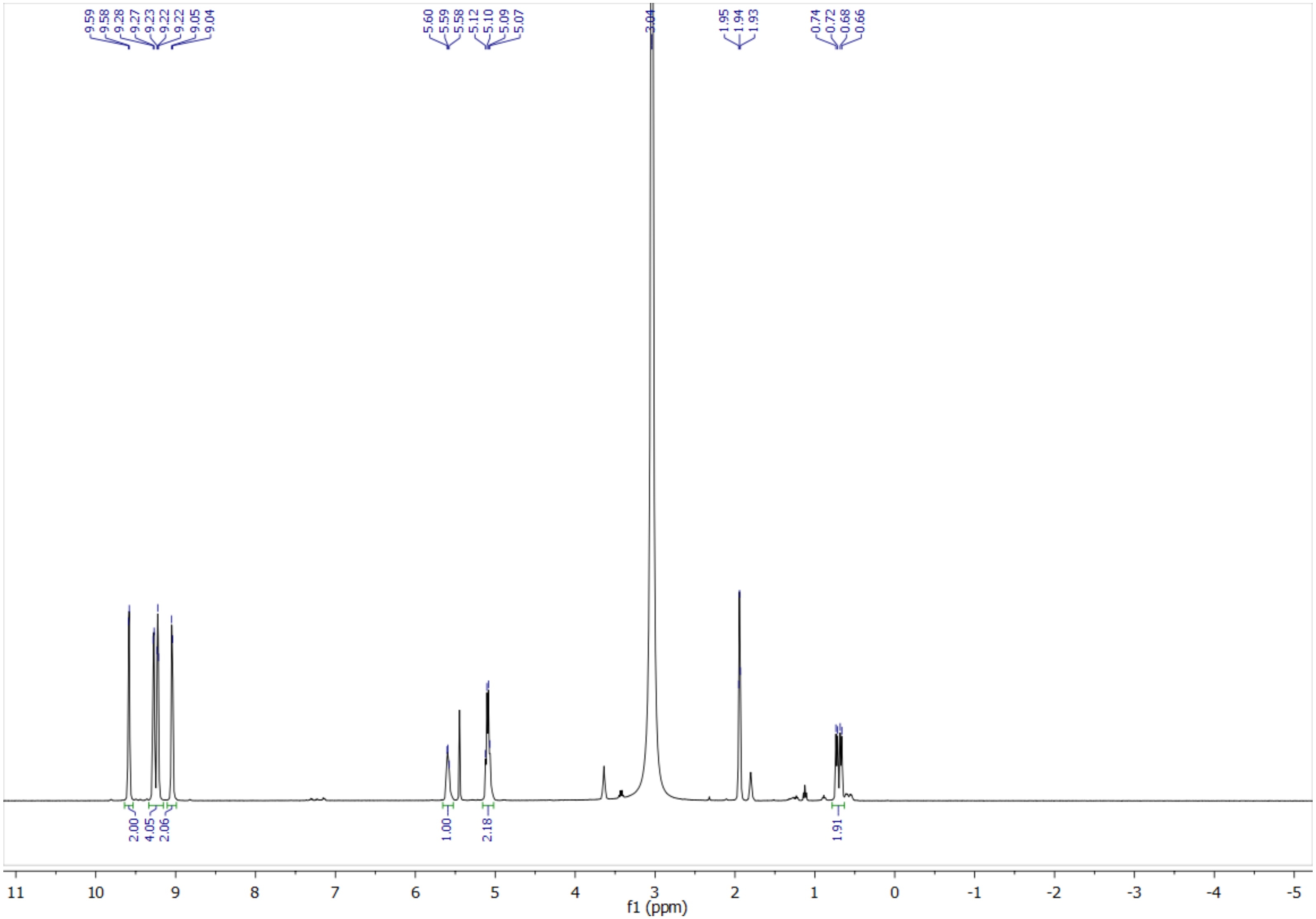

Figure S42. *^{1}H NMR spectrum of **3⁺** in CD_3CN with 10 equiv H_2O.*

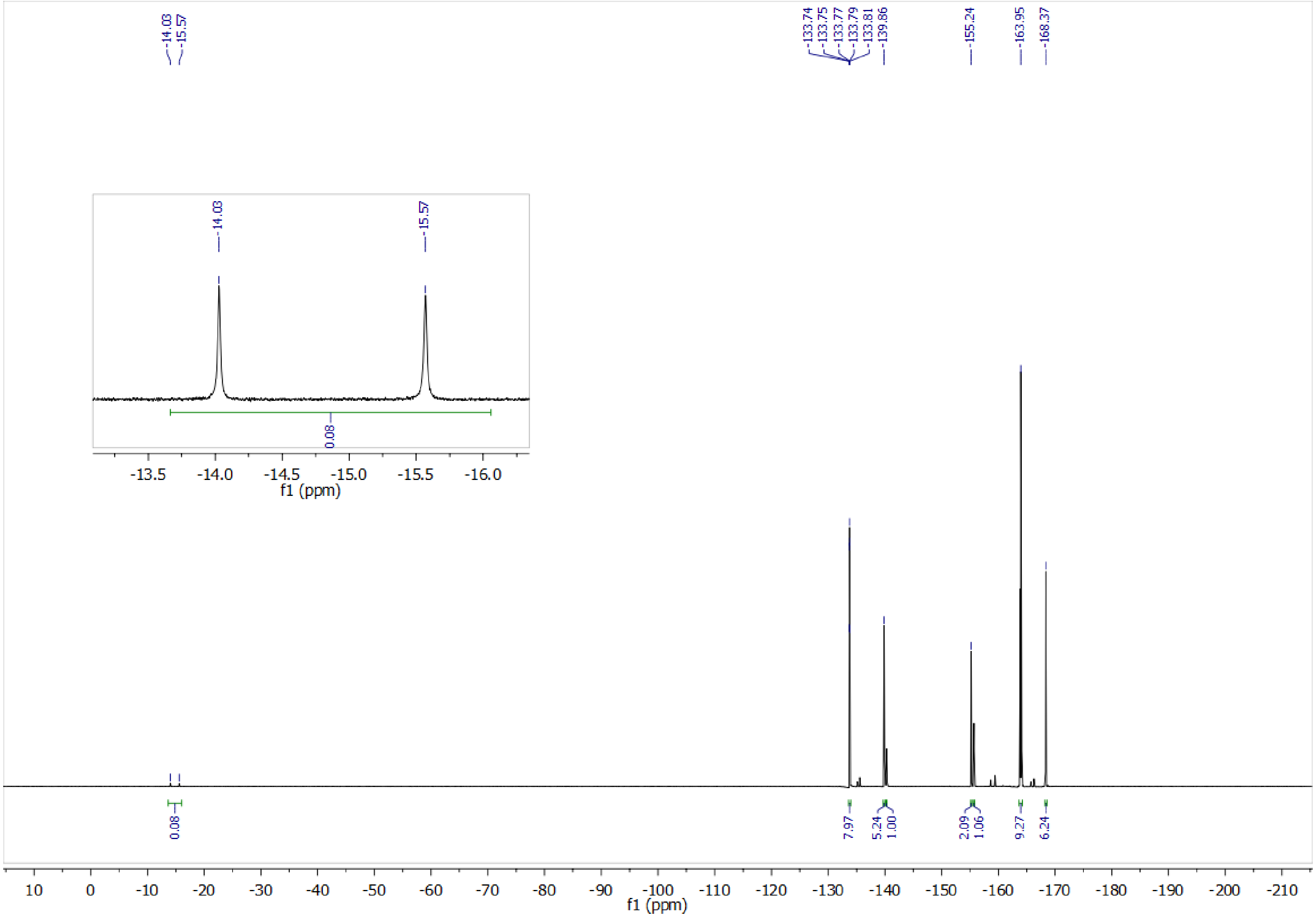

Figure S43. *^{19}F NMR spectrum of **3^+** in CD_3CN with 10 equiv H_2O.*

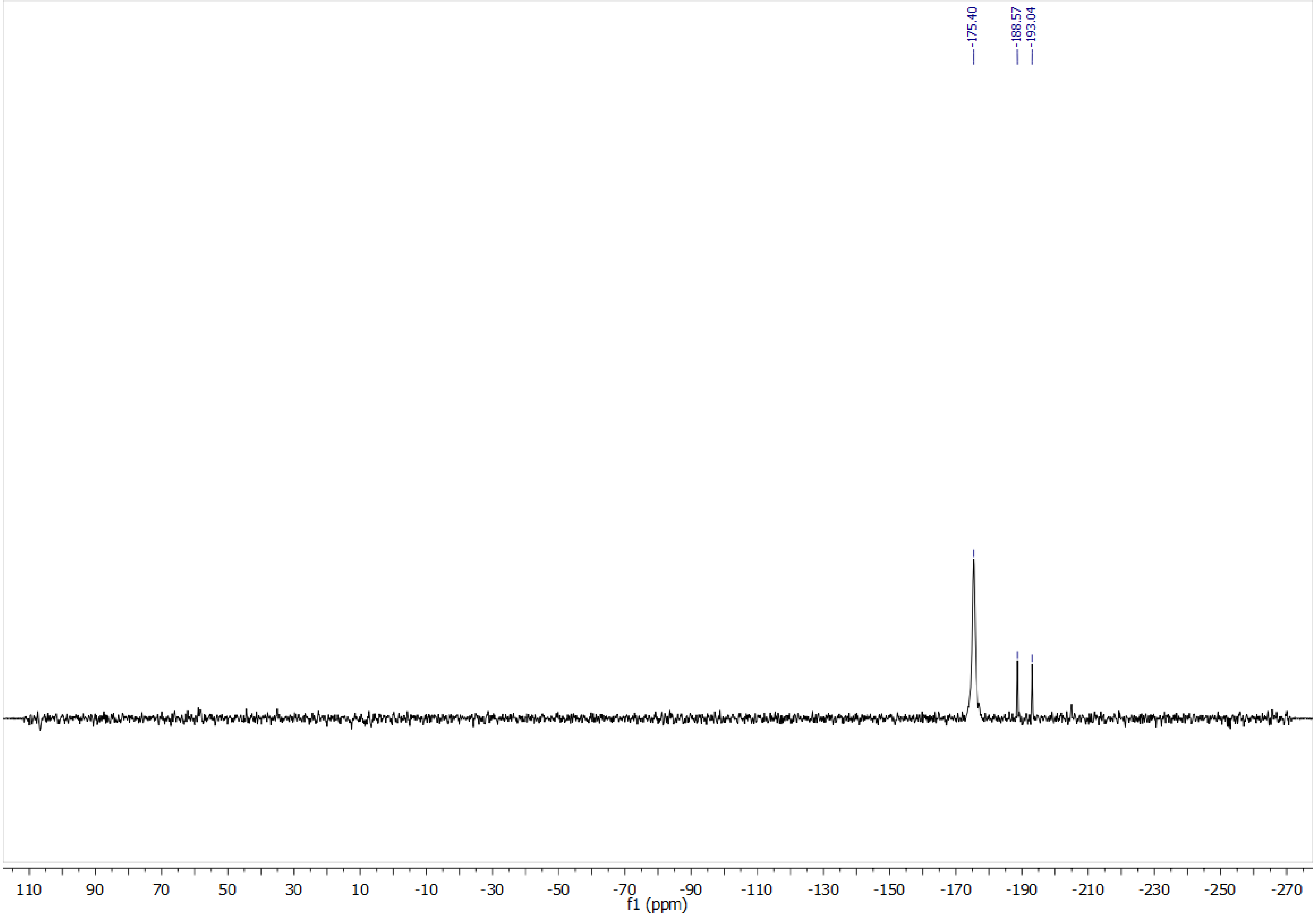

Figure S44. *$^{31}P\{^1H\}$ NMR spectrum of **3^+** in CD_3CN with 10 equiv H_2O.*

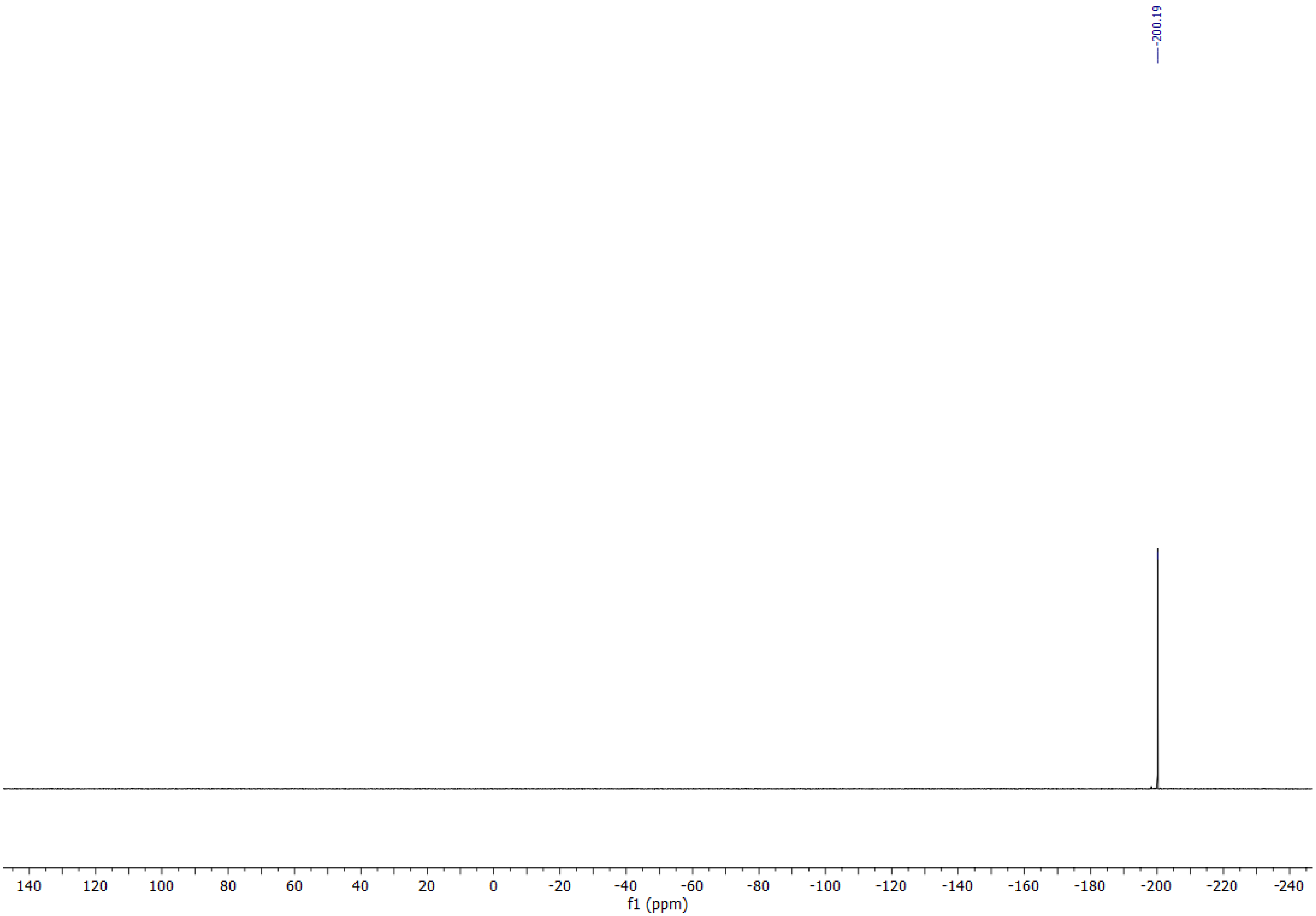

Figure S45. *$^{31}P\{^{1}H\}$ NMR spectrum of **3⁺** with water and magnesium sulfate.*

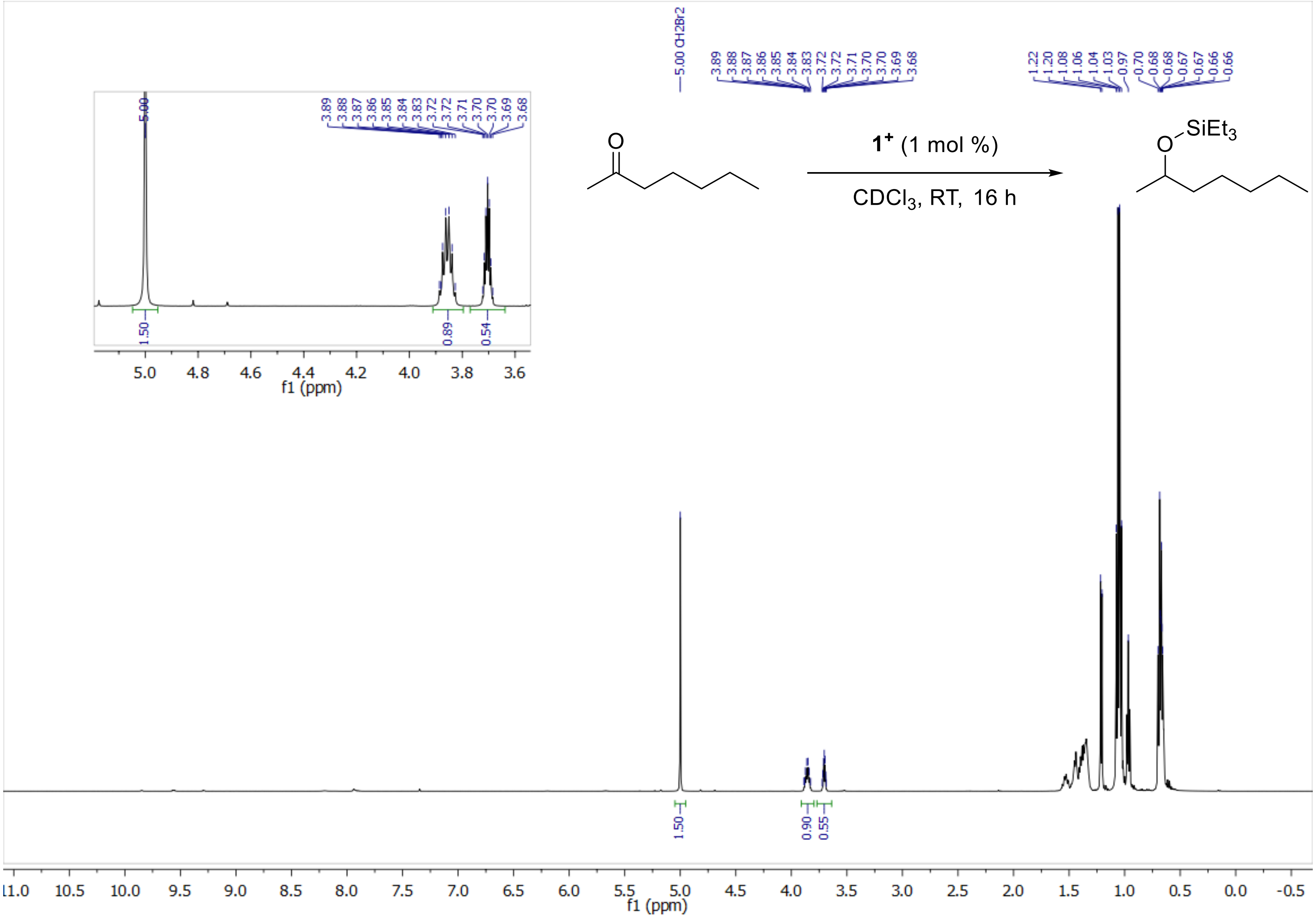

Figure S46. *^{1}H NMR spectrum of the 2-heptanone hydrosilylation reaction mixture. Excess $HSiEt_3$ is visible at δ 3.70.*

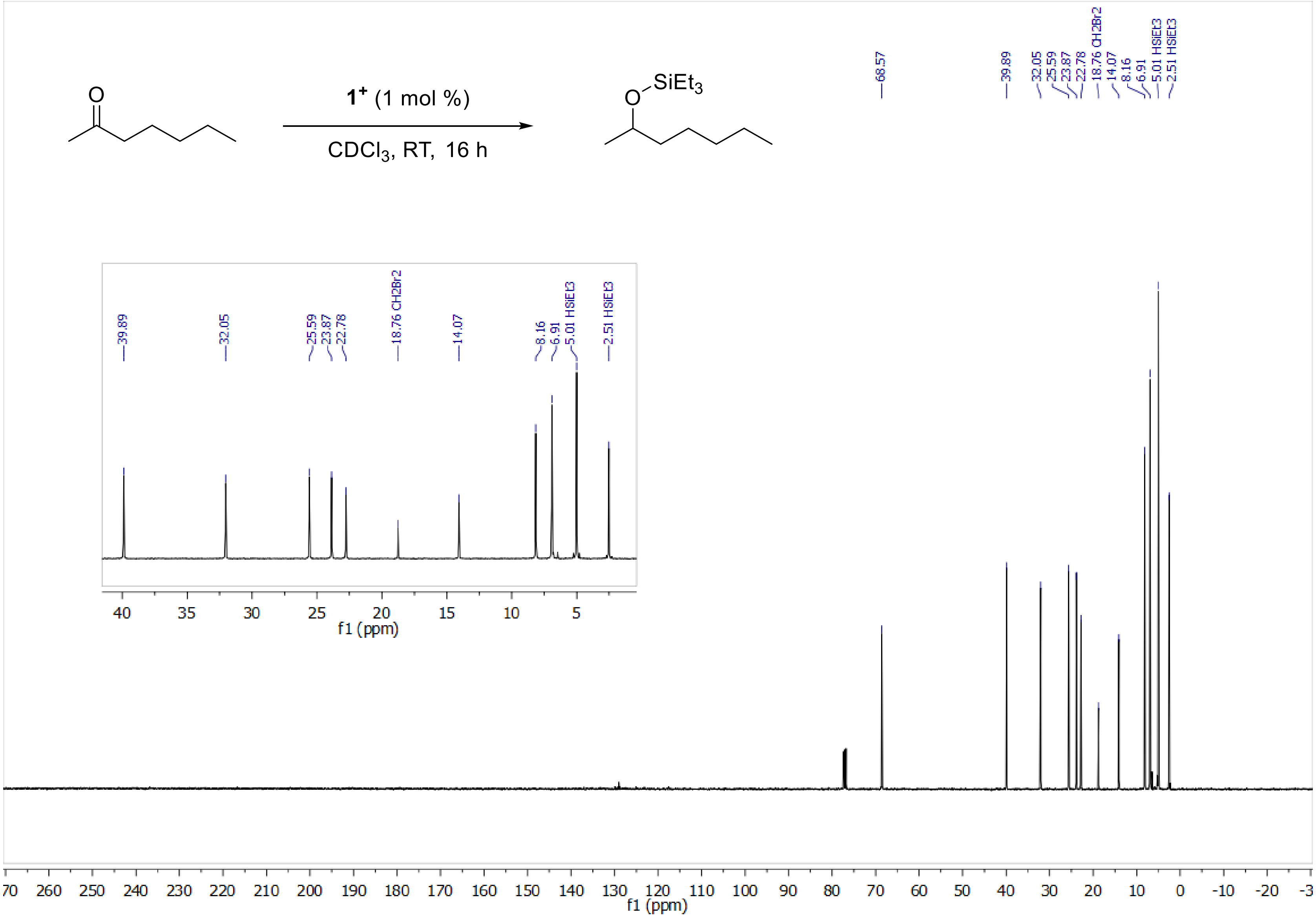

Figure S47. *$^{13}C\{^1H\}$ NMR spectrum of the 2-heptanone hydrosilylation reaction mixture.*

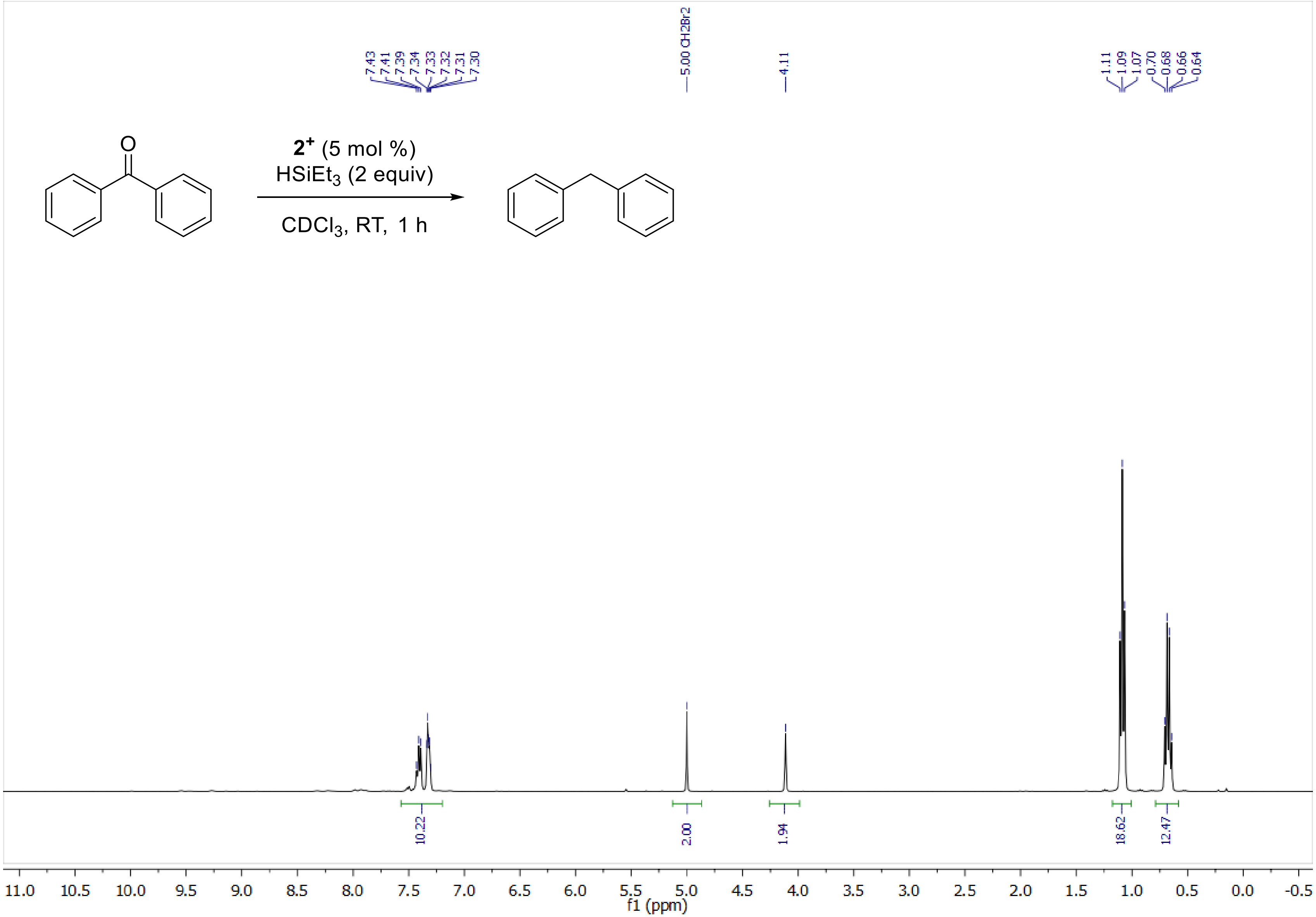

Figure S48. *^{1}H NMR spectrum of the benzophenone deoxygenation reaction mixture.*

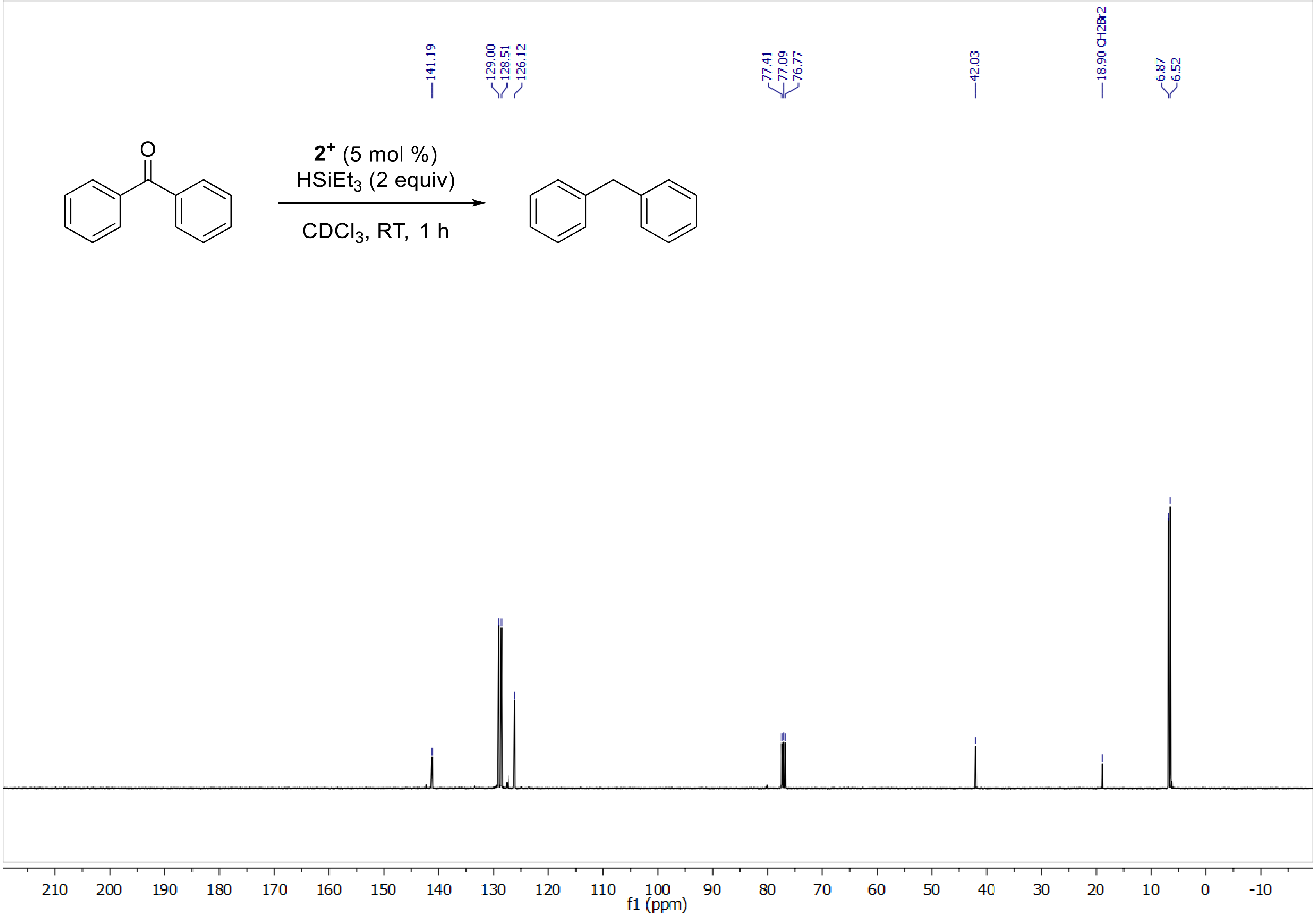

Figure S49. *$^{13}C\{^1H\}$ NMR spectrum of the benzophenone deoxygenation reaction mixture.*

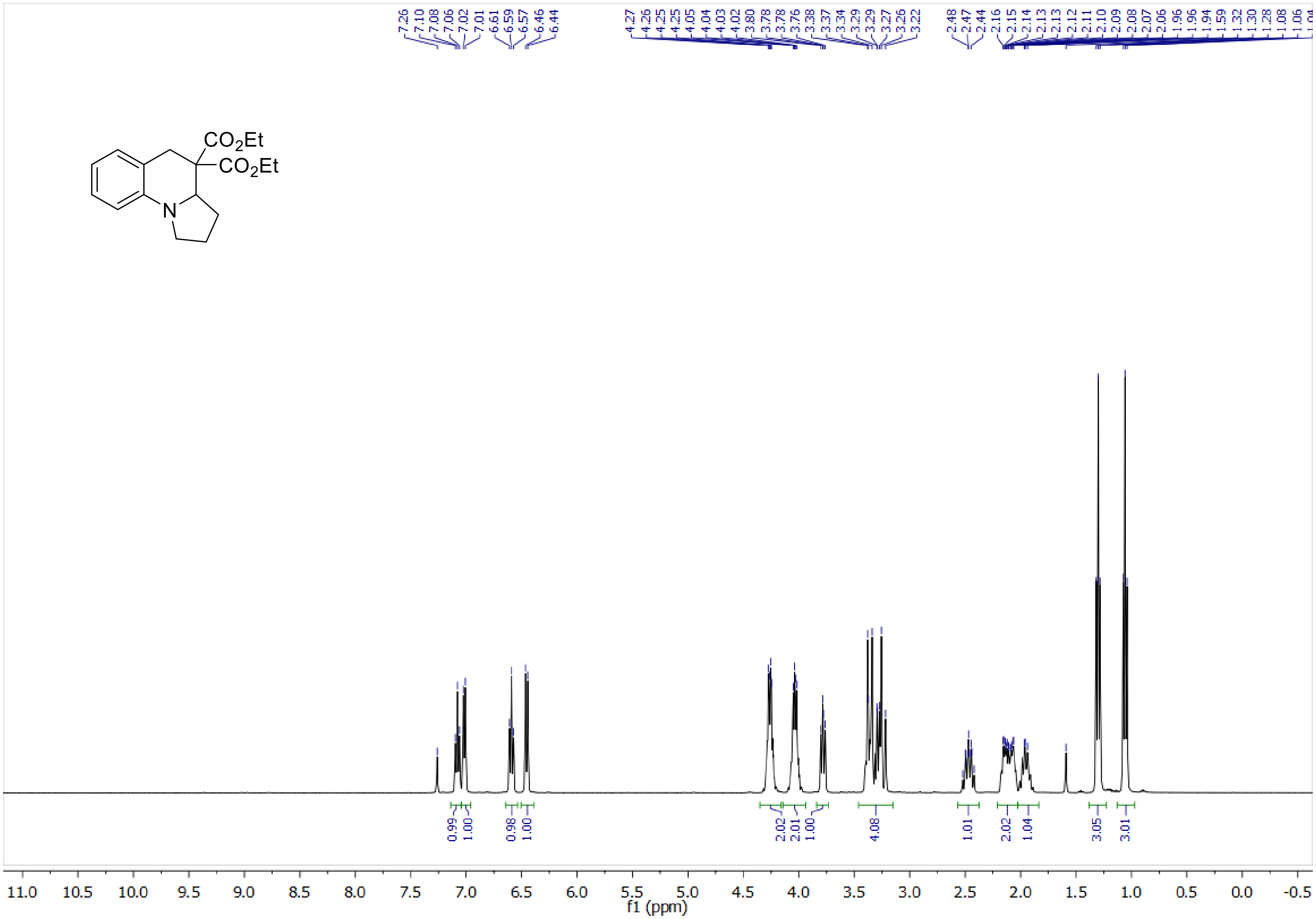

Figure S50. *^{1}H NMR spectrum of diethyl 1,2,3,3a-tetrahydropyrrolo[1,2-a]quinoline-4,4(5H)-dicarboxylate.*

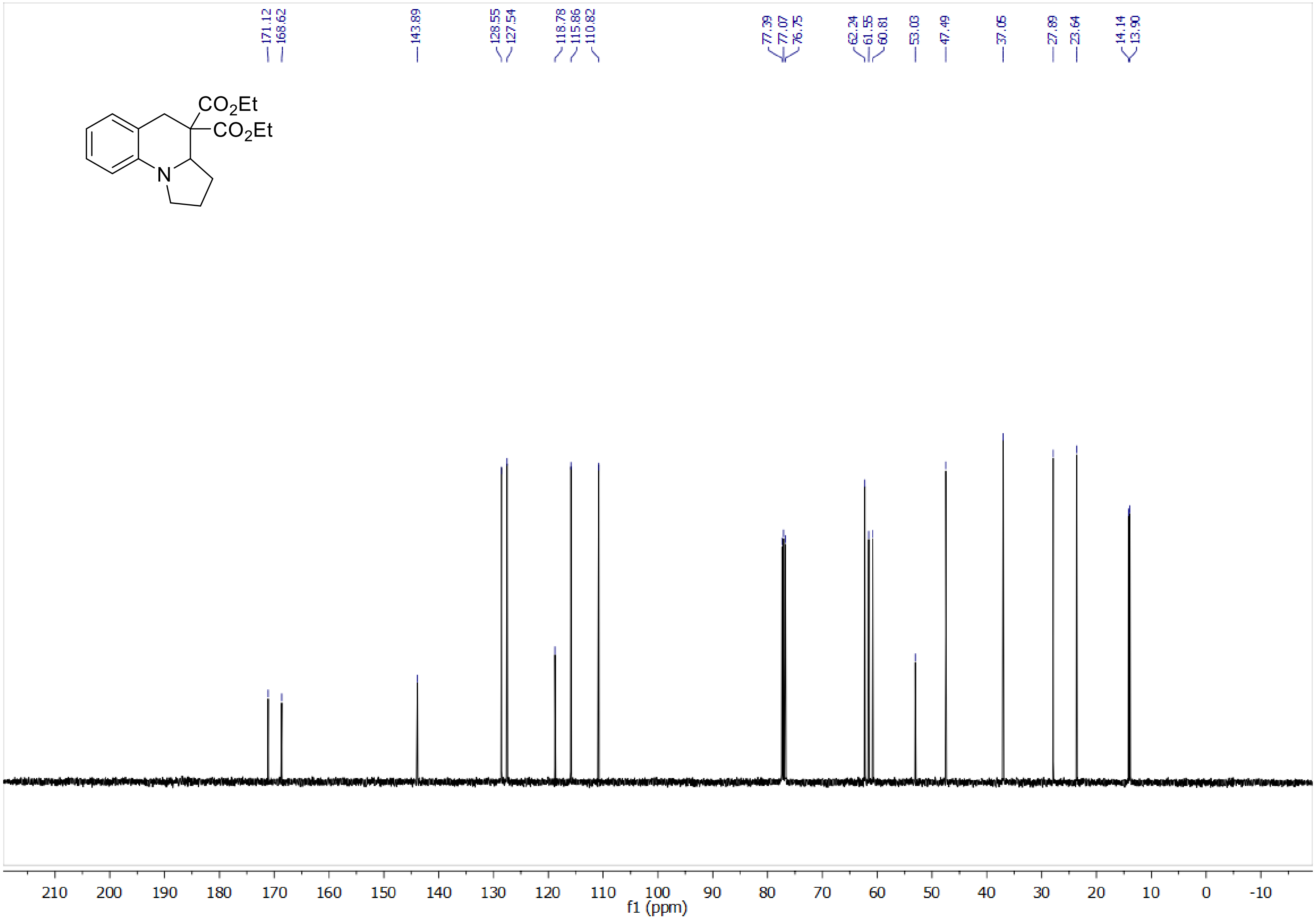

Figure S51. *$^{13}C\{^{1}H\}$ NMR spectrum of diethyl 1,2,3,3a-tetrahydropyrrolo[1,2-a]quinoline-4,4(5H)-dicarboxylate.*

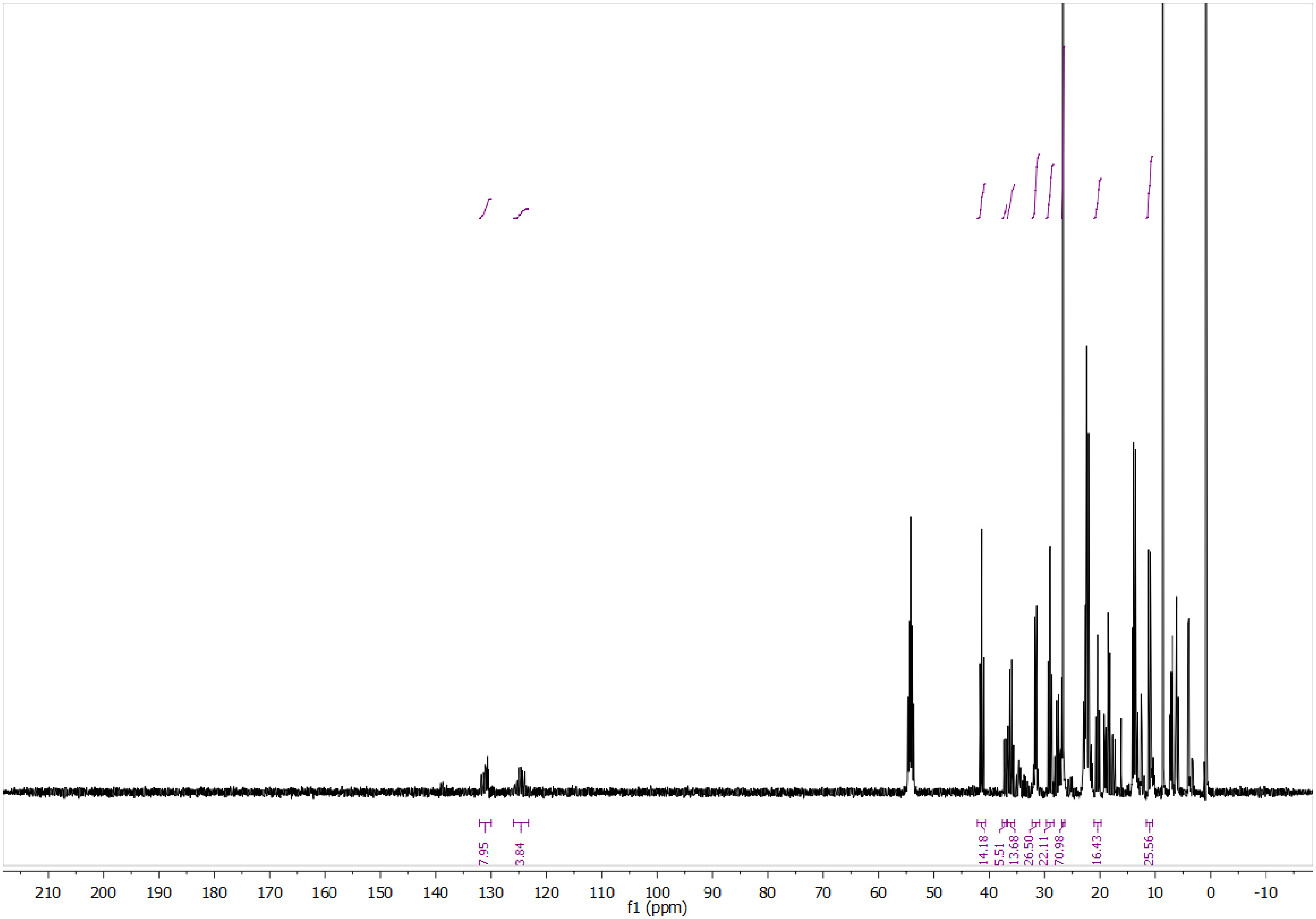

Figure S52. *Full $^{13}C\{^{1}H\}$ NMR spectrum of the $^{13}C_6$-D-glucose deoxygenation reaction mixture. Note that C–C coupling is observed.*

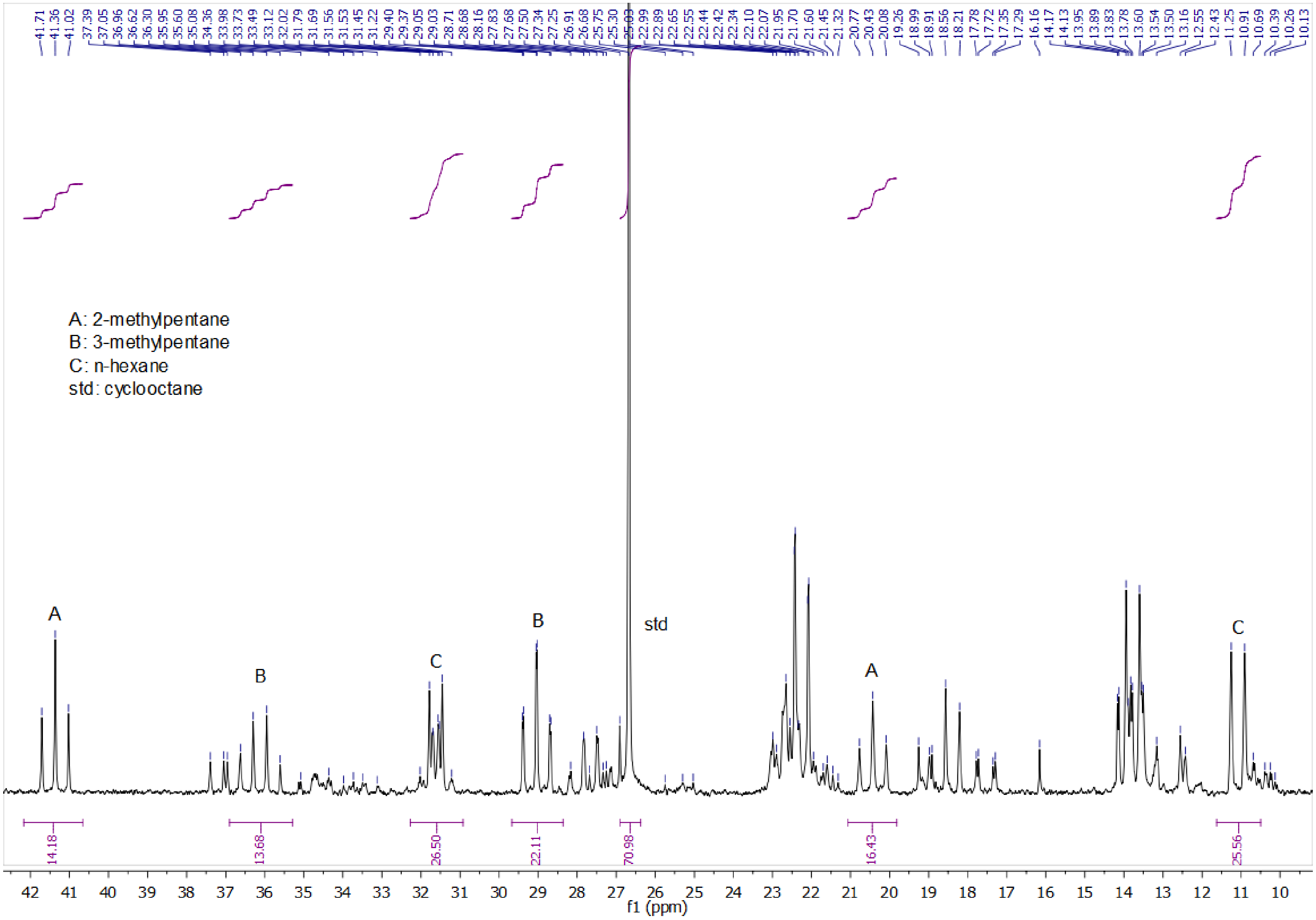

Figure S53. *Aliphatic region of the above $^{13}C\{^{1}H\}$ NMR spectrum of the $^{13}C_6$-D-glucose deoxygenation reaction. Note that C–C coupling is observed.*

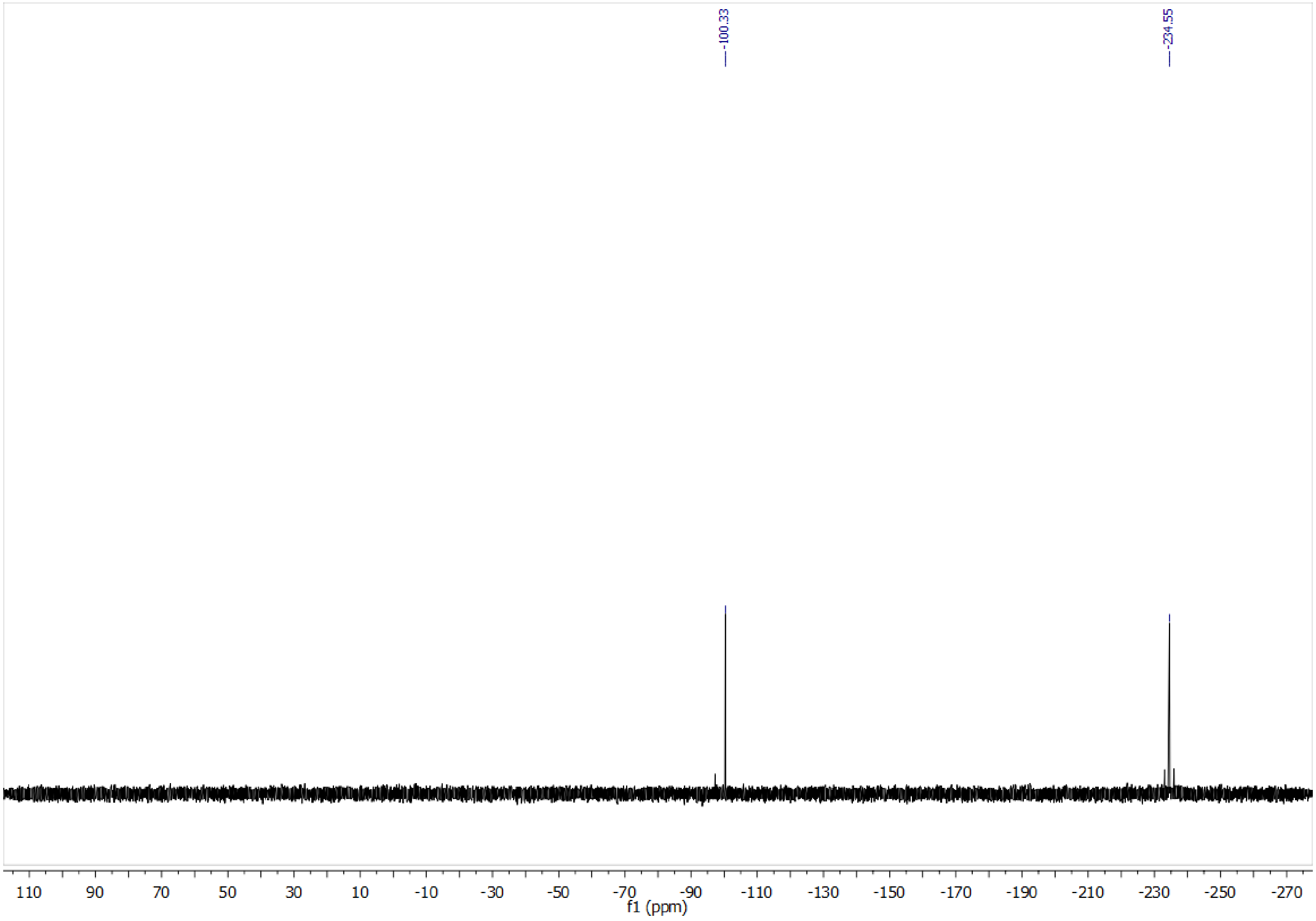

Figure S54. *$^{31}P\{^{1}H\}$ NMR spectrum of the glucose deoxygenation reaction after 1 d.*

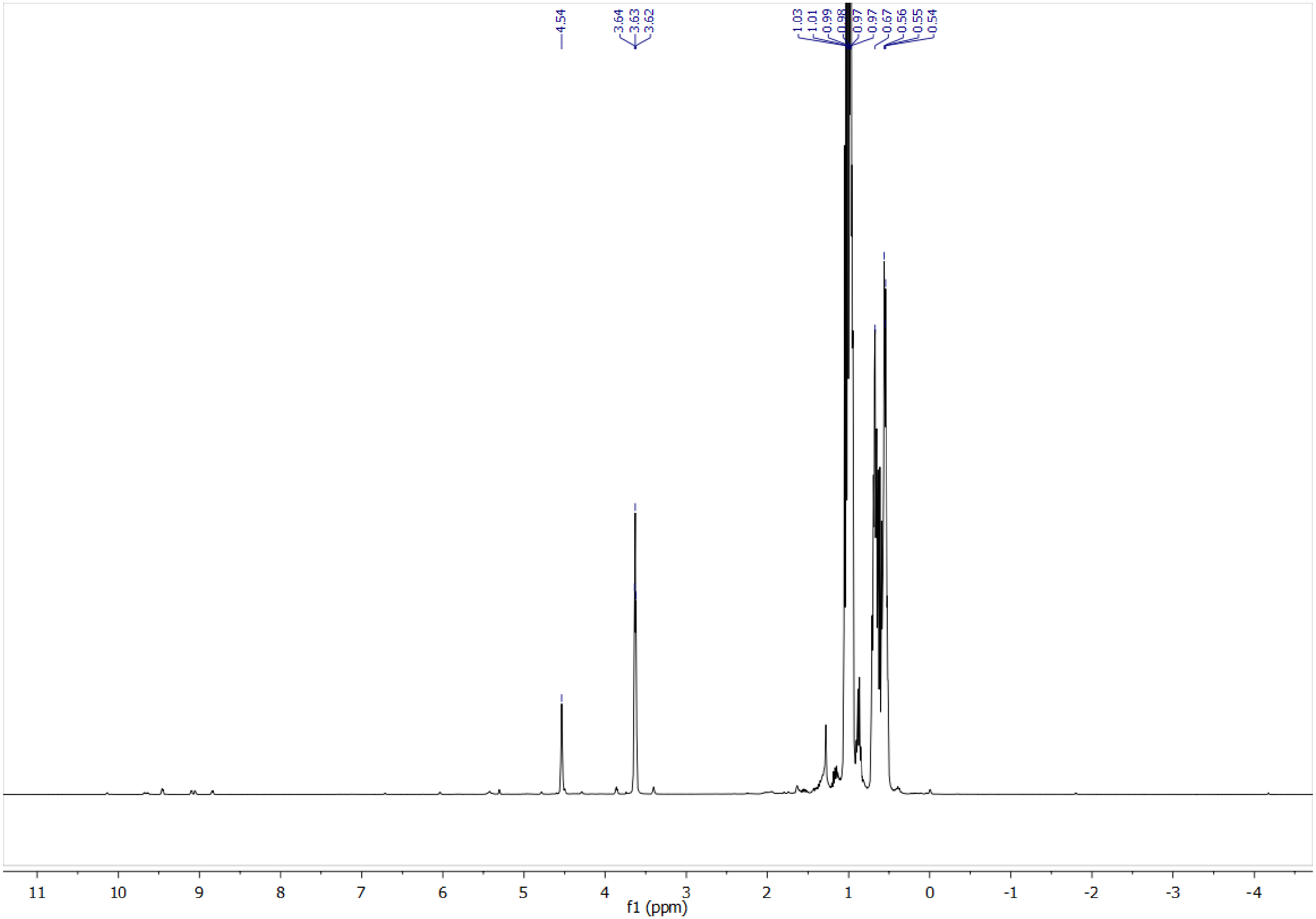

Figure S55. *^{1}H NMR spectrum of the glucose deoxygenation reaction after 1 d.*

8. UV-VIS spectra

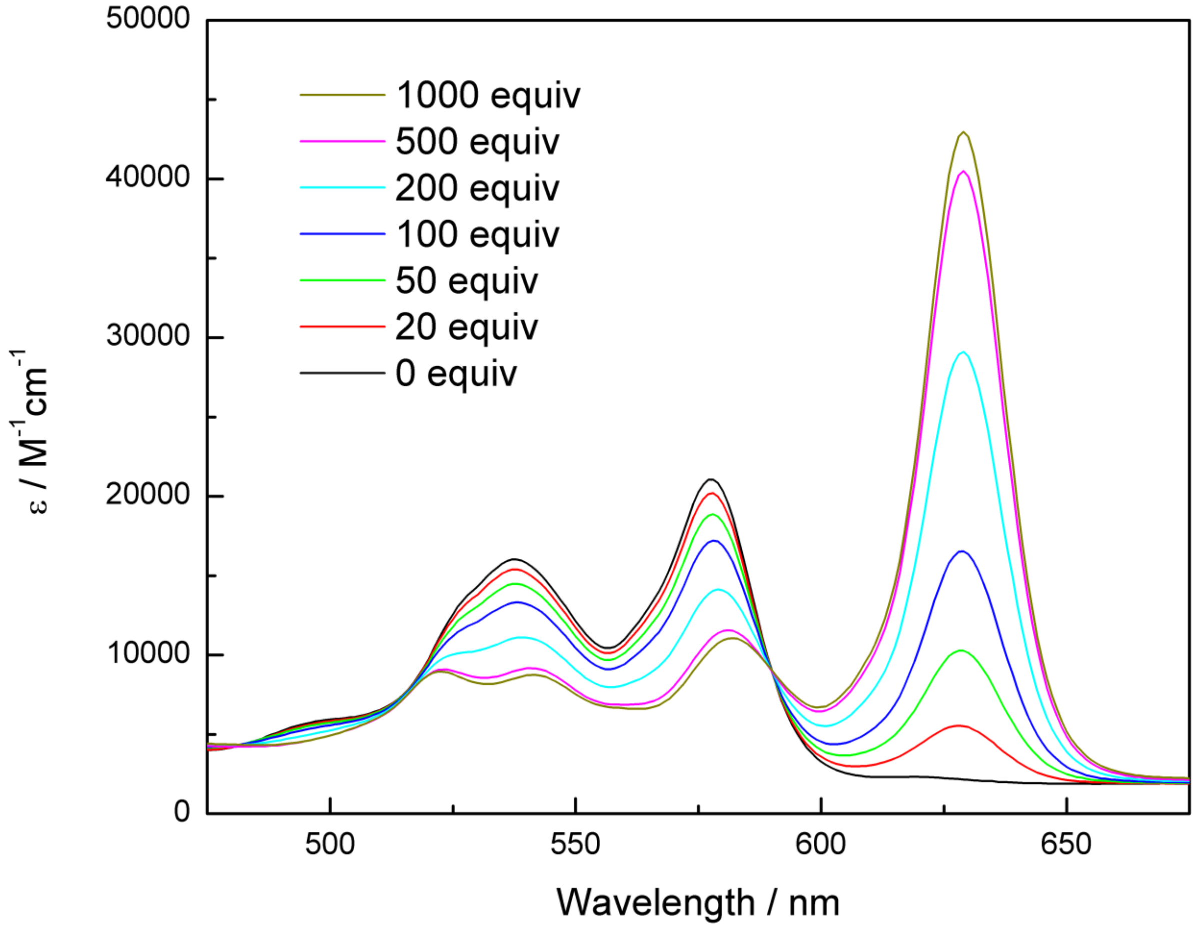

Figure S56. *Q-band region of* ***1***$^{+}$ *with varying equivalences of (*n*-octyl)*$_3$*P=O.*

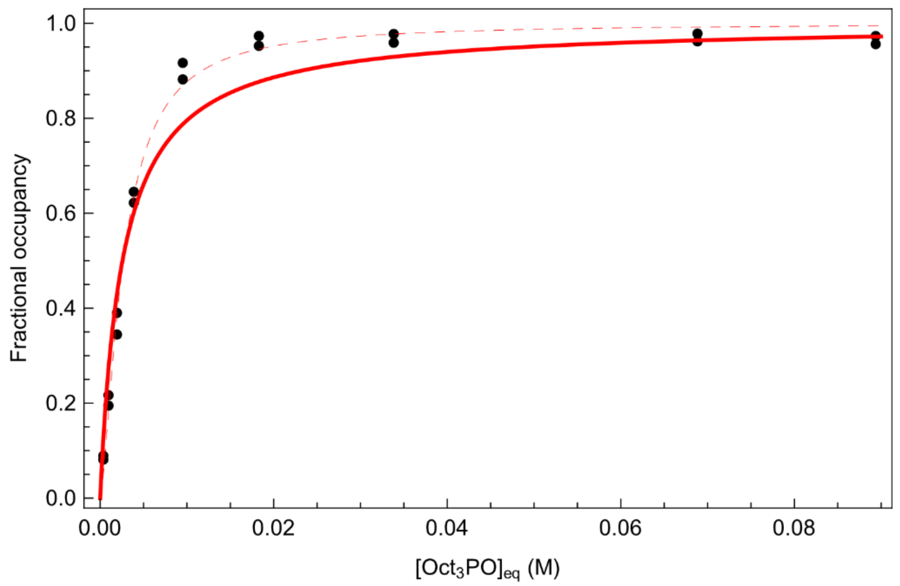

Figure S57. *Binding curves (solid: Michaelis-Menten; dashed: Hill) for titration of* ***1⁺*** *with* (n-*octyl)₃P=O.*

Michaelis-Menten equation fit parameters:

$$Fractional\ occupancy = \frac{[Oct_3PO]_{eq}}{K_d + [Oct_3PO]_{eq}}$$

Parameter	Estimate	Standard error	t-statistic	p-value
K_d	0.00256969	0.00025298	10.1577	1.23044e-8

$R^2 = 0.993$

Hill equation fit parameters:

$$Fractional\ occupancy = \frac{{[Oct_3PO]_{eq}}^n}{{K_d}^n + {[Oct_3PO]_{eq}}^n}$$

Parameter	Estimate	Standard error	t-statistic	p-value
K_d	0.00265617	0.0000877992	30.2528	1.50296e-15
n	1.48117	0.0670867	22.0784	2.07298e-13

$R^2 = 0.998$

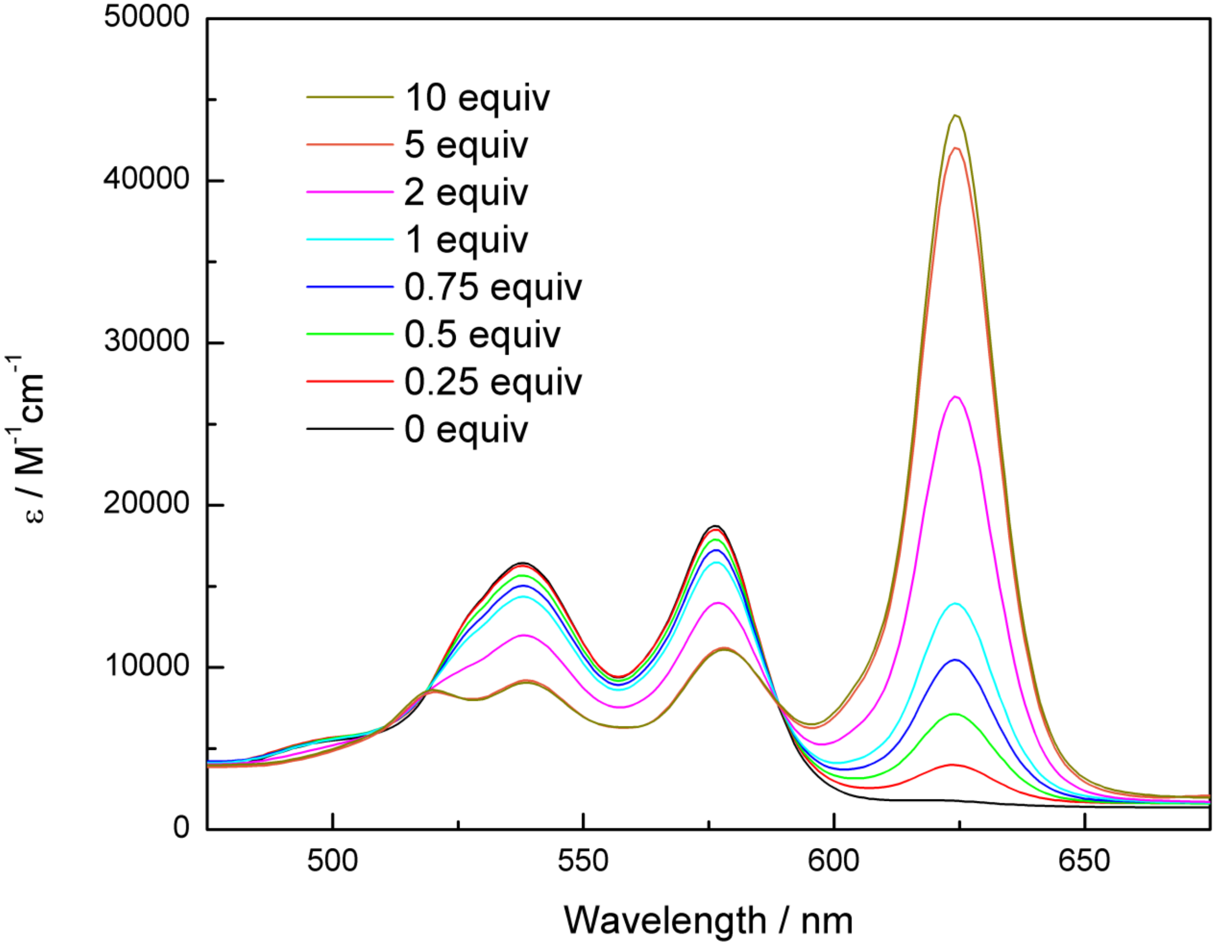

Figure S58. Q-band region of **2**$^+$ with varying equivalences of (*n*-octyl)$_3$P=O.

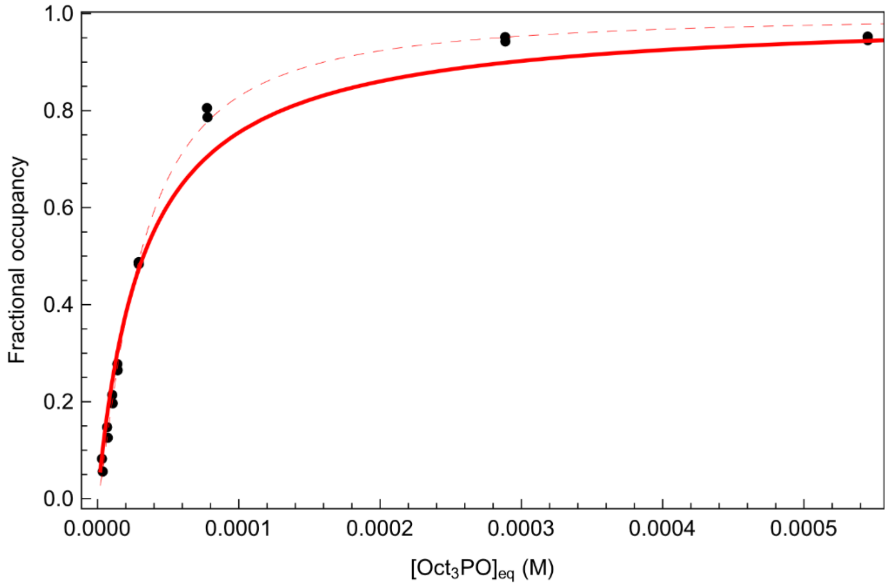

Figure S59. *Binding curves (solid: Michaelis-Menten; dashed: Hill) for titration of* ***2***$^{+}$ *with (*n-*octyl)*$_3$*P=O.*

Michaelis-Menten equation fit parameters:

$$Fractional\ occupancy = \frac{[Oct_3PO]_{eq}}{K_d + [Oct_3PO]_{eq}}$$

Parameter	Estimate	Standard error	t-statistic	p-value
K_d	0.0000324852	2.34497e-6	13.8531	5.93441e-10

R^2 = 0.993

Hill equation fit parameters:

$$Fractional\ occupancy = \frac{[Oct_3PO]_{eq}^{\ n}}{K_d^{\ n} + [Oct_3PO]_{eq}^{\ n}}$$

Parameter	Estimate	Standard error	t-statistic	p-value
K_d	0.000030052	7.43199e-5	40.4360	6.68284e-16
n	1.31334	0.0376445	34.8878	5.17596e-15

R^2 = 0.999

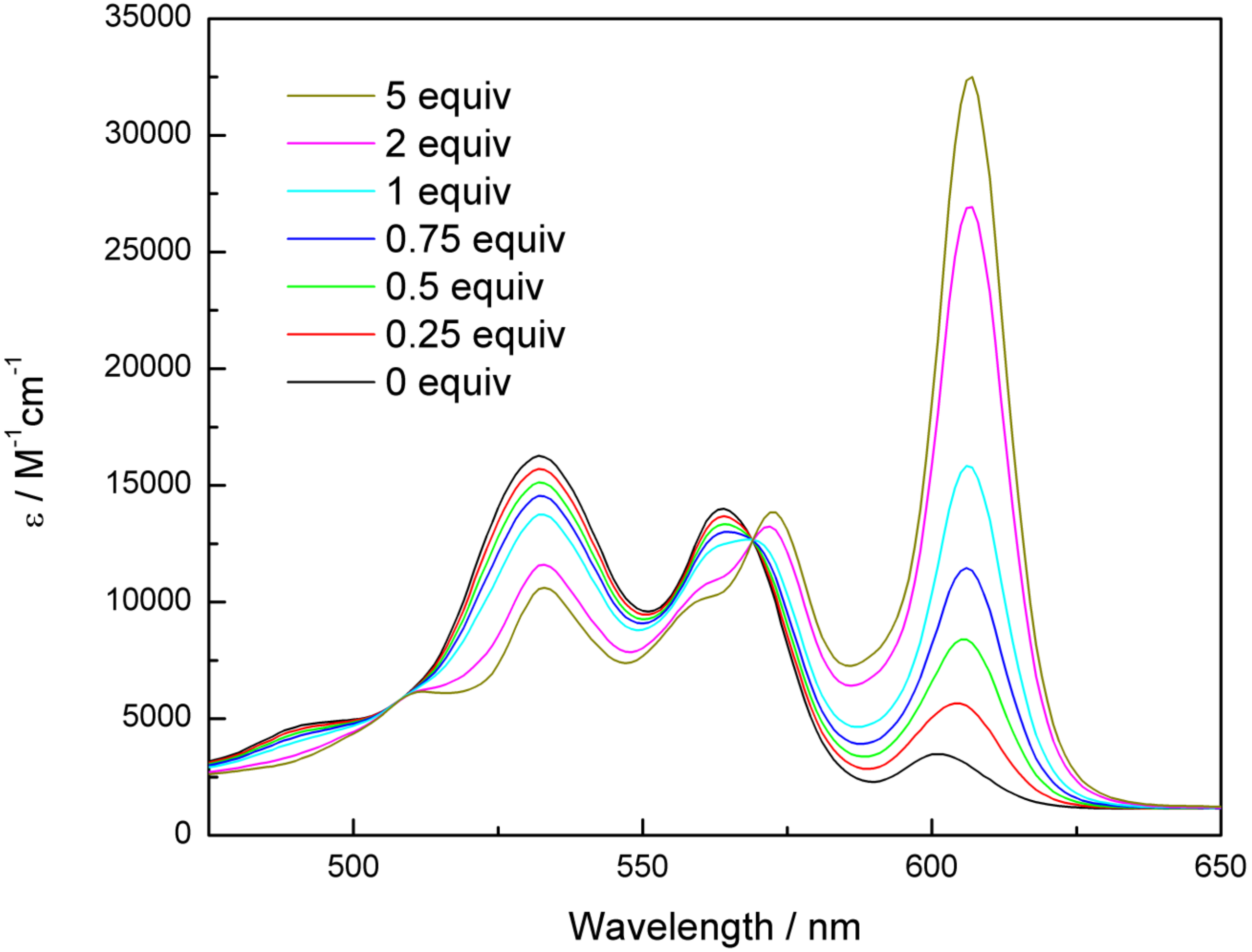

Figure S60. Q-band region of **3**$^{+}$ with varying equivalences of (*n*-octyl)$_3$P=O.

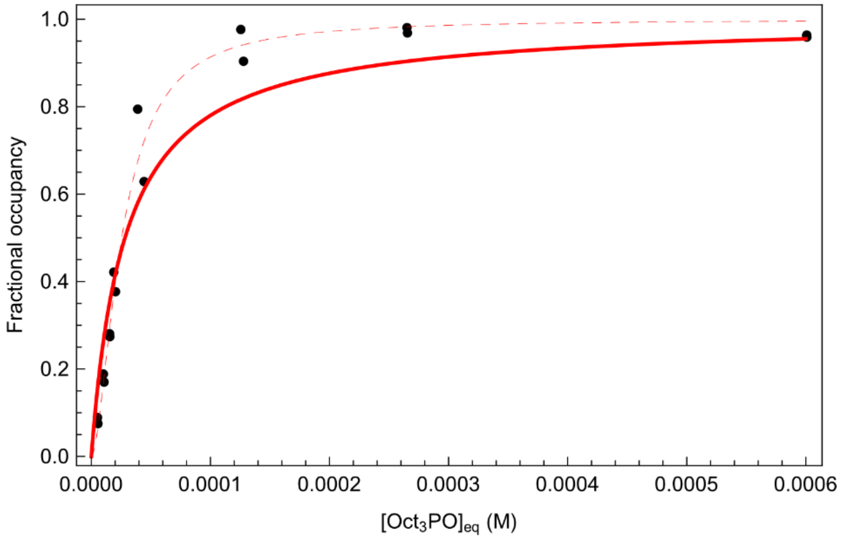

Figure S61. *Binding curves (solid: Michaelis-Menten; dashed: Hill) for titration of* ***3***$^{+}$ *with (*n-*octyl)*$_3$*P=O.*

Michaelis-Menten equation fit parameters:

$$Fractional\ occupancy = \frac{[Oct_3PO]_{eq}}{K_d + [Oct_3PO]_{eq}}$$

Parameter	Estimate	Standard error	t-statistic	p-value
K_d	0.0000282225	3.73091e-6	7.56451	1.70374e-6

R^2 = 0.980

Hill equation fit parameters:

$$Fractional\ occupancy = \frac{{[Oct_3PO]_{eq}}^{n}}{{K_d}^{n} + {[Oct_3PO]_{eq}}^{n}}$$

Parameter	Estimate	Standard error	t-statistic	p-value
K_d	0.0000255336	1.29969	19.6458	1.36988e-11
n	1.7256	0.148709	11.6039	1.43732e-8

R^2 = 0.995

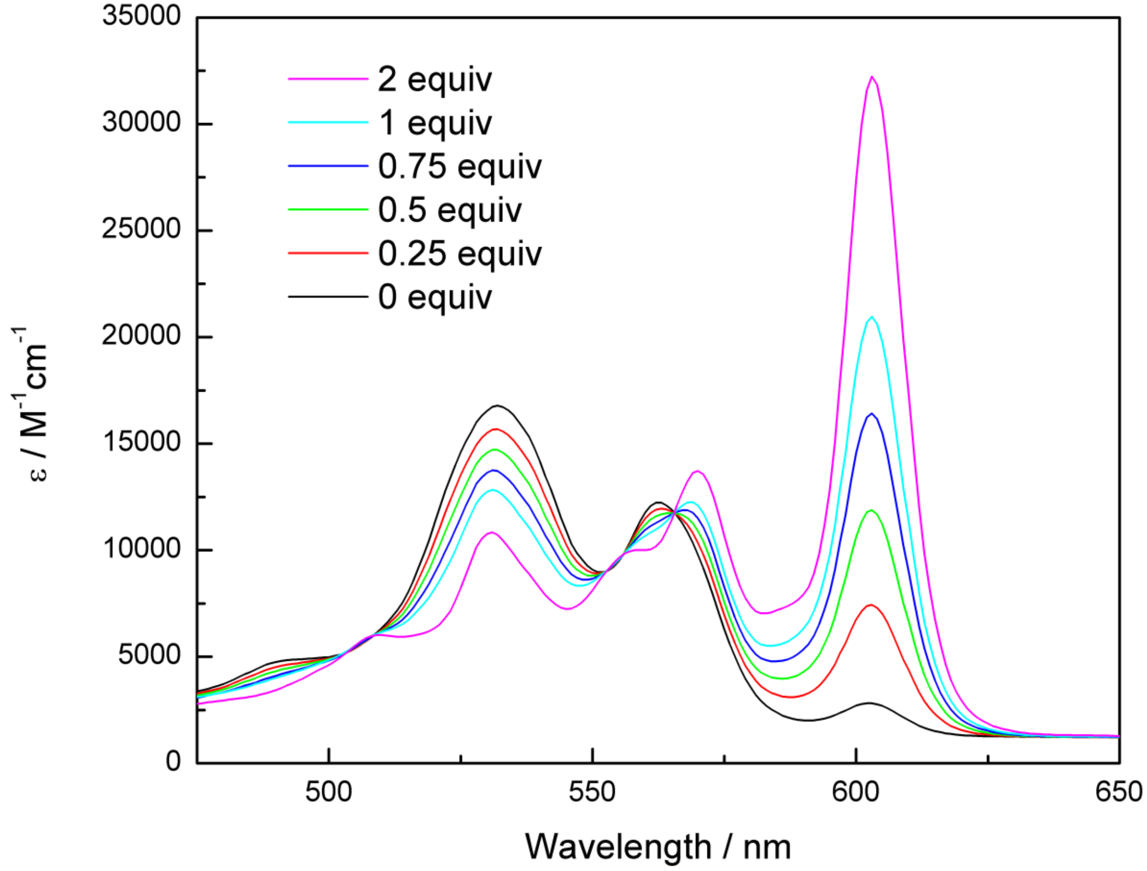

Figure S62. *Q-band region of* ***4⁺*** *with varying equivalences of (*n*-octyl)$_3$P=O.*

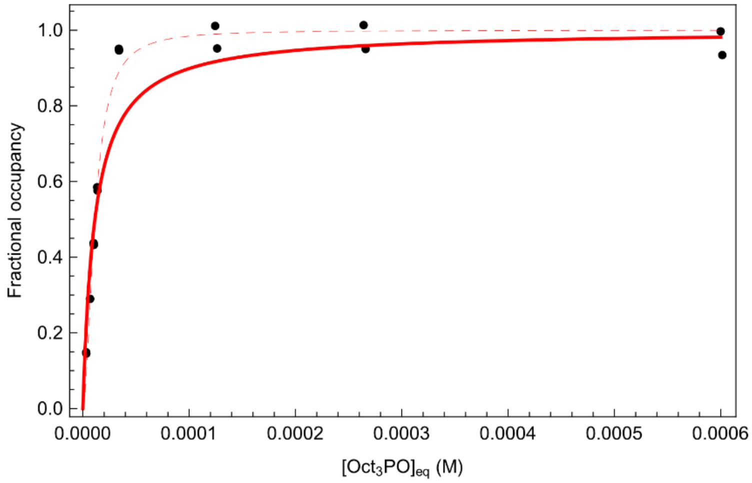

Figure S63. *Binding curves (solid: Michaelis-Menten; dashed: Hill) for titration of* ***4***$^{+}$ *with (n-octyl)*$_3$*P=O.*

Michaelis-Menten equation fit parameters:

$$Fractional\ occupancy = \frac{[Oct_3PO]_{eq}}{K_d + [Oct_3PO]_{eq}}$$

Parameter	Estimate	Standard error	t-statistic	p-value
K_d	0.0000112832	1.48123e-6	7.61742	1.56615e-6

$R^2 = 0.984$

Hill equation fit parameters:

$$Fractional\ occupancy = \frac{[Oct_3PO]_{eq}{}^{n}}{K_d{}^{n} + [Oct_3PO]_{eq}{}^{n}}$$

Parameter	Estimate	Standard error	t-statistic	p-value
K_d	0.0000112983	4.06365e-7	27.8034	1.18985e-13
n	1.8992	0.154184	12.3177	6.68298e-9

$R^2 = 0.997$

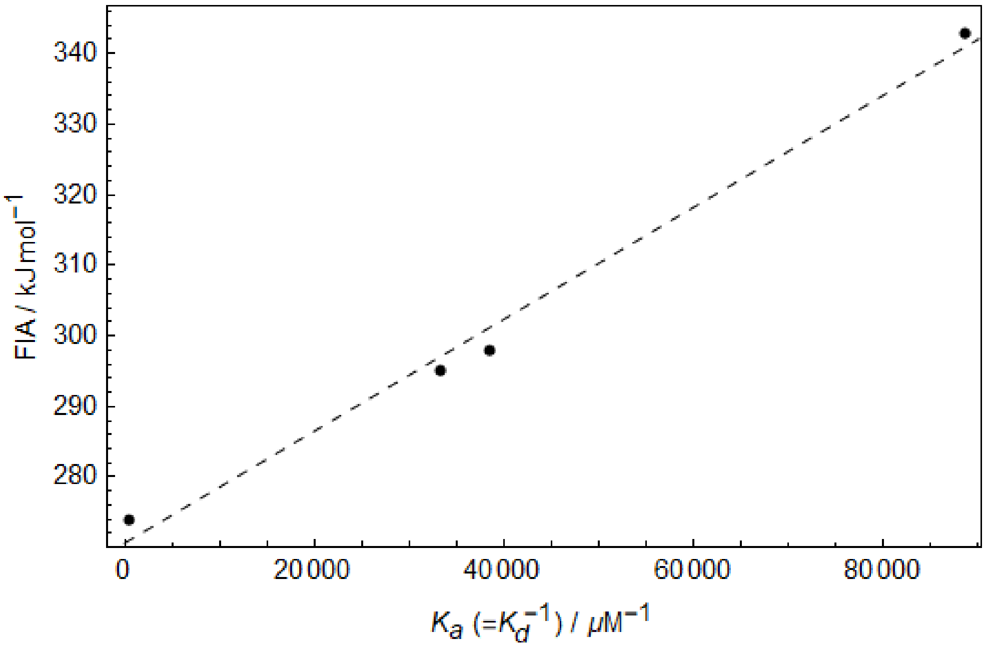

Figure S64. *Correlation between association constant K_a (inverse of K_d) and FIA for* **1⁺–4⁺**. *R^2 = 0.989.*

9. Mass spectra

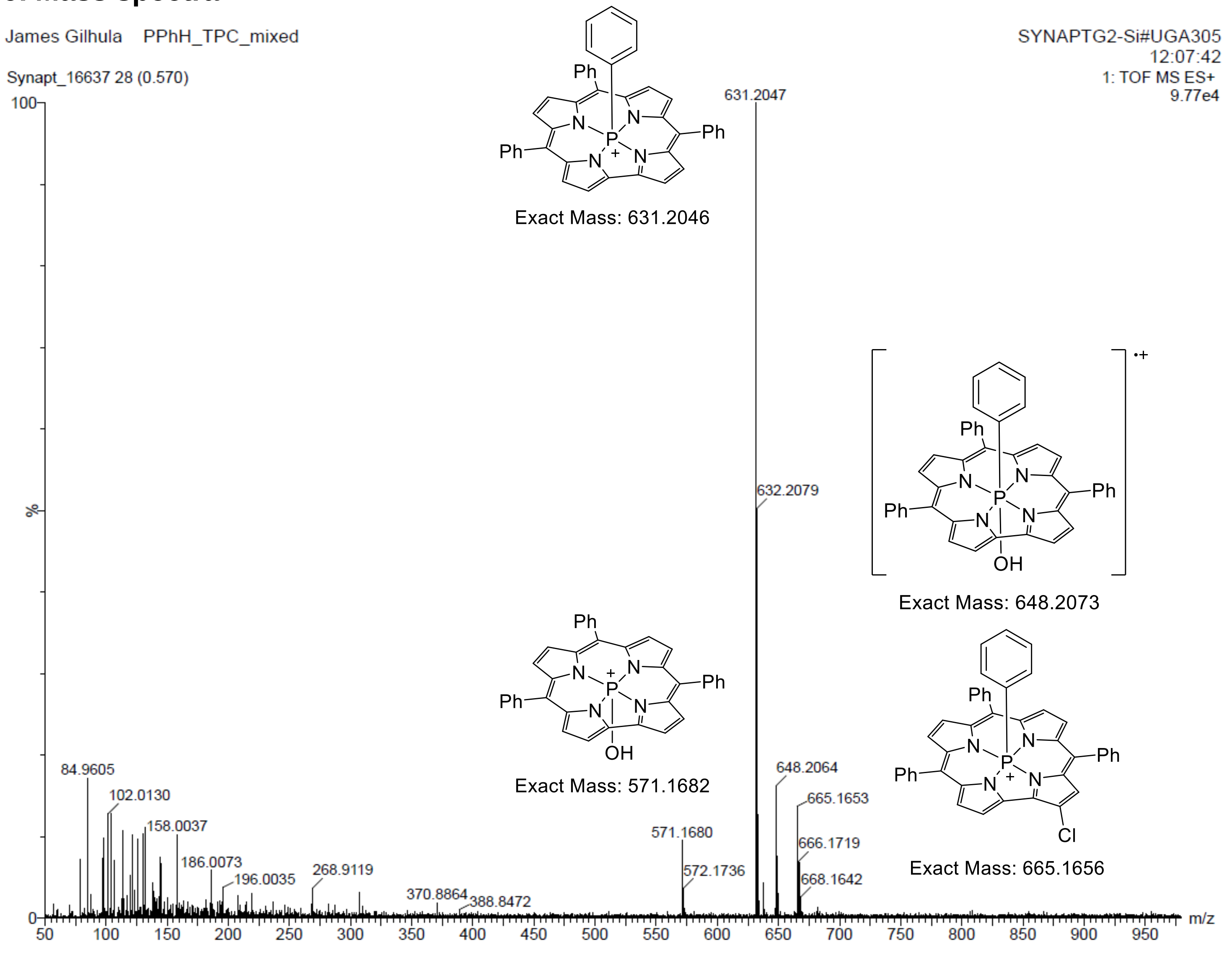

Figure S65. *HRMS (ESI) of **1•OH** before hydrodechlorination with H_2 and Pd/C.*

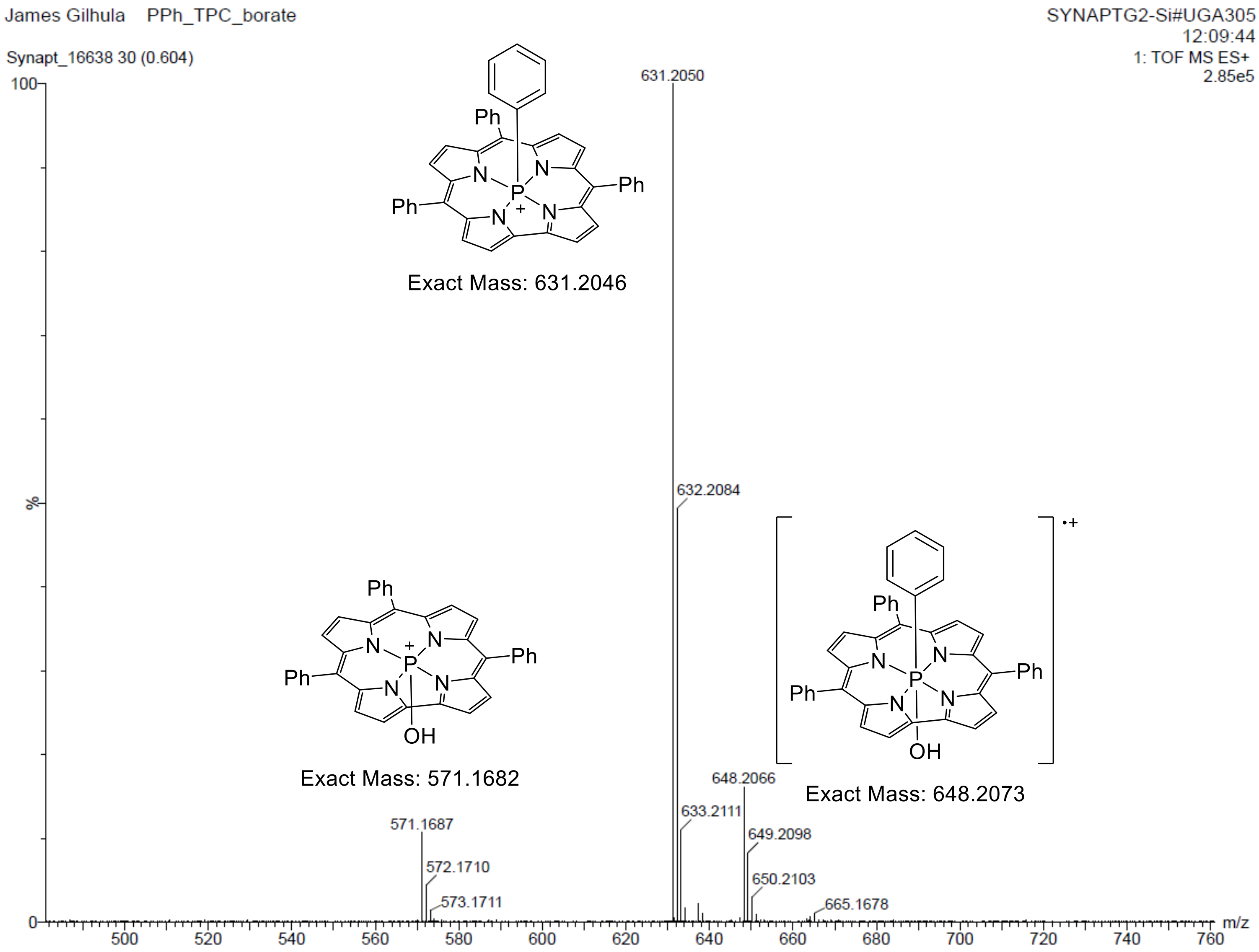

Figure S66. *HRMS (ESI) of **1**$^+$.*

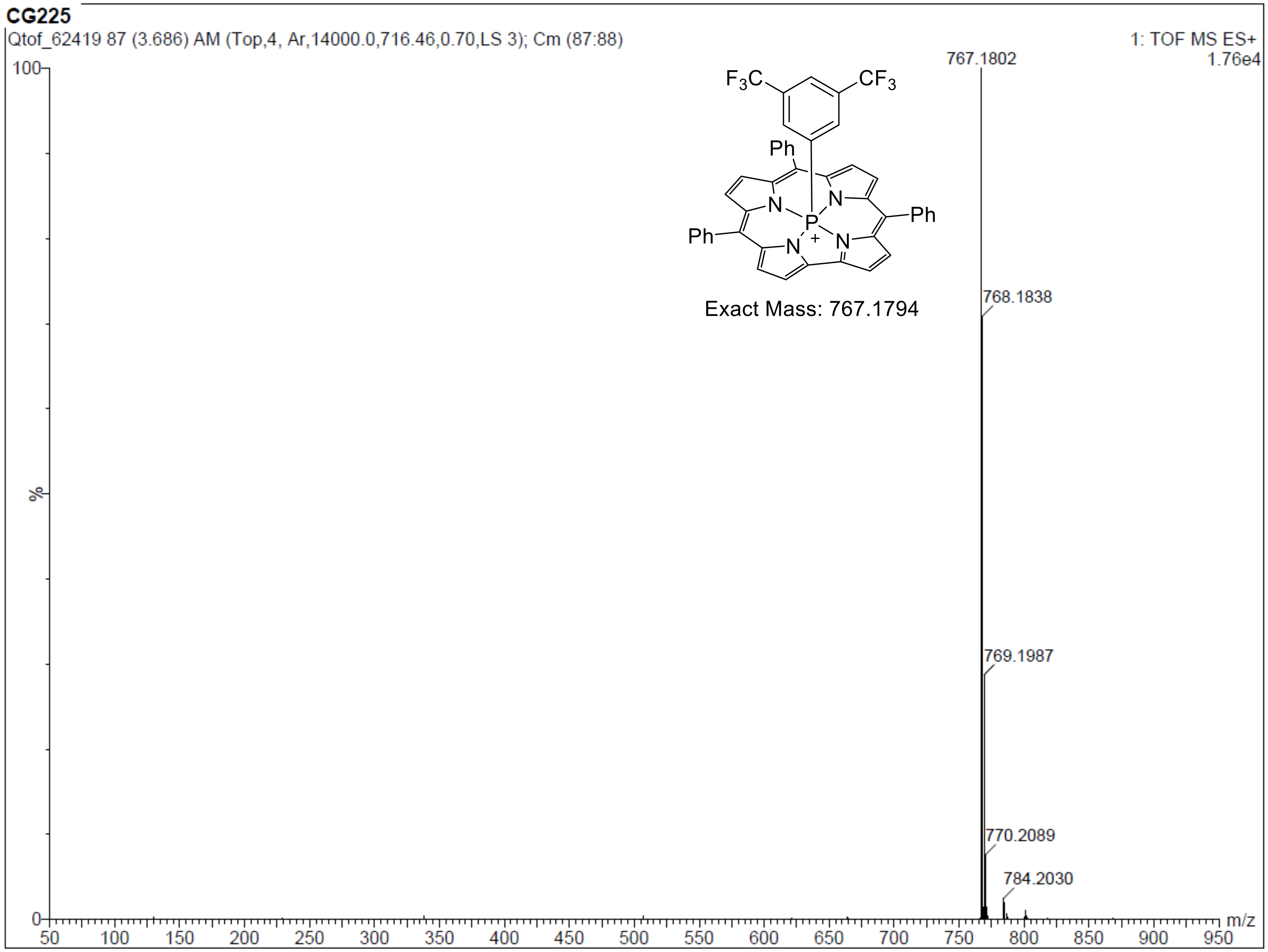

Figure S67. *HRMS (ESI) of **2•H**.*

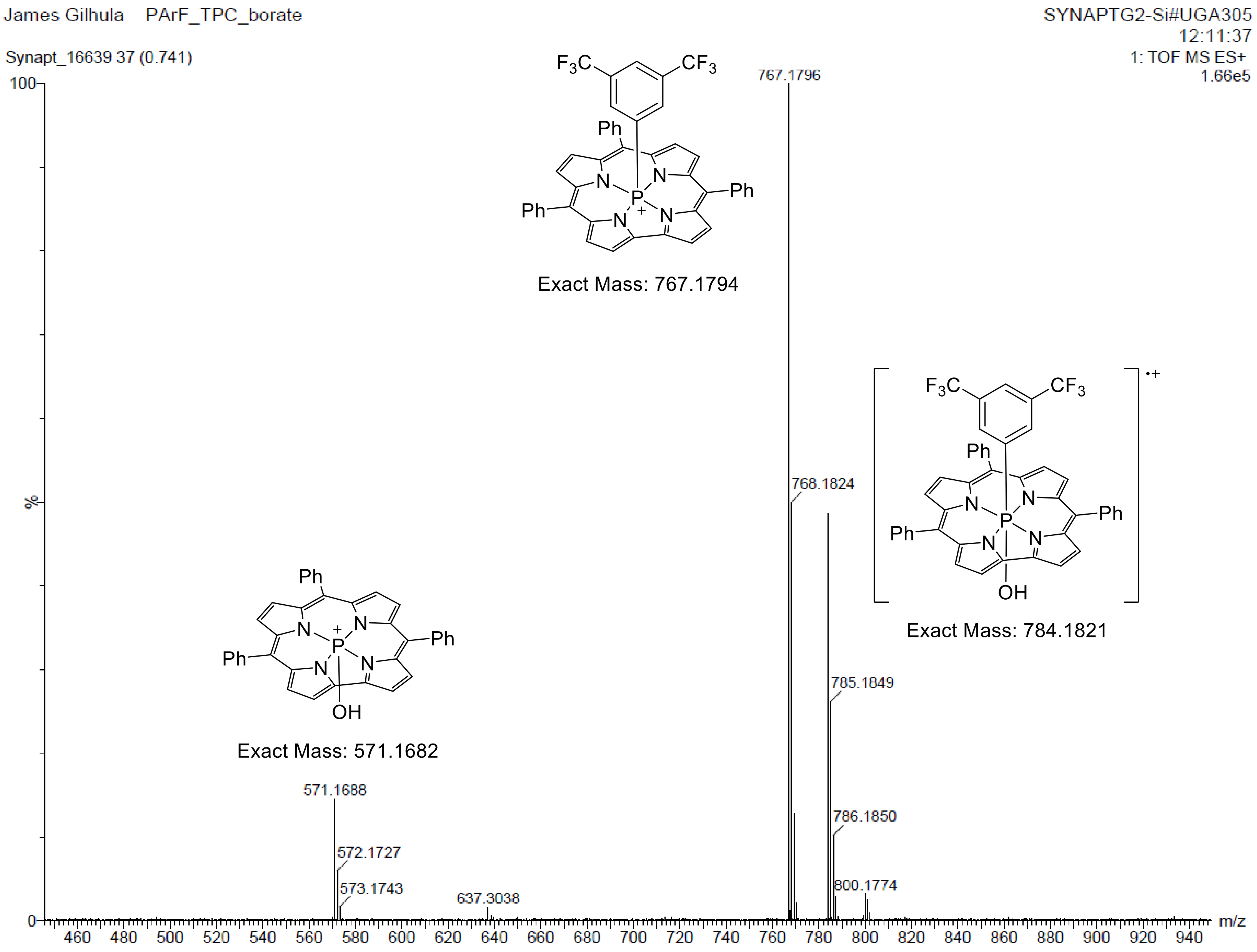

Figure S68. *HRMS (ESI) of* ***2***$^{+}$.

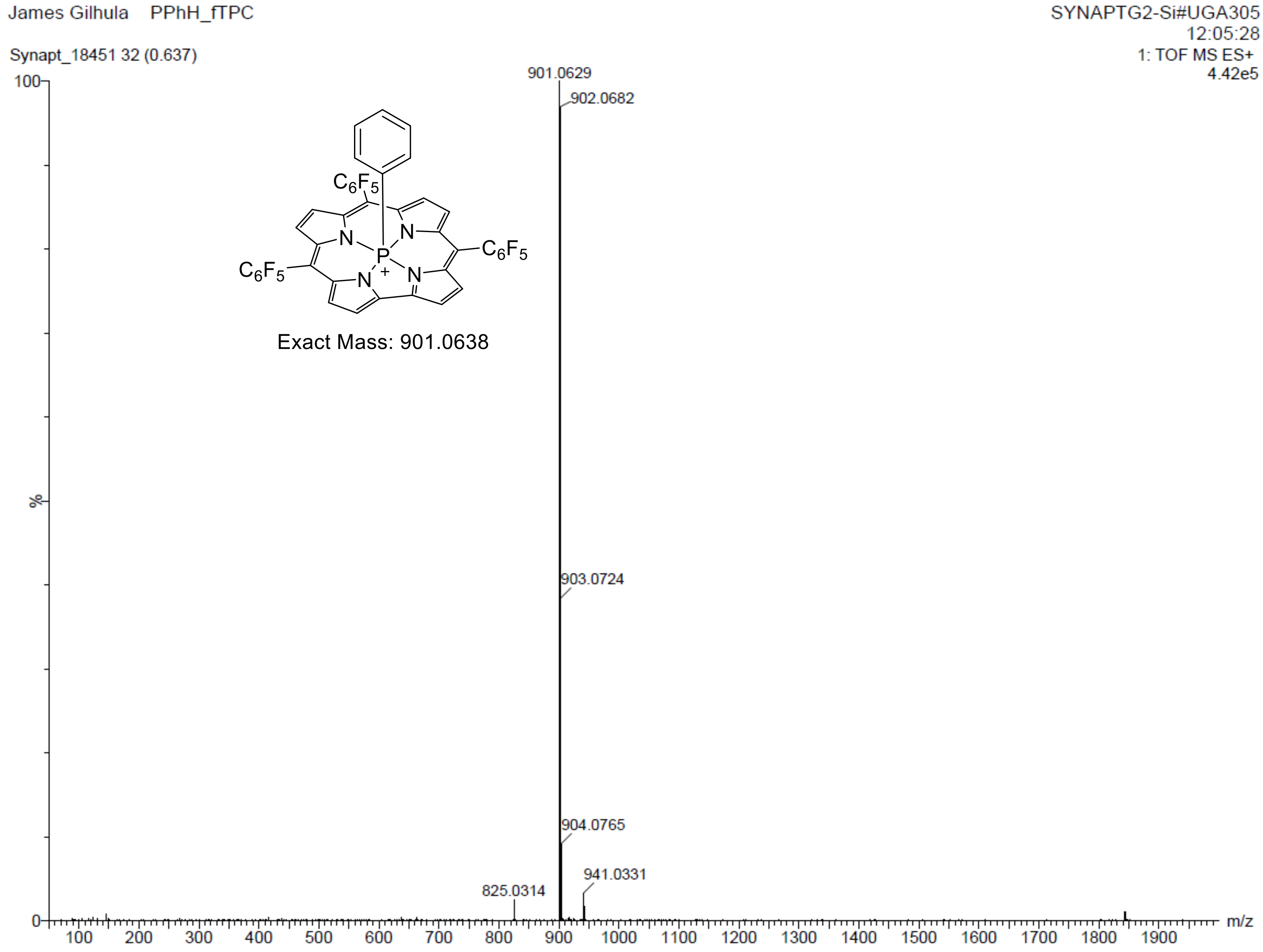

Figure S69. *HRMS (ESI) of* ***3•H****.*

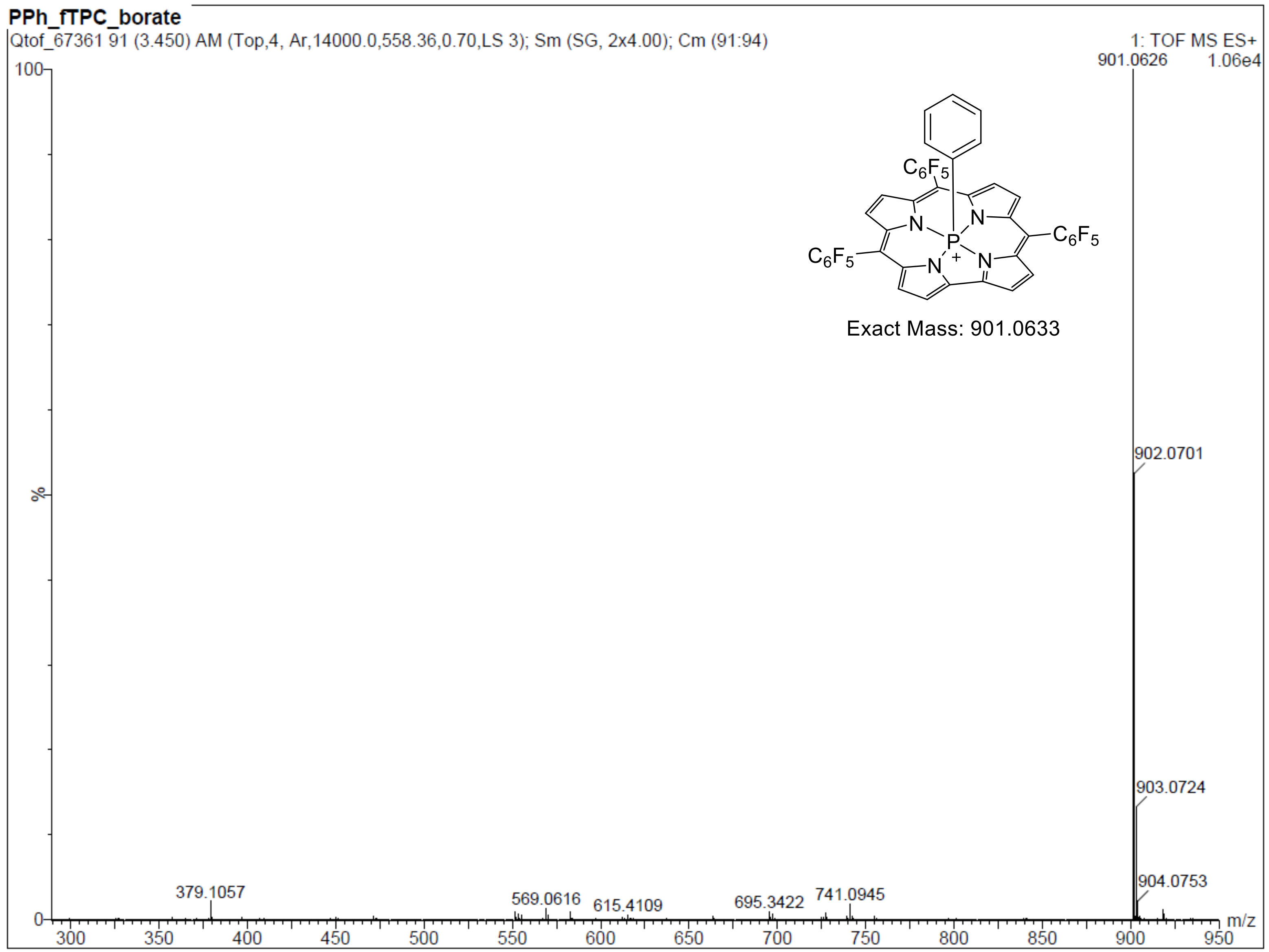

Figure S70. *HRMS (ESI) of* ***3***$^{+}$*.*

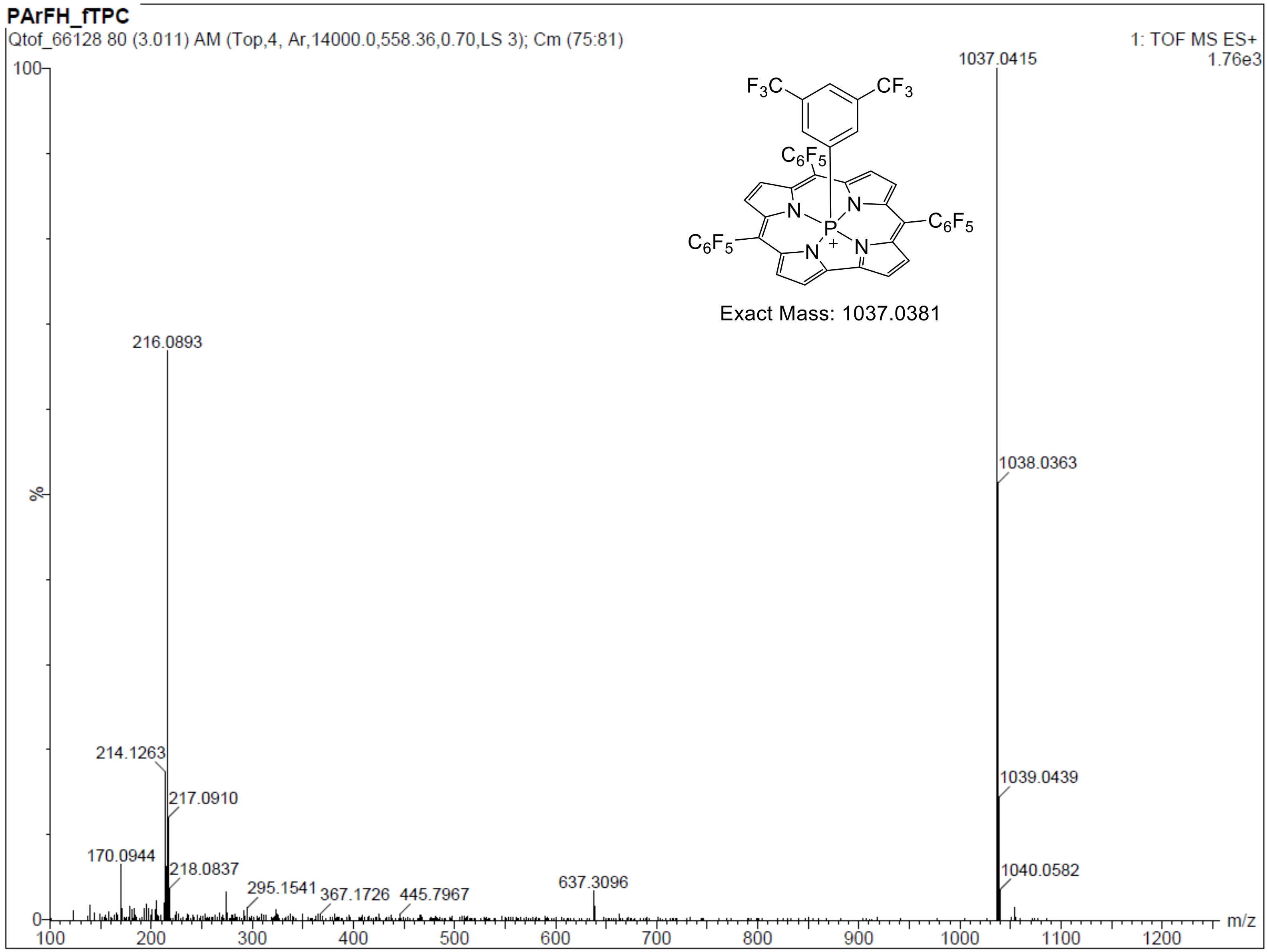

Figure S71. *HRMS (ESI) of **4•H**.*

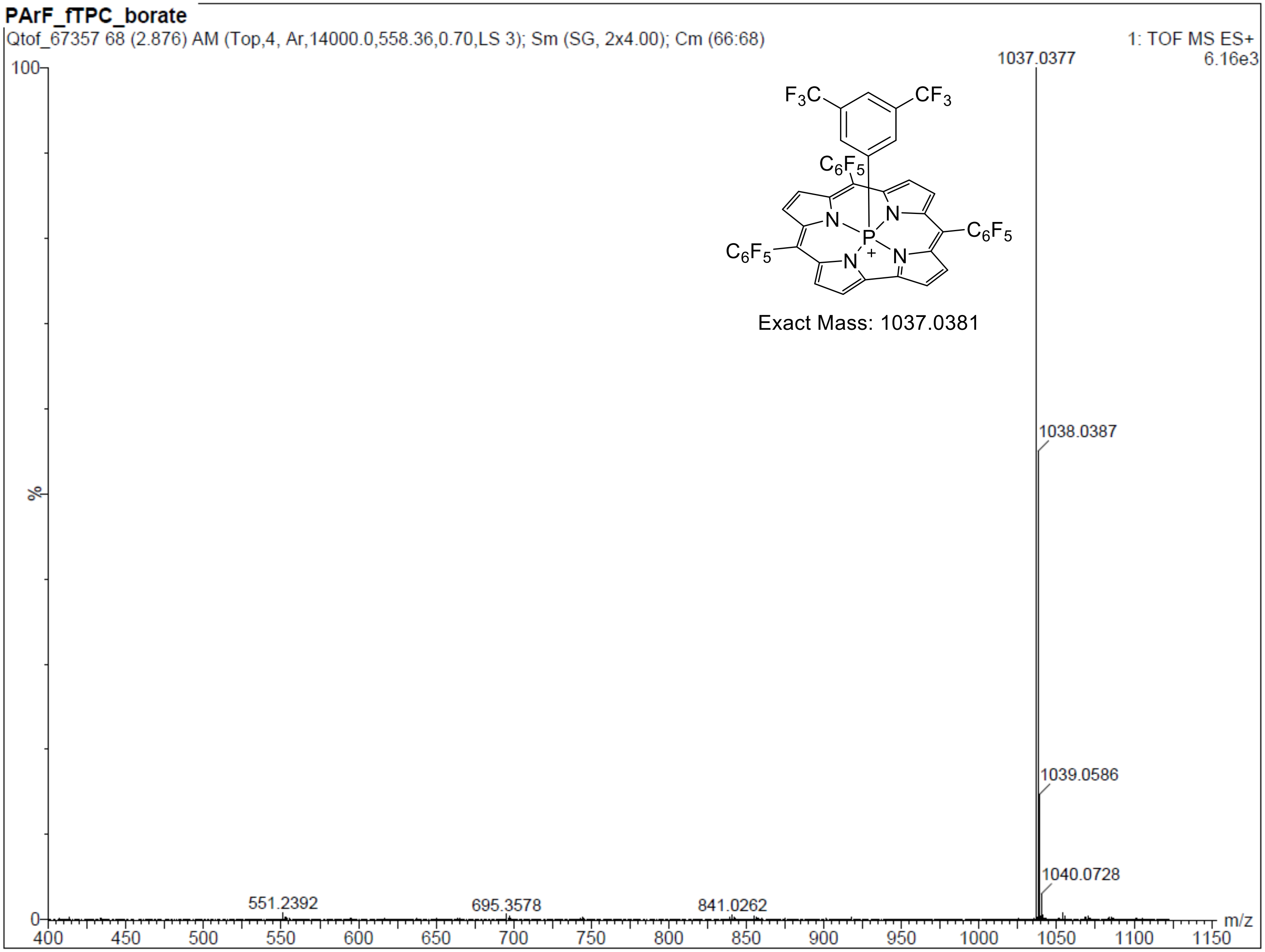

Figure S72. *HRMS (ESI) of* ***4⁺***.

10. X-Ray structures

Single crystals suitable for X-ray diffraction were grown from solutions of chloroform and pentane or dichloromethane and pentane. X-ray diffraction data was collected on a Bruker APEX-II CCD area detector system using CuKα radiation or at the ANL/APS synchrotron source. Raw data integration and reduction were performed with the SAINT and SADABS programs. Structures were solved using direct methods using SHELXT and refined using least-squares methods on F^2 using SHELX-2018 with the WinGX software package. All non-hydrogen atoms were refined with anisotropic displacement parameters. Hydrogen atoms were fixed in their ideal geometries. XP was used for graphical representations.

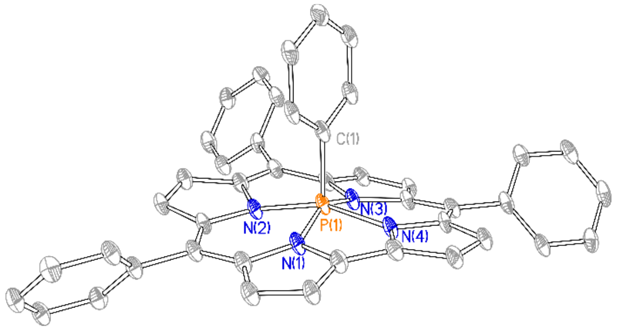

Table S1. Crystal data and structure refinement for **1**$^+$.

Identification code	pcorrole_a	
Empirical formula	C22.50 H14.50 Cl1.50 F1.50 N2 O1.50 P0.50 S0.50	
Formula weight	450.05	
Temperature	20(2) K	
Wavelength	0.41328 Å	
Crystal system	Triclinic	
Space group	P-1	
Unit cell dimensions	a = 11.5292(8) Å	a= 78.0350(10)°.
	b = 12.5645(9) Å	b= 85.101(2)°.
	c = 14.1583(10) Å	g = 85.3720(10)°.
Volume	1994.9(2) Å^3	
Z	4	
Density (calculated)	1.498 Mg/m^3	
Absorption coefficient	0.204 mm^{-1}	
F(000)	920	
Crystal size	0.01 x 0.01 x 0.01 mm^3	
Theta range for data collection	0.86 to 14.23°.	
Index ranges	-13<=h<=13, -14<=k<=14, -16<=l<=16	
Reflections collected	39814	

Independent reflections	6857 [R(int) = 0.0791]
Completeness to theta = 14.23°	97.6 %
Absorption correction	Semi-empirical from equivalents
Max. and min. transmission	0.9980 and 0.9980
Refinement method	Full-matrix least-squares on F^2
Data / restraints / parameters	6857 / 0 / 657
Goodness-of-fit on F^2	1.043
Final R indices [I>2sigma(I)]	R1 = 0.0571, wR2 = 0.1492
R indices (all data)	R1 = 0.0694, wR2 = 0.1603
Largest diff. peak and hole	0.935 and -0.525 e.$Å^{-3}$

Table S2. Atomic coordinates (x 10^4) and equivalent isotropic displacement parameters (Å^{2}x 10^3) for **1$^+$**. U(eq) is defined as one third of the trace of the orthogonalized U^{ij} tensor.

	x	y	z	U(eq)
C(44)	6517(3)	8117(3)	7894(3)	30(1)
O(1)	4632(2)	8238(2)	7028(2)	39(1)
O(2)	4503(2)	8765(2)	8602(2)	36(1)
O(3)	4823(2)	6863(2)	8499(2)	34(1)
F(1)	6779(2)	9151(2)	7537(2)	47(1)
F(2)	7002(2)	7800(2)	8736(2)	46(1)
F(3)	7044(2)	7505(2)	7290(2)	37(1)
S(1)	4937(1)	7985(1)	8018(1)	24(1)
C(45)	2452(3)	10054(3)	9491(2)	26(1)
Cl(1)	3506(1)	11007(1)	9488(1)	34(1)
Cl(2)	1623(1)	9834(1)	10622(1)	36(1)
Cl(3)	1513(1)	10532(1)	8549(1)	31(1)
C(1)	9876(2)	6941(2)	2576(2)	20(1)
C(2)	10677(3)	7760(3)	2488(2)	22(1)
C(3)	10749(3)	8011(3)	3362(2)	23(1)
C(4)	10032(2)	7323(3)	4052(2)	21(1)
C(5)	9968(2)	7228(3)	5043(2)	21(1)
C(6)	9350(2)	6408(3)	5632(2)	21(1)
C(7)	9357(3)	6105(3)	6652(2)	22(1)
C(8)	8555(3)	5348(3)	6971(2)	22(1)
C(9)	8014(3)	5144(3)	6172(2)	21(1)
C(10)	7060(3)	4515(3)	6235(2)	22(1)
C(11)	6645(3)	4382(3)	5382(2)	21(1)
C(12)	5764(3)	3731(3)	5193(2)	22(1)
C(13)	5784(3)	3792(3)	4213(2)	23(1)
C(14)	6659(3)	4498(3)	3784(2)	21(1)
C(15)	7200(3)	4882(3)	2874(2)	22(1)
C(16)	7207(3)	4697(3)	1938(2)	22(1)
C(17)	8102(3)	5273(3)	1398(2)	24(1)
C(18)	8633(3)	5834(3)	2004(2)	21(1)
C(19)	9530(3)	6537(3)	1808(2)	21(1)

C(20)	10593(3)	7969(2)	5499(2)	21(1)
C(21)	11809(3)	8027(3)	5355(2)	24(1)
C(22)	12378(3)	8724(3)	5777(2)	27(1)
C(23)	11751(3)	9369(3)	6355(2)	27(1)
C(24)	10543(3)	9312(3)	6511(2)	25(1)
C(25)	9965(3)	8623(3)	6084(2)	23(1)
C(26)	6513(2)	3991(3)	7203(2)	22(1)
C(27)	6058(3)	4636(3)	7868(2)	24(1)
C(28)	5569(3)	4140(3)	8772(2)	28(1)
C(29)	5525(3)	3013(3)	9025(2)	28(1)
C(30)	5955(3)	2372(3)	8361(2)	26(1)
C(31)	6435(3)	2864(3)	7450(2)	24(1)
C(32)	10095(3)	6859(2)	806(2)	21(1)
C(33)	9410(3)	7250(3)	19(2)	24(1)
C(34)	9932(3)	7556(3)	-916(2)	26(1)
C(35)	11139(3)	7482(3)	-1080(2)	26(1)
C(36)	11831(3)	7090(3)	-302(2)	23(1)
C(37)	11315(3)	6777(3)	628(2)	22(1)
C(38)	7105(2)	7167(2)	4022(2)	21(1)
C(39)	6690(3)	7719(3)	3138(2)	23(1)
C(40)	5929(3)	8648(3)	3097(2)	24(1)
C(41)	5569(3)	9023(3)	3951(2)	25(1)
C(42)	5972(3)	8474(3)	4829(2)	25(1)
C(43)	6741(3)	7555(3)	4873(2)	22(1)
N(1)	9451(2)	6653(2)	3574(2)	20(1)
N(2)	8567(2)	5769(2)	5292(2)	21(1)
N(3)	7179(2)	4876(2)	4487(2)	21(1)
N(4)	8064(2)	5581(2)	2938(2)	20(1)
P(1)	8130(1)	5981(1)	4075(1)	19(1)

Table S3. Bond lengths [Å] and angles [°] for **1⁺**.

C(44)-F(2)	1.331(4)
C(44)-F(1)	1.342(4)
C(44)-F(3)	1.343(4)
C(44)-S(1)	1.833(3)
O(1)-S(1)	1.439(3)
O(2)-S(1)	1.442(3)
O(3)-S(1)	1.444(3)
C(45)-Cl(3)	1.767(3)
C(45)-Cl(1)	1.772(3)
C(45)-Cl(2)	1.775(3)
C(45)-H(45)	1.00(3)
C(1)-C(19)	1.391(4)
C(1)-C(2)	1.417(4)
C(1)-N(1)	1.437(4)
C(2)-C(3)	1.350(5)
C(2)-H(2)	0.89(4)
C(3)-C(4)	1.418(4)
C(3)-H(3)	0.91(4)
C(4)-C(5)	1.381(4)
C(4)-N(1)	1.421(4)
C(5)-C(6)	1.387(4)
C(5)-C(20)	1.497(4)
C(6)-C(7)	1.415(4)
C(6)-N(2)	1.429(4)
C(7)-C(8)	1.362(4)
C(7)-H(7)	0.92(4)
C(8)-C(9)	1.414(4)
C(8)-H(8)	0.93(4)
C(9)-C(10)	1.392(4)
C(9)-N(2)	1.454(4)
C(10)-C(11)	1.383(4)
C(10)-C(26)	1.501(4)
C(11)-N(3)	1.404(4)
C(11)-C(12)	1.429(4)

C(12)-C(13)	1.372(5)
C(12)-H(12)	1.00(4)
C(13)-C(14)	1.411(4)
C(13)-H(13)	0.81(5)
C(14)-N(3)	1.385(4)
C(14)-C(15)	1.392(4)
C(15)-C(16)	1.391(5)
C(15)-N(4)	1.401(4)
C(16)-C(17)	1.389(4)
C(16)-H(16)	0.88(4)
C(17)-C(18)	1.421(4)
C(17)-H(17)	1.00(4)
C(18)-C(19)	1.387(4)
C(18)-N(4)	1.411(4)
C(19)-C(32)	1.499(4)
C(20)-C(25)	1.403(5)
C(20)-C(21)	1.405(4)
C(21)-C(22)	1.389(5)
C(21)-H(21)	0.99(4)
C(22)-C(23)	1.389(5)
C(22)-H(22)	0.92(4)
C(23)-C(24)	1.397(5)
C(23)-H(23)	0.94(4)
C(24)-C(25)	1.393(5)
C(24)-H(24)	0.91(3)
C(25)-H(25)	0.87(4)
C(26)-C(31)	1.395(5)
C(26)-C(27)	1.409(5)
C(27)-C(28)	1.396(5)
C(27)-H(27)	0.94(4)
C(28)-C(29)	1.391(5)
C(28)-H(28)	0.90(4)
C(29)-C(30)	1.396(5)
C(29)-H(29)	0.93(5)
C(30)-C(31)	1.398(5)
C(30)-H(30)	0.88(3)

C(31)-H(31)	0.87(3)
C(32)-C(33)	1.403(5)
C(32)-C(37)	1.407(4)
C(33)-C(34)	1.397(5)
C(33)-H(33)	0.95(4)
C(34)-C(35)	1.390(5)
C(34)-H(34)	0.79(4)
C(35)-C(36)	1.398(5)
C(35)-H(35)	0.94(4)
C(36)-C(37)	1.390(4)
C(36)-H(36)	0.92(4)
C(37)-H(37)	0.87(4)
C(38)-C(39)	1.401(4)
C(38)-C(43)	1.409(4)
C(38)-P(1)	1.819(3)
C(39)-C(40)	1.396(5)
C(39)-H(39)	0.92(4)
C(40)-C(41)	1.406(5)
C(40)-H(40)	0.95(4)
C(41)-C(42)	1.389(5)
C(41)-H(41)	0.91(4)
C(42)-C(43)	1.393(5)
C(42)-H(42)	0.94(4)
C(43)-H(43)	0.91(3)
N(1)-P(1)	1.819(2)
N(2)-P(1)	1.797(3)
N(3)-P(1)	1.809(3)
N(4)-P(1)	1.792(3)
F(2)-C(44)-F(1)	107.4(3)
F(2)-C(44)-F(3)	107.1(3)
F(1)-C(44)-F(3)	107.3(3)
F(2)-C(44)-S(1)	112.3(2)
F(1)-C(44)-S(1)	111.5(2)
F(3)-C(44)-S(1)	111.0(2)
O(1)-S(1)-O(2)	115.96(16)
O(1)-S(1)-O(3)	114.77(16)

O(2)-S(1)-O(3)	113.99(15)
O(1)-S(1)-C(44)	102.55(16)
O(2)-S(1)-C(44)	103.72(15)
O(3)-S(1)-C(44)	103.51(15)
Cl(3)-C(45)-Cl(1)	110.67(18)
Cl(3)-C(45)-Cl(2)	109.57(17)
Cl(1)-C(45)-Cl(2)	109.63(18)
Cl(3)-C(45)-H(45)	109.5(17)
Cl(1)-C(45)-H(45)	105.5(18)
Cl(2)-C(45)-H(45)	111.9(17)
C(19)-C(1)-C(2)	124.9(3)
C(19)-C(1)-N(1)	126.7(3)
C(2)-C(1)-N(1)	108.4(3)
C(3)-C(2)-C(1)	109.2(3)
C(3)-C(2)-H(2)	128(2)
C(1)-C(2)-H(2)	122(2)
C(2)-C(3)-C(4)	108.3(3)
C(2)-C(3)-H(3)	126(2)
C(4)-C(3)-H(3)	125(2)
C(5)-C(4)-C(3)	126.6(3)
C(5)-C(4)-N(1)	123.8(3)
C(3)-C(4)-N(1)	109.3(3)
C(4)-C(5)-C(6)	119.7(3)
C(4)-C(5)-C(20)	121.0(3)
C(6)-C(5)-C(20)	119.2(3)
C(5)-C(6)-C(7)	125.9(3)
C(5)-C(6)-N(2)	124.3(3)
C(7)-C(6)-N(2)	109.8(3)
C(8)-C(7)-C(6)	108.2(3)
C(8)-C(7)-H(7)	128(2)
C(6)-C(7)-H(7)	124(2)
C(7)-C(8)-C(9)	109.4(3)
C(7)-C(8)-H(8)	123(2)
C(9)-C(8)-H(8)	128(2)
C(10)-C(9)-C(8)	125.1(3)
C(10)-C(9)-N(2)	126.4(3)

C(8)-C(9)-N(2)	108.3(2)
C(11)-C(10)-C(9)	117.9(3)
C(11)-C(10)-C(26)	121.4(3)
C(9)-C(10)-C(26)	120.6(3)
C(10)-C(11)-N(3)	120.2(3)
C(10)-C(11)-C(12)	131.9(3)
N(3)-C(11)-C(12)	107.7(3)
C(13)-C(12)-C(11)	108.7(3)
C(13)-C(12)-H(12)	128(2)
C(11)-C(12)-H(12)	124(2)
C(12)-C(13)-C(14)	106.7(3)
C(12)-C(13)-H(13)	124(3)
C(14)-C(13)-H(13)	128(3)
N(3)-C(14)-C(15)	110.5(3)
N(3)-C(14)-C(13)	110.4(3)
C(15)-C(14)-C(13)	139.0(3)
C(16)-C(15)-C(14)	138.4(3)
C(16)-C(15)-N(4)	110.9(3)
C(14)-C(15)-N(4)	110.4(3)
C(17)-C(16)-C(15)	106.7(3)
C(17)-C(16)-H(16)	128(3)
C(15)-C(16)-H(16)	125(3)
C(16)-C(17)-C(18)	108.7(3)
C(16)-C(17)-H(17)	128(2)
C(18)-C(17)-H(17)	123(2)
C(19)-C(18)-N(4)	121.0(3)
C(19)-C(18)-C(17)	131.2(3)
N(4)-C(18)-C(17)	107.8(3)
C(18)-C(19)-C(1)	117.4(3)
C(18)-C(19)-C(32)	121.4(3)
C(1)-C(19)-C(32)	121.3(3)
C(25)-C(20)-C(21)	118.7(3)
C(25)-C(20)-C(5)	120.0(3)
C(21)-C(20)-C(5)	121.3(3)
C(22)-C(21)-C(20)	120.7(3)
C(22)-C(21)-H(21)	123(2)

C(20)-C(21)-H(21)	116(2)
C(23)-C(22)-C(21)	120.3(3)
C(23)-C(22)-H(22)	120(2)
C(21)-C(22)-H(22)	119(2)
C(22)-C(23)-C(24)	119.6(3)
C(22)-C(23)-H(23)	121(2)
C(24)-C(23)-H(23)	119(2)
C(25)-C(24)-C(23)	120.4(3)
C(25)-C(24)-H(24)	121(2)
C(23)-C(24)-H(24)	119(2)
C(24)-C(25)-C(20)	120.3(3)
C(24)-C(25)-H(25)	120(2)
C(20)-C(25)-H(25)	120(2)
C(31)-C(26)-C(27)	119.1(3)
C(31)-C(26)-C(10)	120.6(3)
C(27)-C(26)-C(10)	120.2(3)
C(28)-C(27)-C(26)	119.8(3)
C(28)-C(27)-H(27)	120(2)
C(26)-C(27)-H(27)	120(2)
C(29)-C(28)-C(27)	120.7(3)
C(29)-C(28)-H(28)	119(2)
C(27)-C(28)-H(28)	120(2)
C(28)-C(29)-C(30)	119.7(3)
C(28)-C(29)-H(29)	119(3)
C(30)-C(29)-H(29)	121(3)
C(29)-C(30)-C(31)	119.9(3)
C(29)-C(30)-H(30)	120(2)
C(31)-C(30)-H(30)	120(2)
C(26)-C(31)-C(30)	120.7(3)
C(26)-C(31)-H(31)	124(2)
C(30)-C(31)-H(31)	115(2)
C(33)-C(32)-C(37)	118.3(3)
C(33)-C(32)-C(19)	120.3(3)
C(37)-C(32)-C(19)	121.4(3)
C(34)-C(33)-C(32)	120.5(3)
C(34)-C(33)-H(33)	123(2)

C(32)-C(33)-H(33)	116(2)
C(35)-C(34)-C(33)	120.5(3)
C(35)-C(34)-H(34)	118(3)
C(33)-C(34)-H(34)	121(3)
C(34)-C(35)-C(36)	119.5(3)
C(34)-C(35)-H(35)	118(2)
C(36)-C(35)-H(35)	122(2)
C(37)-C(36)-C(35)	120.2(3)
C(37)-C(36)-H(36)	123(2)
C(35)-C(36)-H(36)	117(2)
C(36)-C(37)-C(32)	120.9(3)
C(36)-C(37)-H(37)	125(3)
C(32)-C(37)-H(37)	114(3)
C(39)-C(38)-C(43)	119.4(3)
C(39)-C(38)-P(1)	120.7(2)
C(43)-C(38)-P(1)	119.9(2)
C(40)-C(39)-C(38)	120.5(3)
C(40)-C(39)-H(39)	120(2)
C(38)-C(39)-H(39)	119(2)
C(39)-C(40)-C(41)	119.6(3)
C(39)-C(40)-H(40)	121(2)
C(41)-C(40)-H(40)	119(2)
C(42)-C(41)-C(40)	120.1(3)
C(42)-C(41)-H(41)	121(2)
C(40)-C(41)-H(41)	118(2)
C(41)-C(42)-C(43)	120.5(3)
C(41)-C(42)-H(42)	122(2)
C(43)-C(42)-H(42)	118(2)
C(42)-C(43)-C(38)	119.9(3)
C(42)-C(43)-H(43)	118(2)
C(38)-C(43)-H(43)	122(2)
C(4)-N(1)-C(1)	104.7(2)
C(4)-N(1)-P(1)	123.9(2)
C(1)-N(1)-P(1)	128.2(2)
C(6)-N(2)-C(9)	104.0(2)
C(6)-N(2)-P(1)	125.7(2)

C(9)-N(2)-P(1)	128.5(2)
C(14)-N(3)-C(11)	106.5(2)
C(14)-N(3)-P(1)	117.0(2)
C(11)-N(3)-P(1)	135.5(2)
C(15)-N(4)-C(18)	105.9(2)
C(15)-N(4)-P(1)	117.3(2)
C(18)-N(4)-P(1)	136.7(2)
N(4)-P(1)-N(2)	152.91(13)
N(4)-P(1)-N(3)	82.11(12)
N(2)-P(1)-N(3)	87.78(12)
N(4)-P(1)-C(38)	104.67(13)
N(2)-P(1)-C(38)	101.95(13)
N(3)-P(1)-C(38)	101.67(12)
N(4)-P(1)-N(1)	87.86(11)
N(2)-P(1)-N(1)	92.52(11)
N(3)-P(1)-N(1)	158.07(12)
C(38)-P(1)-N(1)	99.71(13)

Symmetry transformations used to generate equivalent atoms:

Table S4. Anisotropic displacement parameters ($Å^2 x 10^3$) for **1⁺.** The anisotropic displacement factor exponent takes the form: $-2p^2[h^2 a^{*2}U^{11} + ... + 2 h k a^* b^* U^{12}]$

	U^{11}	U^{22}	U^{33}	U^{23}	U^{13}	U^{12}
C(44)	17(2)	40(2)	35(2)	-12(2)	1(1)	-7(1)
O(1)	25(1)	62(2)	29(1)	-5(1)	-1(1)	-8(1)
O(2)	18(1)	50(2)	40(2)	-16(1)	6(1)	-6(1)
O(3)	21(1)	42(1)	39(1)	-7(1)	5(1)	-8(1)
F(1)	28(1)	50(1)	66(2)	-18(1)	13(1)	-17(1)
F(2)	22(1)	83(2)	38(1)	-20(1)	-7(1)	-1(1)
F(3)	18(1)	56(1)	42(1)	-23(1)	7(1)	-6(1)
S(1)	14(1)	35(1)	24(1)	-6(1)	0(1)	-6(1)
C(45)	16(2)	35(2)	28(2)	-6(1)	6(1)	-7(1)
Cl(1)	18(1)	54(1)	35(1)	-16(1)	4(1)	-13(1)
Cl(2)	32(1)	40(1)	32(1)	-1(1)	13(1)	-4(1)
Cl(3)	19(1)	42(1)	33(1)	-9(1)	-1(1)	-7(1)
C(1)	10(1)	31(2)	19(2)	-4(1)	3(1)	-3(1)
C(2)	13(1)	34(2)	20(2)	-4(1)	4(1)	-5(1)
C(3)	11(1)	32(2)	25(2)	-5(1)	2(1)	-7(1)
C(4)	11(1)	30(2)	23(2)	-6(1)	0(1)	-4(1)
C(5)	10(1)	30(2)	22(2)	-6(1)	1(1)	-3(1)
C(6)	10(1)	33(2)	22(2)	-9(1)	1(1)	-5(1)
C(7)	12(1)	33(2)	22(2)	-9(1)	0(1)	-4(1)
C(8)	15(2)	29(2)	20(2)	-3(1)	3(1)	-3(1)
C(9)	12(1)	30(2)	20(2)	-4(1)	4(1)	-4(1)
C(10)	12(1)	31(2)	23(2)	-7(1)	2(1)	-2(1)
C(11)	12(1)	29(2)	21(2)	-3(1)	6(1)	-6(1)
C(12)	9(1)	31(2)	26(2)	-5(1)	3(1)	-6(1)
C(13)	10(1)	32(2)	29(2)	-9(1)	3(1)	-7(1)
C(14)	13(1)	31(2)	22(2)	-7(1)	0(1)	-3(1)
C(15)	10(1)	30(2)	26(2)	-6(1)	0(1)	-7(1)
C(16)	13(1)	32(2)	23(2)	-6(1)	2(1)	-7(1)
C(17)	13(2)	36(2)	22(2)	-8(1)	3(1)	-6(1)
C(18)	13(1)	31(2)	20(2)	-6(1)	1(1)	-4(1)
C(19)	11(1)	31(2)	21(2)	-6(1)	3(1)	-4(1)

C(20)	15(1)	30(2)	18(2)	-3(1)	1(1)	-7(1)
C(21)	14(2)	36(2)	23(2)	-7(1)	2(1)	-5(1)
C(22)	15(2)	42(2)	25(2)	-6(1)	0(1)	-8(1)
C(23)	22(2)	34(2)	26(2)	-7(1)	-1(1)	-13(1)
C(24)	21(2)	31(2)	25(2)	-9(1)	2(1)	-4(1)
C(25)	13(2)	36(2)	21(2)	-6(1)	2(1)	-6(1)
C(26)	7(1)	37(2)	21(2)	-5(1)	-1(1)	-5(1)
C(27)	12(1)	36(2)	25(2)	-5(1)	-1(1)	-5(1)
C(28)	13(2)	49(2)	25(2)	-11(2)	2(1)	-6(1)
C(29)	14(2)	48(2)	22(2)	-2(2)	0(1)	-11(1)
C(30)	11(2)	38(2)	28(2)	-1(1)	-3(1)	-9(1)
C(31)	9(1)	39(2)	24(2)	-8(1)	1(1)	-6(1)
C(32)	13(1)	30(2)	20(2)	-6(1)	3(1)	-6(1)
C(33)	15(2)	35(2)	25(2)	-8(1)	4(1)	-6(1)
C(34)	21(2)	36(2)	20(2)	-4(1)	-4(1)	-3(1)
C(35)	21(2)	34(2)	22(2)	-7(1)	6(1)	-7(1)
C(36)	13(2)	34(2)	24(2)	-8(1)	4(1)	-7(1)
C(37)	16(2)	29(2)	23(2)	-6(1)	-1(1)	-7(1)
C(38)	8(1)	31(2)	24(2)	-6(1)	2(1)	-7(1)
C(39)	13(1)	34(2)	22(2)	-6(1)	6(1)	-8(1)
C(40)	14(2)	35(2)	23(2)	-2(1)	1(1)	-7(1)
C(41)	12(1)	31(2)	32(2)	-7(1)	3(1)	-6(1)
C(42)	16(2)	34(2)	26(2)	-11(1)	6(1)	-9(1)
C(43)	12(1)	35(2)	21(2)	-5(1)	1(1)	-8(1)
N(1)	11(1)	30(1)	20(1)	-6(1)	4(1)	-6(1)
N(2)	10(1)	31(1)	22(1)	-6(1)	3(1)	-8(1)
N(3)	12(1)	32(1)	19(1)	-7(1)	4(1)	-7(1)
N(4)	10(1)	32(1)	19(1)	-6(1)	3(1)	-7(1)
P(1)	9(1)	30(1)	19(1)	-5(1)	3(1)	-6(1)

Table S5. Hydrogen coordinates (x 10^4) and isotropic displacement parameters ($Å^2$x 10^3) for **1^+.**

	x	y	z	U(eq)
H(45)	2910(30)	9370(20)	9380(20)	9(7)
H(43)	6920(30)	7170(30)	5470(30)	15(8)
H(25)	9210(40)	8640(30)	6140(30)	23(9)
H(12)	5270(30)	3280(30)	5710(30)	23(9)
H(39)	6970(30)	7500(30)	2580(30)	29(9)
H(7)	9850(40)	6380(30)	7010(30)	30(10)
H(8)	8420(30)	5020(30)	7620(30)	24(9)
H(42)	5700(30)	8680(30)	5420(30)	26(9)
H(2)	11010(30)	8070(30)	1920(30)	28(9)
H(13)	5430(40)	3410(40)	3960(30)	47(13)
H(36)	12620(40)	7050(30)	-440(30)	27(9)
H(40)	5670(30)	9050(30)	2500(30)	20(8)
H(33)	8590(40)	7300(30)	160(30)	29(9)
H(37)	11700(30)	6510(30)	1140(30)	29(10)
H(16)	6740(40)	4270(30)	1750(30)	31(10)
H(30)	6010(30)	1660(30)	8540(20)	8(7)
H(35)	11460(30)	7710(30)	-1720(30)	30(10)
H(24)	10150(30)	9730(30)	6900(20)	11(7)
H(17)	8370(40)	5310(30)	700(30)	38(10)
H(22)	13180(40)	8700(30)	5720(30)	32(10)
H(29)	5300(40)	2690(30)	9660(30)	41(11)
H(34)	9560(40)	7820(30)	-1360(30)	29(10)
H(23)	12130(30)	9820(30)	6670(30)	22(8)
H(27)	6100(30)	5390(30)	7710(30)	23(9)
H(3)	11170(30)	8540(30)	3490(20)	20(8)
H(31)	6720(30)	2410(20)	7080(20)	9(7)
H(28)	5260(30)	4550(30)	9190(30)	29(10)
H(41)	5110(30)	9660(30)	3900(20)	22(9)
H(21)	12220(30)	7530(30)	4960(30)	20(8)

Table S6. Torsion angles [°] for **1$^+$**.

F(2)-C(44)-S(1)-O(1)	-173.3(2)
F(1)-C(44)-S(1)-O(1)	66.1(3)
F(3)-C(44)-S(1)-O(1)	-53.4(3)
F(2)-C(44)-S(1)-O(2)	65.7(3)
F(1)-C(44)-S(1)-O(2)	-54.9(3)
F(3)-C(44)-S(1)-O(2)	-174.4(2)
F(2)-C(44)-S(1)-O(3)	-53.6(3)
F(1)-C(44)-S(1)-O(3)	-174.2(2)
F(3)-C(44)-S(1)-O(3)	66.3(3)
C(19)-C(1)-C(2)-C(3)	175.6(3)
N(1)-C(1)-C(2)-C(3)	-1.4(4)
C(1)-C(2)-C(3)-C(4)	2.5(4)
C(2)-C(3)-C(4)-C(5)	171.8(3)
C(2)-C(3)-C(4)-N(1)	-2.7(4)
C(3)-C(4)-C(5)-C(6)	-170.8(3)
N(1)-C(4)-C(5)-C(6)	3.0(5)
C(3)-C(4)-C(5)-C(20)	6.8(5)
N(1)-C(4)-C(5)-C(20)	-179.4(3)
C(4)-C(5)-C(6)-C(7)	169.6(3)
C(20)-C(5)-C(6)-C(7)	-8.1(5)
C(4)-C(5)-C(6)-N(2)	-13.1(5)
C(20)-C(5)-C(6)-N(2)	169.2(3)
C(5)-C(6)-C(7)-C(8)	173.9(3)
N(2)-C(6)-C(7)-C(8)	-3.7(4)
C(6)-C(7)-C(8)-C(9)	0.0(4)
C(7)-C(8)-C(9)-C(10)	-172.1(3)
C(7)-C(8)-C(9)-N(2)	3.6(3)
C(8)-C(9)-C(10)-C(11)	-179.0(3)
N(2)-C(9)-C(10)-C(11)	6.0(5)
C(8)-C(9)-C(10)-C(26)	0.4(5)
N(2)-C(9)-C(10)-C(26)	-174.6(3)
C(9)-C(10)-C(11)-N(3)	-0.2(4)
C(26)-C(10)-C(11)-N(3)	-179.6(3)
C(9)-C(10)-C(11)-C(12)	174.2(3)

C(26)-C(10)-C(11)-C(12)	-5.2(5)
C(10)-C(11)-C(12)-C(13)	-172.8(3)
N(3)-C(11)-C(12)-C(13)	2.1(4)
C(11)-C(12)-C(13)-C(14)	-1.2(4)
C(12)-C(13)-C(14)-N(3)	-0.2(4)
C(12)-C(13)-C(14)-C(15)	175.4(4)
N(3)-C(14)-C(15)-C(16)	169.1(4)
C(13)-C(14)-C(15)-C(16)	-6.4(7)
N(3)-C(14)-C(15)-N(4)	-3.1(4)
C(13)-C(14)-C(15)-N(4)	-178.6(4)
C(14)-C(15)-C(16)-C(17)	-171.3(4)
N(4)-C(15)-C(16)-C(17)	0.9(4)
C(15)-C(16)-C(17)-C(18)	-1.1(4)
C(16)-C(17)-C(18)-C(19)	-177.9(3)
C(16)-C(17)-C(18)-N(4)	1.0(4)
N(4)-C(18)-C(19)-C(1)	2.7(4)
C(17)-C(18)-C(19)-C(1)	-178.5(3)
N(4)-C(18)-C(19)-C(32)	-176.6(3)
C(17)-C(18)-C(19)-C(32)	2.2(5)
C(2)-C(1)-C(19)-C(18)	-172.2(3)
N(1)-C(1)-C(19)-C(18)	4.3(5)
C(2)-C(1)-C(19)-C(32)	7.1(5)
N(1)-C(1)-C(19)-C(32)	-176.4(3)
C(4)-C(5)-C(20)-C(25)	121.3(3)
C(6)-C(5)-C(20)-C(25)	-61.0(4)
C(4)-C(5)-C(20)-C(21)	-58.8(4)
C(6)-C(5)-C(20)-C(21)	118.8(3)
C(25)-C(20)-C(21)-C(22)	-0.6(5)
C(5)-C(20)-C(21)-C(22)	179.6(3)
C(20)-C(21)-C(22)-C(23)	0.5(5)
C(21)-C(22)-C(23)-C(24)	0.1(5)
C(22)-C(23)-C(24)-C(25)	-0.6(5)
C(23)-C(24)-C(25)-C(20)	0.6(5)
C(21)-C(20)-C(25)-C(24)	0.0(5)
C(5)-C(20)-C(25)-C(24)	179.9(3)
C(11)-C(10)-C(26)-C(31)	57.5(4)

C(9)-C(10)-C(26)-C(31)	-121.9(3)
C(11)-C(10)-C(26)-C(27)	-121.7(3)
C(9)-C(10)-C(26)-C(27)	58.9(4)
C(31)-C(26)-C(27)-C(28)	1.9(4)
C(10)-C(26)-C(27)-C(28)	-178.9(3)
C(26)-C(27)-C(28)-C(29)	0.0(5)
C(27)-C(28)-C(29)-C(30)	-1.3(5)
C(28)-C(29)-C(30)-C(31)	0.6(5)
C(27)-C(26)-C(31)-C(30)	-2.6(4)
C(10)-C(26)-C(31)-C(30)	178.2(3)
C(29)-C(30)-C(31)-C(26)	1.4(4)
C(18)-C(19)-C(32)-C(33)	50.5(4)
C(1)-C(19)-C(32)-C(33)	-128.8(3)
C(18)-C(19)-C(32)-C(37)	-129.3(3)
C(1)-C(19)-C(32)-C(37)	51.4(4)
C(37)-C(32)-C(33)-C(34)	-0.5(5)
C(19)-C(32)-C(33)-C(34)	179.7(3)
C(32)-C(33)-C(34)-C(35)	-0.2(5)
C(33)-C(34)-C(35)-C(36)	0.5(5)
C(34)-C(35)-C(36)-C(37)	0.0(5)
C(35)-C(36)-C(37)-C(32)	-0.7(5)
C(33)-C(32)-C(37)-C(36)	1.0(5)
C(19)-C(32)-C(37)-C(36)	-179.2(3)
C(43)-C(38)-C(39)-C(40)	0.4(4)
P(1)-C(38)-C(39)-C(40)	-178.1(2)
C(38)-C(39)-C(40)-C(41)	-0.6(4)
C(39)-C(40)-C(41)-C(42)	0.2(4)
C(40)-C(41)-C(42)-C(43)	0.5(5)
C(41)-C(42)-C(43)-C(38)	-0.7(4)
C(39)-C(38)-C(43)-C(42)	0.3(4)
P(1)-C(38)-C(43)-C(42)	178.8(2)
C(5)-C(4)-N(1)-C(1)	-172.9(3)
C(3)-C(4)-N(1)-C(1)	1.7(3)
C(5)-C(4)-N(1)-P(1)	25.8(4)
C(3)-C(4)-N(1)-P(1)	-159.5(2)
C(19)-C(1)-N(1)-C(4)	-177.2(3)

C(2)-C(1)-N(1)-C(4)	-0.2(3)
C(19)-C(1)-N(1)-P(1)	-17.1(5)
C(2)-C(1)-N(1)-P(1)	159.9(2)
C(5)-C(6)-N(2)-C(9)	-172.0(3)
C(7)-C(6)-N(2)-C(9)	5.7(3)
C(5)-C(6)-N(2)-P(1)	-6.1(4)
C(7)-C(6)-N(2)-P(1)	171.6(2)
C(10)-C(9)-N(2)-C(6)	170.1(3)
C(8)-C(9)-N(2)-C(6)	-5.6(3)
C(10)-C(9)-N(2)-P(1)	4.7(5)
C(8)-C(9)-N(2)-P(1)	-170.9(2)
C(15)-C(14)-N(3)-C(11)	-175.4(3)
C(13)-C(14)-N(3)-C(11)	1.4(3)
C(15)-C(14)-N(3)-P(1)	14.3(3)
C(13)-C(14)-N(3)-P(1)	-168.9(2)
C(10)-C(11)-N(3)-C(14)	173.5(3)
C(12)-C(11)-N(3)-C(14)	-2.1(3)
C(10)-C(11)-N(3)-P(1)	-18.9(5)
C(12)-C(11)-N(3)-P(1)	165.5(2)
C(16)-C(15)-N(4)-C(18)	-0.3(3)
C(14)-C(15)-N(4)-C(18)	174.2(3)
C(16)-C(15)-N(4)-P(1)	176.3(2)
C(14)-C(15)-N(4)-P(1)	-9.2(3)
C(19)-C(18)-N(4)-C(15)	178.6(3)
C(17)-C(18)-N(4)-C(15)	-0.4(3)
C(19)-C(18)-N(4)-P(1)	3.0(5)
C(17)-C(18)-N(4)-P(1)	-176.0(2)
C(15)-N(4)-P(1)-N(2)	82.7(3)
C(18)-N(4)-P(1)-N(2)	-102.0(4)
C(15)-N(4)-P(1)-N(3)	13.6(2)
C(18)-N(4)-P(1)-N(3)	-171.1(3)
C(15)-N(4)-P(1)-C(38)	-86.4(2)
C(18)-N(4)-P(1)-C(38)	88.8(3)
C(15)-N(4)-P(1)-N(1)	174.1(2)
C(18)-N(4)-P(1)-N(1)	-10.6(3)
C(6)-N(2)-P(1)-N(4)	115.3(3)

C(9)-N(2)-P(1)-N(4)	-82.3(4)
C(6)-N(2)-P(1)-N(3)	-176.9(3)
C(9)-N(2)-P(1)-N(3)	-14.5(3)
C(6)-N(2)-P(1)-C(38)	-75.4(3)
C(9)-N(2)-P(1)-C(38)	87.0(3)
C(6)-N(2)-P(1)-N(1)	25.1(3)
C(9)-N(2)-P(1)-N(1)	-172.5(3)
C(14)-N(3)-P(1)-N(4)	-15.6(2)
C(11)-N(3)-P(1)-N(4)	177.7(3)
C(14)-N(3)-P(1)-N(2)	-170.4(2)
C(11)-N(3)-P(1)-N(2)	22.9(3)
C(14)-N(3)-P(1)-C(38)	87.8(2)
C(11)-N(3)-P(1)-C(38)	-78.9(3)
C(14)-N(3)-P(1)-N(1)	-79.2(4)
C(11)-N(3)-P(1)-N(1)	114.1(4)
C(39)-C(38)-P(1)-N(4)	-15.4(3)
C(43)-C(38)-P(1)-N(4)	166.1(2)
C(39)-C(38)-P(1)-N(2)	169.7(2)
C(43)-C(38)-P(1)-N(2)	-8.9(3)
C(39)-C(38)-P(1)-N(3)	-100.2(2)
C(43)-C(38)-P(1)-N(3)	81.3(2)
C(39)-C(38)-P(1)-N(1)	75.0(3)
C(43)-C(38)-P(1)-N(1)	-103.6(2)
C(4)-N(1)-P(1)-N(4)	172.9(2)
C(1)-N(1)-P(1)-N(4)	16.3(3)
C(4)-N(1)-P(1)-N(2)	-34.2(3)
C(1)-N(1)-P(1)-N(2)	169.1(3)
C(4)-N(1)-P(1)-N(3)	-124.5(3)
C(1)-N(1)-P(1)-N(3)	78.8(4)
C(4)-N(1)-P(1)-C(38)	68.4(3)
C(1)-N(1)-P(1)-C(38)	-88.3(3)

Symmetry transformations used to generate equivalent atoms:

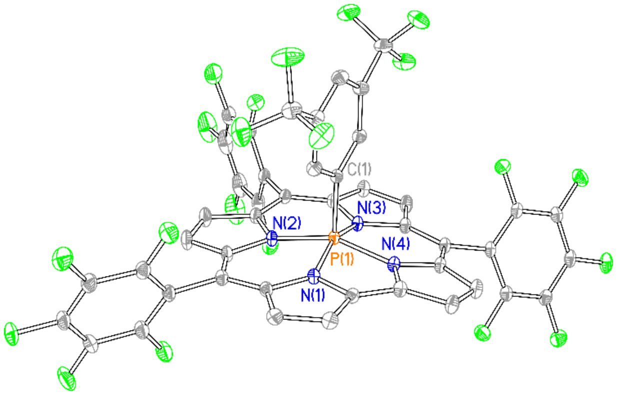

Table S7. Crystal data and structure refinement for **4$^+$**.

Identification code	cg424	
Empirical formula	C69 H11 B F41 N4 P	
Formula weight	1716.60	
Temperature	100(2) K	
Wavelength	0.71073 Å	
Crystal system	Monoclinic	
Space group	Cc	
Unit cell dimensions	a = 23.0082(11) Å	a= 90°.
	b = 18.2410(9) Å	b= 127.241(2)°.
	c = 18.4271(9) Å	g = 90°.
Volume	6156.8(5) Å^3	
Z	4	
Density (calculated)	1.852 Mg/m^3	
Absorption coefficient	0.221 mm^{-1}	
F(000)	3368	
Crystal size	0.429 x 0.426 x 0.058 mm^3	
Theta range for data collection	1.576 to 36.420°.	
Index ranges	-38<=h<=38, -30<=k<=30, -30<=l<=30	
Reflections collected	290451	
Independent reflections	30013 [R(int) = 0.0635]	
Completeness to theta = 25.242°	100.0 %	
Absorption correction	Semi-empirical from equivalents	
Refinement method	Full-matrix least-squares on F^2	
Data / restraints / parameters	30013 / 2 / 1045	
Goodness-of-fit on F^2	1.026	

Final R indices [I>2sigma(I)]	R1 = 0.0384, wR2 = 0.0901
R indices (all data)	R1 = 0.0499, wR2 = 0.0978
Absolute structure parameter	0.01(2)
Extinction coefficient	n/a
Largest diff. peak and hole	0.438 and -0.333 e.$Å^{-3}$

Table S8. Atomic coordinates (x 10^4) and equivalent isotropic displacement parameters ($Å^2$x 10^3) for **4^+.** U(eq) is defined as one third of the trace of the orthogonalized U^{ij} tensor.

	x	y	z	U(eq)
P(1)	5360(1)	2632(1)	3152(1)	11(1)
F(21)	6294(1)	557(1)	5758(1)	22(1)
F(37)	3887(1)	1910(1)	7822(1)	22(1)
F(22)	5334(1)	1200(1)	9072(1)	23(1)
F(32)	3855(1)	2062(1)	5434(1)	22(1)
F(38)	2898(1)	852(1)	7277(1)	25(1)
F(17)	3771(1)	455(1)	3350(1)	21(1)
F(20)	6130(1)	-525(1)	6619(1)	24(1)
F(31)	5041(1)	3054(1)	6138(1)	24(1)
F(27)	5998(1)	998(1)	8106(1)	23(1)
F(2)	2246(1)	1588(1)	-86(1)	31(1)
F(19)	4818(1)	-1145(1)	5847(1)	24(1)
F(40)	3819(1)	-410(1)	5941(1)	31(1)
F(16)	5119(1)	4280(1)	5443(1)	28(1)
F(11)	5927(1)	4344(1)	1201(1)	26(1)
F(39)	2871(1)	-335(1)	6357(1)	30(1)
F(18)	3627(1)	-662(1)	4198(1)	25(1)
F(3)	2560(1)	1944(1)	1219(1)	29(1)
F(36)	4447(1)	3448(1)	7976(1)	32(1)
F(10)	6516(1)	5516(1)	972(1)	30(1)
F(28)	7055(1)	896(1)	7922(1)	31(1)
F(26)	5727(1)	3414(1)	8075(1)	28(1)
F(41)	4740(1)	668(1)	6368(1)	26(1)
F(7)	7311(1)	4879(1)	4338(1)	27(1)
F(9)	7494(1)	6382(1)	2434(1)	32(1)
F(5)	4570(1)	2279(1)	-343(1)	30(1)
F(1)	2088(1)	2713(1)	109(1)	30(1)
F(4)	3553(1)	2861(1)	-1177(1)	36(1)
F(25)	6828(1)	3741(1)	9799(1)	39(1)
F(30)	6066(1)	2912(1)	5911(1)	29(1)
F(29)	7117(1)	1851(1)	6833(1)	33(1)

F(8)	7908(1)	6039(1)	4105(1)	36(1)
F(23)	6434(1)	1534(1)	10770(1)	37(1)
F(6)	4545(1)	3432(1)	-167(1)	34(1)
F(12)	3183(1)	4360(1)	2310(1)	36(1)
F(24)	7207(1)	2794(1)	11177(1)	40(1)
F(33)	2709(1)	2923(1)	4329(1)	40(1)
F(35)	3317(1)	4302(1)	6865(2)	57(1)
N(2)	5431(1)	3607(1)	3328(1)	14(1)
N(1)	6065(1)	2694(1)	3018(1)	13(1)
F(13)	2522(1)	5400(1)	2641(2)	55(1)
F(15)	4405(1)	5233(1)	5774(2)	51(1)
F(34)	2423(1)	4073(1)	5028(2)	57(1)
N(4)	5666(1)	1703(1)	3427(1)	14(1)
N(3)	4951(1)	2505(1)	3721(1)	13(1)
F(14)	3102(1)	5794(1)	4359(2)	61(1)
C(17)	5016(1)	4043(1)	3495(1)	16(1)
C(19)	4548(1)	3032(1)	3813(1)	14(1)
C(27)	6105(1)	1459(1)	3196(1)	15(1)
C(45)	5632(1)	265(1)	5356(1)	16(1)
C(47)	5670(1)	1849(1)	9202(1)	19(1)
C(9)	6328(1)	2038(1)	2938(1)	14(1)
C(24)	5540(1)	1097(1)	3774(1)	14(1)
C(57)	5510(1)	2491(1)	6576(1)	18(1)
C(13)	6242(1)	3973(1)	2907(1)	16(1)
C(10)	6782(1)	2172(1)	2683(1)	17(1)
C(22)	4841(1)	1843(1)	4039(1)	15(1)
C(11)	6813(1)	2924(1)	2621(1)	16(1)
C(8)	2548(1)	2139(1)	519(1)	21(1)
C(23)	5127(1)	1163(1)	4084(1)	14(1)
C(14)	5798(1)	4126(1)	3155(1)	16(1)
C(44)	5559(1)	-295(1)	5804(1)	17(1)
C(7)	4173(1)	2810(1)	-339(1)	20(1)
C(67)	3350(1)	205(1)	6605(1)	21(1)
C(46)	5455(1)	2298(1)	8473(1)	17(1)
C(6)	3887(1)	2371(1)	1714(1)	15(1)
C(18)	4569(1)	3771(1)	3691(1)	15(1)

C(5)	3294(1)	2329(1)	798(1)	16(1)
C(66)	3372(1)	807(1)	7080(1)	19(1)
C(34)	4169(1)	4292(1)	3868(1)	18(1)
C(42)	4279(1)	-361(1)	4572(1)	17(1)
C(41)	4363(1)	208(1)	4146(1)	16(1)
C(1)	4572(1)	2568(1)	1962(1)	13(1)
C(26)	6243(1)	705(1)	3375(1)	18(1)
C(12)	6374(1)	3250(1)	2834(1)	14(1)
C(3)	4052(1)	2669(1)	367(1)	16(1)
C(2)	4650(1)	2723(1)	1282(1)	15(1)
C(43)	4884(1)	-608(1)	5412(1)	18(1)
C(4)	3371(1)	2473(1)	116(1)	18(1)
C(29)	7104(1)	5029(1)	3504(1)	20(1)
C(51)	5873(1)	2937(1)	8727(1)	21(1)
C(59)	3746(1)	2617(1)	5824(1)	20(1)
C(39)	4466(1)	4525(1)	4745(2)	22(1)
C(33)	6411(1)	4759(1)	1925(1)	18(1)
C(53)	5991(1)	1486(1)	7551(1)	18(1)
C(69)	4325(1)	739(1)	6658(1)	19(1)
C(20)	4187(1)	2700(1)	4131(1)	18(1)
C(32)	6706(1)	5364(1)	1793(1)	22(1)
C(40)	5037(1)	535(1)	4520(1)	14(1)
C(65)	3887(1)	1347(1)	7349(1)	17(1)
C(55)	6582(1)	1904(1)	6917(2)	23(1)
C(25)	5893(1)	481(1)	3734(1)	18(1)
C(31)	7202(1)	5799(1)	2534(2)	23(1)
C(52)	5444(1)	2016(1)	7113(1)	16(1)
C(68)	3832(1)	172(1)	6399(1)	22(1)
C(56)	6051(1)	2440(1)	6459(1)	22(1)
C(21)	4381(1)	1980(1)	4288(1)	18(1)
C(30)	7410(1)	5630(1)	3392(2)	23(1)
C(63)	4053(1)	3295(1)	7078(2)	25(1)
C(48)	6246(1)	2005(1)	10100(1)	24(1)
C(15)	5607(1)	4846(1)	3237(2)	22(1)
C(64)	4385(1)	1348(1)	7152(1)	16(1)
C(16)	5132(1)	4796(1)	3438(2)	22(1)

C(54)	6546(1)	1421(1)	7468(1)	22(1)
C(49)	6643(1)	2640(1)	10312(1)	27(1)
C(28)	6593(1)	4585(1)	2773(1)	16(1)
C(58)	4232(1)	2714(1)	6763(1)	18(1)
C(50)	6449(1)	3117(1)	9614(2)	26(1)
C(60)	3146(1)	3056(1)	5232(2)	28(1)
C(35)	3497(1)	4581(1)	3159(2)	26(1)
C(62)	3454(2)	3750(1)	6510(2)	35(1)
C(38)	4109(2)	5019(1)	4922(2)	34(1)
C(37)	3448(2)	5304(1)	4200(3)	40(1)
C(36)	3148(1)	5100(1)	3327(2)	37(1)
C(61)	3000(1)	3636(2)	5578(2)	36(1)
B(1)	4877(1)	2094(1)	7376(1)	16(1)

Table S9. Bond lengths [Å] and angles [°] for **4⁺**.

P(1)-N(4)	1.7860(14)
P(1)-N(1)	1.7873(15)
P(1)-N(3)	1.7977(14)
P(1)-N(2)	1.7981(14)
P(1)-C(1)	1.8168(16)
F(21)-C(45)	1.336(2)
F(37)-C(65)	1.347(2)
F(22)-C(47)	1.353(2)
F(32)-C(59)	1.349(2)
F(38)-C(66)	1.343(2)
F(17)-C(41)	1.339(2)
F(20)-C(44)	1.330(2)
F(31)-C(57)	1.348(2)
F(27)-C(53)	1.349(2)
F(2)-C(8)	1.340(2)
F(19)-C(43)	1.331(2)
F(40)-C(68)	1.345(2)
F(16)-C(39)	1.332(3)
F(11)-C(33)	1.339(2)

F(39)-C(67)	1.335(2)
F(18)-C(42)	1.332(2)
F(3)-C(8)	1.324(2)
F(36)-C(63)	1.350(3)
F(10)-C(32)	1.326(2)
F(28)-C(54)	1.344(2)
F(26)-C(51)	1.350(2)
F(41)-C(69)	1.355(2)
F(7)-C(29)	1.333(2)
F(9)-C(31)	1.329(2)
F(5)-C(7)	1.334(2)
F(1)-C(8)	1.348(2)
F(4)-C(7)	1.327(2)
F(25)-C(50)	1.347(2)
F(30)-C(56)	1.344(2)
F(29)-C(55)	1.334(2)
F(8)-C(30)	1.331(2)
F(23)-C(48)	1.342(3)
F(6)-C(7)	1.340(2)
F(12)-C(35)	1.327(3)
F(24)-C(49)	1.341(2)
F(33)-C(60)	1.347(3)
F(35)-C(62)	1.339(3)
N(2)-C(17)	1.413(2)
N(2)-C(14)	1.426(2)
N(1)-C(9)	1.387(2)
N(1)-C(12)	1.392(2)
F(13)-C(36)	1.329(3)
F(15)-C(38)	1.337(3)
F(34)-C(61)	1.340(3)
N(4)-C(27)	1.385(2)
N(4)-C(24)	1.393(2)
N(3)-C(19)	1.415(2)
N(3)-C(22)	1.429(2)
F(14)-C(37)	1.342(3)
C(17)-C(18)	1.371(2)

C(17)-C(16)	1.416(2)
C(19)-C(18)	1.374(2)
C(19)-C(20)	1.409(2)
C(27)-C(9)	1.381(2)
C(27)-C(26)	1.405(2)
C(45)-C(44)	1.385(2)
C(45)-C(40)	1.392(2)
C(47)-C(46)	1.384(3)
C(47)-C(48)	1.387(3)
C(9)-C(10)	1.401(2)
C(24)-C(23)	1.378(2)
C(24)-C(25)	1.415(2)
C(57)-C(56)	1.388(3)
C(57)-C(52)	1.391(2)
C(13)-C(14)	1.377(2)
C(13)-C(12)	1.377(2)
C(13)-C(28)	1.484(2)
C(10)-C(11)	1.383(2)
C(10)-H(1)	0.9500
C(22)-C(23)	1.383(2)
C(22)-C(21)	1.409(2)
C(11)-C(12)	1.417(2)
C(11)-H(2)	0.9500
C(8)-C(5)	1.505(3)
C(23)-C(40)	1.485(2)
C(14)-C(15)	1.422(2)
C(44)-C(43)	1.380(3)
C(7)-C(3)	1.507(2)
C(67)-C(68)	1.373(3)
C(67)-C(66)	1.386(3)
C(46)-C(51)	1.399(3)
C(46)-B(1)	1.656(3)
C(6)-C(5)	1.389(2)
C(6)-C(1)	1.395(2)
C(6)-H(11)	0.9500
C(18)-C(34)	1.490(2)

C(5)-C(4)	1.395(3)
C(66)-C(65)	1.382(3)
C(34)-C(39)	1.386(3)
C(34)-C(35)	1.390(3)
C(42)-C(41)	1.385(2)
C(42)-C(43)	1.389(3)
C(41)-C(40)	1.393(2)
C(1)-C(2)	1.397(2)
C(26)-C(25)	1.377(3)
C(26)-H(8)	0.9500
C(3)-C(4)	1.386(3)
C(3)-C(2)	1.393(2)
C(2)-H(9)	0.9500
C(4)-H(10)	0.9500
C(29)-C(30)	1.385(3)
C(29)-C(28)	1.391(3)
C(51)-C(50)	1.383(3)
C(59)-C(60)	1.384(3)
C(59)-C(58)	1.392(3)
C(39)-C(38)	1.385(3)
C(33)-C(28)	1.388(3)
C(33)-C(32)	1.393(3)
C(53)-C(54)	1.379(3)
C(53)-C(52)	1.394(3)
C(69)-C(68)	1.389(3)
C(69)-C(64)	1.390(2)
C(20)-C(21)	1.360(2)
C(20)-H(5)	0.9500
C(32)-C(31)	1.381(3)
C(65)-C(64)	1.395(2)
C(55)-C(56)	1.383(3)
C(55)-C(54)	1.384(3)
C(25)-H(7)	0.9500
C(31)-C(30)	1.383(3)
C(52)-B(1)	1.650(3)
C(21)-H(6)	0.9500

C(63)-C(58)	1.385(3)
C(63)-C(62)	1.391(3)
C(48)-C(49)	1.376(3)
C(15)-C(16)	1.353(3)
C(15)-H(3)	0.9500
C(64)-B(1)	1.654(3)
C(16)-H(4)	0.9500
C(49)-C(50)	1.384(3)
C(58)-B(1)	1.652(3)
C(60)-C(61)	1.378(4)
C(35)-C(36)	1.391(3)
C(62)-C(61)	1.382(4)
C(38)-C(37)	1.379(4)
C(37)-C(36)	1.364(5)
N(4)-P(1)-N(1)	82.15(7)
N(4)-P(1)-N(3)	88.21(6)
N(1)-P(1)-N(3)	158.21(7)
N(4)-P(1)-N(2)	155.48(7)
N(1)-P(1)-N(2)	88.35(7)
N(3)-P(1)-N(2)	92.57(7)
N(4)-P(1)-C(1)	102.47(7)
N(1)-P(1)-C(1)	99.40(7)
N(3)-P(1)-C(1)	101.75(7)
N(2)-P(1)-C(1)	101.35(7)
C(17)-N(2)-C(14)	104.19(13)
C(17)-N(2)-P(1)	126.60(11)
C(14)-N(2)-P(1)	128.14(12)
C(9)-N(1)-C(12)	106.40(14)
C(9)-N(1)-P(1)	116.87(11)
C(12)-N(1)-P(1)	135.95(12)
C(27)-N(4)-C(24)	106.14(13)
C(27)-N(4)-P(1)	117.00(11)
C(24)-N(4)-P(1)	136.66(12)
C(19)-N(3)-C(22)	103.55(13)
C(19)-N(3)-P(1)	126.51(11)
C(22)-N(3)-P(1)	129.15(11)

C(18)-C(17)-N(2)	124.55(15)
C(18)-C(17)-C(16)	125.23(16)
N(2)-C(17)-C(16)	110.22(15)
C(18)-C(19)-C(20)	125.30(15)
C(18)-C(19)-N(3)	123.81(15)
C(20)-C(19)-N(3)	110.61(14)
C(9)-C(27)-N(4)	110.63(14)
C(9)-C(27)-C(26)	138.70(16)
N(4)-C(27)-C(26)	110.33(14)
F(21)-C(45)-C(44)	118.17(16)
F(21)-C(45)-C(40)	120.00(15)
C(44)-C(45)-C(40)	121.82(16)
F(22)-C(47)-C(46)	121.04(16)
F(22)-C(47)-C(48)	114.97(17)
C(46)-C(47)-C(48)	123.95(17)
C(27)-C(9)-N(1)	110.19(14)
C(27)-C(9)-C(10)	139.24(16)
N(1)-C(9)-C(10)	110.35(14)
C(23)-C(24)-N(4)	120.53(14)
C(23)-C(24)-C(25)	130.74(15)
N(4)-C(24)-C(25)	108.73(14)
F(31)-C(57)-C(56)	115.24(16)
F(31)-C(57)-C(52)	120.50(16)
C(56)-C(57)-C(52)	124.26(17)
C(14)-C(13)-C(12)	118.52(15)
C(14)-C(13)-C(28)	119.35(15)
C(12)-C(13)-C(28)	122.06(16)
C(11)-C(10)-C(9)	106.61(15)
C(11)-C(10)-H(1)	126.7
C(9)-C(10)-H(1)	126.7
C(23)-C(22)-C(21)	124.35(15)
C(23)-C(22)-N(3)	125.99(15)
C(21)-C(22)-N(3)	109.65(14)
C(10)-C(11)-C(12)	108.20(15)
C(10)-C(11)-H(2)	125.9
C(12)-C(11)-H(2)	125.9

F(3)-C(8)-F(2)	107.62(17)
F(3)-C(8)-F(1)	107.54(17)
F(2)-C(8)-F(1)	106.23(16)
F(3)-C(8)-C(5)	112.54(15)
F(2)-C(8)-C(5)	111.56(16)
F(1)-C(8)-C(5)	111.05(16)
C(24)-C(23)-C(22)	118.36(14)
C(24)-C(23)-C(40)	121.00(14)
C(22)-C(23)-C(40)	120.47(15)
C(13)-C(14)-C(15)	124.10(15)
C(13)-C(14)-N(2)	126.73(15)
C(15)-C(14)-N(2)	109.11(15)
F(20)-C(44)-C(43)	119.25(16)
F(20)-C(44)-C(45)	120.88(17)
C(43)-C(44)-C(45)	119.85(16)
F(4)-C(7)-F(5)	107.55(16)
F(4)-C(7)-F(6)	107.34(17)
F(5)-C(7)-F(6)	105.95(17)
F(4)-C(7)-C(3)	112.77(16)
F(5)-C(7)-C(3)	111.44(16)
F(6)-C(7)-C(3)	111.43(15)
F(39)-C(67)-C(68)	120.65(17)
F(39)-C(67)-C(66)	120.51(18)
C(68)-C(67)-C(66)	118.84(17)
C(47)-C(46)-C(51)	113.54(17)
C(47)-C(46)-B(1)	127.07(15)
C(51)-C(46)-B(1)	118.51(15)
C(5)-C(6)-C(1)	119.66(15)
C(5)-C(6)-H(11)	120.2
C(1)-C(6)-H(11)	120.2
C(17)-C(18)-C(19)	120.33(15)
C(17)-C(18)-C(34)	119.15(14)
C(19)-C(18)-C(34)	120.20(15)
C(6)-C(5)-C(4)	121.23(16)
C(6)-C(5)-C(8)	120.39(16)
C(4)-C(5)-C(8)	118.37(16)

F(38)-C(66)-C(65)	121.25(17)
F(38)-C(66)-C(67)	119.60(17)
C(65)-C(66)-C(67)	119.15(17)
C(39)-C(34)-C(35)	117.58(18)
C(39)-C(34)-C(18)	121.10(17)
C(35)-C(34)-C(18)	121.29(18)
F(18)-C(42)-C(41)	121.03(17)
F(18)-C(42)-C(43)	119.97(16)
C(41)-C(42)-C(43)	118.99(16)
F(17)-C(41)-C(42)	118.28(16)
F(17)-C(41)-C(40)	119.27(15)
C(42)-C(41)-C(40)	122.45(16)
C(6)-C(1)-C(2)	119.44(15)
C(6)-C(1)-P(1)	121.02(12)
C(2)-C(1)-P(1)	119.54(13)
C(25)-C(26)-C(27)	106.67(15)
C(25)-C(26)-H(8)	126.7
C(27)-C(26)-H(8)	126.7
C(13)-C(12)-N(1)	119.97(15)
C(13)-C(12)-C(11)	131.59(16)
N(1)-C(12)-C(11)	108.41(14)
C(4)-C(3)-C(2)	120.71(15)
C(4)-C(3)-C(7)	121.19(16)
C(2)-C(3)-C(7)	118.08(16)
C(3)-C(2)-C(1)	120.17(16)
C(3)-C(2)-H(9)	119.9
C(1)-C(2)-H(9)	119.9
F(19)-C(43)-C(44)	119.52(17)
F(19)-C(43)-C(42)	120.42(17)
C(44)-C(43)-C(42)	120.05(15)
C(3)-C(4)-C(5)	118.79(16)
C(3)-C(4)-H(10)	120.6
C(5)-C(4)-H(10)	120.6
F(7)-C(29)-C(30)	118.34(17)
F(7)-C(29)-C(28)	120.07(16)
C(30)-C(29)-C(28)	121.59(18)

F(26)-C(51)-C(50)	116.31(17)
F(26)-C(51)-C(46)	119.23(17)
C(50)-C(51)-C(46)	124.45(18)
F(32)-C(59)-C(60)	115.69(18)
F(32)-C(59)-C(58)	119.34(17)
C(60)-C(59)-C(58)	124.97(19)
F(16)-C(39)-C(38)	118.3(2)
F(16)-C(39)-C(34)	119.72(17)
C(38)-C(39)-C(34)	122.0(2)
F(11)-C(33)-C(28)	119.66(16)
F(11)-C(33)-C(32)	118.29(17)
C(28)-C(33)-C(32)	122.04(18)
F(27)-C(53)-C(54)	116.18(17)
F(27)-C(53)-C(52)	119.22(16)
C(54)-C(53)-C(52)	124.59(17)
F(41)-C(69)-C(68)	114.75(15)
F(41)-C(69)-C(64)	121.35(16)
C(68)-C(69)-C(64)	123.88(17)
C(21)-C(20)-C(19)	107.62(15)
C(21)-C(20)-H(5)	126.2
C(19)-C(20)-H(5)	126.2
F(10)-C(32)-C(31)	120.83(17)
F(10)-C(32)-C(33)	120.37(19)
C(31)-C(32)-C(33)	118.80(18)
C(45)-C(40)-C(41)	116.83(15)
C(45)-C(40)-C(23)	120.39(15)
C(41)-C(40)-C(23)	122.73(15)
F(37)-C(65)-C(66)	116.02(16)
F(37)-C(65)-C(64)	119.31(15)
C(66)-C(65)-C(64)	124.67(16)
F(29)-C(55)-C(56)	121.15(19)
F(29)-C(55)-C(54)	120.53(19)
C(56)-C(55)-C(54)	118.32(18)
C(26)-C(25)-C(24)	108.12(15)
C(26)-C(25)-H(7)	125.9
C(24)-C(25)-H(7)	125.9

F(9)-C(31)-C(32)	120.53(19)
F(9)-C(31)-C(30)	118.85(19)
C(32)-C(31)-C(30)	120.62(17)
C(57)-C(52)-C(53)	113.22(16)
C(57)-C(52)-B(1)	126.99(15)
C(53)-C(52)-B(1)	119.19(15)
F(40)-C(68)-C(67)	119.63(17)
F(40)-C(68)-C(69)	120.33(18)
C(67)-C(68)-C(69)	120.04(17)
F(30)-C(56)-C(55)	119.73(18)
F(30)-C(56)-C(57)	120.49(18)
C(55)-C(56)-C(57)	119.78(18)
C(20)-C(21)-C(22)	108.49(15)
C(20)-C(21)-H(6)	125.8
C(22)-C(21)-H(6)	125.8
F(8)-C(30)-C(31)	119.96(17)
F(8)-C(30)-C(29)	120.53(19)
C(31)-C(30)-C(29)	119.50(19)
F(36)-C(63)-C(58)	121.46(18)
F(36)-C(63)-C(62)	115.04(19)
C(58)-C(63)-C(62)	123.5(2)
F(23)-C(48)-C(49)	119.25(19)
F(23)-C(48)-C(47)	120.59(18)
C(49)-C(48)-C(47)	120.14(19)
C(16)-C(15)-C(14)	108.56(15)
C(16)-C(15)-H(3)	125.7
C(14)-C(15)-H(3)	125.7
C(69)-C(64)-C(65)	113.38(15)
C(69)-C(64)-B(1)	127.11(16)
C(65)-C(64)-B(1)	118.80(15)
C(15)-C(16)-C(17)	107.91(16)
C(15)-C(16)-H(4)	126.0
C(17)-C(16)-H(4)	126.0
F(28)-C(54)-C(53)	120.59(19)
F(28)-C(54)-C(55)	119.61(18)
C(53)-C(54)-C(55)	119.80(18)

F(24)-C(49)-C(48)	120.8(2)
F(24)-C(49)-C(50)	120.6(2)
C(48)-C(49)-C(50)	118.65(19)
C(33)-C(28)-C(29)	117.42(16)
C(33)-C(28)-C(13)	122.39(16)
C(29)-C(28)-C(13)	120.15(16)
C(63)-C(58)-C(59)	113.57(18)
C(63)-C(58)-B(1)	127.61(17)
C(59)-C(58)-B(1)	118.38(15)
F(25)-C(50)-C(51)	120.7(2)
F(25)-C(50)-C(49)	120.03(19)
C(51)-C(50)-C(49)	119.27(18)
F(33)-C(60)-C(61)	120.1(2)
F(33)-C(60)-C(59)	120.8(2)
C(61)-C(60)-C(59)	119.1(2)
F(12)-C(35)-C(34)	119.69(19)
F(12)-C(35)-C(36)	119.5(2)
C(34)-C(35)-C(36)	120.8(2)
F(35)-C(62)-C(61)	119.7(2)
F(35)-C(62)-C(63)	120.0(2)
C(61)-C(62)-C(63)	120.3(2)
F(15)-C(38)-C(37)	120.3(2)
F(15)-C(38)-C(39)	120.9(3)
C(37)-C(38)-C(39)	118.8(3)
F(14)-C(37)-C(36)	119.7(3)
F(14)-C(37)-C(38)	119.5(3)
C(36)-C(37)-C(38)	120.7(2)
F(13)-C(36)-C(37)	119.6(2)
F(13)-C(36)-C(35)	120.5(3)
C(37)-C(36)-C(35)	120.0(2)
F(34)-C(61)-C(60)	121.0(2)
F(34)-C(61)-C(62)	120.5(2)
C(60)-C(61)-C(62)	118.5(2)
C(52)-B(1)-C(58)	113.64(14)
C(52)-B(1)-C(64)	113.50(13)
C(58)-B(1)-C(64)	101.41(14)

C(52)-B(1)-C(46)	100.63(14)
C(58)-B(1)-C(46)	113.94(14)
C(64)-B(1)-C(46)	114.33(14)

Symmetry transformations used to generate equivalent atoms:

Table S10. Anisotropic displacement parameters ($Å^2 x 10^3$) for **4^+.** The anisotropic displacement factor exponent takes the form: $-2p^2[h^2 a^{*2}U^{11} + ... + 2 h k a^* b^* U^{12}]$

	U^{11}	U^{22}	U^{33}	U^{23}	U^{13}	U^{12}
P(1)	14(1)	10(1)	11(1)	0(1)	8(1)	-1(1)
F(21)	21(1)	23(1)	19(1)	1(1)	10(1)	-4(1)
F(37)	30(1)	19(1)	24(1)	-6(1)	21(1)	-2(1)
F(22)	31(1)	18(1)	20(1)	0(1)	15(1)	-7(1)
F(32)	25(1)	26(1)	18(1)	-3(1)	14(1)	-1(1)
F(38)	26(1)	27(1)	30(1)	-1(1)	22(1)	-3(1)
F(17)	20(1)	20(1)	18(1)	3(1)	9(1)	0(1)
F(20)	28(1)	24(1)	16(1)	6(1)	11(1)	2(1)
F(31)	24(1)	22(1)	26(1)	9(1)	16(1)	3(1)
F(27)	32(1)	15(1)	26(1)	5(1)	19(1)	3(1)
F(2)	24(1)	36(1)	25(1)	-8(1)	11(1)	-11(1)
F(19)	38(1)	16(1)	27(1)	5(1)	23(1)	-2(1)
F(40)	38(1)	21(1)	41(1)	-18(1)	28(1)	-11(1)
F(16)	36(1)	22(1)	25(1)	0(1)	18(1)	1(1)
F(11)	30(1)	27(1)	20(1)	-1(1)	15(1)	-5(1)
F(39)	32(1)	24(1)	37(1)	-10(1)	23(1)	-14(1)
F(18)	25(1)	24(1)	28(1)	-1(1)	16(1)	-8(1)
F(3)	21(1)	46(1)	21(1)	5(1)	13(1)	-3(1)
F(36)	43(1)	23(1)	29(1)	-8(1)	21(1)	4(1)
F(10)	33(1)	37(1)	29(1)	12(1)	23(1)	1(1)
F(28)	29(1)	24(1)	40(1)	2(1)	21(1)	8(1)
F(26)	42(1)	14(1)	26(1)	-1(1)	19(1)	-8(1)
F(41)	33(1)	24(1)	35(1)	-14(1)	28(1)	-9(1)
F(7)	32(1)	27(1)	20(1)	-2(1)	14(1)	-11(1)

F(9)	31(1)	25(1)	45(1)	11(1)	25(1)	-6(1)
F(5)	41(1)	34(1)	29(1)	6(1)	28(1)	12(1)
F(1)	19(1)	39(1)	28(1)	7(1)	11(1)	8(1)
F(4)	26(1)	69(1)	14(1)	8(1)	12(1)	7(1)
F(25)	47(1)	29(1)	32(1)	-12(1)	20(1)	-22(1)
F(30)	31(1)	33(1)	32(1)	6(1)	23(1)	-4(1)
F(29)	30(1)	35(1)	47(1)	-4(1)	30(1)	-3(1)
F(8)	36(1)	30(1)	35(1)	-7(1)	18(1)	-20(1)
F(23)	43(1)	42(1)	17(1)	4(1)	14(1)	-10(1)
F(6)	53(1)	30(1)	32(1)	0(1)	33(1)	-8(1)
F(12)	26(1)	27(1)	34(1)	7(1)	7(1)	-1(1)
F(24)	39(1)	50(1)	19(1)	-12(1)	12(1)	-19(1)
F(33)	31(1)	51(1)	22(1)	7(1)	8(1)	9(1)
F(35)	61(1)	37(1)	60(1)	-7(1)	30(1)	25(1)
N(2)	18(1)	9(1)	18(1)	0(1)	12(1)	-1(1)
N(1)	16(1)	11(1)	15(1)	0(1)	11(1)	-1(1)
F(13)	23(1)	28(1)	99(2)	16(1)	28(1)	10(1)
F(15)	85(1)	36(1)	68(1)	-20(1)	66(1)	-10(1)
F(34)	47(1)	49(1)	52(1)	12(1)	18(1)	29(1)
N(4)	18(1)	10(1)	15(1)	1(1)	11(1)	1(1)
N(3)	18(1)	10(1)	14(1)	0(1)	12(1)	0(1)
F(14)	66(1)	26(1)	133(2)	-10(1)	83(2)	3(1)
C(17)	19(1)	12(1)	22(1)	1(1)	14(1)	0(1)
C(19)	17(1)	12(1)	15(1)	0(1)	11(1)	0(1)
C(27)	19(1)	13(1)	16(1)	-1(1)	12(1)	0(1)
C(45)	20(1)	14(1)	17(1)	0(1)	12(1)	-1(1)
C(47)	24(1)	16(1)	17(1)	-2(1)	13(1)	-4(1)
C(9)	16(1)	13(1)	13(1)	-1(1)	9(1)	1(1)
C(24)	20(1)	10(1)	15(1)	1(1)	12(1)	0(1)
C(57)	20(1)	18(1)	18(1)	1(1)	12(1)	-2(1)
C(13)	18(1)	13(1)	17(1)	2(1)	11(1)	-1(1)
C(10)	19(1)	18(1)	17(1)	0(1)	13(1)	1(1)
C(22)	21(1)	11(1)	15(1)	0(1)	13(1)	-1(1)
C(11)	18(1)	18(1)	16(1)	0(1)	12(1)	-1(1)
C(8)	16(1)	28(1)	15(1)	2(1)	8(1)	0(1)
C(23)	20(1)	11(1)	14(1)	0(1)	11(1)	-1(1)

C(14)	19(1)	11(1)	21(1)	1(1)	13(1)	-2(1)
C(44)	24(1)	15(1)	14(1)	2(1)	12(1)	1(1)
C(7)	22(1)	26(1)	14(1)	1(1)	12(1)	1(1)
C(67)	24(1)	18(1)	22(1)	-4(1)	14(1)	-7(1)
C(46)	23(1)	13(1)	17(1)	-2(1)	13(1)	-2(1)
C(6)	17(1)	15(1)	12(1)	1(1)	9(1)	1(1)
C(18)	17(1)	12(1)	18(1)	1(1)	12(1)	1(1)
C(5)	16(1)	19(1)	13(1)	0(1)	8(1)	0(1)
C(66)	22(1)	20(1)	19(1)	0(1)	14(1)	-2(1)
C(34)	19(1)	11(1)	28(1)	2(1)	16(1)	1(1)
C(42)	22(1)	14(1)	19(1)	-1(1)	14(1)	-3(1)
C(41)	21(1)	12(1)	17(1)	1(1)	12(1)	0(1)
C(1)	16(1)	12(1)	11(1)	0(1)	8(1)	0(1)
C(26)	25(1)	13(1)	21(1)	1(1)	16(1)	2(1)
C(12)	16(1)	14(1)	14(1)	1(1)	9(1)	-2(1)
C(3)	22(1)	17(1)	13(1)	1(1)	12(1)	2(1)
C(2)	17(1)	17(1)	13(1)	1(1)	10(1)	0(1)
C(43)	28(1)	12(1)	19(1)	1(1)	16(1)	-1(1)
C(4)	18(1)	20(1)	12(1)	0(1)	8(1)	1(1)
C(29)	22(1)	18(1)	22(1)	1(1)	14(1)	-4(1)
C(51)	30(1)	14(1)	20(1)	-3(1)	16(1)	-5(1)
C(59)	22(1)	20(1)	20(1)	2(1)	14(1)	0(1)
C(39)	30(1)	14(1)	33(1)	-1(1)	25(1)	0(1)
C(33)	21(1)	18(1)	21(1)	3(1)	14(1)	0(1)
C(53)	24(1)	14(1)	19(1)	-1(1)	13(1)	-1(1)
C(69)	25(1)	16(1)	22(1)	-6(1)	17(1)	-4(1)
C(20)	24(1)	14(1)	22(1)	1(1)	17(1)	1(1)
C(32)	23(1)	22(1)	26(1)	7(1)	18(1)	1(1)
C(40)	22(1)	10(1)	15(1)	0(1)	13(1)	-1(1)
C(65)	23(1)	15(1)	16(1)	-2(1)	13(1)	-2(1)
C(55)	22(1)	24(1)	27(1)	-6(1)	17(1)	-4(1)
C(25)	27(1)	12(1)	22(1)	1(1)	18(1)	1(1)
C(31)	22(1)	19(1)	34(1)	7(1)	19(1)	-2(1)
C(52)	20(1)	13(1)	16(1)	-1(1)	12(1)	-2(1)
C(68)	26(1)	17(1)	23(1)	-7(1)	16(1)	-5(1)
C(56)	23(1)	23(1)	22(1)	-1(1)	15(1)	-5(1)

C(21)	28(1)	14(1)	23(1)	1(1)	21(1)	0(1)
C(30)	22(1)	20(1)	27(1)	0(1)	14(1)	-6(1)
C(63)	31(1)	18(1)	27(1)	-2(1)	18(1)	3(1)
C(48)	29(1)	28(1)	16(1)	-2(1)	13(1)	-6(1)
C(15)	28(1)	10(1)	34(1)	1(1)	23(1)	-1(1)
C(64)	21(1)	14(1)	16(1)	-2(1)	12(1)	-2(1)
C(16)	28(1)	11(1)	37(1)	1(1)	24(1)	0(1)
C(54)	22(1)	18(1)	24(1)	-3(1)	14(1)	1(1)
C(49)	30(1)	31(1)	17(1)	-8(1)	12(1)	-9(1)
C(28)	18(1)	14(1)	21(1)	2(1)	14(1)	-1(1)
C(58)	22(1)	15(1)	19(1)	0(1)	14(1)	0(1)
C(50)	33(1)	22(1)	25(1)	-9(1)	17(1)	-11(1)
C(60)	26(1)	32(1)	22(1)	6(1)	12(1)	4(1)
C(35)	20(1)	16(1)	38(1)	5(1)	16(1)	2(1)
C(62)	40(1)	23(1)	41(1)	0(1)	24(1)	12(1)
C(38)	50(1)	19(1)	58(2)	-8(1)	47(1)	-4(1)
C(37)	42(1)	18(1)	87(2)	-5(1)	53(2)	0(1)
C(36)	21(1)	17(1)	71(2)	8(1)	27(1)	5(1)
C(61)	32(1)	32(1)	36(1)	8(1)	16(1)	14(1)
B(1)	22(1)	12(1)	16(1)	-2(1)	13(1)	-2(1)

Table S11. Hydrogen coordinates (x 10^4) and isotropic displacement parameters (Å^{2}x 10 ^{3}) for **4^+**.

	x	y	z	U(eq)
H(1)	7022	1815	2575	20
H(2)	7081	3179	2462	20
H(11)	3827	2266	2170	18
H(8)	6524	407	3269	22
H(9)	5112	2866	1446	18
H(10)	2963	2437	-509	21
H(5)	3866	2937	4219	21
H(7)	5890	-3	3921	22

H(6)	4231	1630	4526	22
H(3)	5784	5289	3163	26
H(4)	4915	5195	3525	27

11. DFT calculations

The cationic portions of **1^+–3^+** were optimized in the gas phase at the B3LYP/def2-TZVP level as implemented in the ORCA 4.0.0 package. The cationic fragment of **4^+** was optimized using B3LYP/def2-SVP. The geometries of CF_2O and CF_3O were optimized in the gas phase at the B3LYP/def2-TZVP or def2-SVP level. All geometries were subjected to frequency calculations, and lack of any imaginary modes confirmed that the structures represented minima on their respective potential energy surfaces. The single point energies of **1^+–4^+** were recomputed at the same level using the CPCM(CH_2Cl_2) solvent correction. These energies were used in calculating FIA and GEI. Fluoride ion affinities were computed according to Christe's method, taking 208.8 kJ/mol as the experimental FIA of CF_2O.[8]

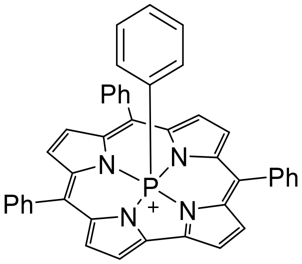

1^+

N	-0.17728	0.07314	34.25090
N	1.75970	1.50048	33.26428
N	0.68269	1.19418	31.02942
N	-1.07550	-0.07184	31.92224
C	-1.42126	-0.54055	34.60545
C	-1.38900	-0.84634	35.98511
H	-2.21230	-1.28570	36.52031
C	-0.15748	-0.53531	36.47182
H	0.21257	-0.67804	37.47230
C	0.59255	0.04992	35.42859
C	1.85463	0.59430	35.56078
C	2.60506	0.44429	36.84383
C	3.22775	-0.76684	37.15756
H	3.17635	-1.59302	36.45853
C	3.91569	-0.91520	38.35750
H	4.39207	-1.85823	38.59295
C	3.99338	0.14457	39.25328
H	4.52711	0.02675	40.18734
C	3.38079	1.35474	38.94740
H	3.43673	2.18291	39.64274

C	2.68911	1.50317	37.75043
H	2.20184	2.44359	37.52345
C	2.39138	1.30975	34.51009
C	3.60161	2.03819	34.53977
H	4.29016	2.02560	35.36747
C	3.71445	2.72189	33.36799
H	4.51806	3.36687	33.05502
C	2.61572	2.39011	32.54416
C	2.51052	2.74784	31.20930
C	3.39234	3.78876	30.61848
C	3.20912	5.12332	30.99380
H	2.45188	5.37154	31.72753
C	3.97011	6.13075	30.41536
H	3.80454	7.15987	30.70854
C	4.93420	5.81547	29.46311
H	5.52817	6.59909	29.01137
C	5.13468	4.48918	29.09524
H	5.89172	4.23822	28.36306
C	4.36812	3.47880	29.66621
H	4.53738	2.44829	29.37823
C	1.58987	2.06230	30.43532
C	1.40337	1.99526	29.03617
H	1.97594	2.55724	28.31727
C	0.40599	1.07419	28.77533
H	0.02368	0.77112	27.81440
C	-0.04865	0.61104	30.01467
C	-1.08261	-0.14561	30.54120
C	-2.18630	-0.89138	30.11284
H	-2.43256	-1.13034	29.09134
C	-2.88269	-1.25807	31.24904
H	-3.77839	-1.85399	31.29926
C	-2.19875	-0.75003	32.37574
C	-2.43021	-0.91665	33.73177
C	-3.68145	-1.57410	34.18733
C	-4.91780	-1.00458	33.86392
H	-4.94996	-0.07235	33.31405
C	-6.10175	-1.61586	34.25599
H	-7.04940	-1.15752	34.00377
C	-6.06938	-2.80726	34.97173
H	-6.99178	-3.28378	35.27734
C	-4.84643	-3.38839	35.28820
H	-4.81401	-4.32462	35.83016
C	-3.66097	-2.77722	34.90096
H	-2.71492	-3.24971	35.13257
H	-0.93759	5.19773	35.11239
C	-1.21999	4.54645	34.29512
C	-2.29004	4.87926	33.47206
H	-2.84700	5.78978	33.65092
C	-2.64391	4.03946	32.42204
H	-3.47372	4.29630	31.77641
C	-1.93283	2.86667	32.19388

H	-2.22630	2.22458	31.37434
C	-0.85266	2.52868	33.01406
C	-0.50189	3.37964	34.06707
H	0.33148	3.14183	34.71378
P	0.10754	1.00909	32.72995

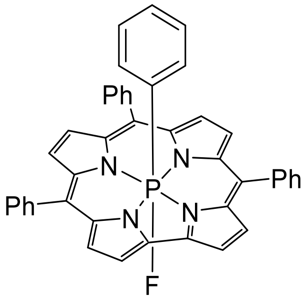

1•F

N	-0.26382	0.12957	34.31845
N	1.76191	1.65019	33.28292
N	0.59027	1.35060	30.96548
N	-1.19677	0.01636	31.87195
C	-1.45133	-0.55546	34.61336
C	-1.41632	-0.89728	35.99645
H	-2.20962	-1.40188	36.51947
C	-0.21586	-0.50795	36.50388
H	0.14501	-0.65123	37.50719
C	0.50699	0.14994	35.46556
C	1.75649	0.76168	35.58499
C	2.50726	0.63944	36.86839
C	3.71087	-0.07185	36.91811
H	4.09629	-0.52252	36.01186
C	4.40584	-0.21745	38.11359
H	5.33291	-0.77682	38.13144
C	3.90851	0.34594	39.28342
H	4.44419	0.22458	40.21675
C	2.71906	1.06550	39.24640
H	2.32726	1.51675	40.14987
C	2.02800	1.21448	38.04915
H	1.10492	1.77941	38.02732
C	2.32044	1.49054	34.53710
C	3.51207	2.27140	34.60287
H	4.14611	2.34697	35.46965
C	3.67505	2.88823	33.40012
H	4.47932	3.53949	33.10298
C	2.60351	2.49629	32.54568
C	2.50616	2.79853	31.18774
C	3.49325	3.71312	30.55304
C	3.59428	5.05135	30.95045
H	2.93875	5.42426	31.72779
C	4.51535	5.90412	30.35315
H	4.58064	6.93524	30.67887

C	5.34751	5.43962	29.33998
H	6.06148	6.10574	28.87202
C	5.25719	4.11227	28.93508
H	5.90717	3.73644	28.15429
C	4.34006	3.25786	29.53575
H	4.28892	2.22180	29.22599
C	1.52390	2.18035	30.40452
C	1.28398	2.19393	29.00271
H	1.84821	2.76739	28.28640
C	0.22420	1.34439	28.73898
H	-0.21321	1.11750	27.78042
C	-0.20315	0.83567	29.97819
C	-1.23809	0.04740	30.50523
C	-2.36637	-0.67180	30.06900
H	-2.66352	-0.83088	29.04526
C	-3.00829	-1.12948	31.20577
H	-3.90153	-1.72965	31.24727
C	-2.26904	-0.69137	32.33888
C	-2.43765	-0.94118	33.70640
C	-3.65051	-1.67570	34.15027
C	-4.92159	-1.16703	33.85848
H	-5.00420	-0.22179	33.33747
C	-6.06975	-1.85051	34.23827
H	-7.04166	-1.43311	34.00537
C	-5.97053	-3.05789	34.92061
H	-6.86402	-3.59035	35.22122
C	-4.71448	-3.57856	35.21147
H	-4.62631	-4.52550	35.72928
C	-3.56601	-2.89676	34.82876
H	-2.59351	-3.32255	35.03972
H	-1.16955	4.79447	35.46121
C	-1.34600	4.27700	34.52579
C	-2.28970	4.75821	33.62677
H	-2.85737	5.65197	33.85417
C	-2.49095	4.07728	32.43316
H	-3.21773	4.43871	31.71546
C	-1.76068	2.92664	32.14670
H	-1.95108	2.42585	31.20793
C	-0.80560	2.42777	33.03721
C	-0.61811	3.12918	34.23333
H	0.10355	2.78908	34.96140
P	0.20235	0.85985	32.68233
F	1.09576	-0.51262	32.43484

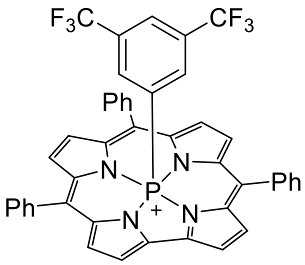

2^+

N	-0.31889	0.33775	34.93685
N	1.53340	1.21210	33.32050
N	0.14092	0.27252	31.46101
N	-1.54535	-0.48308	32.91260
C	-1.48131	-0.14794	35.62064
C	-1.21951	-0.14801	37.00778
H	-1.92442	-0.49267	37.74478
C	0.04996	0.29409	37.21489
H	0.57526	0.38451	38.15005
C	0.60931	0.62665	35.96396
C	1.84700	1.20110	35.77090
C	2.25798	1.50917	34.49186
C	3.45397	2.17942	34.15662
H	4.18205	2.50593	34.87911
C	3.49660	2.31597	32.80458
H	4.26816	2.77838	32.21338
C	2.35303	1.69744	32.25173
C	2.14127	1.50646	30.89770
C	1.06061	0.73134	30.51991
C	0.69659	0.20561	29.26179
H	1.24793	0.37445	28.35185
C	-0.43850	-0.56561	29.42208
H	-0.97145	-1.11445	28.66326
C	-0.77892	-0.50486	30.77643
C	-1.78061	-0.93195	31.62813
C	-3.00062	-1.61495	31.57511
H	-3.42791	-2.07633	30.70015
C	-3.53951	-1.56170	32.84521
H	-4.47393	-1.98450	33.17354
C	-2.63182	-0.87826	33.68527
C	-2.63685	-0.65862	35.05059
C	-2.74574	4.36237	30.33538
C	-2.21449	3.94191	31.68780
C	-2.37833	4.75397	32.80209
H	-2.88614	5.70324	32.71756
C	-1.88393	4.32754	34.02757
C	-2.04309	5.18186	35.26454
C	-1.22934	3.10617	34.14159
H	-0.85794	2.79989	35.10926
C	-1.06121	2.28961	33.02316
C	-1.56068	2.71969	31.79289
H	-1.44976	2.11236	30.90619

P	-0.19284	0.68188	33.17258
F	-2.66491	4.49941	36.25026
F	-3.31753	5.57280	30.36954
F	-3.66375	3.48519	29.88030
F	-1.75764	4.40306	29.41531
F	-2.75503	6.29053	35.02595
F	-0.84261	5.56110	35.75158
C	-3.79604	-1.05085	35.89370
C	-4.19026	-2.38895	35.98962
C	-4.51258	-0.08075	36.60122
C	-5.27867	-2.74696	36.77609
H	-3.63072	-3.15333	35.46470
C	-5.60381	-0.44129	37.38213
H	-4.22127	0.95989	36.52969
C	-5.98911	-1.77444	37.47133
H	-5.56753	-3.78760	36.84987
H	-6.15462	0.32078	37.91848
H	-6.83770	-2.05474	38.08188
C	2.73738	1.48569	36.93496
C	3.81725	0.64334	37.20989
C	2.50895	2.58932	37.75952
C	4.65215	0.89947	38.29147
H	3.99970	-0.21823	36.57913
C	3.34748	2.84576	38.83872
H	1.67915	3.25415	37.55255
C	4.41907	2.00120	39.10715
H	5.48287	0.23645	38.49697
H	3.16370	3.70727	39.46785
H	5.07041	2.20107	39.94825
C	3.08466	2.03243	29.87395
C	4.37914	1.51700	29.76042
C	2.67376	3.03573	28.99227
C	5.24673	2.00144	28.78903
H	4.70223	0.72538	30.42526
C	3.54606	3.52236	28.02524
H	1.67452	3.44575	29.07242
C	4.83292	3.00647	27.92147
H	6.24428	1.58913	28.70708
H	3.21950	4.30650	27.35428
H	5.51025	3.38454	27.16661

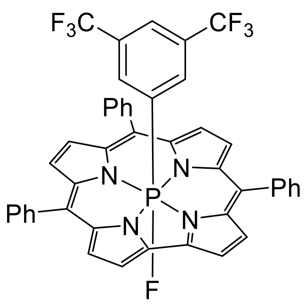

2•F

N	-0.71689	0.07432	34.53810
N	1.45562	1.20278	33.33608
N	0.15978	0.91200	31.08444
N	-1.76970	-0.05240	32.14685
C	-1.96349	-0.44887	34.91711
C	-1.90387	-0.74639	36.30876
H	-2.72628	-1.14907	36.87527
C	-0.64833	-0.46796	36.75346
H	-0.25811	-0.59899	37.74820
C	0.09992	0.04937	35.65700
C	1.41546	0.50324	35.70059
C	2.17744	0.42179	36.98345
C	3.15751	-0.55786	37.16285
H	3.34862	-1.26678	36.36653
C	3.88173	-0.63037	38.34789
H	4.63622	-1.39771	38.47035
C	3.63586	0.27623	39.37338
H	4.20018	0.22189	40.29600
C	2.66045	1.25328	39.20769
H	2.46429	1.96532	39.99977
C	1.93701	1.32522	38.02211
H	1.18424	2.09400	37.89693
C	2.03640	1.05855	34.58382
C	3.34011	1.63131	34.54576
H	4.01809	1.65678	35.38160
C	3.54610	2.11837	33.29181
H	4.43144	2.60026	32.91358
C	2.38777	1.84662	32.50828
C	2.27174	2.07478	31.13933
C	3.33757	2.81343	30.41061
C	3.58022	4.16380	30.68170
H	2.98129	4.66982	31.42865
C	4.56734	4.86063	29.99464
H	4.73667	5.90744	30.21461
C	5.32859	4.21938	29.02353
H	6.09758	4.76182	28.48792
C	5.09690	2.87672	28.74501
H	5.69027	2.36730	27.99575
C	4.10940	2.17978	29.43149
H	3.94306	1.13084	29.22039
C	1.17482	1.56523	30.43650
C	0.86225	1.55792	29.05047
H	1.46845	2.00309	28.27957
C	-0.33393	0.88405	28.88141
H	-0.85629	0.70321	27.95645
C	-0.76565	0.49781	30.16309
C	-1.88862	-0.07095	30.78287
C	-3.14886	-0.59633	30.44332
H	-3.52919	-0.73277	29.44434
C	-3.79040	-0.89175	31.63308

H	-4.77227	-1.31860	31.75405
C	-2.91746	-0.55384	32.70148
C	-3.04078	-0.72394	34.08223
C	-4.30960	-1.27447	34.64004
C	-5.44392	-0.46612	34.75039
H	-5.38914	0.57110	34.44357
C	-6.63290	-0.97824	35.25827
H	-7.50064	-0.33582	35.34357
C	-6.70688	-2.30769	35.65889
H	-7.63275	-2.70653	36.05437
C	-5.58561	-3.12283	35.54773
H	-5.63627	-4.16125	35.85123
C	-4.39623	-2.61004	35.04220
H	-3.52659	-3.24907	34.95070
C	-1.30897	4.28719	34.49562
C	-1.99671	4.93911	33.48270
H	-2.39011	5.93382	33.63201
C	-2.16121	4.28267	32.26972
C	-1.65052	3.00330	32.07696
H	-1.80173	2.53933	31.11456
C	-0.95465	2.33659	33.08702
C	-0.79565	3.00796	34.29856
H	-0.26376	2.54289	35.11518
P	-0.21463	0.59583	32.83934
F	0.45552	-0.89994	32.66668
C	-1.07024	4.96116	35.82086
C	-2.93719	4.93931	31.15797
F	-1.80153	6.08139	35.97040
F	-1.37028	4.14895	36.85906
F	0.22782	5.31356	35.97681
F	-2.87960	6.28498	31.21916
F	-2.48467	4.57576	29.94107
F	-4.24926	4.60592	31.19955

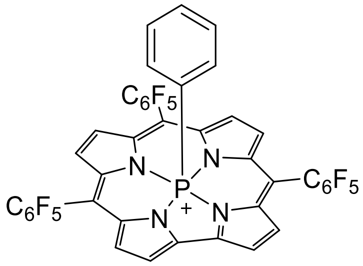

3$^+$

N	-0.18942	-0.22850	34.08899
N	1.97863	0.97415	33.23949
N	0.92547	1.03405	30.96277
N	-1.00423	-0.07885	31.71860
C	-1.47119	-0.80295	34.32927
C	-1.54360	-1.21462	35.67958
H	-2.40381	-1.68940	36.11986

C	-0.35316	-0.94615	36.27547
H	-0.05993	-1.16185	37.28869
C	0.48335	-0.33207	35.31732
C	1.76710	0.10981	35.54355
C	2.37833	-0.02848	36.89553
C	3.10324	-1.15874	37.26767
F	3.25459	-2.16444	36.40124
C	3.68450	-1.27557	38.52360
F	4.37485	-2.36290	38.85011
C	3.54096	-0.24223	39.44253
F	4.09147	-0.34343	40.64313
C	2.81991	0.89691	39.10161
F	2.68052	1.88623	39.97803
C	2.25221	0.98894	37.83925
F	1.56581	2.09526	37.52839
C	2.47948	0.71394	34.53153
C	3.79737	1.20399	34.64119
H	4.41163	1.10160	35.51937
C	4.12758	1.79124	33.46052
H	5.06334	2.25532	33.19890
C	3.03635	1.66747	32.57447
C	3.04957	2.10148	31.26124
C	4.20905	2.86602	30.73057
C	4.37485	4.21961	31.02017
F	3.47369	4.84828	31.78196
C	5.45474	4.94721	30.54096
F	5.58217	6.23745	30.83255
C	6.40782	4.31459	29.75108
F	7.44805	4.99597	29.29517
C	6.26988	2.96618	29.44247
F	7.18256	2.35908	28.69217
C	5.17805	2.26209	29.93095
F	5.07414	0.96341	29.63162
C	2.00049	1.73817	30.44372
C	1.81735	1.86115	29.04622
H	2.50752	2.34861	28.37767
C	0.64851	1.21504	28.70701
H	0.22115	1.10360	27.72426
C	0.10495	0.71933	29.90066
C	-1.02117	0.05306	30.34043
C	-2.19875	-0.49493	29.81988
H	-2.46358	-0.54299	28.77657
C	-2.93131	-0.95286	30.89557
H	-3.88714	-1.44956	30.86683
C	-2.19604	-0.69860	32.07446
C	-2.46556	-1.00870	33.39208
C	-3.78199	-1.58576	33.77404
C	-4.91674	-0.77820	33.84139
F	-4.81660	0.52856	33.57218
C	-6.16065	-1.28261	34.19135
F	-7.21900	-0.48160	34.25204

C	-6.29189	-2.63532	34.48310
F	-7.47394	-3.12898	34.82013
C	-5.18114	-3.46888	34.41976
F	-5.30462	-4.76296	34.69399
C	-3.94763	-2.93906	34.06584
F	-2.89868	-3.76464	34.00397
P	0.27952	0.78132	32.63798
C	-0.48296	2.38497	33.02094
C	0.16347	3.29188	33.86834
C	-1.73130	2.72583	32.48602
C	-0.42777	4.51166	34.17128
H	1.12606	3.06167	34.30028
C	-2.31396	3.94859	32.79144
H	-2.26262	2.04869	31.83378
C	-1.66551	4.84343	33.63417
H	0.08400	5.20203	34.82879
H	-3.27953	4.19738	32.37090
H	-2.12344	5.79487	33.87182

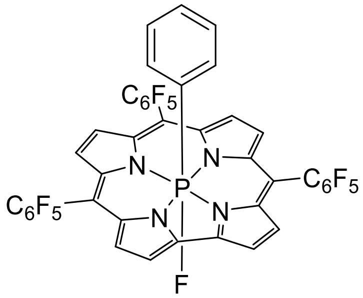

3•F

N	-0.21689	0.06977	34.32589
N	1.79013	1.55883	33.20376
N	0.52376	1.24794	30.92572
N	-1.23382	-0.06592	31.90986
C	-1.38832	-0.61115	34.66592
C	-1.30333	-0.96723	36.04372
H	-2.06631	-1.50295	36.58363
C	-0.09790	-0.54858	36.51599
H	0.29500	-0.67504	37.51084
C	0.58613	0.10173	35.44681
C	1.83111	0.72195	35.51479
C	2.56489	0.73368	36.81353
C	3.42852	-0.29142	37.19515
F	3.60634	-1.34888	36.39886
C	4.13199	-0.25510	38.39290
F	4.95948	-1.24699	38.72506
C	3.97673	0.82693	39.24837
F	4.64436	0.86883	40.39934
C	3.12233	1.86420	38.90040
F	2.97326	2.90920	39.71636
C	2.43193	1.80251	37.69871

F	1.61555	2.81883	37.39621
C	2.38701	1.40986	34.43839
C	3.61820	2.12860	34.43803
H	4.30153	2.17995	35.26879
C	3.75691	2.71270	33.21733
H	4.57916	3.31781	32.87413
C	2.63161	2.35156	32.42074
C	2.46665	2.65165	31.07140
C	3.43067	3.56478	30.40217
C	3.42404	4.93695	30.65478
F	2.53122	5.45585	31.50245
C	4.31984	5.80466	30.04575
F	4.28613	7.11148	30.31165
C	5.25492	5.30581	29.14917
F	6.12233	6.12875	28.56429
C	5.28409	3.94680	28.86750
F	6.18514	3.46379	28.00983
C	4.38032	3.09864	29.49314
F	4.45421	1.79375	29.21349
C	1.43907	2.06664	30.32844
C	1.13614	2.09850	28.93846
H	1.68485	2.65426	28.19600
C	0.04515	1.28021	28.71929
H	-0.43941	1.07609	27.77878
C	-0.33061	0.76170	29.97529
C	-1.35380	-0.00773	30.54890
C	-2.54042	-0.66782	30.16959
H	-2.90411	-0.78792	29.16245
C	-3.13759	-1.11107	31.33401
H	-4.06047	-1.66134	31.41812
C	-2.30764	-0.73275	32.42612
C	-2.41262	-0.97103	33.79726
C	-3.62442	-1.66354	34.31797
C	-4.74172	-0.94237	34.73617
F	-4.72447	0.39255	34.70152
C	-5.89566	-1.56593	35.19058
F	-6.94899	-0.84462	35.57755
C	-5.95083	-2.95215	35.23678
F	-7.05318	-3.56229	35.66634
C	-4.85433	-3.70071	34.83150
F	-4.90509	-5.03296	34.87680
C	-3.71295	-3.05294	34.37808
F	-2.67955	-3.80596	33.99120
H	-1.08644	4.75667	35.42097
C	-1.27707	4.23493	34.49123
C	-2.21437	4.72514	33.59150
H	-2.76415	5.63206	33.81009
C	-2.43792	4.03418	32.40849
H	-3.16604	4.39766	31.69367
C	-1.73073	2.86973	32.12883
H	-1.93919	2.36753	31.19578

C	-0.78113	2.36149	33.02031
C	-0.57222	3.07084	34.20723
H	0.14683	2.72893	34.93654
P	0.19637	0.78119	32.66076
F	1.07665	-0.58996	32.40327

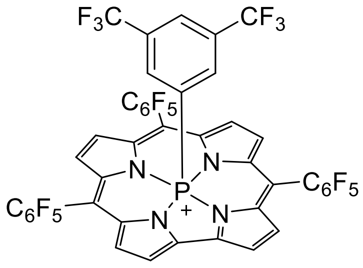

4⁺

P	8.89824	4.99291	4.81668
F	7.77923	0.70908	8.40874
F	5.31613	1.20107	4.39912
F	6.43283	-1.57229	8.94803
F	5.43201	5.45387	-0.55102
F	4.51528	-2.46300	7.23383
F	5.44908	7.58476	8.33855
F	12.00450	7.62599	1.36749
F	3.97542	-1.08339	4.94849
F	5.21287	6.60118	1.28035
F	13.79547	9.46743	0.51871
F	11.99347	9.62972	5.65120
F	14.68938	11.38825	2.22365
F	9.34982	0.97192	1.40431
F	6.77135	7.10433	-0.13423
F	7.84865	1.23591	-0.14161
F	13.78844	11.46039	4.79659
F	9.81214	2.11280	-0.37822
F	4.99889	8.46126	3.71327
N	8.75670	6.81494	4.88704
N	10.70144	5.19525	4.65513
F	2.88889	10.08192	4.23099
F	3.33419	9.19935	8.84581
N	9.41167	3.36347	5.43339
N	7.30443	4.75533	5.70087
F	2.04916	10.44281	6.79565
C	7.62860	7.58731	5.24394
C	6.29488	5.70713	5.93441
C	10.74911	3.01220	5.31864
C	6.85440	0.28340	7.54884
C	11.50485	4.08714	4.86403
C	8.71922	2.21373	5.80793
C	11.02918	7.50198	4.01726
C	12.84056	4.39113	4.53474
H	13.68690	3.71157	4.61181

C	6.71961	3.50008	6.04067
C	12.85104	5.71183	4.11286
H	13.71486	6.29613	3.80304
C	6.10455	6.08057	0.42029
C	7.35125	2.25796	6.05491
C	9.71583	7.76199	4.40155
C	6.16349	-0.89482	7.84274
C	8.84709	1.84271	0.51176
C	7.50167	5.46861	2.41888
H	7.13181	6.38569	2.87942
C	6.43336	7.07353	5.72858
C	7.05325	5.13671	1.13548
C	5.28770	7.98290	6.01287
C	4.89767	-0.64143	5.78987
C	5.60103	0.53381	5.51679
C	8.38223	4.61343	3.09505
C	10.91727	1.65066	5.63371
H	11.86121	1.10933	5.63499
C	11.52488	6.21615	4.20011
C	8.36478	3.11069	1.19110
C	8.81249	3.43008	2.47372
H	9.48380	2.73576	2.97824
C	5.17897	-1.35507	6.96080
C	7.47951	3.95967	0.51820
H	7.10927	3.69677	-0.47419
C	12.41898	9.57953	4.38995
C	4.83561	8.19294	7.32437
C	12.42674	8.56091	2.21660
C	5.12161	5.05812	6.40032
H	4.20080	5.56793	6.66943
C	13.35142	9.50580	1.76516
C	6.59377	1.01975	6.38276
C	9.65538	1.15140	5.92001
H	9.40598	0.13406	6.21215
C	13.81256	10.49414	2.64224
C	5.37935	3.71722	6.45734
H	4.71031	2.92821	6.79083
C	13.34519	10.53341	3.96104
C	9.14295	9.05915	4.45685
H	9.65852	9.96452	4.14768
C	7.88842	8.95819	4.99020
H	7.19171	9.76428	5.20423
C	11.94221	8.57491	3.53352
C	4.60647	8.63816	4.97581
C	3.74449	9.02049	7.59970
C	3.08355	9.66157	6.54534
C	3.51612	9.47489	5.22685

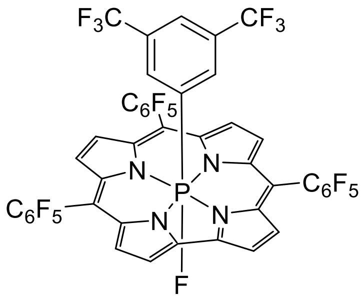

4•F

N	1.68290	4.84101	30.72925
N	-0.33584	6.65974	30.46058
N	-1.68223	4.73816	29.29186
N	0.09503	3.14205	29.52699
C	2.50202	3.71346	30.85339
C	3.74850	4.13677	31.39664
H	4.57848	3.48129	31.60119
C	3.70408	5.48659	31.56140
H	4.48658	6.12487	31.93518
C	2.41598	5.93452	31.14900
C	1.92916	7.23783	31.22341
C	2.82673	8.30621	31.74795
C	3.83721	8.87395	30.97302
F	4.01040	8.47941	29.70808
C	4.68729	9.85295	31.47169
F	5.64731	10.37382	30.70634
C	4.53363	10.29407	32.77910
F	5.34178	11.23203	33.26689
C	3.53450	9.75379	33.57718
F	3.38751	10.17280	34.83428
C	2.70189	8.77348	33.05623
F	1.75840	8.26580	33.85533
C	0.61433	7.56240	30.89873
C	0.00299	8.84402	31.01822
H	0.51026	9.73997	31.33353
C	-1.30470	8.71631	30.66626
H	-2.05240	9.49124	30.63904
C	-1.53235	7.36303	30.28454
C	-2.70671	6.86573	29.73227
C	-3.91522	7.73111	29.63576
C	-4.89473	7.71797	30.62735
F	-4.73754	6.94963	31.70883
C	-6.04918	8.48326	30.53301
F	-6.97128	8.44368	31.49519
C	-6.24391	9.29241	29.42171
F	-7.34610	10.03112	29.32191
C	-5.28681	9.32993	28.41638
F	-5.47591	10.10263	27.34575
C	-4.14060	8.55555	28.53517
F	-3.23735	8.61487	27.55222
C	-2.75750	5.57641	29.20189

C	-3.76495	4.89690	28.46287
H	-4.73366	5.30138	28.21944
C	-3.27210	3.65662	28.10608
H	-3.77704	2.89105	27.54060
C	-1.97017	3.57004	28.63763
C	-0.93320	2.63726	28.77657
C	-0.65228	1.29143	28.46814
H	-1.27571	0.63133	27.88803
C	0.55707	0.99150	29.06439
H	1.07637	0.04786	29.03130
C	1.02659	2.16136	29.72291
C	2.20234	2.42459	30.42586
C	3.14971	1.31087	30.70583
C	3.15946	0.68888	31.95376
F	2.32061	1.10389	32.90723
C	4.01152	-0.36624	32.24730
F	3.99506	-0.93837	33.45150
C	4.88360	-0.82987	31.27146
F	5.70209	-1.84448	31.53713
C	4.89902	-0.23449	30.01724
F	5.74083	-0.68002	29.08341
C	4.03853	0.82271	29.74976
F	4.08390	1.37785	28.53482
C	-0.28449	5.38429	35.42173
C	-0.78584	4.63880	34.21022
C	-1.74947	3.64825	34.34070
H	-2.13410	3.37607	35.31188
C	-2.20599	3.01669	33.19364
C	-3.24907	1.93173	33.27513
C	-1.70864	3.36695	31.94087
H	-2.09814	2.84474	31.07926
C	-0.74103	4.36053	31.79480
C	-0.29139	4.98884	32.95800
H	0.45593	5.76669	32.91128
P	-0.05225	4.86804	30.09196
F	0.57119	5.35274	28.64921
F	-2.77045	0.74585	32.83413
F	-4.32558	2.21736	32.50877
F	-3.69490	1.73703	34.52940
F	-0.76670	6.64658	35.45918
F	1.06153	5.48338	35.42751
F	-0.64494	4.79339	36.57629

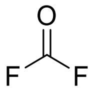

CF_2O (def2-TZVP)

F	1.02054	0.05185	0.27277
C	-0.23765	-0.02128	0.60608
O	-0.65748	-0.06149	1.69029

F	-0.93419	-0.04412	-0.49582

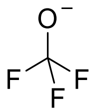

CF_3O (def2-TZVP)

F	0.93084	0.75207	0.49568
C	-0.17930	-0.10935	0.45464
O	-0.56719	-0.56402	1.51115
F	-1.08878	0.66831	-0.28203
F	0.23809	-1.03235	-0.52104

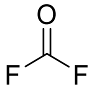

CF_2O (def2-SVP)

F	1.02233	0.05228	0.27347
C	-0.23548	-0.02226	0.60368
O	-0.65932	-0.06115	1.69050
F	-0.93632	-0.04392	-0.49433

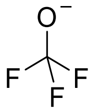

CF_3O (def2-SVP)

F	0.93084	0.75207	0.49568
C	-0.17930	-0.10935	0.45464
O	-0.56719	-0.56402	1.51115
F	-1.08878	0.66831	-0.28203
F	0.23809	-1.03235	-0.52104

12. References

[1] D. T. Gryko, B. Koszarna, *Org. Biomol. Chem.* **2003**, *1*, 350–357.
[2] P. Wucher, J. B. Schwaderer, S. Mecking, *ACS Catal.* **2014**, *4*, 2672–2679.
[3] E. Ihara, Young Victor G., R. F. Jordan, *J. Am. Chem. Soc.* **1998**, *120*, 8277–8278.
[4] S. Murarka, C. Zhang, M. D. Konieczynska, D. Seidel, *Org. Lett.* **2009**, *11*, 129–132.
[5] B. Koszarna, D. T. Gryko, *J. Org. Chem.* **2006**, *71*, 3707–3717.
[6] D. A. Evans, K. T. Chapman, *Tet. Lett.* **1986**, *27*, 5939–5942.
[7] L. L. Adduci, M. P. McLaughlin, T. A. Bender, J. J. Becker, M. R. Gagné, *Angew. Chem. Int. Ed.* **2014**, *53*, 1646–1649.
[8] K. O. Christe, D. A. Dixon, D. McLemore, W. W. Wilson, J. A. Sheehy, J. A. Boatz, *J. Fluorine Chem.* **2000**, *101*, 151–153.
